# Novel fidaxomicin antibiotics through site-selective catalysis

**DOI:** 10.1038/s42004-021-00501-6

**Published:** 2021-05-10

**Authors:** David Dailler, Andrea Dorst, Daniel Schäfle, Peter Sander, Karl Gademann

**Affiliations:** 1 Department of Chemistry, University of Zurich, Zurich, Switzerland; 2 Institute of Medical Microbiology, University of Zurich, Zurich, Switzerland; 3 National Center for Mycobacteria, University of Zurich, Zurich, Switzerland

**Keywords:** Carbohydrate chemistry, Pharmaceutics, Structure-based drug design, Cryoelectron microscopy

## Abstract

Fidaxomicin (FDX) is a marketed antibiotic for the treatment of *Clostridioides difficile* infections (CDI). Fidaxomicin displays antibacterial properties against many Gram-positive bacteria, yet the application of this antibiotic is currently limited to treatment of CDI. Semisynthetic modifications present a promising strategy to improve its pharmacokinetic properties and also circumvent resistance development by broadening the structural diversity of the derivatives. Here, based on a rational design using cryo-EM structural analysis, we implement two strategic site-selective catalytic reactions with a special emphasis to study the role of the carbohydrate units. Site-selective introduction of various ester moieties on the noviose as well as a Tsuji–Trost type rhamnose cleavage allow the synthesis of novel fidaxomicin analogs with promising antibacterial activities against *C. difficile* and *Mycobacterium tuberculosis*.

## Abstract (web summary)

Fidaxomicin is a narrow spectrum antibiotic, and broadening its activity through structural modification could provide new antibiotics. Here semi-synthetic derivatives are prepared through site-selective esterification and allylic substitution to efficiently modify or substitute key carbohydrate moieties.

## Introduction

Fidaxomicin (**1**, tiacumicin B, lipiarmycin A3) constitutes an 18-membered narrow-spectrum macrolide antibiotic that was extracted from several actinomycetes^1–7^ and shows antibacterial properties against mainly Gram-positive bacteria^8^, in particular against methicillin-resistant *Staphylococcus aureus*^9^, multidrug-resistant *Mycobacterium tuberculosis*^7^, and *Clostridioides difficile*^10–12^. To date, the clinical use is limited to the treatment of *C. difficile* infections, the main cause of hospital-acquired diarrhea, against which fidaxomicin was approved by the FDA in 2011^13–17^. Fidaxomicin inhibits the RNA polymerase (RNAP) via a new mechanism of action, rendering second-generation derivatives thereof particularly attractive^18–20^. Resistance development in bacteria accelerated over the last few decades and new mechanisms of resistance have emerged, which calls for the development of antibiotics with unique mechanisms of action^21^.

Globally, an estimated 10.0 million people fell ill with tuberculosis (TB) in 2019 and more than 1 million people died of it. Drug-resistant TB continues to be a public health threat. Almost half a million people developed rifampicin-resistant TB (RR-TB), of which 78% were multidrug resistant (MDR)^22^. Multidrug-resistant *M. tuberculosis* strains exhibit resistance toward the two most effective drugs, isoniazid and rifampicin, of the four drug standard treatment regimen. Alarmingly, in some countries, more than 50% of previously treated TB cases had MDR/RR-TB. Patients suffering from MDR-TB require a treatment with second-line drugs, which are less effective, more toxic, and more expensive. New treatment regimens combining compounds with novel mode of actions like bedaquiline^23^ and repurposed drugs (linezolid) are currently evaluated for the treatment of MDR-TB^24^. However, resistant strains were detected soon after approval of novel drugs^25^. These findings indicate that the TB drug-pipeline has to be fueled continuously.

**Fig. 1:**
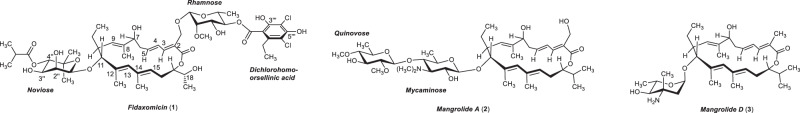
Glycosylated macrocyclic antibiotics. Structures of fidaxomicin (**1**), mangrolide A (**2**), and D (**3**).

Susceptibility of *C. difficile* to fidaxomicin remains generally very high in the clinic, although there have been very few cases of fidaxomicin-resistant strains emerging^26,27^, which calls for the development of second-generation derivatives. Although fidaxomicin has a high potential to deliver next-generation antibiotics, there are only few successful approaches toward new derivatives documented that might broaden structure–activity relationship (SAR) information and thereby further extend its application^18,28,29^. So far, most known derivatives were obtained through fermentation of gene-knockout mutants of the producer strain or by chemical modification of the homoorsellinic acid moiety^30–35^. Furthermore, several compounds with a similar scaffold were found along with fidaxomicin (**1**), some of which lack the rhamnose-orsellinate and/or the noviose moiety^8,36^. Those truncated products missing one of the sugar moieties display significantly decreased antibiotic activity and thereby point out the importance of these carbohydrate units^37^. Interestingly, nature also provides similar scaffolds from other species, such as mangrolide A (**2**) and D (**3**) that possess different sugar moieties and a similar macrolactone (Fig. 1)^38,39^. Total syntheses campaigns of these compounds were started and successfully accomplished, but not answering the intriguing question on the role of the carbohydrate units^40–44^.

Over the past few decades, derivatization of natural products has been demonstrated to be an effective approach for the development of new drug candidates^21^. However, the direct and selective functionalization of these scaffolds to gain specific SAR information still remains challenging due to their structural complexity and broad variety of functional groups. In order to address these issues, chemo- and site-selective functionalization recently emerged as a particularly appealing approach^45^. Especially, site-selective transition-metal catalysis and organocatalysis started to find widespread application in derivatization of natural products^46^. In this study, we report on the generation of novel fidaxomicin derivatives through site-selective catalysis using a rational design based on a cryo-electron microscopy (cryo-EM) structure of fidaxomicin binding to *M. tuberculosis* RNAP^18,19^. In this context, semisynthetic strategies for the modification of fidaxomicin on the noviose- as well as on the rhamnose-orsellinate moiety were developed: (1) the application of site-selective organocatalysis for the generation of a library of 3”-acylated fidaxomicin derivatives and (2) a palladium-catalyzed allylic substitution for the selective cleavage of the rhamnose-resorcylate moiety. By these strategies, potent fidaxomicin derivatives that cannot be obtained by genetic modification could be prepared by chemical synthesis.

## Results and discussion

### Design based on cryo-EM structural analysis

**Fig. 2:**
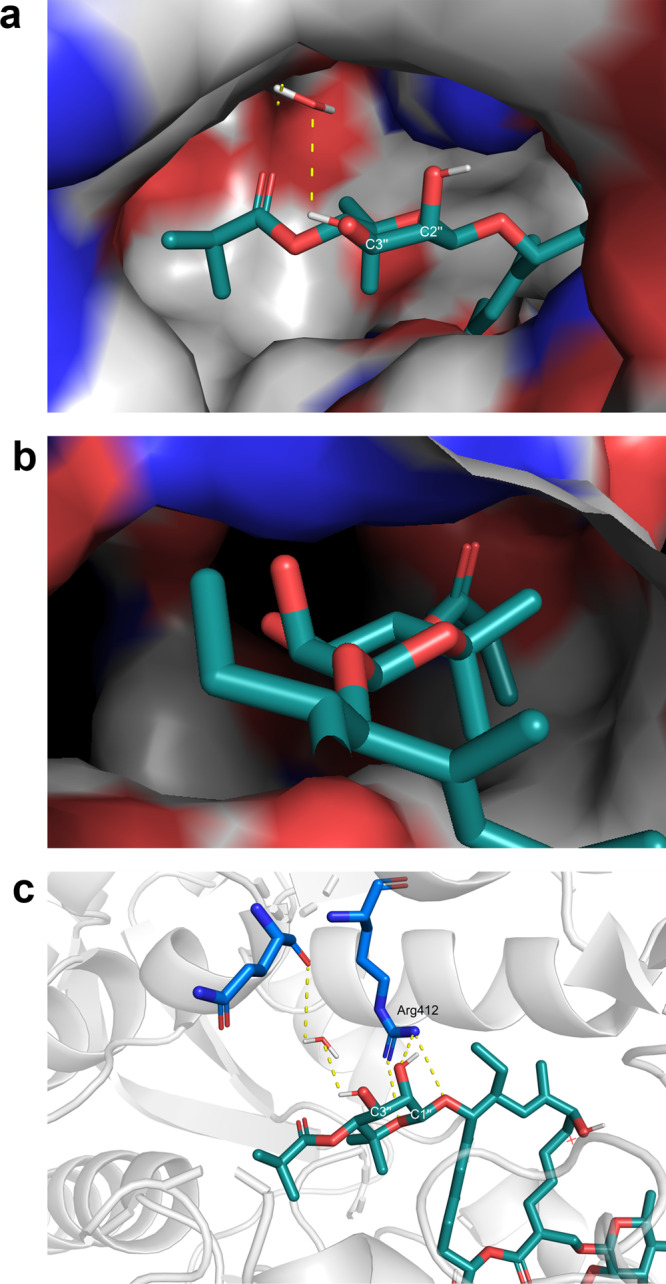
Cryo-EM structure of fidaxomicin binding to *M. tuberculosis* RNAP (PDB ID: 6FBV)^18^. **a** Fidaxomicin (cyan), protein (surface representation/gray). Interaction of C3”-OH with H_2_O. Limited space around isobutyric ester moiety. **b** Detailed view on the binding pocket of fidaxomicin’s noviose moiety. C3”-OH points toward an “open space.” C4”-isobutyric ester fits into its pocket. **c** Detailed view on the interactions of the noviose part to the protein. C2”-OH is blocked by an interaction with Arg412 (blue).

Previous studies on the fermentation conditions led to the isolation of several fidaxomicin congeners with different ester moieties in various positions on the noviose moiety^47^. These analogs displayed decreased antibiotic activities compared to the isobutyric ester moiety in 4”-position of the parent compound. Intriguingly, isobutyric ester migration to 3”-position (tiacumicin F) did not lead to a significant decrease of activity^3,5,8^, which is in agreement with the recently disclosed cryo-EM structure. Indeed, there is an “open space” toward the C3”-hydroxy group that allows the introduction of functional groups (Fig. 2a, b). In contrast, the C4”-isobutyric ester fits perfectly into this pocket, but the space for further modifications in this position is limited. Moreover, potential interaction with or replacement of water in the active site constitutes a promising strategy that has already been demonstrated to be beneficial in drug design^48–50^. Finally, the C2”-hydroxy group is involved in multiple hydrogen-bonding interactions between Arg412 and acetal of the noviose moiety (Fig. 2c)^18,19^. Therefore, we expect C2”-acylation of fidaxomicin derivatives to have a detrimental effect on their bioactivity. Thus, we hypothesized that modifications on the C3”-hydroxy group of fidaxomicin constitute a viable design strategy that could lead to retained or enhanced bioactivity.

**Fig. 3:**
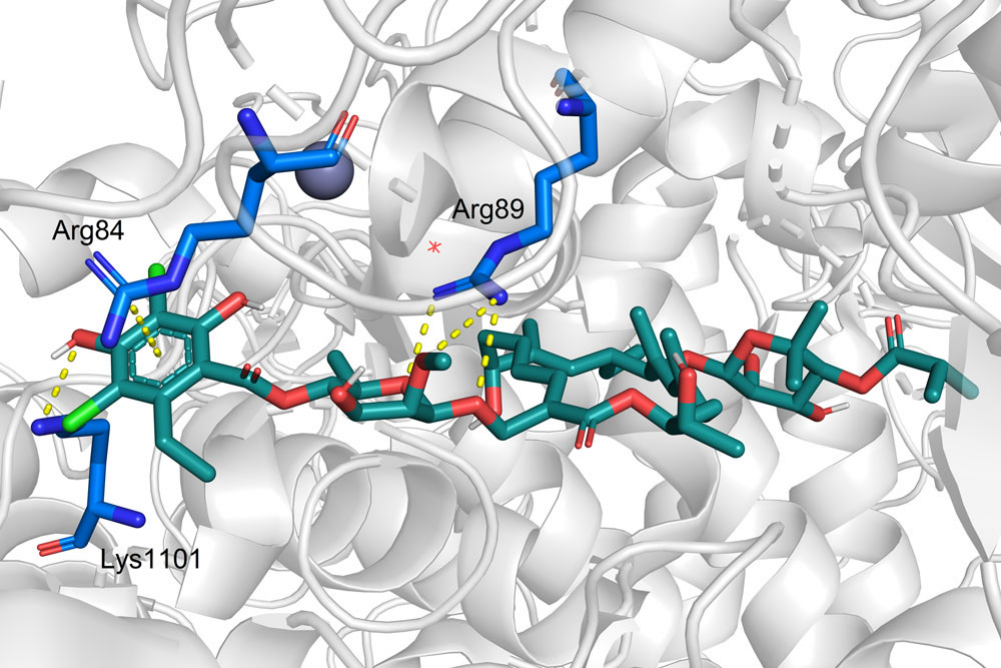
Cryo-EM structure of fidaxomicin binding to *M. tuberculosis* RNAP (PDB ID: 6FBV)^18^. Fidaxomicin (cyan), protein (gray), and amino acid residues (blue). Detailed view on the interactions of the rhamnose-resorcylate part to the protein.

On the other part of fidaxomicin, several hydrogen-bonding interactions of Arg89 with the rhamnose moiety and Lys1101 with the phenolic hydroxy group on the homoorsellinic acid moiety as well as a cation-π interaction of Arg84 with the aromatic moiety are present (Fig. 3). Therefore, we aimed to study the influence of variations on the rhamnose-resorcylate moiety by modifying this part of the lead structure.

### Modifications on the noviose unit

**Fig. 4:**
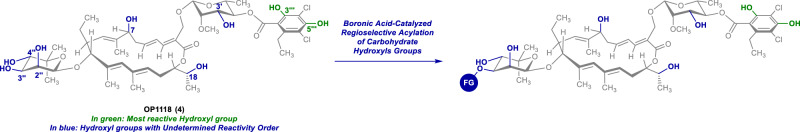
Site-selective acylation of 4. Overview of the strategy.

As discussed above, careful analysis of the cryo-EM structure of fidaxomicin binding to *M. tuberculosis* RNAP and previously reported bioactivities prompted us to consider selective C3”-acylation of the noviose moiety to gain SAR and potentially identify novel lead structures for drug discovery. Given the nature and high number of hydroxy groups in OP1118 (**4**), a degradation metabolite of fidaxomicin (**1**), as well as the lack of data concerning their reactivities and the chemical fragility of fidaxomicin, site-selective functionalization of C3”-OH represents an interesting challenge (Fig. 4)^51^.

**Fig. 5:**
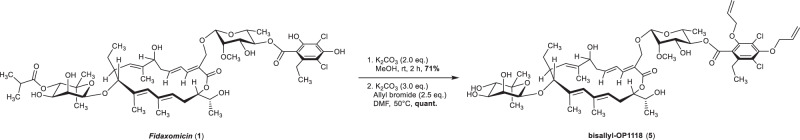
Preparation of the catalysis-precursor bisallyl-OP1118 (5). Synthesis of **5** from natural product **1** via isobutyl ester hydrolysis and allyl protection.

In order to address this reactivity challenge, we envisioned to apply non-enzymatic methodology^52^. Over the past 15 years, extensive research efforts led to the development of efficient and complementary synthetic methods to access regioselective functionalization of carbohydrate hydroxy groups^53^. Analysis of the structural feature of fidaxomicin allowed us to identify the *cis*-vicinal diol as a potential platform for catalysis based on reversible covalent interactions of organoboron compounds. Indeed, this approach enabled by molecular recognition has proven to be effective for various transformations^54,55^. In addition to provide high regioselectivity, this strategy would also potentially outcompete the initially more reactive secondary hydroxy groups (C7-OH/C18-OH) through activation of the *cis*-vicinal diol via tetracoordinate borate, therefore avoiding undesired over-acylation^56^. In order to study the feasibility of this transformation, we thus started with the synthesis of the required precursor for catalysis. Considering the inherently higher nucleophilicity of phenol moiety, we thus turned our attention to the synthesis of bisallyl-OP1118 (**5**) (Fig. 5). Starting from commercially available fidaxomicin (**1**), cleavage of the isobutyrate ester via methanolysis followed by bisallyl protection of the phenol moiety afforded the starting compound for catalysis in 71% over two steps.

**Fig. 6:**
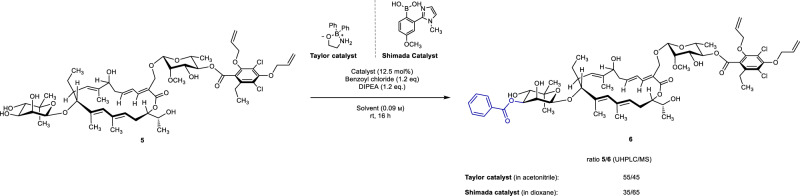
C3”-OH acylation of 5. Initial catalyst evaluation to access C3”-acylated fidaxomicin derivatives.

With the required starting material in hand, we turned our attention to the screening of initially described ethanolamine ester of diphenylborinic acid catalyst (Taylor’s catalyst)^56^ along with a boronic acid catalyst recently developed by Shimada et al.^57^. Under classic conditions, both catalysts afforded conversion to the desired product (Fig. 6).

**Fig. 7:**
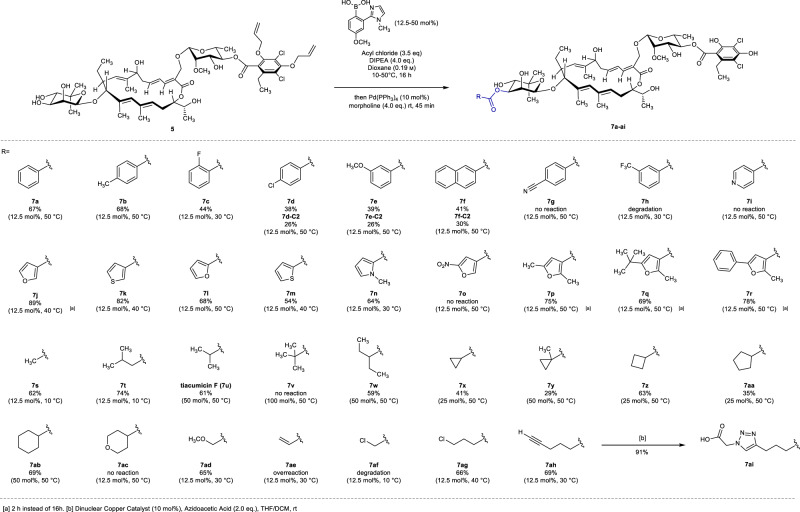
Scope of selective C3”-functionalization of 5. Acylation of **5** applying the optimized catalyst system with various acyl chlorides.

Encouraged by this finding, we therefore optimized the reaction with the Shimada catalyst since it offered slightly higher catalytic activity in these initial experiments. After screening of all the parameters of the reaction, we were able to obtain full conversion (UHPLC/MS analysis) to the desired compound **7** (confirmed by 2D NMR analysis; see Supplementary Fig. 10, section NMR Spectra) with the conditions depicted in Fig. 7.

Noteworthy, control experiments without catalyst confirming its presence are essential to ensure high conversion and site-selectivity. Moreover, the temperature (50 °C) is elevated compared to previously reported conditions; this is crucial to achieve complete conversion, which might arise from steric hindrance or conformational aspects imputed to the *gem*-dimethyl moiety of the noviose. Finally, subsequent deprotection of the allyl moiety in a one-pot two-step manner allowed us to obtain the desired product **7** in 67% yield (for experimental details refer to the Supplementary Methods). As expected, attempts to perform this selective C3”-acylation directly with the compound OP1118 (**4**) led to the functionalization of the dichlorohomo-orsellinic acid moiety. Furthermore, experiments with bisallyl-fidaxomicin^51^ were performed in order to study C4”-C3”-diacylated fidaxomicin derivatives. Unfortunately, we were not able to observe any traces of the desired functionalization even with stoichiometric amount of the organocatalyst. This could be explained by the steric hindrance caused by the C4”-isobutyrate moiety close to the reactive site. With the optimized conditions in hand, we turned our attention to the delivery of a library of C3”-acylated fidaxomicin derivatives. First, benzoyl chloride derivatives with different substitution patterns were screened. We were pleased to find that *ortho*-, *meta*-, and *para*-substitutions are tolerated, in addition to electron withdrawing and electron donating groups **7a–f**. Moreover, in some cases (**7d–f**), concomitant formation of C2”-benzoylated fidaxomicin derivatives (confirmed by 2D NMR analysis; see Supplementary Fig. 28) were observed in not negligible amount. Presumably, the C2”-benzoylated compounds arise from competing activation of the second hydroxy group of the *cis*-vicinal diol rather than benzoyl migration, as resubmission of the C3”-acylated fidaxomicin to the reaction conditions did not lead to any isomerization. Gratifyingly, the C2”/C3”-benzoylated fidaxomicin mixture was separable by preparative HPLC purification, allowing us to gain more SAR information. Heteroaromatic scaffolds were also introduced successfully with almost exclusive regioselectivity to afford compounds **7j–n** in medium to high yield, except the pyridine moiety **7i**. Preliminary interesting biological activities for the 3-furoyl derivatives **7j** led us to explore the impact of the substitution of the latter, leading to structures **7o–r**. In consideration of the bioactivities of tiacumicin F (**7u**), we envisioned to study non-aromatic C3”-acylated fidaxomicin derivatives. While C3”-acylated fidaxomicin derivatives **7s–t** were obtained in good yield at 10 °C to control overreaction and degradation (THF ring formation)^51^, increase of substitution at carbon α to the carbonyl led to degradation of the reactivity. This highlighted the crowded environment of the noviose, which is in line with previous observations made during the optimization of the reaction conditions. To ensure generation of sufficient amounts for subsequent biological evaluation, we therefore increased the amount of Shimada catalyst in the most difficult cases to afford tiacumicin F (**7u**) and compounds **7w**–**ab** in moderate to excellent yields. Notably, stoichiometric amount of Shimada catalyst provides only slight trace of the desired compound **7v** with pivaloyl chloride. In addition, some functionalities were successfully introduced, such as methoxy **7ad**, chloride **7ag**, or terminal alkyne **7ah**. Noteworthy, this latter represents a unique platform for introduction of various triazole moiety through copper(I)-catalyzed alkyne-azide cycloaddition (CuAAC). Gratifyingly, dinuclear copper catalyst **21** developed by Straub et al.^58^ allowed the formation of derivatives **7ai** in excellent yield. Finally, in order to study the impact of other functional groups on the biological activity, further electrophiles such as carbamoyl chloride, chloroformate, and tosylate were tested under our optimized conditions. Unfortunately, all attempts were found to be unproductive.

### Biological evaluation of C3”-acylated fidaxomicin derivatives

**Fig. 8:**
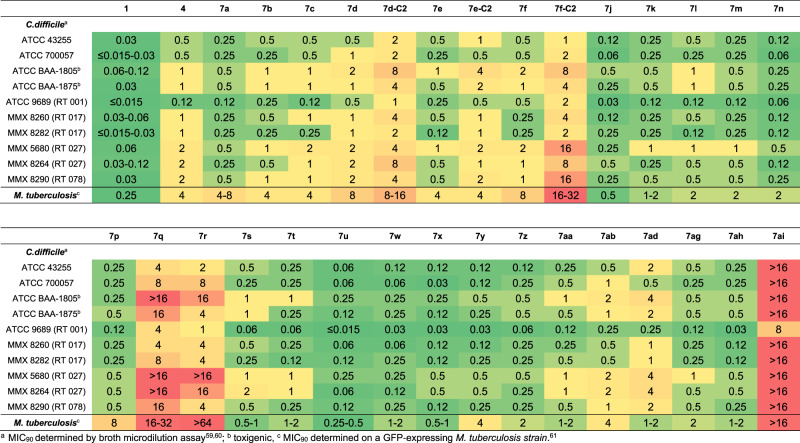
Minimum inhibitory concentrations (MIC) values. Determination of the MIC in µg/mL against a panel of different *C. difficile* strains and *M. tuberculosis*. RT Ribotype.

The biological activities for these 30 fidaxomicin analogs were evaluated by determination of the minimum inhibitory concentration (MIC) and are summarized in Fig. 8. The MIC values for *C. difficile* were determined using the broth microdilution assay and determination of bacterial growth by observation of turbitity (MIC_90_)^59,60^. The values against *M. tuberculosis* were evaluated using a recently developed method on a GFP-expressing *M. tuberculosis* strain and observation of growth by fluorescence^61^. A fluorescence reduction of 90% as compared to the no-drug control was reported as MIC_90_ (for details see Supplementary Information, section Determination of the minimum inhibitory concentration). All compounds were evaluated against *M. tuberculosis* as well as ten *C. difficile* isolates. As expected with previously reported data, OP1118 (**4**) displayed reduced antibacterial activity, pointing out the pivotal role of the isobutyric ester moiety for the bioactivity. Intriguingly, aromatic derivatives **7a–7f** showed similar bioactivities than for OP1118 (**4**). Interestingly, C2”-benzoylated derivatives **7d–f–C2** were found to be less active than the C3”-benzoylated analogs that supports hypotheses drawn from the cryo-EM structural analysis. To our delight, heteroaromatic derivatives **7j–n** were found to have good bioactivity for all the tested bacterial strains compared to the aromatic series **7a** to **7f–C2**. Noteworthy, derivative **7j** is highly active against *M. tuberculosis* (MIC = 0.5 µg/mL) as well as the *C. difficile* strains; therefore, the latter represents an excellent landmark for implementation of substitution on the furan moiety. Unfortunately, first decorated versions **7p–r** of the more potent heteroaromatic derivative **7j** were found to be less active, such as the derivatives **7q–r**, which are sterically more demanding. Nevertheless, this first batch of substituted furan derivatives indicated that smaller functional groups and/or functionalizations at the position 4 of the furan should be considered in the future for the modification of derivative **7r**. On the other hand, considering the reported bioactivities of tiacumicin F (**7u**), non-aromatic C3”-acylated fidaxomicin derivatives were expected to have similar or improved bioactivities. Indeed, fidaxomicin analogs **7s-ah** were found to have good bioactivity, and especially, derivatives **7w–x**. Whereas derivatives **7j**, **7u**, and **7x** have excellent bioactivities against all tested bacterial strains, these are slightly less potent than fidaxomicin itself. Nevertheless, this study validates our hypothesis and a fine-tuning of those derivatives may pave the way to discover more potent antibiotic derivatives.

### Allylic substitution of the rhamnose-resorcylate moiety

**Fig. 9:**
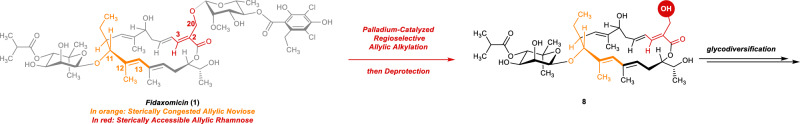
Site-selective allylic functionalization of (1). Overview of the strategy.

Over the past 50 years, metal-catalyzed allylic (asymmetric) substitution emerged as an extremely appealing and efficient tool to form C-C and C-heteroatom bonds with applications in target-oriented synthesis of natural products and active pharmaceutical ingredients^62^. Whereas numerous developments allow the use of a broad range of allylic substrates, the use of allylic acetals as allyl donors remains scarce and were used as carbon sources in palladium-catalyzed umpolung allylations^63,64^. Nonetheless, these reports led us to consider the allylic noviose and the allylic rhamnose of fidaxomicin as potential electrophilic partner in metal-catalyzed allylic substitution. Considering the steric congestion around the allylic noviose moiety as well as the electronically favored environment around the rhamnose due to the proximity to the conjugated macrolactone, we envisioned that discrimination of the allylic rhamnose versus allylic noviose would be possible. Moreover, using an appropriate oxygen nucleophile would lead, after deprotection, to **8** that represents a unique platform for glycodiversification (Fig. 9).

**Fig. 10:**
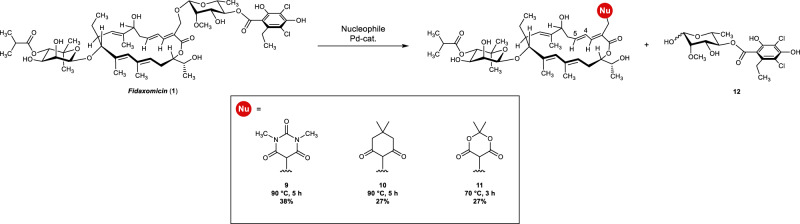
Allylic substitution of the rhamnose-resorcylate moiety. Scope of the site-selective allylic functionalization of (**1**).

Unfortunately, extensive screening with differently protected *O*-nucleophiles remained unsuccessful as either no conversion or complex mixtures were obtained. This prompted us to consider other types of nucleophiles. To our delight, we were pleased to find that *C-*nucleophiles were competent, thus validating our strategy (Fig. 10). Cyclic 1,3-dicarbonyls, such as **9**, **10**, and **11** were synthesized with concomitant isomerization of the diene moiety (between C4 and C5), which is in line with the ability of palladium to migrate along a π system^65^. Whereas substitution with *N*,*N*-dimethyl barbituric acid and dimedone required temperatures of 90 °C, the reaction with Meldrum’s acid led to decomposition; however, lowering the temperature to 70 °C was beneficial to provide the desired product **11** in 27% yield. Unfortunately, extensive screening of the parameters of the reaction with special emphasis on phosphine ligand did not improve the reaction further and isomerization of the double bond could not be prevented. Noteworthy, we ruled out a plausible Baylis–Hillman type mechanism by control experiments (see Supplementary Information, section Experimental procedures Tsuji–Trost functionalizations).

**Fig. 11:**
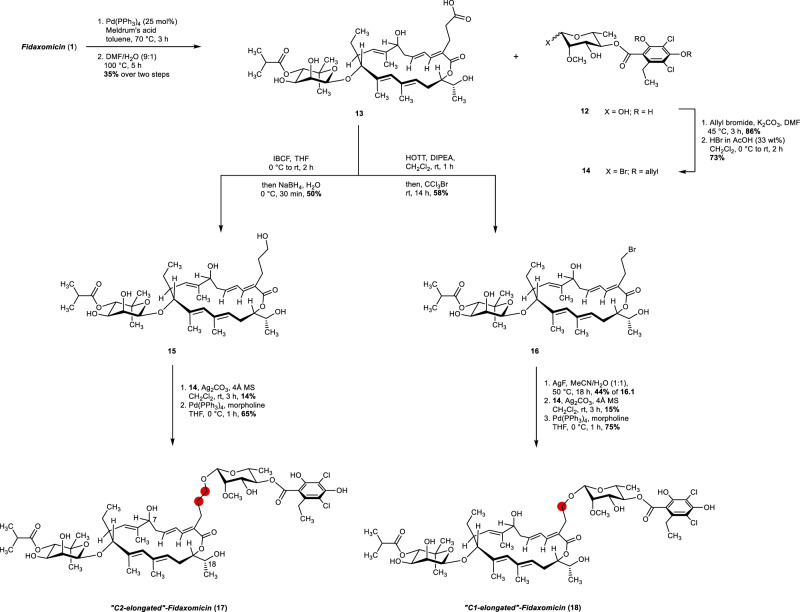
Synthesis of C2- and C1-elongated fidaxomicin analogs. Detailed synthetic route toward C2- and C1-elongated fidaxomicin analogs **17** and **18**. IBCF Isobutyl chloroformate, HOTT S-(1-oxido-2-pyridinyl)−1,1,3,3-tetramethylthiouronium hexafluorophosphate.

Therefore, to best of our knowledge, this transformation represents a unique palladium-catalyzed allylic substitution with such a complex electrophilic partner. Moreover, given the abundance of allylic glycoside derivatives in biologically active natural products (especially in polyene macrolides), we believe that this finding will pave the way for further development in drug discovery^66^. Next, we decided to take advantage of the Meldrum’s acid derivative **11**. Upon hydrolysis and decarboxylation of the Meldrum’s acid moiety, carboxylic acid **13** was obtained in 35% yield over two steps (Fig. 11). Usually, Meldrum’s acid **11** was directly submitted to hydrolysis without further purification due to the difficult separation of **11** from its *E*,*Z*-isomer, which is formed in 12% yield as a by-product of the Tsuji–Trost reaction. The carboxylic acid **13** acts as suitable functional group for a great variety of transformations such as esterification/amide coupling, reduction, Curtius rearrangement, or decarboxylative brominations and consequently allowing the synthesis of analogs with one or two additional CH_2_-groups, which are difficult or not even possible to obtain by fermentation of a genetically modified producer strain.

As we were able to regain high quantities of the cleaved rhamnose-orsellinate moiety **12** after the Tsuji–Trost reaction, we prepared C2- and C1-elongated fidaxomicin analogs, starting from fidaxomicin (**1**). Therefore, we addressed the selective reduction of the carboxylic acid **13** next. Upon screening of several conditions, we found that formation of the mixed anhydride using isobutyl chloroformate and subsequent reduction with NaBH_4_ yields the desired alcohol **15**, besides some traces of competing isobutyl ester cleavage on the noviose part^67^.

Transformation of the rhamnosyl-orsellinate **12** into a suitable glycosyl donor was achieved via allyl protection of the phenolic hydroxy groups, followed by substitution of the anomeric hydroxy group by bromide using HBr to give glycosyl bromide **14** as a single anomer. With glycosyl bromide **14** and alcohol **15** in hands, we performed the glycosylation using Königs–Knorr conditions^68^ that gave after allyl deprotection, the desired C2-elongated fidaxomicin **17**. Besides glycosylation on the primary alcohol, minor amounts of glycosylation at C7- and C18 hydroxy groups were observed as well, which accounts for the relatively low yield. The desired C2-elongated fidaxomicin **17** was surprisingly obtained as a single anomer. NOESY spectra as well as similar ^1^J_CH_ coupling constants (**1**: ^1^J_CH_ = 155 Hz, **17**: ^1^J_CH_ = 157 Hz) indicate that most probably β-glycosylation occurred^69,70^. NOESY analysis of the glycosyl bromide **14**, however, revealed that the α-anomer was obtained as the major isomer, indicating an S_N_2-type mechanism (see Supplementary Figs. 1, 88, 100, and 101)

Furthermore, we investigated decarboxylative brominations via a Barton ester to give bromide **16**. Best yields were achieved when Barton ester formation was performed using the coupling reagent HOTT^71–73^ directly followed by addition of bromotrichloromethane^74^. The following hydrolysis of bromide **16** was found to be troublesome^75^. Various by-products that originate from transesterifications on the macrolactone were observed and made purification difficult. Upon optimization, yields of up to 44% of the desired alcohol were obtained. Final glycosylation reaction, which had to be warmed to 40 °C in order to get complete conversion, and allyl deprotection then yielded the C1-elongated fidaxomicin **18**.

**Fig. 12:**
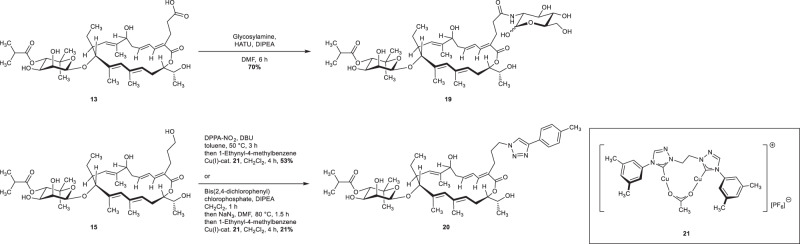
Functionalization of C2-elongated carboxylic acid 13 and alcohol 15. Synthesis of glucosamide-fidaxomicin **19** and triazol **20**. HATU 1-[Bis(dimethylamino)methylene]−1H-1,2,3-triazolo[4,5-b]pyridinium 3-oxide hexafluorophosphate, DPPA-NO_2_Di-(nitrophenyl)phosphoryl azide.

As already outlined before, the carboxylic acid **13** as well as the alcohol **15** and **16.1** can serve as a platform for further modifications. As we are interested in the role of the sugar moieties on fidaxomicin, we investigated displacement by other sugar moieties on this stage. As a suitable carbohydrate, we choose glycosylamine, which could be coupled to the carboxylic acid moiety using HATU to afford the desired glycosylamide **19** in high yields (Fig. 12). On the another hand, we envisioned to introduce 1,2,3-triazole scaffolds, which are relatively stable to hydrolytic conditions, metabolic degradation, and redox conditions. Moreover, they are known to be bioisosteres of numerous functional groups and they are prone to H-bonds and π–π stackings interactions. All these features rendering 1,2,3-triazole ring highly attractive to access lead compounds in medicinal chemistry^76–78^. Whereas activation of the alcohol **15** through sulfonate formation followed by sodium azide displacement was unselective, we found out that the one-pot azidation of alcohol **15** using bis (*p*-nitrophenyl) phosphorazidate was highly selective for the primary alcohol^79^. Subsequent Cu-mediated cycloaddition delivered the triazole **20** in good overall yield. Unfortunately, reproducibility issues with the azidation step prompt us to consider another sequence. After optimization, the alcohol **15** was fully converted to the desired phosphate using bis-(2,4-dichlorophenyl) chlorophosphate. Upon S_N_2-type mechanism with sodium azide followed by previously used CuAAC with 1-ethynyl-4-methylbenzene, the triazole **20** was obtained in an acceptable yield. Gratifyingly, due to the highly selective activation of the primary alcohol of **15**, this strategy would allow us to obtain an expeditious library of C2-elongated fidaxomicin analogs via S_N_2-type mechanism by varying the nucleophile.

### Biological evaluation of rhamnose-edited fidaxomicin derivatives

**Fig. 13:**
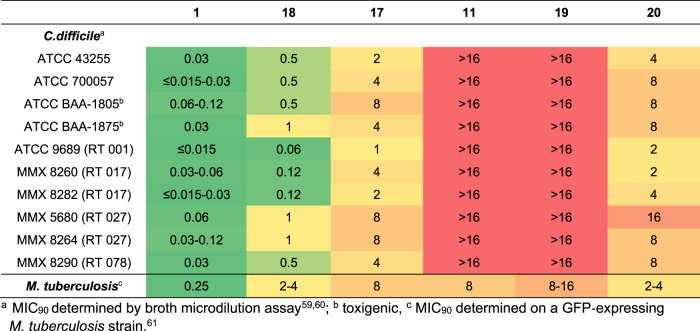
Minimum inhibitory concentration (MIC) values. Determination of the MIC in µg/mL against a panel of different *C. difficile* strains and *M. tuberculosis*. RT Ribotype.

The biological activities for these compounds bearing variations on the rhamnose-homoorsellinate part are summarized in Fig. 13. While fidaxomicin (**1**) is highly active against *M. tuberculosis* (MIC = 0.25 µg/mL) and *C. difficile*, the activity for the C1- and C2-elongated derivatives gradually drops, thereby indicating that binding to the RNAP is diminished probably due to a reduced interaction of the rhamnose sugar with Arg89. Therefore, we conclude that the position of the rhamnose-resorcylate moiety is crucial for the excellent activity of fidaxomicin (**1**) due to optimal interactions with amino acid residues in the binding pocket.

Interestingly, derivatives **11** and **19**, where the rhamnose moiety is exchanged by Meldrum’s acid and glycosylamine, respectively, are still active against *M. tuberculosis*, even though the activity significantly decreased. However, activity against *C. difficile* was lost. To our delight, triazole **20**, whose structure considerably differs from fidaxomicin, still features good activity against *M. tuberculosis*.

## Conclusion

In conclusion, we achieved the synthesis of a variety of novel fidaxomicin derivatives based on structural information provided by cryo-EM structures of fidaxomicin binding to the target bacterial RNAP. A highly site-selective method for the acylation of the noviose moiety using Shimada’s catalyst gave access to 30 novel fidaxomicin analogs with modifications on the 3”-OH group. These derivatives showed similar antibacterial activities compared to natural product **1** and thereby confirming our hypothesis that modifications in these positions are beneficial in retaining activity. In addition, our search for new reactions regarding fidaxomicin resulted in the development of a Tsuji–Trost type allylic substitution with cyclic 1,3-diketones to furnish rhamnose-resorcylate cleavage and thereby giving access to carboxylic acid **13** that would serve as a basis for a variety of glycodiversifications such as **19**. Furthermore, triazole **20** retained some antibacterial activity and therefore use of these azide precursors represents a promising platform for further derivatizations. At present, this Tsuji–Trost approach is limited to *C*-nucleophiles, but investigations to broaden the scope of this reaction are currently under investigation and will be reported in due course. Applying this reaction, C1- and C2-elongated fidaxomicin analogs **17** and **18** were synthesized, thus representing the first examples of a complex, multi-step modification of natural antibiotic fidaxomicin (**1**). These new fidaxomicin derivatives and further derivatives prepared by the methods described in this work may prove to be promising, future candidates for the treatment of bacterial infections and may contribute to ongoing efforts to reduce rate of resistance development.

## Methods

Full methods and data are given in the Supplementary Material, see Supplementary Methods section.

### Reporting summary

Further information on research design is available in the Nature Research Reporting Summary linked to this article.

## Supplementary information

Supplementary Information

Reporting Summary

## Data Availability

Compounds and the corresponding biological data were deposited at PubChem and PubChem BioAssay (SID 440785998–440786033). Procedures and analytical data, including NMR spectra, are available in the Supplementary Information or can be requested from the corresponding author.
