# Supplementary material for: Novel fidaxomicin antibiotics through site-selective catalysis
Source: Commun Chem. 2021 May 10;4:59. doi: 10.1038/s42004-021-00501-6 (PMC9814943; doi:10.1038/s42004-021-00501-6)
Supplement: Supplementary file 1 — Supplementary Information [file 42004_2021_501_MOESM1_ESM.pdf]

SUPPLEMENTARY INFORMATION

Novel Fidaxomicin Antibiotics through Site-Selective Catalysis

David Dailler[1†], Andrea Dorst[1†], Daniel Schäfle[2], Peter Sander[2], Karl Gademann[1]*

[1] Department of Chemistry, University of Zurich, Winterthurerstrasse 190, 8057 Zürich.
E-mail: karl.gademann@chem.uzh.ch

[2] Institute of Medical Microbiology, University of Zurich, Gloriastrasse 30/32, 8006 Zürich and National Laboratory for Mycobactery, Gloriastrasse 30, 8006 Zürich.

[†] these authors contributed equally

Table of Content

Supplementary Methods 3

General Methods and Materials 3

Experimental Procedures C3''-Functionalizations 5

Experimental Procedures Tsuji-Trost Functionalizations 37

Determination of the Minimum Inhibitory Concentration 55

Supplementary References 57

NMR Spectra 58

Supplementary Methods

General Methods and Materials

Unless otherwise stated, all chemicals were of reagent grade and purchased from *Sigma-Aldrich, Merck, Fluorochem* or *Honeywell*. Fidaxomicin was either obtained by fermentation of *Actinoplanes deccanensis* (ATCC 21983) or purchased from commercial suppliers (BOC Sciences). Reactions were carried out under protecting gas (N_2 or Ar) and, unless otherweise stated, monitored for completion by UHPLC-MS (ESI). Solvents for reactions were of p.a. grade. Evaporation of solvents *in vacuo* was carried out on a rotary evaporator at 40 °C bath temperature and appropriate pressure. **Ultra high performance liquid chromatography coupled to mass spectrometry** (UHPLC-MS): *Ultimate 3000 LC* instrument (*Thermo Fisher Scientific*) coupled to a triple quadrupole *Quantum Ultra EMR* MS (*Thermo Fisher Scientific*) using a reversed-phase column (*Kinetex*® EVO C18; 1.7 µm; 100 Å, 50 x 2.1 mm; *Phenomenex*). The LC was equipped with an *HPG-3400RS* pump, a *WPS-3000TRS* autosampler, a *TCC-3000RS* column oven and a *Vanquish DAD* detector (all *Thermo Fisher Scientific*). The following solvents were applied: H_2O + 0.1% HCOOH (A), MeCN + 0.1% HCOOH (B). Samples were prepared using HPLC grade solvents (MeCN, MeOH, H_2O) and filtered over a 4 mm syringe filter, PTFE (hydrophilic), pore size: 0.22 µm obtained from BGB Analytik AG. The MS was equipped with an H-ESI II ion source. The source temperature was 250 °C, the capillary temperature 270 °C and capillary voltage 3500 V, and datasets were acquired at resolution 0.7 on Q3 in centroid mode. **High performance liquid chromatography** (HPLC): All samples were pre-purified with a Discovery® DSC-18 SPE cartridge. *Prominence* modular HPLC instrument (*Shimadzu*) coupled to an *SPD-20A* UV/Vis detector (*Shimadzu*) using a reversed-phase column (*Gemini NX* C18, 3 µm, 10 Å, 150 mm x 4.6 mm) for analytical HPLC, and a reversed-phase column (*Gemini NX* C18, 5 µm, 110 Å, 250 mm × 21.2 mm; for preparative HPLC. The LC was equipped with a *CBM-20A* system controller, *LC-20A* solvent delivery unit, a *DGU-20A* degassing unit, *FRC-10A* fraction collector (all *Shimadzu*). The following solvents were used: H_2O + 0.1% HCOOH (A), MeCN + 0.1% HCOOH (B). **Specific optical rotation** $[\alpha]_{\mathrm{D}}^{\mathrm{T}}$: *Jasco P-2000 Polarimeter*; measured at the indicated temperature *T*. All given values for $[\alpha]_{\mathrm{D}}^{\mathrm{T}}$ have the dimension ° mL dm^{-1} g^{-1}. **Infrared spectra** (IR): *SpectrumTwo FT-IR Spectrometer* (*Perkin–Elmer*) equipped with a *Specac Golden Gate^{TM} ATR* (attenuated total reflection) accessory; applied as neat samples or as films; 1/λ in cm^{-1}. **Nuclear magnetic resonance spectra** (NMR): ^1H NMR spectra were recorded in $CDCl_3$ or acetone-d_6 on the instruments *AV-500* (500 MHz) or *AV-400* (400 MHz); chemical shift δ in ppm relative to solvent signals (δ = 7.26 ppm for $CDCl_3$, 2.05 ppm for acetone-d_6, 3.31 ppm for CD_3OD),[1] coupling constant *J* is given in Hz. ^{13}C NMR spectra were recorded in $CDCl_3$ or acetone-d_6 on the instruments *Bruker AV-500* (125 MHz) or *AV-400* (100 MHz); chemical shift in ppm relative to solvent signals (δ = 77.16 ppm for $CDCl_3$, 29.84 ppm for acetone-d_6, 49.00 ppm for CD_3OD).[1] **High-resolution electrospray ionization mass spectra** (HRMS): *On flow injection*: High-resolution mass spectra were acquired on a *Qexactive* instrument (*ThermoFisher Scientific*, Bremen, Germany) equipped with a heated electrospray (ESI) ionization source and connected to a *Dionex Ultimate 3000* UHPLC system (*ThermoFischer Scientifics*, Germering, Germany). The samples were dissolved in MeOH, MeOH/CH_2Cl_2 3:1, MeOH/H_2O 1:1, DMSO/H_2O 1:10 or H_2O at a concentration of ca. 50 µg mL^{-1} thereof

1 µL was injected on-flow with a XRS auto-sampler (*CTC*, Zwingen, Switzerland). The mobile phase (120 µL mL^{-1} flow rate) consisting of MeOH + 0.1% HCOOH or MeCN/H_2O 2:8 + 0.1% HCOOH was chosen according to the solubility. Ion source parameters were set as follow: spray voltage 3.0 kV; capillary temperature 280 °C; sheath gas 30 L min^{-1}; aux gas 8; 30 L min^{-1}; s-lens RF level 55.0; and aux gas temperature 250 °C. Full scan MS were acquired in the alternating (+)/(–)-ESI mode and over the ranges *m/z* = 80-1'200, 133-2'000, or 200-3'000 at 70'000 resolution (full width half-maximum) and with automatic gain control (AGC) target of 3.00E+06. The maximum allowed ion transfer time (IT) was 30 ms. Masses were calibrated below 2 ppm accuracy between *m/z* = 130.06619 and 1621.96509 in the positive and between 265.14790 and 1779.96528 in the negative ESI mode using the Pierce® ESI calibration solutions (ThermoFisher Scientific, Rockford, USA). Additionally, contaminations of erucamide (*m/z* = 338.34174, (+)-ESI) and palmitic acid (*m/z* = 255.23295, (–)-ESI) were used as lock masses in (+)-and (–)-ESI, respectively. *LC-MS*: Samples (1 µL injection) were analyzed with a Dionex Ultimate 3000UHPLC system (*ThermoFischer Scientifics*, Germering, Germany) connected to an *Acquity* eλ detector and a *Qexactive* high-resolution mass spectrometer (*ThermoFisher Scientific*, Bremen, Germany) equipped with a heated electrospray (ESI) ionization source. Separation was performed with an *AcquityBEH* C18 HPLC column (1.7 µm particle size, 2x100 mm, *Waters*) kept at 30 °C. The mobile phase was consisting of A: H_2O + 0.1% HCOOH and B: CH_3CN + 0.1% HCOOH. A linear gradient was run from 5 to 98% B within 5 min followed by flushing with 98% B for 1 min at 400 µL min^{-1} flow rate. UV spectra were recorded between 200 and 600 nm at 1.2 nm resolution and 20 points s^{-1}. MS ion source parameters were set as follows: spray voltage, 3.5 kV; capillary temperature, 260 °C; sheath gas 45 L min^{-1}; aux gas 15 L min^{-1}; sweep gas 2 L min^{-1}; s-lens RF level 450.0, and aux gas temperature 250 °C. Full scan MS were acquired in the (+)-ESI mode and over the ranges *m/z* = 80-1'200, 133-2'000, or 200-3'000 at 70'000 resolution (full width half-maximum) and with automatic gain control (AGC) target of 3.00E+06. The maximum allowed ion transfer time (IT) was 30 ms. Masses were calibrated below 2 ppm accuracy between *m/z* = 130.06619 and 1621.96509 in the positive and between 265.14790 and 1779.96528 in the negative ESI mode using the Pierce® ESI calibration solutions (*ThermoFisher Scientific*, Rockford, USA). Additionally, contaminations of erucamide (*m/z* = 338.34174, (+)-ESI) and palmitic acid (*m/z* = 255.23295, (–)-ESI) were used as lock masses in (+)-and (–)-ESI, respectively.

Experimental Procedures C3''-Functionalizations

OP-1118 (**4**)

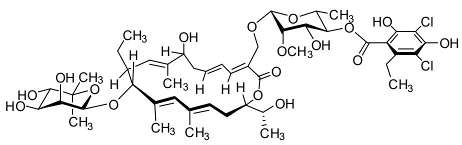

Fidaxomicin (**1**) (1.50 g, 1.42 mmol, 1.0 eq.) was dissolved in MeOH (15 mL) and K_2CO_3 (393 mg, 2.84 mmol, 2.0 eq.) was added. The reaction mixture was stirred at room temperature for 2 h. The reaction mixture was quenched with aq. sat. NH_4Cl. The aqueous phase was extracted with EtOAc (3x). The combined organic layers were washed with aq. sat. NaCl, dried over $MgSO_4$ and the solvent was evaporated under reduced pressure. The crude was purified by RP-HPLC [Gemini NX C18, 5 µ, 110 Å, 250 mm × 21.2 mm, solvent A: H_2O + 0.1% HCOOH, solvent B: MeCN + 0.1% HCOOH; 20 mL/min; LC time program (min –% B): 0.0 min – 36%, 15.0 min – 36%, 100 min – 41%, 110 min – 100%] to afford, after lyophilization, **OP-1118** (**4**) (t_R = 31.0 min, 995 mg, 1.01 mmol, 71%) as a colorless solid.

Specific Rotation $[\alpha]_D^{25\,°C}$ = −23.0 (c = 0.81, MeOH); **FT-IR** $\tilde{\nu}$ (film) 3430, 2975, 2936, 1697, 1591, 1378, 1311, 1242, 1068, 1024 cm^{-1}; **^{1}H NMR** (500 MHz, acetone-*d*$_6$) δ 7.15 (d, *J* = 11.4 Hz, 1H), 6.59 – 6.47 (m, 1H), 5.87 (ddd, *J* = 14.6, 9.6, 4.6 Hz, 1H), 5.73 (s, 1H), 5.53 (t, *J* = 7.4 Hz, 1H), 5.12 (dt, *J* = 10.6, 1.6 Hz, 1H), 5.01 (t, *J* = 9.7 Hz, 1H), 4.64 (dt, *J* = 6.7, 4.9 Hz, 1H), 4.61 (d, *J* = 1.3 Hz, 1H), 4.59 (s, 1H), 4.51 (d, *J* = 11.4 Hz, 1H), 4.33 (d, *J* = 11.5 Hz, 1H), 4.17 (m, 1H), 3.94 (quint, *J* = 6.3 Hz, 1H), 3.79 (dd, *J* = 2.9, 1.3 Hz, 1H), 3.71 (dd, *J* = 9.9, 3.4 Hz, 1H), 3.62 (d, *J* = 9.7 Hz, 1H), 3.56 – 3.47 (m, 2H), 3.42 (s, 3H), 3.41 – 3.38 (m, 2H), 2.91 (qd, *J* = 7.4, 2.3 Hz, 2H), 2.70 – 2.48 (m, 3H), 2.45 – 2.28 (m, 2H), 1.88 – 1.78 (m, 1H), 1.71 (d, *J* = 1.3 Hz, 3H), 1.64 (d, *J* = 1.4 Hz, 3H), 1.56 (s, 3H), 1.21 (d, *J* = 6.1 Hz, 3H), 1.19 – 1.15 (m, 1H), 1.14 – 1.07 (m, 9H), 0.98 (s, 3H), 0.73 (t, *J* = 7.4 Hz, 3H) ppm; **^{13}C NMR** (126 MHz, acetone-*d*$_6$) δ 169.5, 167.8, 155.9, 153.8, 145.5, 143.5, 142.7, 136.8, 136.1, 136.1, 133.7, 128.1, 126.2, 125.3, 124.1, 114.5, 110.6, 108.2, 101.8, 96.8, 92.9, 81.6, 78.2, 77.6, 75.0, 74.8, 72.9, 72.7, 72.3, 72.2, 70.6, 67.7, 63.4, 61.7, 42.1, 37.3, 29.0, 28.4, 26.5, 26.2, 20.7, 18.2, 17.6, 17.5, 15.2, 14.4, 13.8, 11.2 ppm; **HRMS** ESI(+) (MeOH), calculated for $C_{48}H_{72}Cl_2O_{17}N$ $[M+NH_4]^+$: 1004.41718, found: 1004.41728.

Bisallyl-OP-1118 (**5**)

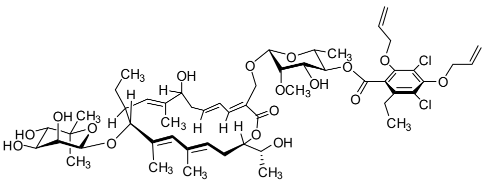

A flame-dired two-necked flask under an inert atmosphere was charged with OP-1118 (**4**) (978 mg, 0.989 mmol, 1.0 eq.) and K_2CO_3 (410 mg, 2.97 mmol, 3.0 eq.). The solids were dissolved in DMF (20 mL) and allyl bromide (215 µL, 2.47 mmol, 2.5 eq.) was added at room temperature. The reaction mixture was stirred at 50 °C until full conversion was observed (UHPLC-MS). If required, additional K_2CO_3 and allyl bromide were added to reach full conversion. The reaction mixture was diluted with EtOAc and quenched with aq. sat. NH_4Cl. The aqueous phase was extracted with EtOAc (3x). The combined organic layers were washed with water (5x), dried over $MgSO_4$ and the solvent was evaporated under reduced pressure to afford **bisallyl-OP-1118** (**5**) (1.06 g, 0.989 mmol, quant.) as a colorless solid. The crude was used without further purification in the next step.

Specific Rotation $[\alpha]_D^{25\,°C}$ = −43.64 (c = 0.71, $CHCl_3$); **FT-IR** $\tilde{\nu}$ (film) 3454, 3083, 2975, 2935, 2877, 1735, 1700, 1642, 1568, 1455, 1402, 1379, 1352, 1314, 1277, 1248, 1198, 1176, 1161, 1132, 1068, 1024 cm^{-1}; **^{1}H NMR** (400 MHz, acetone-d_6) δ 7.22 (d, J = 11.4 Hz, 1H), 6.67 – 6.57 (m, 1H), 6.23 – 6.02 (m, 2H), 5.95 (ddd, J = 14.7, 9.6, 4.6 Hz, 1H), 5.82 (s, 1H), 5.62 (t, J = 8.2 Hz, 1H), 5.49 – 5.38 (m, 2H), 5.31 – 5.23 (m, 2H), 5.20 (d, J = 10.7 Hz, 1H), 5.02 (t, J = 9.7 Hz, 1H), 4.73 (q, J = 5.4 Hz, 1H), 4.69 (s, 1H), 4.65 – 4.49 (m, 6H), 4.40 (d, J = 11.5 Hz, 1H), 4.28 – 4.23 (m, 1H), 4.08 – 3.99 (m, 2H), 3.92 – 3.84 (m, 2H), 3.75 – 3.66 (m, 4H), 3.56 – 3.47 (m, 8H), 3.41 (d, J = 3.8 Hz, 1H), 3.33 (d, J = 6.7 Hz, 1H), 2.87 – 2.79 (m, 2H), 2.76 – 2.57 (m, 3H), 2.54 – 2.37 (m, 2H), 1.97 – 1.87 (m, 1H), 1.80 (s, 3H), 1.73 (s, 3H), 1.65 (s, 3H), 1.33 (d, J = 6.1 Hz, 3H), 1.29 – 1.23 (m, 9H), 1.22 – 1.13 (m, 11H), 1.07 (s, 3H), 0.82 (t, J = 7.4 Hz, 3H) ppm; **^{13}C NMR** (126 MHz, acetone-d_6) δ 167.9, 166.5, 153.9, 152.0, 145.3, 143.3, 140.0, 136.8, 136.1, 136.1, 134.1, 134.1, 133.7, 128.7, 128.2, 126.3, 126.0, 125.4, 124.1, 122.3, 118.8, 118.7, 101.9, 96.8, 92.9, 81.9, 78.3, 77.5, 76.4, 75.0, 74.8, 72.9, 72.7, 72.4, 72.3, 70.7, 67.8, 63.4, 61.7, 42.1, 37.3, 29.0, 28.4, 26.5, 25.6, 20.7, 18.3, 17.6, 17.5, 15.2, 14.4, 13.8, 11.1 ppm; **HRMS** ESI(+) (MeOH), calculated for $C_{54}H_{76}Cl_2O_{17}Na$ $[M+Na]^+$: 1089.43518, found: 1089.43487.

7a

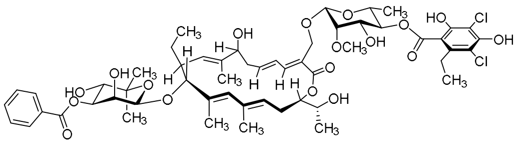

Benzoyl chloride (9.51 µL, 81.9 µmol, 3.5 eq.) was added to a stirred mixture of bisallyl-OP-1118 (**5**) (35.6 mg, 23.4 µmol, 1.0 eq.), boronic acid[1] (679 µg, 2.93 µmol, 12.5 mol%) and DIPEA (15.8 µL, 93.6 µmol, 4.0 eq.) in 1,4-dioxane (125 µL) under ambient atmosphere. The reaction mixture was stirred at 50 °C for 16 h and the reaction was allowed to reach room temperature. $Pd(PPh_3)_4$ (2.70 mg, 2.34 µmol, 10 mol%) followed by morpholine (8.15 µL, 93.6 µmol, 4.0 eq.) were added to the crude mixture under ambient atmosphere and the reaction was stirred at room temperature for 45 min. The solvent was removed under reduced pressure and the crude residue was purified by preparative RP-HPLC [Gemini NX, C18, 5 µ, 110 Å, 250 mm × 21.2 mm, solvent A: H_2O + 0.1% HCOOH, solvent B: MeCN + 0.1% HCOOH, 20 mL/min; LC time program (min –% B): 10 min – 45%, 40 min – 65%, 50 min – 100%] to afford, after lyophilization, **7a** (t_R = 32.5 min, 17.0 mg, 15.6 µmol, 67%) as a slightly yellow solid.

Specific Rotation $[\alpha]_D^{25\,°C}$ = −20.86 (c = 0.33, $CHCl_3$); **FT-IR** $\tilde{\nu}$ (film) 3441, 2975, 2932, 1699, 1587, 1451, 1376, 1314, 1279, 1246, 1178, 1114, 1066, 1024, 902, 809, 757, 720 cm^{-1}; **^{1}H NMR** (500 MHz, acetone-*d*6) δ 8.11 – 8.07 (m, 2H), 7.66 – 7.61 (m, 1H), 7.54 – 7.49 (m, 2H), 7.25 (d, *J* = 11.4 Hz, 1H), 6.64 (dd, *J* = 15.3, 12.0 Hz, 1H), 6.01 – 5.94 (m, 1H), 5.91 (s, 1H), 5.68 (t, *J* = 8.1 Hz, 1H), 5.23 (dt, *J* = 10.5, 1.6 Hz, 1H), 5.11 (t, *J* = 9.7 Hz, 1H), 5.04 (dd, *J* = 10.2, 3.1 Hz, 1H), 4.88 (d, *J* = 1.2 Hz, 1H), 4.80 – 4.72 (m, 1H), 4.69 (d, *J* = 0.9 Hz, 1H), 4.61 (d, *J* = 11.5 Hz, 1H), 4.43 (d, *J* = 11.4 Hz, 1H), 4.27 (s, 1H), 4.18 (dd, *J* = 3.2, 1.2 Hz, 1H), 4.10 – 4.02 (m, 1H), 4.00 (d, *J* = 10.3 Hz, 1H), 3.81 (dt, *J* = 9.9, 1.8 Hz, 1H), 3.67 – 3.58 (m, 2H), 3.52 (s, 3H), 3.01 (qd, *J* = 7.4, 1.5 Hz, 2H), 2.82 – 2.60 (m, 3H), 2.54 – 2.43 (m, 2H), 1.97 – 1.88 (m, 1H), 1.84 (d, *J* = 1.3 Hz, 3H), 1.76 (d, *J* = 1.4 Hz, 3H), 1.66 (dd, *J* = 1.4, 0.7 Hz, 3H), 1.33 – 1.18 (m, 16H), 0.82 (t, *J* = 7.4 Hz, 3H) ppm; **^{13}C NMR** (126 MHz, acetone-*d*6) δ 168.6, 167.0, 165.8, 155.0, 153.0, 144.6, 143.5, 142.6, 141.8, 136.0, 135.3, 135.0, 133.1, 129.7, 128.9, 128.0, 127.3, 125.5, 124.4, 123.1, 113.7, 109.7, 107.3, 100.9, 95.4, 92.2, 80.8, 77.3, 76.7, 74.9, 74.4, 72.0, 71.4, 70.6, 69.9, 69.7, 66.9, 62.5, 60.8, 41.1, 36.4, 28.1, 27.5, 25.6, 25.3, 20.7, 19.8, 17.3, 16.9, 16.7, 14.3, 13.5, 12.9 ppm; **HRMS** ESI(+), (MeOH) calculated for $C_{55}H_{72}O_{18}Cl_2Na$ $[M+Na]^+$: 1113.39934, found: 1113.39856.

[1] The boronic acid catalyst was obtained according to the procedure of Shimada and coworkers.[8]

7b

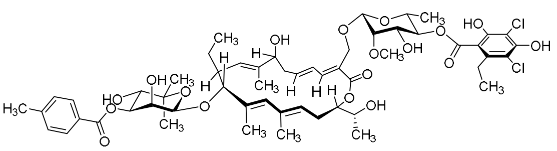

4-Methylbenzoyl chloride (10.8 µL, 81.9 µmol, 3.5 eq.) was added to a stirred mixture of **bisallyl-OP-1118** (**5**) (35.6 mg, 23.4 µmol, 1.0 eq.), boronic acid (679 µg, 2.93 µmol, 12.5 mol%) and DIPEA (15.8 µL, 93.6 µmol, 4.0 eq.) in 1,4-dioxane (125 µL) under ambient atmosphere. The reaction mixture was stirred at 40 °C for 16 h and the reaction was allowed to reach room temperature. $Pd(PPh_3)_4$ (2.70 mg, 2.34 µmol, 10 mol%) followed by morpholine (8.15 µL, 93.6 µmol, 4.0 eq.) were added to the crude mixture under ambient atmosphere and the reaction was stirred at room temperature for 45 min. The solvent was removed under reduced pressure and the crude residue was purified by preparative RP-HPLC [Gemini NX, C18, 5 µ, 110 Å, 250 mm × 21.2 mm, solvent A: H_2O + 0.1% HCOOH, solvent B: MeCN + 0.1% HCOOH, 20 mL/min; LC time program (min –% B): 12 min – 50%, 60 min – 60%, 70 min – 100%] to afford, after lyophilization, **7b** (t_R = 34.3 min, 17.5 mg, 15.8 µmol, 68%) as a slightly yellow solid.

Specific Rotation $[\alpha]_D^{25\,°C}$ = −16.0 (c = 0.25, $CHCl_3$); **FT-IR** $\tilde{\nu}$ (film) 3466, 2975, 2934, 2173, 2031, 1983, 1697, 1612, 1590, 1408, 1378, 1311, 1278, 1243, 1179, 1111, 1066, 1021, 900, 755, 664, 585, 541, 518 cm^{-1}; **^{1}H NMR** (500 MHz, acetone-d_6) δ 7.97 (d, *J* = 8.2 Hz, 2H), 7.31 (d, *J* = 8.0 Hz, 2H), 7.25 (d, *J* = 11.4 Hz, 1H), 6.69 – 6.59 (m, 1H), 5.98 (ddd, *J* = 14.6, 9.6, 4.6 Hz, 1H), 5.91 (s, 1H), 5.68 (t, *J* = 8.3 Hz, 1H), 5.23 (d, *J* = 10.5 Hz, 1H), 5.11 (t, *J* = 9.7 Hz, 1H), 5.02 (dd, *J* = 10.2, 3.2 Hz, 1H), 4.86 (s, 1H), 4.79 – 4.72 (m, 1H), 4.69 (s, 1H), 4.61 (d, *J* = 11.4 Hz, 1H), 4.43 (d, *J* = 11.4 Hz, 1H), 4.27 (s, 1H), 4.17 (s, 1H), 4.10 – 4.00 (m, 1H), 3.99 (d, *J* = 10.2 Hz, 1H), 3.86 – 3.76 (m, 2H), 3.66 – 3.52 (m, 2H), 3.52 (s, 3H), 3.05 – 2.95 (m, 2H), 2.83 – 2.60 (m, 3H), 2.55 – 2.42 (m, 2H), 2.41 (s, 3H), 1.98 – 1.88 (m, 1H), 1.83 (s, 3H), 1.76 (s, 3H), 1.66 (s, 3H), 1.33 – 1.18 (m, 16H), 0.82 (t, *J* = 7.5 Hz, 3H) ppm; **^{13}C NMR** (126 MHz, acetone-d_6) δ 169.5, 167.8, 166.7, 155.9, 153.9, 145.4, 144.4, 143.4, 142.7, 136.9, 136.1, 135.9, 133.9, 130.6, 129.8, 128.9, 128.2, 126.4, 125.3, 124.0, 114.6, 110.6, 108.2, 101.8, 96.3, 93.1, 81.6, 78.2, 77.6, 75.8, 75.3, 72.9, 72.3, 71.5, 70.8, 70.6, 67.8, 63.4, 61.7, 42.0, 37.3, 28.9, 28.4, 26.5, 26.2, 21.6, 20.7, 18.2, 17.8, 17.5, 15.2, 14.4, 13.8, 11.1 ppm; **HRMS** ESI(+), (MeOH) calculated for $C_{56}H_{74}O_{18}Cl_2Na$ $[M+Na]^+$: 1127.41499, found: 1127.41495.

7c

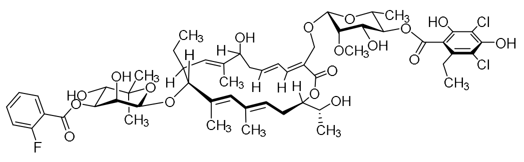

2-Fluorobenzoyl chloride (9.69 µL, 81.9 µmol, 3.5 eq.) was added to a stirred mixture of **bisallyl-OP-1118** (**5**) (35.6 mg, 23.4 µmol, 1.0 eq.), boronic acid (679 µg, 2.93 µmol, 12.5 mol%) and DIPEA (15.8 µL, 93.6 µmol, 4.0 eq.) in 1,4-dioxane (125 µL) under ambient atmosphere. The reaction mixture was stirred at 30 °C for 16 h and the reaction was allowed to reach room temperature. $Pd(PPh_3)_4$ (2.70 mg, 2.34 µmol, 10 mol%) followed by morpholine (8.15 µL, 93.6 µmol, 4.0 eq.) were added to the crude mixture under ambient atmosphere and the reaction was stirred at room temperature for 45 min. The solvent was removed under reduced pressure and the crude residue was purified by preparative RP-HPLC [Gemini NX, C18, 5 µ, 110 Å, 250 mm × 21.2 mm, solvent A: H_2O + 0.1% HCOOH, solvent B: MeCN + 0.1% HCOOH, 20 mL/min LC time program (min –% B): 12 min – 50%, 60 min – 60%, 70 min – 100%] to afford, after lyophilization, **7c** (t_R = 27.6 min, 11.3 mg, 10.2 µmol, 44%) as a slightly yellow solid.]

Specific Rotation $[\alpha]_D^{25\,°C}$ = −25.47 (c = 0.29, $CHCl_3$); **FT-IR** $\tilde{\nu}$ (film) 3457, 2976, 2934, 2108, 1701, 1613, 1588, 1489, 1456, 1377, 1307, 1244, 1160, 1125, 1066, 1022, 901, 801, 757, 662, 525 cm^{-1}; **^{1}H NMR** (500 MHz, acetone-d_6) δ 8.03 (td, *J* = 7.6, 1.8 Hz, 1H), 7.70 – 7.62 (m, 1H), 7.31 (t, *J* = 7.6 Hz, 1H), 7.30 – 7.22 (m, 2H), 6.64 (dd, *J* = 15.0, 11.5 Hz, 1H), 5.98 (ddd, *J* = 14.7, 9.6, 4.6 Hz, 1H), 5.91 (s, 1H), 5.68 (t, *J* = 8.3 Hz, 1H), 5.23 (d, *J* = 10.6 Hz, 1H), 5.11 (t, *J* = 9.7 Hz, 1H), 5.05 (dd, *J* = 10.3, 3.2 Hz, 1H), 4.88 (s, 1H), 4.79 – 4.72 (m, 1H), 4.69 (s, 1H), 4.61 (d, *J* = 11.4 Hz, 1H), 4.43 (d, *J* = 11.4 Hz, 1H), 4.27 (s, 1H), 4.19 (s, 1H), 4.09 – 4.00 (m, 1H), 3.97 (d, *J* = 10.2 Hz, 1H), 3.84 – 3.77 (m, 2H), 3.67 – 3.58 (m, 2H), 3.52 (s, 3H), 3.05 – 2.96 (m, 2H), 2.83 – 2.60 (m, 3H), 2.56 – 2.42 (m, 2H), 1.98 – 1.88 (m, 1H), 1.83 (s, 3H), 1.76 (s, 3H), 1.66 (s, 3H), 1.34 – 1.10 (m, 16H), 0.82 (t, *J* = 7.5 Hz, 3H) ppm; **^{13}C NMR** (126 MHz, acetone-d_6) δ 169.5, 167.8, 163.98 (d, *J* = 3.3 Hz), 162.72 (d, *J* = 258.9 Hz), 156.0, 154.2, 145.4, 143.5, 142.6, 136.9, 136.1, 135.9, 135.55 (d, *J* = 8.7 Hz), 134.0, 133.0, 128.2, 126.4, 125.3, 124.96 (d, *J* = 4.1 Hz), 124.0, 120.18 (d, *J* = 9.9 Hz), 117.65 (d, *J* = 22.0 Hz), 114.7, 110.3, 108.2, 101.8, 96.2, 93.1, 81.7, 78.2, 77.6, 76.3, 75.3, 72.9, 72.3, 71.4, 70.7, 70.6, 67.8, 63.4, 61.7, 42.0, 37.3, 28.9, 28.4, 26.5, 26.3, 20.7, 18.2, 17.8, 17.5, 15.2, 14.4, 13.8, 11.2 ppm; **^{19}F NMR** (376.5 MHz, acetone-d_6) δ −111.4 ppm; **HRMS** ESI(+), (MeOH) calculated for $C_{55}H_{71}O_{18}Cl_2FNa$ $[M+Na]^+$: 1131.38992, found: 1131.38906.

7d

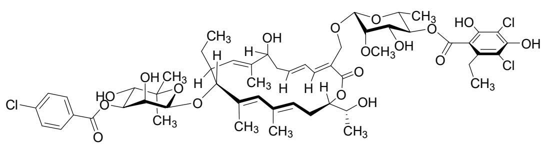

4-Chlorobenzoyl chloride (10.5 µL, 81.9 µmol, 3.5 eq.) was added to a stirred mixture of **bisallyl-OP-1118** (**5**) (35.6 mg, 23.4 µmol, 1.0 eq.), boronic acid (679 µg, 2.93 µmol, 12.5 mol%) and DIPEA (15.8 µL, 93.6 µmol, 4.0 eq.) in 1,4-dioxane (125 µL) under ambient atmosphere. The reaction mixture was stirred at 50 °C for 16 h and the reaction was allowed to reach room temperature. $Pd(PPh_3)_4$ (2.70 mg, 2.34 µmol, 10 mol%) followed by morpholine (8.15 µL, 93.6 µmol, 4.0 eq.) were added to the crude mixture under ambient atmosphere and the reaction was stirred at room temperature for 45 min. The solvent was removed under reduced pressure and the crude residue was purified by preparative RP-HPLC [Gemini NX, C18, 5 µ, 110 Å, 250 mm × 21.2 mm, solvent A: H_2O + 0.1% HCOOH, solvent B: MeCN + 0.1% HCOOH, 20 mL/min; LC time program (min –% B): 12 min – 50%, 60 min – 60%, 70 min – 100%] to afford, after lyophilization, **7d** (t_R = 39.6 min, 10.0 mg, 8.87 µmol, 38%) as a slightly yellow solid and **7d-C2** (t_R = 36.4 min, 6.80 mg, 6.03 µmol, 26%) as a slightly yellow solid.

Specific Rotation $[\alpha]_D^{25\,°C}$ = −24.38 (c = 0.28, $CHCl_3$); **FT-IR** $\tilde{\nu}$ (film) 3454, 2975, 2934, 1702, 1593, 1402, 1377, 1311, 1274, 1242, 1175, 1116, 1090, 1066, 1018, 900, 853, 759, 514 cm^{-1}; **^{1}H NMR** (500 MHz, acetone-d_6) δ 8.09 (d, *J* = 8.4 Hz, 2H), 7.56 (d, *J* = 8.4 Hz, 2H), 7.25 (d, *J* = 11.5 Hz, 1H), 6.68 – 6.59 (m, 1H), 5.97 (ddd, *J* = 14.7, 9.6, 4.6 Hz, 1H), 5.91 (s, 1H), 5.67 (t, *J* = 8.3 Hz, 1H), 5.23 (d, *J* = 10.5 Hz, 1H), 5.10 (t, *J* = 9.7 Hz, 1H), 5.04 (dd, *J* = 10.2, 3.1 Hz, 1H), 4.87 (s, 1H), 4.79 – 4.72 (m, 4H), 4.69 (s, 1H), 4.61 (d, *J* = 11.4 Hz, 1H), 4.43 (d, *J* = 11.4 Hz, 1H), 4.27 (s, 1H), 4.18 (s, 1H), 4.09 – 4.00 (m, 1H), 3.99 (d, *J* = 10.2 Hz, 1H), 3.84 – 3.78 (m, 2H), 3.67 – 3.57 (m, 2H), 3.52 (s, 3H), 3.05 – 2.96 (m, 2H), 2.83 – 2.63 (m, 3H), 2.58 – 2.42 (m, 2H), 1.98 – 1.87 (m, 1H), 1.83 (s, 3H), 1.76 (s, 3H), 1.66 (s, 3H), 1.36 – 1.18 (m, 16H), 0.81 (t, *J* = 7.4 Hz, 3H) ppm; **^{13}C NMR** (126 MHz, acetone-d_6) δ 169.5, 167.8, 165.8, 156.0, 154.2, 145.4, 143.4, 142.6, 139.6, 136.90, 136.1, 135.9, 134.0, 132.2, 130.4, 129.5, 129.5, 128.2, 126.4, 125.3, 124.0, 114.7, 108.2, 101.8, 96.2, 93.1, 81.6, 78.2, 77.6, 76.4, 75.3, 72.9, 72.3, 71.4, 70.7, 70.6, 67.7, 63.4, 61.7, 42.0, 37.3, 28.92, 28.4, 26.5, 26.3, 20.7, 18.2, 17.8, 17.5, 15.2, 14.4, 13.8, 11.1 ppm; **HRMS** ESI(+), (MeOH) calculated for $C_{55}H_{71}O_{18}Cl_3Na$ $[M+Na]^+$: 1147.36037, found: 1147.35900.

7d-C2

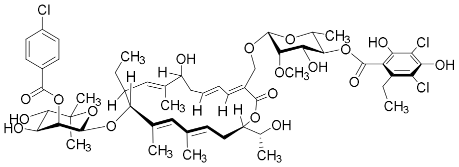

Specific Rotation $[\alpha]_D^{25\,°C}$ = −18.68 (c = 0.26, $CHCl_3$); **FT-IR** $\tilde{\nu}$ (film) 3431, 2975, 2931, 2108, 1707, 1593, 1402, 1371, 1272, 1243, 1174, 1068, 1019, 900, 850, 756, 666, 527 cm^{-1}; **^{1}H NMR** (500 MHz, acetone-d_6) δ 8.08 (d, *J* = 8.6 Hz, 2H), 7.58 (d, *J* = 8.6 Hz, 2H), 7.22 (d, *J* = 11.4 Hz, 1H), 6.61 (dd, *J* = 15.0, 11.5 Hz, 1H), 5.93 (ddd, *J* = 14.6, 9.6, 4.6 Hz, 1H), 5.86 (s, 1H), 5.65 (t, *J* = 8.3 Hz, 1H), 5.61 (dd, *J* = 3.3, 1.2 Hz, 1H), 5.18 (d, *J* = 10.5 Hz, 1H), 5.10 (t, *J* = 9.7 Hz, 1H), 4.95 (d, *J* = 1.3 Hz, 1H), 4.78 – 4.72 (m, 1H), 4.67 (s, 1H), 4.59 (d, *J* = 11.5 Hz, 1H), 4.42 (d, *J* = 11.5 Hz, 1H), 4.23 (s, 1H), 4.08 – 4.01 (m, 1H), 3.89 (dd, *J* = 10.0, 3.4 Hz, 1H), 3.80 (dd, *J* = 9.9, 3.3 Hz, 1H), 3.73 (d, *J* = 9.8 Hz, 1H), 3.66 (d, *J* = 9.9 Hz, 1H), 3.63 – 3.58 (m, 2H), 3.51 (s, 3H), 3.00 (q, *J* = 7.4 Hz, 2H), 2.80 – 2.74 (m, 1H), 2.71 – 2.63 (m, 1H), 2.52 – 2.41 (m, 3H), 1.81 – 1.76 (m, 1H), 1.75 (s, 3H), 1.73 (s, 3H), 1.56 (s, 3H), 1.32 (s, 3H), 1.30 (d, *J* = 6.2 Hz, 3H), 1.24 – 1.14 (m, 10H), 0.72 (t, *J* = 7.4 Hz, 3H) ppm; **^{13}C NMR** (126 MHz, acetone-d_6) δ 169.5, 167.8, 165.6, 156.0, 154.2, 145.4, 143.5, 142.6, 139.3, 136.8, 136.1, 135.8, 134.2, 132.2, 130.9, 129.5, 128.1, 126.5, 125.3, 124.0, 114.7, 110.3, 108.2, 101.7, 94.8, 92.2, 81.6, 78.2, 77.6, 75.42, 75.2, 74.6, 72.9, 72.3, 70.6, 70.5, 67.8, 63.4, 61.7, 41.9, 37.3, 29.2, 28.39, 26.3, 26.1, 20.7, 18.2, 17.7, 17.5, 15.1, 14.4, 13.7, 11.02 ppm; **HRMS** ESI(+), (MeOH) calculated for $C_{55}H_{71}O_{18}Cl_3Na$ $[M+Na]^+$: 1147.36037, found: 1147.36005.

7e

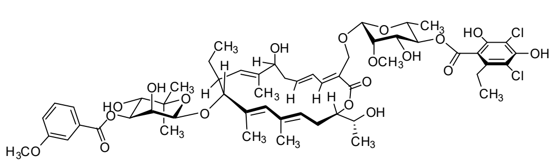

3-Anisoyl chloride (11.5 µL, 81.9 µmol, 3.5 eq.) was added to a stirred mixture of **bisallyl-OP-1118** (**5**) (35.6 mg, 23.4 µmol, 1.0 eq.), boronic acid (679 µg, 2.93 µmol, 12.5 mol%) and DIPEA (15.8 µL, 93.6 µmol, 4.0 eq.) in 1,4-dioxane (125 µL) under ambient atmosphere. The reaction mixture was stirred at 50 °C for 16 h and the reaction was allowed to reach room temperature. $Pd(PPh_3)_4$ (2.70 mg, 2.34 µmol, 10 mol%) followed by morpholine (8.15 µL, 93.6 µmol, 4.0 eq.) were added to the crude mixture under ambient atmosphere and the reaction was stirred at room temperature for 45 min. The solvent was removed under reduced pressure and the crude residue was purified by preparative RP-HPLC [Gemini NX, C18, 5 µ, 110 Å, 250 mm × 21.2 mm, solvent A: H_2O + 0.1% HCOOH, solvent B: MeCN + 0.1% HCOOH, 20 mL/min; LC time program (min –% B): 12 min – 50%, 60 min – 60%, 70 min – 100%] to afford, after lyophilization, **7e** (t_R = 30.8 min, 10.2 mg, 9.09 µmol, 39%) as a slightly yellow solid and **7e-C2** (t_R = 26.8 min, 6.8 mg, 6.06 µmol, 26%) as a slightly yellow solid.

Specific Rotation $[\alpha]_D^{25\,°C}$ = −27.89 (c = 0.38, $CHCl_3$); **FT-IR** $\tilde{\nu}$ (film) 3450, 2974, 2934, 1697, 1588, 1454, 1375, 1280, 1237, 1142, 1110, 1065, 1022, 1004, 898, 800, 755, 683, 666 cm^{-1}; **^{1}H NMR** (500 MHz, acetone-*d*$_6$) δ 7.67 (d, *J* = 7.7 Hz, 1H), 7.63 – 7.58 (m, 1H), 7.42 (t, *J* = 8.0 Hz, 1H), 7.25 (d, *J* = 11.4 Hz, 1H), 7.23 – 7.16 (m, 1H), 6.68 – 6.59 (m, 1H), 5.98 (ddd, *J* = 14.6, 9.6, 4.6 Hz, 1H), 5.91 (s, 1H), 5.68 (t, *J* = 8.1 Hz, 1H), 5.23 (d, *J* = 10.5 Hz, 1H), 5.11 (t, *J* = 9.7 Hz, 1H), 5.02 (dd, *J* = 10.2, 3.1 Hz, 1H), 4.87 (s, 1H), 4.79 – 4.72 (m, 1H), 4.69 (s, 1H), 4.61 (d, *J* = 11.4 Hz, 1H), 4.43 (d, *J* = 11.4 Hz, 1H), 4.27 (s, 1H), 4.19 (s, 1H), 4.10 – 4.00 (m, 1H), 4.00 (d, *J* = 10.2 Hz, 1H), 3.86 (s, 3H), 3.83 – 3.79 (m, 2H), 3.67 – 3.58 (m, 2H), 3.52 (s, 3H), 3.01 (qd, *J* = 7.4, 2.0 Hz, 2H), 2.84 – 2.75 (m, 1H), 2.74 – 2.61 (m, 2H), 2.56 – 2.43 (m, 2H), 1.98 – 1.89 (m, 1H), 1.84 (d, *J* = 1.3 Hz, 3H), 1.76 (s, 3H), 1.66 (s, 3H), 1.34 – 1.17 (m, 16H), 0.82 (*t*, *J* = 7.4 Hz, 3H) ppm; **^{13}C NMR** (126 MHz, acetone-*d*$_6$) δ 169.6, 167.8, 166.5, 160.6, 156.1, 154.4, 145.5, 143.5, 142.6, 136.9, 136.1, 135.9, 133.9, 133.0, 130.3, 128.2, 126.4, 125.3, 124.0, 122.7, 119.5, 115.6, 114.8, 110.0, 108.2, 101.8, 96.2, 93.1, 81.7, 78.2, 77.6, 76.2, 75.3, 72.9, 72.3, 71.4, 70.8, 70.6, 67.8, 63.4, 61.7, 55.8, 42.0, 37.3, 28.9, 28.4, 26.5, 26.3, 20.7, 18.2, 17.8, 17.6, 15.2, 14.4, 13.8, 11.1 ppm; **HRMS** ESI(+), (MeOH) calculated for $C_{56}H_{74}O_{19}Cl_2Na$ $[M+Na]^+$: 1143.40991, found: 1143.40905.

7e-C2

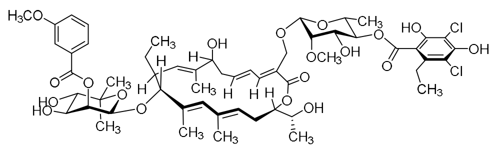

Specific Rotation $[\alpha]_D^{25\,°C}$ = −56.30 (c = 0.24, $CHCl_3$); **FT-IR** $\tilde{\nu}$ (film) 3436, 2974, 2932, 1704, 1588, 1454, 1372, 1278, 1239, 1069, 1024, 900, 756 cm^{-1}; **^{1}H NMR** (500 MHz, acetone-d_6) δ 7.67 (d, *J* = 7.8 Hz, 1H), 7.60 (d, *J* = 3.2 Hz, 1H), 7.44 (t, *J* = 8.0 Hz, 1H), 7.22 (d, *J* = 11.5 Hz, 1H), 7.19 (dd, *J* = 8.0, 3.2 Hz, 1H), 6.67 – 6.57 (m, 1H), 5.93 (ddd, *J* = 14.5, 9.5, 4.5 Hz, 1H), 5.86 (s, 1H), 5.65 (t, *J* = 8.4 Hz, 1H), 5.60 (d, *J* = 3.3 Hz, 1H), 5.19 (d, *J* = 10.5 Hz, 1H), 5.10 (t, *J* = 9.7 Hz, 1H), 4.95 (s, 1H), 4.74 (q, *J* = 5.3 Hz, 1H), 4.68 (s, 1H), 4.59 (d, *J* = 11.5 Hz, 1H), 4.42 (d, *J* = 11.5 Hz, 1H), 4.23 (s, 1H), 4.06 – 4.03 (m, 1H), 3.91 – 3.85 (m, 1H), 3.87 (s, 3H), 3.80 (dd, *J* = 9.7, 3.5 Hz, 1H), 3.76 – 3.71 (m, 1H), 3.67 (d, *J* = 9.9 Hz, 1H), 3.64 – 3.58 (m, 2H), 3.51 (s, 3H), 3.00 (q, *J* = 7.3 Hz, 2H), 2.80 – 2.74 (m, 1H), 2.72 – 2.65 (m, 1H), 2.54 – 2.40 (m, 3H), 1.86 – 1.78 (m, 1H), 1.75 (d, *J* = 1.3 Hz, 3H), 1.74 (s, 3H), 1.56 (s, 3H), 1.33 (s, 3H), 1.30 (d, *J* = 6.2 Hz, 3H), 1.24 – 1.15 (m, 10H), 0.73 (t, *J* = 7.4 Hz, 3H) ppm; **^{13}C NMR** (126 MHz, acetone-d_6) δ 169.5, 167.8, 166.3, 160.6, 155.9, 153.8, 145.5, 143.5, 142.7, 136.7, 136.1, 135.8, 134.1, 133.6, 130.3, 128.1, 126.4, 125.3, 124.1, 122.7, 119.2, 115.6, 114.5, 110.6, 108.2, 101.7, 95.0, 92.3, 81.6, 78.2, 77.6, 75.4, 75.3, 74.3, 72.9, 72.3, 70.6, 70.6, 67.8, 63.4, 61.7, 55.7, 41.9, 37.3, 29.2, 28.4, 26.2, 26.1, 20.7, 18.2, 17.7, 17.5, 15.1, 14.4, 13.7, 11.0 ppm; **HRMS** ESI(+), (MeOH) calculated for $C_{56}H_{74}O_{19}Cl_2Na$ $[M+Na]^+$: 1143.40991, found: 1143.40961.

7f

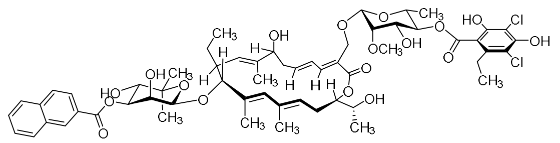

2-Naphthoyl chloride (15.6 mg, 81.9 µmol, 3.5 eq.) was added to a stirred mixture of **bisallyl-OP-1118** (**5**) (35.6 mg, 23.4 µmol, 1.0 eq.), boronic acid (679 µg, 2.93 µmol, 12.5 mol%) and DIPEA (15.8 µL, 93.6 µmol, 4.0 eq.) in 1,4-dioxane (125 µL) under ambient atmosphere. The reaction mixture was stirred at 50 °C for 16 h and the reaction was allowed to reach room temperature. $Pd(PPh_3)_4$ (2.70 mg, 2.34 µmol, 10 mol%) followed by morpholine (8.15 µL, 93.6 µmol, 4.0 eq.) were added to the crude mixture under ambient atmosphere and the reaction was stirred at room temperature for 45 min. The solvent was removed under reduced pressure and the crude residue was purified by preparative RP-HPLC [Gemini NX, C18, 5 µ, 110 Å, 250 mm × 21.2 mm, solvent A: H_2O + 0.1% HCOOH, solvent B: MeCN + 0.1% HCOOH, 20 mL/min; LC time program (min –% B): 12 min – 50%, 60 min – 70%, 70 min – 100%] to afford, after lyophilization, **7f** (t_R = 37.9 min, 10.9 mg, 9.54 µmol, 41%) as a slightly yellow solid and **7f-C2** (t_R = 34.1 min, 7.90 mg, 6.92 µmol, 30%) as a slightly yellow solid.

Specific Rotation $[\alpha]_D^{25\,°C}$ = −24.18 (c = 0.24, $CHCl_3$); **FT-IR** $\tilde{\nu}$ (film) 3446, 2975, 2933, 1696, 1590, 1374, 1285, 1231, 1197, 1131, 1064, 1021, 903, 756, 666 cm^{-1}; **^{1}H NMR** (500 MHz, acetone-*d*$_6$) δ 8.73 (s, 1H), 8.13 (dd, *J* = 8.6, 1.5 Hz, 1H), 8.11 (d, *J* = 8.1 Hz, 1H), 8.02 (dd, *J* = 8.5, 2.8 Hz, 2H), 7.72-7.60 (m, 2H), 7.28 (d, *J* = 11.4 Hz, 1H), 6.70 – 6.61 (m, 1H), 6.00 (ddd, *J* = 14.6, 9.6, 4.6 Hz, 1H), 5.94 (s, 1H), 5.71 (t, *J* = 8.1 Hz, 1H), 5.26 (d, *J* = 10.5 Hz, 1H), 5.13 (ddd, *J* = 9.8, 6.2, 3.1 Hz, 1H), 4.92 (s, 1H), 4.82-4.74 (m, 1H), 4.71 (s, 1H), 4.63 (d, *J* = 11.4 Hz, 1H), 4.45 (d, *J* = 11.4 Hz, 1H), 4.29 (s, 1H), 4.28 – 4.24 (m, 1H), 4.11 – 4.04 (m, 2H), 3.87 – 3.80 (m, 2H), 3.68 – 3.60 (m, 2H), 3.54 (s, 3H), 3.02 (q, *J* = 7.0 Hz, 3H), 2.85 – 2.78 (m, 1H), 2.76 – 2.62 (m, 2H), 2.56 – 2.40 (m, 2H), 2.00 – 1.91 (m, 1H), 1.87 (s, 3H), 1.78 (s, 3H), 1.68 (s, 3H), 1.35 – 1.19 (m, 16H), 0.84 (t, *J* = 7.4 Hz, 3H) ppm; **^{13}C NMR** (126 MHz, acetone-*d*$_6$) δ 169.5, 167.8, 166.8, 156.0, 154.2, 145.4, 143.4, 142.6, 136.9, 136.5, 136.2, 135.9, 134.0, 133.4, 131.8, 130.1, 129.2, 128.9, 128.9, 128.7, 128.2, 127.7, 126.4, 126.2, 125.3, 124.0, 114.7, 110.2, 108.2, 101.7, 96.3, 93.1, 81.7, 78.2, 77.6, 76.2, 75.3, 72.9, 72.3, 71.5, 70.9, 70.6, 67.7, 63.4, 61.7, 42.0, 37.3, 29.0, 28.4, 26.5, 26.3, 20.7, 18.2, 17.9, 17.6, 15.2, 14.4, 13.8, 11.2 ppm; **HRMS** ESI(+), (MeOH) calculated for $C_{59}H_{74}O_{18}Cl_2Na$ $[M+Na]^+$: 1163.41499, found: 1163.41462.

7f-C2

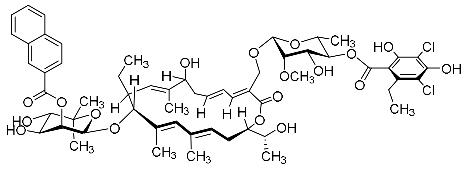

Specific Rotation $[\alpha]_D^{25\,°C}$ = −58.61 (c = 0.24, $CHCl_3$); **FT-IR** $\tilde{\nu}$ (film) 3435, 2975, 2932, 1704, 1590, 1371, 1284, 1232, 1197, 1069, 1023, 902, 778, 757, 666 cm^{-1}; **^{1}H NMR** (500 MHz, acetone-d_6) δ 8.69 (d, *J* = 1.5 Hz, 1H), 8.13 (dd, *J* = 8.6, 1.7 Hz, 1H), 8.10 (d, *J* = 8.1 Hz, 1H), 8.06 – 7.97 (m, 2H), 7.68 – 7.60 (m, 2H), 7.22 (d, *J* = 11.4 Hz, 1H), 6.67 – 6.50 (m, 1H), 5.92 (ddd, *J* = 14.7, 9.7, 4.7 Hz, 1H), 5.87 (s, 1H), 5.72 – 5.62 (m, 2H), 5.19 (d, *J* = 10.4 Hz, 1H), 5.10 (t, *J* = 9.7 Hz, 1H), 4.99 (d, *J* = 1.3 Hz, 1H), 4.75 (q, *J* = 5.4 Hz, 1H), 4.67 (s, 1H), 4.59 (d, *J* = 11.5 Hz, 1H), 4.41 (d, *J* = 11.5 Hz, 1H), 4.21 (s, 1H), 4.08 – 4.00 (m, 1H), 3.92 (dd, *J* = 10.0, 3.4 Hz, 1H), 3.83 – 3.74 (m, 3H), 3.65 – 3.57 (m, 2H), 3.51 (s, 3H), 3.00 (q, *J* = 7.4 Hz, 2H), 2.83 – 2.75 (m, 1H), 2.69 – 2.63 (m, 1H), 2.51 – 2.42 (m, 3H), 1.84 – 1.79 (m, 1H), 1.75 (d, *J* = 1.3 Hz, 3H), 1.73 (d, *J* = 1.4 Hz, 3H), 1.53 (d, *J* = 1.2 Hz, 3H), 1.38 (s, 3H), 1.30 (d, *J* = 6.1 Hz, 3H), 1.25 – 1.16 (m, 10H), 0.72 (t, *J* = 7.4 Hz, 3H) ppm; **^{13}C NMR** (126 MHz, acetone-d_6) δ 169.4, 167.8, 166.6, 155.9, 153.9, 145.5, 143.5, 142.7, 136.7, 136.4, 136.1, 135.8, 134.2, 133.5, 131.7, 130.1, 129.6, 129.1, 128.9, 128.7, 128.1, 127.6, 126.4, 126.4, 125.2, 124.1, 114.6, 110.5, 108.1, 101.7, 95.0, 92.2, 81.6, 78.2, 77.6, 75.4, 75.2, 74.4, 72.8, 72.3, 70.6, 70.5, 67.7, 63.4, 61.7, 41.9, 37.2, 28.4, 28.1, 26.3, 26.1, 20.6, 18.2, 17.7, 17.5, 15.1, 14.4, 13.7, 11.0 ppm; **HRMS** ESI(+), (MeOH) calculated for $C_{59}H_{74}O_{18}Cl_2Na$ $[M+Na]^+$: 1163.41499, found: 1163.41473.

7j

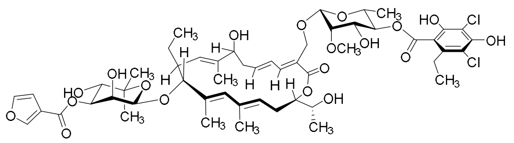

3-Furoyl chloride (15.5 mg, 81.9 µmol, 3.5 eq.) was added to a stirred mixture **bisallyl-OP-1118** (**5**) (35.6 mg, 23.4 µmol, 1.0 eq.), boronic acid (679 µg, 2.93 µmol, 12.5 mol%) and DIPEA (15.8 µL, 93.6 µmol, 4.0 eq.) in 1,4-dioxane (125 µL) under ambient atmosphere. The reaction mixture was stirred at 40 °C for 2 h (*in this case, prolonged reaction time led to decomposition*) and the reaction was allowed to reach room temperature. $Pd(PPh_3)_4$ (2.70 mg, 2.34 µmol, 10 mol%) followed by morpholine (8.15 µL, 93.6 µmol, 4.0 eq.) were added to the crude mixture under ambient atmosphere and the reaction was stirred at room temperature for 45 min. The solvent was removed under reduced pressure and the crude residue was purified by preparative RP-HPLC [Gemini NX, C18, 5 µ, 110 Å, 250 mm × 21.2 mm, solvent A: H_2O + 0.1% HCOOH, solvent B: MeCN + 0.1% HCOOH, 20 mL/min; LC time program (min –% B): 12 min – 50%, 60 min – 60%, 70 min – 100%] to afford, after lyophilization, **7j** (t_R = 20 min, 22.5 mg, 20.8 µmol, 89%) as a slightly yellow solid.

Specific Rotation $[\alpha]_D^{25\,°C}$ = −23.68 (c = 0.27, $CHCl_3$); **FT-IR** $\tilde{\nu}$ (film) 3468, 2975, 2935, 2317, 2223, 2207, 2174, 2105, 1981, 1963, 1703 1582, 1507, 1378, 1312, 1243, 1161, 1141, 1066, 1021, 899, 874, 800, 766, 601, 541, 525 cm^{-1}; **^{1}H NMR** (500 MHz, acetone-*d*$_6$) δ 8.24 – 8.20 (m, 1H), 7.66 (t, *J* = 1.8 Hz, 1H), 7.25 (d, *J* = 11.4 Hz, 1H), 6.80 (d, *J* = 1.8 Hz, 1H), 6.69 – 6.57 (m, 1H), 5.97 (ddd, *J* = 14.6, 9.6, 4.6 Hz, 1H), 5.90 (s, 1H), 5.67 (t, *J* = 8.1 Hz, 1H), 5.23 (d, *J* = 10.5 Hz, 1H), 5.10 (t, *J* = 9.7 Hz, 1H), 4.94 (dd, *J* = 10.3, 3.1 Hz, 1H), 4.84 (s, 1H), 4.77 – 4.72 (m, 1H), 4.68 (s, 1H), 4.61 (d, *J* = 11.5 Hz, 1H), 4.42 (d, *J* = 11.4 Hz, 1H), 4.27 (s, 1H), 4.15 (d, *J* = 3.1 Hz, 1H), 4.08 – 3.98 (m, 1H), 3.91 (d, *J* = 10.2 Hz, 1H), 3.83 – 3.73 (m, 2H), 3.67 – 3.56 (m, 2H), 3.52 (s, 3H), 3.00 (qd, *J* = 7.4, 1.5 Hz, 2H), 2.82 – 2.74 (m, 1H), 2.73 – 2.60 (m, 2H), 2.52 – 2.43 (m, 2H), 1.98 – 1.86 (m, 1H), 1.83 (d, *J* = 1.3 Hz, 3H), 1.75 (d, *J* = 1.3 Hz, 3H), 1.65 (s, 3H), 1.43 – 1.14 (m, 16H), 0.82 (t, *J* = 7.4 Hz, 3H) ppm; **^{13}C NMR** (126 MHz, acetone-*d*$_6$) δ 169.5, 167.8, 163.3, 155.9, 154.0, 149.1, 145.4, 145.1, 143.4, 142.6, 136.9, 136.1, 135.9, 133.9, 128.2, 126.4, 125.3, 124.0, 120.6, 114.6, 110.7, 110.5, 108.2, 101.8, 96.2, 93.0, 81.6, 78.2, 77.6, 75.6, 75.2, 72.9, 72.3, 71.4, 70.8, 70.6, 67.8, 63.4, 61.7, 42.0, 37.3, 28.9, 28.4, 26.5, 26.2, 20.7, 18.2, 17.8, 17.5, 15.2, 14.4, 13.8, 11.1 ppm; **HRMS** ESI(+), (MeOH) calculated for $C_{53}H_{70}O_{19}Cl_2Na$ $[M+Na]^+$: 1103.37861, found: 1103.37844.

7k

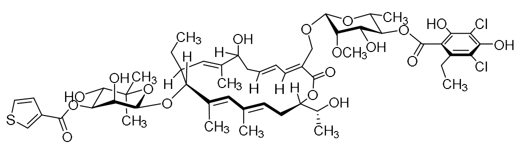

3-Thiophenecarbonyl chloride (12.0 mg, 81.9 µmol, 3.5 eq.) was added to a stirred mixture of **bisallyl-OP-1118** (**5**) (35.6 mg, 23.4 µmol, 1.0 eq.), boronic acid (679 µg, 2.93 µmol, 12.5 mol%) and DIPEA (15.8 µL, 93.6 µmol, 4.0 eq.) in 1,4-dioxane (125 µL) under ambient atmosphere. The reaction mixture was stirred at 40 °C for 16 h and the reaction was allowed to reach room temperature. $Pd(PPh_3)_4$ (2.70 mg, 2.34 µmol, 10 mol%) followed by morpholine (8.15 µL, 93.6 µmol, 4.0 eq.) were added to the crude mixture under ambient atmosphere and the reaction was stirred at room temperature for 45 min. The solvent was removed under reduced pressure and the crude residue was purified by preparative RP-HPLC [Gemini NX, C18, 5 µ, 110 Å, 250 mm × 21.2 mm, solvent A: H_2O + 0.1% HCOOH, solvent B: MeCN + 0.1% HCOOH, 20 mL/min; LC time program (min –% B): 12 min – 50%, 60 min – 60%, 70 min – 100%] to afford, after lyophilization, **7k** (t_R = 23.8 min, 22.7 mg, 19.3 µmol, 82%) as a slightly yellow solid.

Specific Rotation $[\alpha]_D^{25\,°C}$ = −36.14 (c = 0.28, $CHCl_3$); **FT-IR** $\tilde{\nu}$ (film 3440, 2973, 2931, 1695, 1589, 1522, 1408, 1379, 1311, 1245, 1197, 1162, 1142, 1109, 1064, 1021, 1003, 986, 899, 873, 800, 752, 666, 626 cm^{-1}; **^{1}H NMR** (500 MHz, acetone-d_6) δ 8.32 – 8.27 (m, 1H), 7.57 – 7.52 (m, 2H), 7.25 (d, J = 11.5 Hz, 1H), 6.69 – 6.59 (m, 1H), 5.98 (ddd, J = 14.7, 9.6, 4.6 Hz, 1H), 5.91 (s, 1H), 5.72 – 5.64 (m, 1H), 5.23 (d, J = 10.5 Hz, 1H), 5.11 (t, J = 9.7 Hz, 1H), 4.97 (dd, J = 10.3, 3.1 Hz, 1H), 4.86 (s, 1H), 4.78 – 4.72 (m, 1H), 4.69 (s, 1H), 4.61 (d, J = 11.4 Hz, 1H), 4.43 (d, J = 11.5 Hz, 1H), 4.28 – 4.25 (m, 1H), 4.18 – 4.16 (m, 1H), 4.08 – 4.02 (m, 1H), 3.95 (d, J = 10.2 Hz, 1H), 3.84 – 3.78 (m, 2H), 3.65 – 3.58 (m, 2H), 3.52 (s, 3H), 3.01 (qd, J = 7.4, 1.8 Hz, 2H), 2.80 – 2.61 (m, 3H), 2.55 – 2.42 (m, 2H), 1.98 – 1.89 (m, 1H), 1.83 (s, 3H), 1.75 (s, 3H), 1.66 (s, 3H), 1.37 – 1.14 (m, 16H), 0.82 (t, J = 7.4 Hz, 3H) ppm; **^{13}C NMR** (126 MHz, acetone-d_6) δ 169.5, 167.8, 162.9, 156.0, 154.1, 145.4, 143.4, 142.7, 136.9, 136.14, 135.9, 135.0, 133.9, 133.9, 128.8, 128.2, 127.2, 126.4, 125.3, 124.0, 114.6, 110.4, 108.2, 101.8, 96.2, 93.0, 81.6, 78.2, 77.6, 75.8, 75.3, 72.9, 72.3, 71.4, 70.8, 70.6, 67.7, 63.4, 61.7, 42.0, 37.3, 28.9, 28.4, 26.5, 26.3, 20.7, 18.2, 17.8, 17.5, 15.2, 14.4, 13.8, 11.1 ppm; **HRMS** ESI(+), (MeOH) calculated for $C_{53}H_{70}O_{18}Cl_2NaS$ $[M+Na]^+$: 1119.35576, found: 1119.35543.

7l

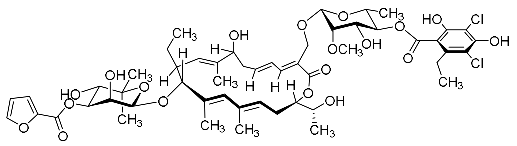

2-Furoyl chloride (8.10 µL, 81.9 µmol, 3.5 eq.) was added to a stirred mixture of **bisallyl-OP-1118** (**5**) (35.6 mg, 23.4 µmol, 1.0 eq.), boronic acid (679 µg, 2.93 µmol, 12.5 mol%) and DIPEA (15.8 µL, 93.6 µmol, 4.0 eq.) in 1,4-dioxane (125 µL) under ambient atmosphere. The reaction mixture was stirred at 50 °C for 16 h and the reaction was allowed to reach room temperature. $Pd(PPh_3)_4$ (2.70 mg, 2.34 µmol, 10 mol%) followed by morpholine (8.15 µL, 93.6 µmol, 4.0 eq.) were added to the crude mixture under ambient atmosphere and the reaction was stirred at room temperature for 45 min. The solvent was removed under reduced pressure and the crude residue was purified by preparative RP-HPLC [Gemini NX, C18, 5 µ, 110 Å, 250 mm × 21.2 mm, solvent A: H_2O + 0.1% HCOOH, solvent B: MeCN + 0.1% HCOOH, 20 mL/min; LC time program (min –% B): 0.0 min to 60 min – 50%, 70 min – 100%] to afford, after lyophilization, **7l** (t_R = 20 min, 17.3 mg, 16.0 µmol, 68%) as a slightly yellow solid.

Specific Rotation $[\alpha]_D^{25\,°C}$ = −2.64 (c = 0.34, $CHCl_3$); **FT-IR** $\tilde{\nu}$ (film) 3458, 2932, 1702, 1379, 1305, 1242,1119, 1066, 1021, 761 cm^{-1}; **^{1}H NMR** (400 MHz, acetone-d_6) δ 7.83 – 7.78 (m, 1H), 7.30 – 7.21 (m, 2H), 6.69 – 6.58 (m, 2H), 5.97 (ddd, *J* = 14.6, 9.6, 4.6 Hz, 1H), 5.90 (s, 1H), 5.67 (t, *J* = 7.9 Hz, 1H), 5.23 (d, *J* = 10.5 Hz, 1H), 5.10 (t, *J* = 9.7 Hz, 1H), 4.99 (dd, *J* = 10.2, 3.0 Hz, 1H), 4.86 (s, 1H), 4.75 (q, *J* = 5.0 Hz, 1H), 4.69 (s, 1H), 4.61 (d, *J* = 11.5 Hz, 1H), 4.43 (d, *J* = 11.4 Hz, 1H), 4.27 (s, 1H), 4.15 (s, 1H), 4.09 – 4.00 (m, 1H), 3.93 (d, *J* = 10.1 Hz, 1H), 3.81 (m, 2H), 3.67 – 3.57 (m, 2H), 3.52 (s, 3H), 3.01 (q, *J* = 7.3 Hz, 2H), 2.81 – 2.60 (m, 3H), 2.54 – 2.42 (m, 2H), 1.97 – 1.87 (m, 1H), 1.84 – 1.80 (m, 3H), 1.75 (s, 3H), 1.66 (s, 3H), 1.35 – 1.14 (m, 16H), 0.82 (t, *J* = 7.4 Hz, 3H) ppm; **^{13}C NMR** (126 MHz, acetone-d_6) δ 169.5, 167.8, 158.8, 155.9, 153.9, 147.7, 145.9, 145.4, 143.4, 142.7, 136.9, 136.1, 135.9, 134.0, 128.2, 126.4, 125.3, 124.0, 118.8, 114.6, 112.7, 110.5, 108.2, 101.8, 96.2, 93.1, 81.6, 78.2, 77.6, 75.9, 75.3, 72.9, 72.3, 71.4, 70.8, 70.6, 67.8, 63.4, 61.7, 42.0, 37.3, 28.9, 28.4, 26.5, 26.2, 20.7, 18.2, 17.8, 17.5, 15.2, 14.4, 13.8, 11.1 ppm; **HRMS** ESI(+), (MeOH) calculated for $C_{53}H_{70}O_{19}Cl_2Na$ $[M+Na]^+$: 1103.37861, found: 1103.37839.

7m

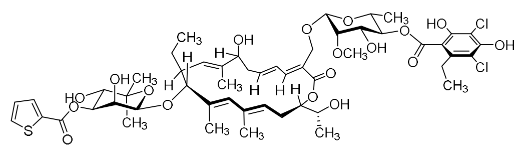

2-Thiophenecarbonyl chloride (8.76 µL, 81.9 µmol, 3.5 eq.) was added to a stirred mixture of **bisallyl-OP-1118** (**5**) (35.6 mg, 23.4 µmol, 1.0 eq.), boronic acid (679 µg, 2.93 µmol, 12.5 mol%) and DIPEA (15.8 µL, 93.6 µmol, 4.0 eq.) in 1,4-dioxane (125 µL) under ambient atmosphere. The reaction mixture was stirred at 40 °C for 16 h and the reaction was allowed to reach room temperature. $Pd(PPh_3)_4$ (2.70 mg, 2.34 µmol, 10 mol%) followed by morpholine (8.15 µL, 93.6 µmol, 4.0 eq.) were added to the crude mixture under ambient atmosphere and the reaction was stirred at room temperature for 45 min. The solvent was removed under reduced pressure and the crude residue was purified by preparative RP-HPLC [Gemini NX, C18, 5 µ, 110 Å, 250 mm × 21.2 mm, solvent A: H_2O + 0.1% HCOOH, solvent B: MeCN + 0.1% HCOOH, 20 mL/min LC time program (min –% B): 12 min – 50%, 60 min – 70%, 70 min – 100%] to afford, after lyophilization, **7m** (t_R = 24.4 min, 15.0 mg, 12.7 µmol, 54%) as a slightly yellow solid.

Specific Rotation $[\alpha]_D^{25\,°C}$ = +10.87 (c = 0.25, $CHCl_3$); **FT-IR** $\tilde{\nu}$ (film) 3460, 2975, 2934, 2034, 1981, 1694, 1589, 1524, 1416, 1372, 1246, 1142, 1093, 1067, 1022, 900, 755, 601, 524 cm^{-1}; **^{1}H NMR** (500 MHz, acetone-d_6) δ 7.87 – 7.80 (m, 2H), 7.25 (d, *J* = 11.5 Hz, 1H), 7.020 (dd, *J* = 5.0, 3.7 Hz, 1H), 6.69 – 6.57 (m, 1H), 5.97 (ddd, *J* = 14.7, 9.6, 4.6 Hz, 1H), 5.90 (s, 1H), 5.67 (t, *J* = 8.3 Hz, 1H), 5.23 (d, *J* = 10.5, 1H), 5.11 (t, *J* = 9.7 Hz, 1H), 4.99 (dd, *J* = 10.2, 3.1 Hz, 1H), 4.86 (d, *J* = 1.1 Hz, 1H), 4.75 (m, 1H), 4.69 (s, 1H), 4.61 (d, *J* = 11.5 Hz, 1H), 4.43 (d, *J* = 11.4 Hz, 1H), 4.27 (m, 1H), 4.16 (m, 1H), 4.04 (m, 1H), 3.95 (m, 1H), 3.81 (m, 2H), 3.61 (m, 2H), 3.52 (s, 3H), 3.13 – 2.92 (m, 2H), 2.74 – 2.60 (m, 3H), 2.48 (m, 2H), 1.98 – 1.88 (m, 1H), 1.83 (d, *J* = 1.3 Hz, 3H), 1.75 (d, *J* = 1.3 Hz, 3H), 1.68 – 1.63 (m, 3H), 1.37 – 1.16 (m, 16H), 0.82 (t, *J* = 7.4 Hz, 3H) ppm; **^{13}C NMR** (126 MHz, acetone-d_6) δ 169.5, 167.8, 162.5, 156.0, 154.1, 145.4, 143.5, 142.6, 136.9, 136.1, 135.9, 135.1, 134.4, 134.0, 133.8, 128.7, 128.2, 126.4, 125.3, 124.0, 114.7, 110.3, 108.2, 101.8, 96.2, 93.1, 81.7, 78.2, 77.6, 76.23, 75.3, 72.9, 72.3, 71.4, 70.8, 70.7, 70.6, 67.8, 63.4, 61.7, 42.0, 37.3, 28.9, 28.4, 26.5, 26.3, 20.7, 18.2, 17.8, 17.5, 15.2, 14.4, 13.8, 11.1 ppm; **HRMS** ESI(+), (MeOH) calculated for $C_{53}H_{70}O_{18}Cl_2NaS$ $[M+Na]^+$: 1119.35576, found: 1119.35551.

7n

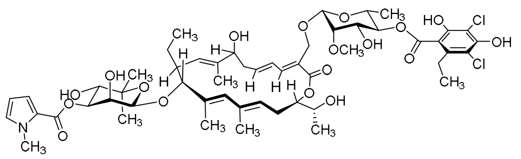

1-Methylpyrrole-2-carbonyl chloride (11.8 mg, 81.9 µmol, 3.5 eq.) was added to a stirred mixture of **bisallyl-OP-1118** (**5**) (35.6 mg, 23.4 µmol, 1.0 eq.), boronic acid (679 µg, 2.93 µmol, 12.5 mol%) and DIPEA (15.8 µL, 93.6 µmol, 4.0 eq.) in 1,4-dioxane (125 µL) under ambient atmosphere. The reaction mixture was stirred at 30 °C for 16 h and the reaction was allowed to reach room temperature. $Pd(PPh_3)_4$ (2.70 mg, 2.34 µmol, 10 mol%) followed by morpholine (8.15 µL, 93.6 µmol, 4.0 eq.) were added to the crude mixture under ambient atmosphere and the reaction was stirred at room temperature for 45 min. The solvent was removed under reduced pressure and the crude residue was purified by preparative RP-HPLC [Gemini NX, C18, 5 µ, 110 Å, 250 mm × 21.2 mm, solvent A: H_2O + 0.1% HCOOH, solvent B: MeCN + 0.1% HCOOH, 20 mL/min; LC time program (min –% B): 12 min – 50%, 60 min – 60%, 70 min – 100%] to afford, after lyophilization, **7n** (t_R = 24.2 min, 17.5 mg, 14.9 µmol, 64%) as a slightly yellow solid.

Specific Rotation $[\alpha]_D^{25\,°C}$ = −25.00 (c = 0.26, $CHCl_3$); **FT-IR** $\tilde{\nu}$ (film) 3464, 2975, 2935, 2067, 2013, 1983, 1964, 1689, 1589, 1532, 1411, 1383, 1323, 1245, 1141, 1112, 1065, 1020, 898, 801, 755, 665, 601, 584, 540, 510 cm^{-1}; **^{1}H NMR** (500 MHz, acetone-*d*6) δ 7.25 (d, *J* = 11.5 Hz, 1H), 7.00 – 6.92 (m, 2H), 6.63 (dd, *J* = 15.0, 11.5 Hz, 1H), 6.07 (dd, *J* = 4.0, 2.5 Hz, 1H), 5.97 (ddd, *J* = 14.6, 9.6, 4.6 Hz, 1H), 5.90 (s, 1H), 5.67 (t, *J* = 8.1 Hz, 1H), 5.23 (d, *J* = 10.6 Hz, 1H), 5.10 (t, *J* = 9.7 Hz, 1H), 4.96 (dd, *J* = 10.3, 3.1 Hz, 1H), 4.84 (s, 1H), 4.79 – 4.72 (m, 1H), 4.69 (s, 1H), 4.61 (d, *J* = 11.4 Hz, 1H), 4.43 (d, *J* = 11.5 Hz, 1H), 4.27 (s, 1H), 4.12 (s, 1H), 4.08 – 4.01 (m, 1H), 3.95 – 3.89 (m, 4H), 3.84 – 3.77 (m, 2H), 3.66 – 3.58 (m, 2H), 3.52 (s, 3H), 3.00 (q, *J* = 7.3 Hz, 2H), 2.75 – 2.60 (m, 3H), 2.57 – 2.41 (m, 2H), 1.95 – 1.90 (m, 1H), 1.83 (s, 3H), 1.75 (s, 3H), 1.66 (s, 3H), 1.38 – 1.15 (m, 16H), 0.82 (t, *J* = 7.4 Hz, 3H) ppm; **^{13}C NMR** (126 MHz, acetone-*d*6) δ 169.5, 167.8, 161.5, 155.9, 153.8, 145.4, 143.4, 142.7, 136.9, 136.1, 136.0, 133.9, 130.5, 128.2, 126.4, 125.3, 124.0, 123.6, 118.9, 114.5, 110.6, 108.3, 108.2, 101.8, 96.3, 93.0, 81.7, 78.2, 77.6, 75.3, 74.7, 72.9, 72.3, 71.5, 71.0, 70.6, 67.8, 63.4, 61.7, 42.0, 37.3, 37.1, 28.9, 28.4, 26.5, 26.2, 20.7, 18.23, 17.8, 17.5, 15.2, 14.4, 13.8, 11.2 ppm; **HRMS** ESI(+), (MeOH) calculated for $C_{54}H_{73}O_{18}Cl_2NNa$ $[M+Na]^+$: 1116.41024, found: 1116.40971.

7p

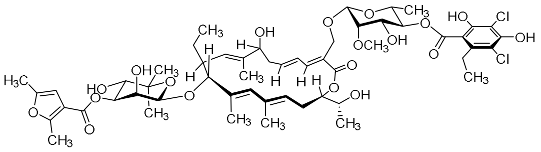

2,5-Dimethylfuran-3-carbonyl chloride (10.9 µL, 81.9 µmol, 3.5 eq.) was added to a stirred mixture of **bisallyl-OP-1118** (**5**) (35.6 mg, 23.4 µmol, 1.0 eq.), boronic acid (679 µg, 2.93 µmol, 12.5 mol%) and DIPEA (15.8 µL, 93.6 µmol, 4.0 eq.) in 1,4-dioxane (125 µL) under ambient atmosphere. The reaction mixture was stirred at 50 °C for 2 h and the reaction was allowed to reach room temperature. $Pd(PPh_3)_4$ (2.70 mg, 2.34 µmol, 10 mol%) followed by morpholine (8.15 µL, 93.6 µmol, 4.0 eq.) were added to the crude mixture under ambient atmosphere and the reaction was stirred at room temperature for 45 min. The solvent was removed under reduced pressure and the crude residue was purified by preparative RP-HPLC [Gemini NX, C18, 5 µ, 110 Å, 250 mm × 21.2 mm, solvent A: H_2O + 0.1% HCOOH, solvent B: MeCN + 0.1% HCOOH, 20 mL/min LC time program (min –% B): 12 min – 50%, 60 min – 60%, 70 min – 100%] to afford, after lyophilization, **7p** (t_R = 30.4 min, 19.4 mg, 17.5 µmol, 75%) as a slightly yellow solid.

Specific Rotation $[\alpha]_D^{25\,°C}$ = −42.65 (c = 0.49, $CHCl_3$); **FT-IR** $\tilde{\nu}$ (film) 3463, 2975, 1701, 1589, 1367, 1235, 1068, 1022 cm^{-1}; **^{1}H NMR** (500 MHz, acetone-d_6) δ 7.25 (d, *J* = 11.4 Hz, 1H), 6.63 (dd, *J* = 15.0, 11.5 Hz, 1H), 6.28 (s, 1H), 5.97 (ddd, *J* = 14.6, 9.6, 4.6 Hz, 1H), 5.90 (s, 1H), 5.67 (t, *J* = 8.3 Hz, 1H), 5.23 (d, *J* = 10.4 Hz, 1H), 5.10 (t, *J* = 9.7 Hz, 1H), 4.95 (dd, *J* = 10.3, 3.1 Hz, 1H), 4.83 (s, 1H), 4.79 – 4.71 (m, 1H), 4.69 (s, 1H), 4.61 (d, *J* = 11.4 Hz, 1H), 4.43 (d, *J* = 11.4 Hz, 1H), 4.27 (s, 1H), 4.10 (d, *J* = 3.0 Hz, 1H), 4.09 – 4.00 (m, 1H), 3.90 (d, *J* = 10.3 Hz, 1H), 3.85 – 3.76 (m, 2H), 3.66 – 3.57 (m, 2H), 3.52 (s, 3H), 3.06 – 2.95 (m, 2H), 2.81 – 2.74 (m, 1H), 2.75 – 2.60 (m, 2H), 2.52 (s, 3H), 2.52 – 2.41 (m, 2H), 2.23 (s, 3H), 1.98 – 1.86 (m, 1H), 1.83 (s, 3H), 1.75 (s, 3H), 1.66 (s, 3H), 1.34 – 1.16 (m, 16H), 0.82 (t, *J* = 7.4 Hz, 3H) ppm; **^{13}C NMR** (126 MHz, acetone-d_6) δ 169.5, 167.8, 164.2, 158.3, 156.0, 154.1, 150.8, 145.4, 143.4, 142.6, 136.9, 136.1, 135.9, 133.9, 128.2, 126.4, 125.3, 124.0, 115.2, 114.6, 110.4, 108.2, 107.3, 101.8, 96.2, 93.0, 81.6, 78.2, 77.6, 75.3, 74.9, 72.9, 72.2, 71.5, 70.9, 70.6, 67.8, 63.4, 61.7, 42.0, 37.3, 28.9, 28.4, 26.5, 26.2, 20.7, 18.2, 17.80, 17.5, 15.2, 14.4, 13.8, 13.8, 13.7, 11.1 ppm; **HRMS** ESI(+), (MeOH) calculated for $C_{55}H_{74}Cl_2O_{19}Na$ $[M+Na]^+$: 1131.40936, found: 1131.40922.

7q

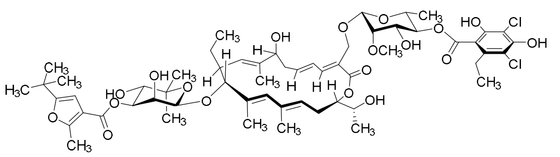

5-*tert*-Butyl-2-methylfuran-3-carbonylchloride (16.4 mg, 81.9 µmol, 3.5 eq.) was added to a stirred mixture of **bisallyl-OP-1118** (**5**) (35.6 mg, 23.4 µmol, 1.0 eq.), boronic acid (679 µg, 2.93 µmol, 12.5 mol%) and DIPEA (15.8 µL, 93.6 µmol, 4.0 eq.) in 1,4-dioxane (125 µL) under ambient atmosphere. The reaction mixture was stirred at 50 °C for 2 h and the reaction was allowed to reach room temperature. $Pd(PPh_3)_4$ (2.70 mg, 2.34 µmol, 10 mol%) followed by morpholine (8.15 µL, 93.6 µmol, 4.0 eq.) were added to the crude mixture under ambient atmosphere and the reaction was stirred at room temperature for 45 min. The solvent was removed under reduced pressure and the crude residue was purified by preparative RP-HPLC [Gemini NX, C18, 5 µ, 110 Å, 250 mm × 21.2 mm, solvent A: H_2O + 0.1% HCOOH, solvent B: MeCN + 0.1% HCOOH, 20 mL/min; LC time program (min –% B): 12 min – 50%, 60 min – 60%, 70 min – 100%] to afford, after lyophilization, **7q** (t_R = 37.7 min, 18.5 mg, 16.1 µmol, 69%) as a slightly yellow solid.

Specific Rotation $[\alpha]_D^{25\,°C}$ = −31.87 (c = 0.46, $CHCl_3$); **FT-IR** $\tilde{\nu}$ (film) 3428, 2971, 2934, 2874, 1704, 1611, 1584, 1402, 1365, 1312, 1233, 1208, 1177, 1143, 1118, 1067, 1022, 1004, 987 cm^{-1}; **^{1}H NMR** (500 MHz, acetone-d_6) δ 7.25 (d, *J* = 11.5 Hz, 1H), 6.63 (dd, *J* = 15.0, 11.5 Hz, 1H), 6.28 (s, 1H), 5.97 (ddd, *J* = 14.6, 9.6, 4.6 Hz, 1H), 5.90 (s, 1H), 5.67 (t, *J* = 8.3 Hz, 1H), 5.23 (d, *J* = 10.5 Hz, 1H), 5.10 (t, *J* = 9.7 Hz, 1H), 4.95 (dd, *J* = 10.3, 3.1 Hz, 1H), 4.83 (s, 1H), 4.79 – 4.71 (m, 1H), 4.69 (s, 1H), 4.61 (d, *J* = 11.4 Hz, 1H), 4.42 (d, *J* = 11.4 Hz, 1H), 4.27 (s, 1H), 4.11 (s, 1H), 4.11 – 4.03 (m, 1H), 3.86 – 3.75 (m, 3H), 3.65 – 3.58 (m, 2H), 3.52 (s, 3H), 3.00 (q, *J* = 7.3 Hz, 2H), 2.79 – 2.60 (m, 3H), 2.54 (s, 3H), 2.52 – 2.42 (m, 2H), 1.97 – 1.89 (m, 1H), 1.83 (d, *J* = 1.4 Hz, 3H), 1.75 (d, *J* = 1.3 Hz, 3H), 1.65 (s, 3H), 1.35 – 1.15 (m, 25H), 0.82 (t, *J* = 7.4 Hz, 3H) ppm; **^{13}C NMR** (126 MHz, acetone-d_6) δ 169.5, 167.8, 164.4, 162.7, 158.2, 155.8, 153.8, 145.4, 143.4, 142.6, 136.9, 136.1, 135.9, 133.9, 128.2, 126.4, 125.3, 124.0, 114.7, 114.5, 110.7, 108.2, 103.9, 101.8, 96.2, 93.0, 81.6, 78.2, 77.6, 75.3, 74.9, 72.9, 72.3, 71.5, 70.9, 70.6, 67.8, 63.4, 61.7, 42.0, 37.3, 33.0, 29.1, 28.9, 28.4, 26.5, 26.2, 20.7, 18.2, 17.8, 17.5, 15.2, 14.4, 13.9, 13.8, 11.1 ppm; **HRMS** ESI(+), (MeOH) calculated for $C_{58}H_{80}Cl_2O_{19}Na$ $[M+Na]^+$: 1173.45631, found: 1173.45632.

7r

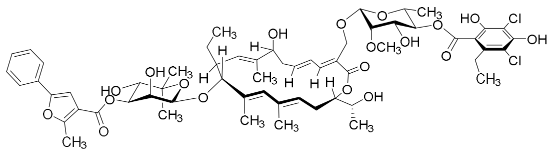

2-Methyl-5-phenylfuran-3-carbonyl chloride (18.1 mg, 81.9 µmol, 3.5 eq.) was added to a stirred mixture of **bisallyl-OP-1118** (**5**) (35.6 mg, 23.4 µmol, 1.0 eq.), boronic acid (679 µg, 2.93 µmol, 12.5 mol%) and DIPEA (15.8 µL, 93.6 µmol, 4.0 eq.) in 1,4-dioxane (125 µL) under ambient atmosphere. The reaction mixture was stirred at 50 °C for 2 h and the reaction was allowed to reach room temperature. $Pd(PPh_3)_4$ (2.70 mg, 2.34 µmol, 10 mol%) followed by morpholine (8.15 µL, 93.6 µmol, 4.0 eq.) were added to the crude mixture under ambient atmosphere and the reaction was stirred at room temperature for 45 min. The solvent was removed under reduced pressure and the crude residue was purified by preparative RP-HPLC [Gemini NX, C18, 5 µ, 110 Å, 250 mm × 21.2 mm, solvent A: H_2O + 0.1% HCOOH, solvent B: MeCN + 0.1% HCOOH, 20 mL/min; LC time program (min –% B): 12 min – 50%, 60 min – 60%, 70 min – 100%] to afford, after lyophilization, **7r** (t_R = 53.4 min, 21.5 mg, 18.3 µmol, 78%) as a slightly yellow solid.

Specific Rotation $[\alpha]_D^{25\,°C}$ = −25.00 (c = 0.38, $CHCl_3$); **FT-IR** $\tilde{\nu}$ (film) 3416, 2975, 2934, 2875, 1704, 1644, 1613, 1589, 1560, 1532, 1450, 1404, 1366, 1312, 1294, 1233, 1199, 1176, 1163, 1144, 1093 cm^{-1}; **^{1}H NMR** (500 MHz, acetone-d_6) δ 7.73 (d, *J* = 7.4 Hz, 2H), 7.44 (t, *J* = 7.8 Hz, 2H), 7.32 (t, *J* = 7.4 Hz, 1H), 7.25 (d, *J* = 11.4 Hz, 1H), 7.08 (s, 1H), 6.80 – 6.59 (m, 1H), 5.97 (ddd, *J* = 14.6, 9.6, 4.6 Hz, 1H), 5.91 (s, 1H), 5.67 (t, *J* = 8.3 Hz, 1H), 5.23 (d, *J* = 11.0 Hz, 1H), 5.10 (t, *J* = 9.7 Hz, 1H), 5.00 (dd, *J* = 10.3, 3.1 Hz, 1H), 4.86 (s, 1H), 4.81 – 4.72 (m, 1H), 4.69 (s, 1H), 4.61 (d, *J* = 11.4 Hz, 1H), 4.43 (d, *J* = 11.4 Hz, 1H), 4.27 (s, 1H), 4.15 (d, *J* = 2.7 Hz, 1H), 4.11 – 4.00 (m, 1H), 3.95 (d, *J* = 10.3 Hz, 1H), 3.86 – 3.78 (m, 2H), 3.69 – 3.57 (m, 2H), 3.52 (s, 3H), 3.04 – 2.96 (m, 2H), 2.83 – 2.62 (m, 3H), 2.67 (s, 3H), 2.56 – 2.42 (m, 2H), 1.96 – 1.90 (m, 1H), 1.83 (d, *J* = 1.3 Hz, 3H), 1.76 (s, 3H), 1.66 (s, 3H), 1.34 – 1.17 (m, 16H), 0.82 (t, *J* = 7.4 Hz, 3H) ppm; **^{13}C NMR** (126 MHz, acetone-d_6) δ 169.5, 167.9, 164.0, 159.6, 155.8, 153.9, 152.5, 145.4, 143.4, 142.6, 136.9, 136.1, 135.9, 134.0, 130.9, 129.7, 128.6, 128.2, 126.4, 125.3, 124.3, 124.0, 116.6, 114.5, 110.7, 108.2, 106.8, 101.8, 96.2, 93.0, 81.6, 78.2, 77.6, 75.3, 75.2, 72.9, 72.3, 71.5, 70.8, 70.6, 67.8, 63.4, 61.7, 42.0, 37.3, 28.9, 28.4, 26.5, 26.2, 20.7, 18.2, 17.8, 17.5, 15.2, 14.4, 14.0, 13.8, 11.1 ppm; **HRMS** ESI(+), (MeOH) calculated for $C_{60}H_{76}Cl_2O_{19}Na$ $[M+Na]^+$: 1193.42501, found: 1193.42554.

7s

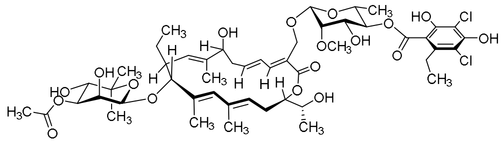

Acetyl chloride (5.84 µL, 81.9 µmol, 3.5 eq.) was added to a stirred mixture of **bisallyl-OP-1118** (**5**) (35.6 mg, 23.4 µmol, 1.0 eq.), boronic acid (679 µg, 2.93 µmol, 12.5 mol%) and DIPEA (15.8 µL, 93.6 µmol, 4.0 eq.) in 1,4-dioxane (125 µL) under ambient atmosphere. The reaction mixture was stirred at 10 °C for 16 h and the reaction was allowed to reach room temperature. $Pd(PPh_3)_4$ (2.70 mg, 2.34 µmol, 10 mol%) followed by morpholine (8.15 µL, 93.6 µmol, 4.0 eq.) were added to the crude mixture under ambient atmosphere and the reaction was stirred at room temperature for 45 min. The solvent was removed under reduced pressure and the crude residue was purified by preparative RP-HPLC [Gemini NX, C18, 5 µ, 110 Å, 250 mm × 21.2 mm, solvent A: H_2O + 0.1% HCOOH, solvent B: MeCN + 0.1% HCOOH, 20 mL/min; LC time program (min –% B): 12 min – 45%, 60 min – 55%, 70 min – 100%] to afford, after lyophilization, **7s** (t_R = 18.1 min, 15.0 mg, 14.6 µmol, 62%) as a slightly yellow solid.

Specific Rotation $[\alpha]_D^{25\,°C}$ = −27.33 (c = 0.29, $CHCl_3$); **FT-IR** $\tilde{\nu}$ (film) 3477, 2977, 2015, 1979, 1704, 1377, 1243, 1067, 772, 524 cm^{-1}; **^{1}H NMR** (500 MHz, acetone-d_6) δ 7.24 (dd, *J* = 11.4, 0.9 Hz, 1H), 6.70 – 6.59 (m, 1H), 5.96 (ddd, *J* = 14.5, 9.5, 4.6 Hz, 1H), 5.88 (s, 1H), 5.76 – 5.58 (m, 1H), 5.22 (d, *J* = 10.6 Hz, 1H), 5.10 (t, *J* = 9.7 Hz, 1H), 4.78 (d, *J* = 1.2 Hz, 1H), 4.77 – 4.73 (m, 2H), 4.68 (d, *J* = 0.9 Hz, 1H), 4.60 (d, *J* = 11.4 Hz, 1H), 4.42 (d, *J* = 11.5 Hz, 1H), 4.26 (s, 1H), 4.08 – 3.99 (m, 2H), 3.85 – 3.75 (m, 3H), 3.68 – 3.58 (m, 2H), 3.52 (s, 3H), 3.08 – 2.94 (m, 2H), 2.81 – 2.74 (m, 1H), 2.73 – 2.68 (m, 1H), 2.67 – 2.58 (m, 1H), 2.54 – 2.41 (m, 2H), 2.02 (s, 3H), 1.97 – 1.88 (m, 1H), 1.81 (d, *J* = 1.3 Hz, 3H), 1.74 (d, *J* = 1.5 Hz, 3H), 1.65 (d, *J* = 0.7 Hz, 3H), 1.31 (d, *J* = 6.2 Hz, 3H), 1.28 – 1.17 (m, 10H), 1.14 (s, 3H), 0.82 (t, *J* = 7.5 Hz, 3H) ppm; **^{13}C NMR** (126 MHz, acetone-d_6)) δ 171.0, 169.5, 167.8, 156.0, 154.2, 145.4, 143.4, 142.6, 136.9, 136.1, 135.9, 133.9, 128.2, 126.4, 125.3, 124.0, 114.7, 110.2, 108.3, 101.8, 96.2, 93.0, 81.6, 78.2, 77.6, 75.2 (2C), 72.9, 72.3, 71.3, 70.7, 70.6, 67.8, 63.4, 61.7, 42.0, 37.3, 28.9, 28.4, 26.5, 26.2, 21.1, 20.7, 18.2, 17.7, 17.5, 15.2, 14.4, 13.8, 11.1 ppm; **HRMS** ESI(+), (MeOH) calculated for $C_{50}H_{70}O_{18}Cl_2Na$ $[M+Na]^+$: 1051.38314, found: 1051.38344.

7t

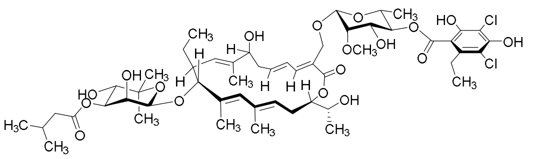

Isovaleryl chloride (10.0 µL, 81.9 µmol, 3.5 eq.) was added to a stirred mixture of **bisallyl-OP-1118** (**5**) (35.6 mg, 23.4 µmol, 1.0 eq.), boronic acid (679 µg, 2.93 µmol, 12.5 mol%) and DIPEA (15.8 µL, 93.6 µmol, 4.0 eq.) in 1,4-dioxane (125 µL) under ambient atmosphere. The reaction mixture was stirred at 10 °C for 16 h and the reaction was allowed to reach room temperature. $Pd(PPh_3)_4$ (2.70 mg, 2.34 µmol, 10 mol%) followed by morpholine (8.15 µL, 93.6 µmol, 4.0 eq.) were added to the crude mixture under ambient atmosphere and the reaction was stirred at room temperature for 45 min. The solvent was removed under reduced pressure and the crude residue was purified by preparative RP-HPLC [Gemini NX, C18, 5 µ, 110 Å, 250 mm × 21.2 mm, solvent A: H_2O + 0.1% HCOOH, solvent B: MeCN + 0.1% HCOOH, 20 mL/min; LC time program (min –% B): 60 min – 50%, 70 min – 100%] to afford, after lyophilization, **7t** (t_R = 28.7 min, 20.0 mg, 17.4 µmol, 74%) as a slightly yellow solid.

Specific Rotation $[\alpha]_D^{25\,°C}$ = −26.42 (c = 0.44, $CHCl_3$); **FT-IR** $\tilde{\nu}$ (film) 3456, 2968, 2934, 2874, 1698, 1590, 1374, 1310, 1244, 1198, 1163, 1143, 1066, 1021, 899, 800, 758, 665, 600 cm^{-1}; **^{1}H NMR** (500 MHz, acetone-*d*$_6$) δ 7.26 (d, *J* = 11.5 Hz, 1H), 6.65 (dd, *J* = 15.0, 11.5 Hz, 1H), 5.98 (ddd, *J* = 14.7, 9.6, 4.6 Hz, 1H), 5.90 (s, 1H), 5.67 (t, *J* = 8.3 Hz, 1H), 5.23 (d, *J* = 10.7 Hz, 1H), 5.12 (t, *J* = 9.7 Hz, 1H), 4.84 – 4.79 (m, 2H), 4.78 – 4.74 (m, 1H), 4.70 (s, 1H), 4.62 (d, *J* = 11.5 Hz, 1H), 4.44 (d, *J* = 11.4 Hz, 1H), 4.28 (s, 1H), 4.09 – 4.02 (m, 2H), 3.86 – 3.78 (m, 3H), 3.69 – 3.59 (m, 2H), 3.54 (s, 3H), 3.02 (q, *J* = 7.3 Hz, 2H), 2.82 – 2.76 (m, 1H), 2.75 – 2.69 (m, 1H), 2.68 – 2.62 (m, 1H), 2.56 – 2.43 (m, 2H), 2.23 (dd, *J* = 7.1, 2.1 Hz, 2H), 2.15 – 2.08 (m, 1H), 1.99 – 1.88 (m, 1H), 1.83 (s, 3H), 1.76 (s, 3H), 1.67 (s, 3H), 1.33 (d, *J* = 6.2 Hz, 3H), 1.27 – 1.19 (m, 10H), 1.16 (s, 3H), 0.97 (d, *J* = 6.6 Hz, 6H), 0.83 (t, *J* = 7.4 Hz, 3H) ppm; **^{13}C NMR** (126 MHz, acetone-*d*$_6$) δ 173.0, 169.5, 167.8, 155.9, 153.9, 145.4, 143.4, 142.6, 136.9, 136.1, 135.9, 133.9, 128.2, 126.4, 125.3, 124.0, 114.6, 110.5, 108.2, 101.8, 96.2, 93.0, 81.6, 78.2, 77.6, 75.2, 74.8, 72.9, 72.3, 71.4, 70.8, 70.6, 67.7, 63.4, 61.7, 43.9, 42.1, 37.3, 28.9, 28.4, 26.5, 26.3, 26.2, 22.7 (2C), 20.7, 18.2, 17.8, 17.5, 15.2, 14.4, 13.8, 11.2 ppm; **HRMS** ESI(+), (MeOH) calculated for $C_{53}H_{76}O_{18}Cl_2Na$ $[M+Na]^+$: 1093.43064, found: 1093.42969.

7u

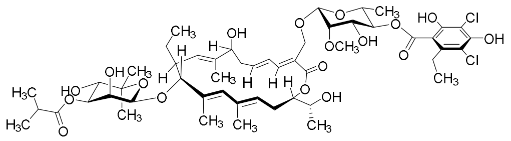

Isobutyryl chloride (8.56 µL, 81.9 µmol, 3.5 eq.) was added to a stirred mixture of **bisallyl-OP-1118** (**5**) (35.6 mg, 23.4 µmol, 1.0 eq.), boronic acid (2.71 mg, 11.7 µmol, 50 mol%) and DIPEA (15.8 µL, 93.6 µmol, 4.0 eq.) in 1,4-dioxane (125 µL) under ambient atmosphere. The reaction mixture was stirred at 50 °C for 16 h and the reaction was allowed to reach room temperature. $Pd(PPh_3)_4$ (2.70 mg, 2.34 µmol, 10 mol%) followed by morpholine (8.15 µL, 93.6 µmol, 4.0 eq.) were added to the crude mixture under ambient atmosphere and the reaction was stirred at room temperature for 45 min. The solvent was removed under reduced pressure and the crude residue was purified by preparative RP-HPLC [Gemini NX, C18, 5 µ, 110 Å, 250 mm × 21.2 mm, solvent A: H_2O + 0.1% HCOOH, solvent B: MeCN + 0.1% HCOOH, 20 mL/min; LC time program (min –% B): 12 min – 40%, 60 min – 60%, 61 min – 100%] to afford, after lyophilization, **7u** (t_R = 35.4 min, 15.1 mg, 14.3 µmol, 61%) as a slightly yellow solid.

Specific Rotation $[\alpha]_D^{25\,°C}$ = −35.55 (c = 0.25, $CHCl_3$); **FT-IR** $\tilde{\nu}$ (film) 3466, 2967, 2932, 1692, 1590, 1382, 1311, 1259, 1199, 1143, 1066, 1021, 898, 799, 758; cm^{-1}; **^{1}H NMR** (500 MHz, acetone-d_6) δ 7.26 (d, *J* = 11.4 Hz, 1H), 6.65 (dd, *J* = 15.0, 11.5 Hz, 1H), 5.98 (ddd, *J* = 14.7, 9.6, 4.6 Hz, 1H), 5.89 (s, 1H), 5.67 (t, *J* = 8.2 Hz, 1H), 5.23 (d, *J* = 10.5 Hz, 1H), 5.12 (t, *J* = 9.7 Hz, 1H), 4.85 – 4.73 (m, 3H), 4.70 (s, 1H), 4.62 (d, *J* = 11.4 Hz, 1H), 4.44 (d, *J* = 11.5 Hz, 1H), 4.28 (s, 1H), 4.10 – 3.97 (m, 2H), 3.91 – 3.77 (m, 3H), 3.71 – 3.60 (m, 2H), 3.54 (s, 3H), 3.09 – 2.96 (m, 2H), 2.83 – 2.76 (m, 1H), 2.75 – 2.69 (m, 1H), 2.68 – 2.64 (m, 1H), 2.63 – 2.57 (m, 1H), 2.55 – 2.44 (m, 2H), 1.97 – 1.89 (m, 1H), 1.83 (s, 3H), 1.76 (s, 3H), 1.67 (s, 3H), 1.33 (d, *J* = 6.2 Hz, 3H), 1.28 – 1.07 (m, 19H), 0.83 (t, *J* = 7.4 Hz, 3H) ppm; **^{13}C NMR** (126 MHz, acetone-d_6) δ 177.1, 169.5, 167.8, 155.9, 154.0, 145.4, 143.4, 142.6, 136.9, 136.1, 135.9, 133.9, 128.2, 126.4, 125.3, 124.0, 114.6, 110.5, 108.2, 101.8, 96.2, 93.0, 81.6, 78.2, 77.6, 75.2, 74.8, 72.9, 72.3, 71.4, 70.7, 70.6, 67.7, 63.4, 61.7, 42.0, 37.3, 34.7, 28.9, 28.4, 26.5, 26.2, 20.7, 19.4, 19.2, 18.2, 17.7, 17.5, 15.2, 14.4, 13.8, 11.2 ppm; **HRMS** ESI(-), (MeOH) calculated for $C_{52}H_{73}Cl_2O_{18}$ $[M-H]^-$: 1055.41588, found: 1055.41794

7w

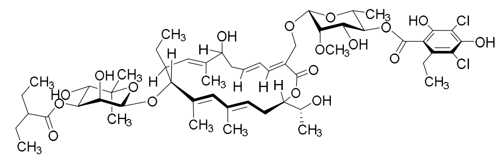

2-Ethylbutyryl chloride (11.2 µL, 81.9 µmol, 3.5 eq.) was added to a stirred mixture of **bisallyl-OP-1118** (**5**) (35.6 mg, 23.4 µmol, 1.0 eq.), boronic acid (2.71 mg, 11.7 µmol, 50 mol%) and DIPEA (15.8 µL, 93.6 µmol, 4.0 eq.) in 1,4-dioxane (125 µL) under ambient atmosphere. The reaction mixture was stirred at 50 °C for 16 h and the reaction was allowed to reach room temperature. $Pd(PPh_3)_4$ (2.70 mg, 2.34 µmol, 10 mol%) followed by morpholine (8.15 µL, 93.6 µmol, 4.0 eq.) were added to the crude mixture under ambient atmosphere and the reaction was stirred at room temperature for 45 min. The solvent was removed under reduced pressure and the crude residue was purified by preparative RP-HPLC [Gemini NX, C18, 5 µ, 110 Å, 250 mm × 21.2 mm, solvent A: H_2O + 0.1% HCOOH, solvent B: MeCN + 0.1% HCOOH, 20 mL/min; LC time program (min –% B): 12 min – 45%, 60 min – 65%, 60.5 min – 100%] to afford, after lyophilization, **7w** (t_R = 36.0 min, 15.1 mg, 13.9 µmol, 59%) as a slightly yellow solid.

Specific Rotation $[\alpha]_D^{25\,°C}$ = −29.56 (c = 0.36, $CHCl_3$); **FT-IR** $\tilde{\nu}$ (film) 3455, 2967, 2933, 1692, 1589, 1459, 1381, 1311, 1242, 1197, 1142, 1066, 1022, 898, 799, 755 cm^{-1}; **^{1}H NMR** (500 MHz, acetone-d_6) δ 7.12 (d, *J* = 11.5 Hz, 1H), 6.51 (dd, *J* = 15.0, 11.5 Hz, 1H), 5.84 (ddd, *J* = 14.6, 9.6, 4.6 Hz, 1H), 5.76 (s, 1H), 5.53 (t, *J* = 8.3 Hz, 1H), 5.09 (d, *J* = 10.3 Hz, 1H), 4.98 (t, *J* = 9.7 Hz, 1H), 4.69 (dd, *J* = 10.4, 3.1 Hz, 1H), 4.67 (s, 1H), 4.67 – 4.59 (m, 1H), 4.56 (s, 1H), 4.48 (d, *J* = 11.5 Hz, 1H), 4.30 (d, *J* = 11.4 Hz, 1H), 4.14 (s, 1H), 3.94 – 3.89 (m, 2H), 3.73 – 3.61 (m, 3H), 3.56 – 3.45 (m, 2H), 3.40 (s, 3H), 2.88 (q, *J* = 7.2 Hz, 2H), 2.65 (m, 1H), 2.61 – 2.56 (m, 1H), 2.55 – 2.47 (m, 1H), 2.44 – 2.27 (m, 2H), 2.15 – 2.08 (m, 1H), 1.84 – 1.76 (m, 1H), 1.69 (s, 3H), 1.62 (s, 3H), 1.57 – 1.47 (m, 5H), 1.45 – 1.35 (m, 2H), 1.19 (d, *J* = 6.1 Hz, 3H), 1.13 – 1.05 (m, 10H), 1.03 (s, 3H), 0.83 – 0.74 (m, 6H), 0.69 (t, *J* = 7.4 Hz, 3H) ppm; **^{13}C NMR** (126 MHz, acetone-d_6) δ 176.1, 169.5, 167.8, 155.9, 154.0, 145.4, 143.4, 142.6, 136.9, 136.1, 135.9, 133.9, 128.2, 126.4, 125.3, 124.0, 114.6, 110.5, 108.2, 101.8, 96.2, 93.1, 81.6, 78.2, 77.6, 75.3, 74.6, 72.9, 72.3, 71.4, 70.9, 70.6, 67.8, 63.4, 61.7, 49.7, 42.1, 37.3, 28.9, 28.4, 26.5, 26.2, 25.8, 25.6, 20.7, 18.2, 17.8, 17.5, 15.2, 14.4, 13.8, 12.1, 12.1, 11.2 ppm; **HRMS** ESI(-), (MeOH) calculated for $C_{54}H_{77}Cl_2O_{18}$ $[M-H]^-$: 1083.44924, found: 1083.44979.

7x

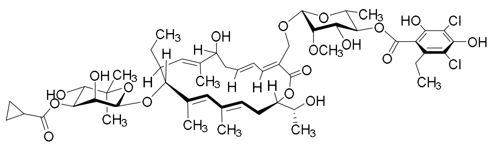

Cyclopropanecarbonyl chloride (7.45 µL, 81.9 µmol, 3.5 eq.) was added to a stirred mixture of **bisallyl-OP-1118** (**5**) (35.6 mg, 23.4 µmol, 1.0 eq.), boronic acid (1.36 mg, 5.85 µmol, 25 mol%) and DIPEA (15.8 µL, 93.6 µmol, 4.0 eq.) in 1,4-dioxane (125 µL) under ambient atmosphere. The reaction mixture was stirred at 50 °C for 16 h and the reaction was allowed to reach room temperature. $Pd(PPh_3)_4$ (2.70 mg, 2.34 µmol, 10 mol%) followed by morpholine (8.15 µL, 93.6 µmol, 4.0 eq.) were added to the crude mixture under ambient atmosphere and the reaction was stirred at room temperature for 45 min. The solvent was removed under reduced pressure and the crude residue was purified by preparative RP-HPLC [Gemini NX, C18, 5 µ, 110 Å, 250 mm × 21.2 mm, solvent A: H_2O + 0.1% HCOOH, solvent B: MeCN + 0.1% HCOOH, 20 mL/min; LC time program (min –% B): 12 min – 40%, 60 min – 60%, 70 min – 100%] to afford, after lyophilization, **7x** (t_R = 36.8 min, 10.0 mg, 9.46 µmol, 41%) as a slightly yellow solid.

Specific Rotation $[\alpha]_D^{25\,°C}$ = −36.59 (c = 0.32, $CHCl_3$); **FT-IR** $\tilde{\nu}$ (film) 3462, 2976, 2934, 1981, 1701, 1590, 1381, 1312, 1243, 1204, 1067, 1022, 952, 900, 764, 520 cm^{-1}; **^{1}H NMR** (500 MHz, acetone-d_6) δ 7.24 (d, *J* = 11.4 Hz, 1H), 6.70 – 6.58 (m, 1H), 5.96 (ddd, *J* = 14.7, 9.6, 4.6 Hz, 1H), 5.87 (s, 1H), 5.65 (t, *J* = 8.3 Hz, 1H), 5.22 (d, *J* = 10.6 Hz, 1H), 5.10 (t, *J* = 9.7 Hz, 1H), 4.81 – 4.72 (m, 3H), 4.68 (s, 1H), 4.60 (d, *J* = 11.4 Hz, 1H), 4.42 (d, *J* = 11.5 Hz, 1H), 4.26 (s, 1H), 4.07 – 3.97 (m, 2H), 3.86 – 3.74 (m, 3H), 3.65 – 3.56 (m, 2H), 3.52 (s, 3H), 3.07 – 2.93 (m, 2H), 2.80 – 2.61 (m, 3H), 2.56 – 2.42 (m, 2H), 1.95 – 1.87 (m, 1H), 1.81 (s, 3H), 1.74 (s, 3H), 1.65 (s, 3H), 1.67 – 1.59 (m, 1H), 1.31 (d, *J* = 6.1 Hz, 3H), 1.25 – 1.17 (m, 10H), 1.14 (s, 3H), 0.94 – 0.90 (m, 2H), 0.90 – 0.84 (m, 2H), 0.82 (t, *J* = 7.4 Hz, 3H) ppm; **^{13}C NMR** (126 MHz, acetone-d_6) δ 174.9, 169.5, 167.8, 155.9, 154.1, 145.4, 143.4, 142.7, 136.9, 136.1, 135.9, 133.9, 128.1, 126.3, 125.3, 124.0, 114.7, 110.3, 108.2, 101.8, 96.2, 93.0, 81.6, 78.2, 77.5, 75.2, 75.1, 72.8, 72.3, 71.3, 70.6 (2C), 67.7, 63.4, 61.7, 42.0, 37.3, 28.9, 28.4, 26.5, 26.3, 20.7, 18.2, 17.7, 17.5, 15.2, 14.4, 13.8, 13.5, 11.1, 8.5, 8.5 ppm; **HRMS** ESI(+), (MeOH) calculated for $C_{52}H_{72}O_{18}Cl_2Na$ $[M+Na]^+$: 1077.39934, found: 1077.39903.

7y

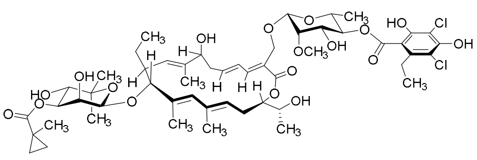

1-Methylcyclopropane-1-carbonyl chloride (9.71 mg, 81.9 µmol, 3.5 eq.) was added to a stirred mixture of **bisallyl-OP-1118** (**5**) (35.6 mg, 23.4 µmol, 1.0 eq.), boronic acid (2.71 mg, 11.7 µmol, 50 mol%) and DIPEA (15.8 µL, 93.6 µmol, 4.0 eq.) in 1,4-dioxane (125 µL) under ambient atmosphere. The reaction mixture was stirred at 50 °C for 16 h and the reaction was allowed to reach room temperature. $Pd(PPh_3)_4$ (2.70 mg, 2.34 µmol, 10 mol%) followed by morpholine (8.15 µL, 93.6 µmol, 4.0 eq.) were added to the crude mixture under ambient atmosphere and the reaction was stirred at room temperature for 45 min. The solvent was removed under reduced pressure and the crude residue was purified by preparative RP-HPLC [Gemini NX, C18, 5 µ, 110 Å, 250 mm × 21.2 mm, solvent A: H_2O + 0.1% HCOOH, solvent B: MeCN + 0.1% HCOOH, 20 mL/min; LC time program (min –% B): 12 min – 45%, 60 min – 65%, 61 min – 100%] to afford, after lyophilization, **7y** (t_R = 29.7 min, 7.3 mg, 6.82 µmol, 29%) as a slightly yellow solid.

Specific Rotation $[\alpha]_D^{25\,°C}$ = −24.40 (c = 0.25, $CHCl_3$); **FT-IR** $\tilde{\nu}$ (film) 3355, 2964, 2925, 1635, 1456, 1381, 1312, 1247, 1176, 1066, 1026, 895, 798 cm^{-1}; **^{1}H NMR** (400 MHz, acetone-d_6) δ 7.24 (d, *J* = 11.5 Hz, 1H), 6.77 – 6.52 (m, 1H), 5.96 (ddd, *J* = 14.6, 9.5, 4.6 Hz, 1H), 5.88 (s, 1H), 5.65 (t, *J* = 8.4 Hz, 1H), 5.22 (d, *J* = 10.6 Hz, 1H), 5.10 (t, *J* = 9.7 Hz, 1H), 4.76 (s, 1H), 4.75 – 4.71 (m, 2H), 4.68 (s, 1H), 4.60 (d, *J* = 11.4 Hz, 1H), 4.42 (d, *J* = 11.5 Hz, 1H), 4.27 (s, 1H), 4.07 – 4.02 (m, 1H), 3.99 (s, 1H), 3.84 – 3.73 (m, 3H), 3.69 – 3.58 (m, 2H), 3.52 (s, 3H), 3.01 (q, *J* = 7.4 Hz, 2H), 2.82 – 2.78 (m, 1H), 2.77 – 2.67 (m, 1H), 2.65 – 2.57 (d, *J* = 9.5 Hz, 1H), 2.53 – 2.40 (m, 2H), 1.97 – 1.87 (m, 1H), 1.81 (s, 3H), 1.74 (s, 3H), 1.65 (s, 3H), 1.32 – 1.17 (m, 18H), 1.13 (s, 3H), 0.82 (t, *J* = 7.4 Hz, 3H), 0.74 – 0.63 (m, 2H) ppm; **^{13}C NMR** (126 MHz, acetone-d_6) δ 175.8, 169.5, 167.8, 156.0, 154.2, 145.4, 143.4, 142.6, 136.9, 136.1, 135.9, 133.9, 128.2, 126.3, 125.3, 124.0, 114.7, 110.2, 108.2, 101.8, 96.1, 93.0, 81.7, 78.2, 77.6, 75.2, 75.1, 72.9, 72.3, 71.3, 70.7, 70.6, 67.7, 63.4, 61.7, 42.0, 37.3, 28.9, 28.4, 26.5, 26.3, 20.7, 19.6, 19.2, 18.2, 17.7, 17.5, 17.0, 16.7, 15.2, 14.4, 13.8, 11.2 ppm; **HRMS** ESI(-), (MeOH) calculated for $C_{53}H_{73}Cl_2O_{18}$ $[M-H]^-$: 1067.41794, found: 1067.41552.

7z

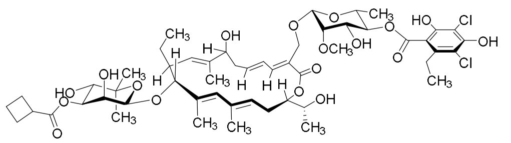

Cyclobutanecarbonyl chloride (9.34 µL, 81.9 µmol, 3.5 eq.) was added to a stirred mixture of **bisallyl-OP-1118** (**5**) (35.6 mg, 23.4 µmol, 1.0 eq.), boronic acid (1.36 mg, 5.85 µmol, 25 mol%) and DIPEA (15.8 µL, 93.6 µmol, 4.0 eq.) in 1,4-dioxane (125 µL) under ambient atmosphere. The reaction mixture was stirred at 50 °C for 16 h and the reaction was allowed to reach room temperature. $Pd(PPh_3)_4$ (2.70 mg, 2.34 µmol, 10 mol%) followed by morpholine (8.15 µL, 93.6 µmol, 4.0 eq.) were added to the crude mixture under ambient atmosphere and the reaction was stirred at room temperature for 45 min. The solvent was removed under reduced pressure and the crude residue was purified by preparative RP-HPLC [Gemini NX, C18, 5 µ, 110 Å, 250 mm × 21.2 mm, solvent A: H_2O + 0.1% HCOOH, solvent B: MeCN + 0.1% HCOOH, 20 mL/min; LC time program (min –% B): 12 min – 45%, 60 min – 65%, 60.5 min – 100%] to afford, after lyophilization, **7z** (t_R = 27.8 min, 15.8 mg, 14.8 µmol, 63%) as a slightly yellow solid.

Specific Rotation $[\alpha]_D^{25\,°C}$ = −25.15 (c = 0.34, $CHCl_3$); **FT-IR** $\tilde{\nu}$ (film) 3439, 2975, 2936, 2874, 1698, 1643, 1589, 1404, 1379, 1312, 1248, 1198, 1162, 1144, 1111, 1066, 1023 cm^{-1}; **^{1}H NMR** (500 MHz, acetone-*d*$_6$) δ 7.26 (d, *J* = 11.4 Hz, 1H), 6.65 (dd, *J* = 15.0, 11.5 Hz, 1H), 5.98 (ddd, *J* = 14.7, 9.6, 4.6 Hz, 1H), 5.90 (s, 1H), 5.67 (t, *J* = 8.2 Hz, 1H), 5.24 (d, *J* = 10.5 Hz, 1H), 5.12 (t, *J* = 9.7 Hz, 1H), 4.87 – 4.74 (m, 3H), 4.70 (s, 1H), 4.62 (d, *J* = 11.4 Hz, 1H), 4.44 (d, *J* = 11.5 Hz, 1H), 4.28 (s, 1H), 4.15 – 3.98 (m, 2H), 3.86 – 3.78 (m, 3H), 3.68 – 3.60 (m, 2H), 3.54 (s, 3H), 3.20 (quint, *J* = 8.4 Hz, 1H), 3.02 (qd, *J* = 7.4, 2.1 Hz, 2H), 2.83 – 2.76 (m, 1H), 2.76 – 2.70 (m, 1H), 2.70 – 2.62 (m, 1H), 2.53 – 2.44 (m, 2H), 2.38 – 2.26 (m, 2H), 2.25 – 2.15 (m, 2H), 2.05 – 1.86 (m, 3H), 1.86 – 1.82 (m, 3H), 1.78 – 1.71 (m, 3H), 1.67 (s, 3H), 1.33 (d, *J* = 6.2 Hz, 3H), 1.29 – 1.19 (m, 10H), 1.16 (s, 3H), 0.83 (t, *J* = 7.4 Hz, 3H) ppm; **^{13}C NMR** (126 MHz, acetone-*d*$_6$) δ 175.4, 169.6, 167.8, 156.1, 154.4, 145.4, 143.4, 142.6, 136.9, 136.1, 135.9, 133.9, 128.2, 126.4, 125.3, 124.0, 114.8, 110.0, 108.2, 101.8, 96.2, 93.0, 81.7, 78.2, 77.5, 75.2, 74.9, 72.9, 72.3, 71.4, 70.7, 70.6, 67.7, 63.4, 61.7, 42.0, 39.0, 37.3, 28.9, 28.4, 26.5, 26.3, 25.9, 25.7, 20.7, 18.9, 18.2, 17.8, 17.5, 15.2, 14.4, 13.8, 11.2 ppm; **HRMS** ESI(-), (MeOH) calculated for $C_{53}H_{73}Cl_2O_{18}$ $[M-H]^-$: 1067.41554, found: 1067.41496.

7aa

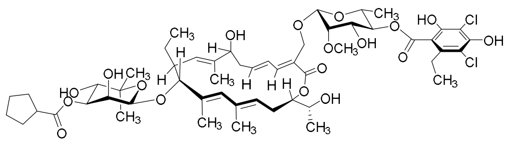

Cyclopentanecarbonyl chloride (9.96 µL, 81.9 µmol, 3.5 eq.) was added to a stirred mixture of **bisallyl-OP-1118** (**5**) (35.6 mg, 23.4 µmol, 1.0 eq.), boronic acid (1.36 mg, 5.85 µmol, 25 mol%) and DIPEA (15.8 µL, 93.6 µmol, 4.0 eq.) in 1,4-dioxane (125 µL) under ambient atmosphere. The reaction mixture was stirred at 50 °C for 16 h and the reaction was allowed to reach room temperature. $Pd(PPh_3)_4$ (2.70 mg, 2.34 µmol, 10 mol%) followed by morpholine (8.15 µL, 93.6 µmol, 4.0 eq.) were added to the crude mixture under ambient atmosphere and the reaction was stirred at room temperature for 45 min. The solvent was removed under reduced pressure and the crude residue was purified by preparative RP-HPLC [Gemini NX, C18, 5 µ, 110 Å, 250 mm × 21.2 mm, solvent A: H_2O + 0.1% HCOOH, solvent B: MeCN + 0.1% HCOOH, 20 mL/min; LC time program (min –% B): 15 min – 45%, 80 min – 65%, 80.5 min – 100%] to afford, after lyophilization, **7aa** (t_R = 43.8 min, 9.50 mg, 8.20 µmol, 35%) as a slightly yellow solid.

Specific Rotation $[\alpha]_D^{25\,°C}$ = −37.34 (c = 0.23, $CHCl_3$); **FT-IR** $\tilde{\nu}$ (film) 3439, 2975, 2936, 2874, 1698, 1643, 1589, 1404, 1379, 1312, 1248, 1198, 1162, 1144, 1111, 1066, 1023 cm^{-1}; **^{1}H NMR** (500 MHz, acetone-d_6) δ 7.24 (d, J = 11.4 Hz, 1H), 6.80 – 6.57 (m, 1H), 5.96 (ddd, J = 14.6, 9.6, 4.6 Hz, 1H), 5.88 (s, 1H), 5.69 – 5.61 (m, 1H), 5.22 (d, J = 10.6 Hz, 1H), 5.10 (t, J = 9.7 Hz, 1H),4.82 – 4.71 (m, 3H), 4.68 (s, 1H), 4.60 (d, J = 11.5 Hz, 1H), 4.42 (d, J = 11.4 Hz, 1H), 4.26 (s, 1H), 4.10 – 3.99 (m, 2H), 3.86 – 3.77 (m, 2H), 3.77 (d, J = 9.7 Hz, 1H), 3.67 – 3.56 (m, 2H), 3.52 (s, 3H), 3.00 (qd, J = 7.4, 1.8 Hz, 2H), 2.86 – 2.72 (m, 2H), 2.75 – 2.66 (m, 1H), 2.70 – 2.58 (m, 1H), 2.55 – 2.40 (m, 2H), 1.94 – 1.89 (m, 1H), 1.88 – 1.82 (m, 4H), 1.81 (d, J = 1.3 Hz, 3H), 1.74 (d, J = 1.4 Hz, 3H), 1.68 – 1.61 (m, 5H), 1.60 – 1.53 (m, 2H), 1.31 (d, J = 6.1 Hz, 3H), 1.26 – 1.16 (m, 10H), 1.14 (s, 3H), 0.82 (t, J = 7.5 Hz, 3H) ppm; **^{13}C NMR** (126 MHz, acetone-d_6) δ 176.7, 169.6, 167.8, 156.0, 154.3, 145.4, 143.4, 142.6, 136.9, 136.1, 135.9, 133.9, 128.2, 126.4, 125.3, 124.0, 114.7, 110.2, 108.2, 101.8, 96.2, 93.0, 81.7, 78.2, 77.6, 75.2, 74.8, 72.9, 72.3, 71.4, 70.7, 70.6, 67.8, 63.4, 61.7, 44.5, 42.1, 37.3, 30.7, 30.4, 28.9, 28.4, 26.5, 26.4, 26.4, 26.3, 20.7, 18.2, 17.8, 17.5, 15.2, 14.4, 13.8, 11.2 ppm; **HRMS** ESI(+), (MeOH) calculated for $C_{54}H_{76}Cl_2O_{18}Na$ $[M+Na]^+$:1105.43009, found: 1105.43035.

7ab

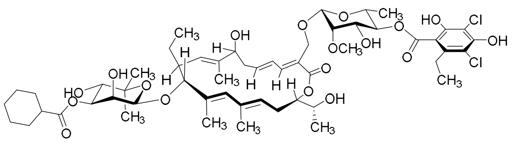

Cyclohexanecarbonyl chloride (10.9 µL, 81.9 µmol, 3.5 eq.) was added to a stirred mixture of **bisallyl-OP-1118** (**5**) (35.6 mg, 23.4 µmol, 1.0 eq.), boronic acid (2.71 mg, 11.7 µmol, 50 mol%) and DIPEA (15.8 µL, 93.6 µmol, 4.0 eq.) in 1,4-dioxane (125 µL) under ambient atmosphere. The reaction mixture was stirred at 50 °C for 16 h and the reaction was allowed to reach room temperature. $Pd(PPh_3)_4$ (2.70 mg, 2.34 µmol, 10 mol%) followed by morpholine (8.15 µL, 93.6 µmol, 4.0 eq.) were added to the crude mixture under ambient atmosphere and the reaction was stirred at room temperature for 45 min. The solvent was removed under reduced pressure and the crude residue was purified by preparative RP-HPLC [Gemini NX, C18, 5 µ, 110 Å, 250 mm × 21.2 mm, solvent A: H_2O + 0.1% HCOOH, solvent B: MeCN + 0.1% HCOOH, 20 mL/min; LC time program (min –% B): 12 min – 50%, 60 min – 70%, 61 min – 100%] to afford, after lyophilization, **7ab** (t_R = 28.1 min, 17.6 mg, 16.0 µmol, 69%) as a slightly yellow solid.

Specific Rotation $[\alpha]_D^{25\,°C}$ = −31.25 (c = 0.30, $CHCl_3$); **FT-IR** $\tilde{\nu}$ (film) 3449, 2930, 2856, 1692, 1590, 1452, 1405, 1380, 1312, 1246, 1198, 1176, 1162, 1134, 1066, 1023, 949, 897, 800, 757 cm^{-1}; **^{1}H NMR** (500 MHz, acetone-*d*$_6$) δ 7.24 (d, *J* = 11.4 Hz, 1H), 6.63 (dd, *J* = 15.0, 11.5 Hz, 1H), 5.96 (ddd, *J* = 14.7, 9.6, 4.6 Hz, 1H), 5.88 (s, 1H), 5.65 (t, *J* = 8.3 Hz, 1H), 5.22 (d, *J* = 10.6 Hz, 1H), 5.10 (t, *J* = 9.7 Hz, 1H), 4.77 (s, 1H), 4.76 – 4.71 (m, 2H), 4.68 (s, 1H), 4.60 (d, *J* = 11.4 Hz, 1H), 4.42 (d, *J* = 11.4 Hz, 1H), 4.26 (s, 1H), 4.10 – 3.99 (m, 2H), 3.85 – 3.79 (m, 2H), 3.77 (d, *J* = 9.7 Hz, 1H), 3.65 – 3.59 (m, 2H), 3.52 (s, 3H), 3.00 (q, *J* = 7.4 Hz, 2H), 2.84 – 2.74 (m, 1H), 2.73 – 2.67 (m, 1H), 2.67 – 2.59 (m, 1H), 2.55 – 2.41 (m, 2H), 2.34 (tt, *J* = 11.0, 3.7 Hz, 1H), 1.95 – 1.87 (m, 3H), 1.81 (s, 3H), 1.74 (s, 3H), 1.73 – 1.70 (m, 2H), 1.65 (s, 3H), 1.64 – 1.60 (m, 1H), 1.50 – 1.39 (m, 2H), 1.35 – 1.17 (m, 16H), 1.14 (s, 3H), 0.82 (t, *J* = 7.4 Hz, 3H) ppm; **^{13}C NMR** (126 MHz, acetone-*d*$_6$) δ 175.9, 169.5, 167.8, 155.9, 153.9, 145.4, 143.4, 142.6, 136.9, 136.1, 135.9, 133.9, 128.2, 126.4, 125.3, 124.0, 114.6, 110.6, 108.2, 101.8, 96.2, 93.0, 81.6, 78.2, 77.6, 75.2, 74.7, 72.9, 72.3, 71.4, 70.7, 70.6, 67.8, 63.4, 61.7, 43.8, 42.0, 37.3, 28.9, 28.4, 26.6, 26.5, 26.2, 26.1, 26.0, 20.7, 18.2, 17.8, 17.5, 15.2, 14.4, 13.8, 11.2 ppm; **HRMS** ESI(-), (MeOH) calculated for $C_{55}H_{77}Cl_2O_{18}$ $[M-H]^-$: 1095.44924, found: 1095.44910.

7ad

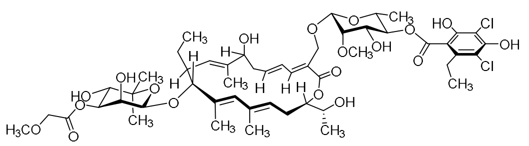

Methoxyacetyl chloride (7.47 µL, 81.9 µmol, 3.5 eq.) was added to a stirred mixture of **bisallyl-OP-1118** (**5**) (35.6 mg, 23.4 µmol, 1.0 eq.), boronic acid (679 µg, 2.93 µmol, 12.5 mol%) and DIPEA (15.8 µL, 93.6 µmol, 4.0 eq.) in 1,4-dioxane (125 µL) under ambient atmosphere. The reaction mixture was stirred at 30 °C for 16 h and the reaction was allowed to reach room temperature. $Pd(PPh_3)_4$ (2.70 mg, 2.34 µmol, 10 mol%) followed by morpholine (8.15 µL, 93.6 µmol, 4.0 eq.) were added to the crude mixture under ambient atmosphere and the reaction was stirred at room temperature for 45 min. The solvent was removed under reduced pressure and the crude residue was purified by preparative RP-HPLC [Gemini NX, C18, 5 µ, 110 Å, 250 mm × 21.2 mm, solvent A: H_2O + 0.1% HCOOH, solvent B: MeCN + 0.1% HCOOH, 20 mL/min; LC time program (min –% B): 12 min – 45%, 60 min – 50%, 70 min – 100%] to afford, after lyophilization, **7ad** (t_R = 17.6 min, 16.0 mg, 15.1 µmol, 65%) as a slightly yellow solid.

Specific Rotation $[\alpha]_D^{25\,°C}$ = −34.52 (c = 0.27, $CHCl_3$); **FT-IR** $\tilde{\nu}$ (film) 3452, 2975, 2934, 2170, 1741, 1693, 1642, 1587, 1452, 1377, 1310, 1245, 1197, 1120, 1066, 1022, 899, 758, 663, 592, 516 cm^{-1}; **^{1}H NMR** (500 MHz, acetone-d_6) δ 7.24 (d, *J* = 11.4 Hz, 1H), 6.69 – 6.51 (m, 1H), 5.96 (ddd, *J* = 14.7, 9.6, 4.6 Hz, 1H), 5.88 (s, 1H), 5.65 (t, *J* = 8.1 Hz, 1H), 5.22 (d, *J* = 10.5 Hz, 1H), 5.10 (t, *J* = 9.7 Hz, 1H), 4.86 (dd, *J* = 10.3, 3.1 Hz, 1H), 4.81 (s, 1H), 4.77 – 4.72 (m, 1H), 4.68 (s, 1H), 4.60 (d, *J* = 11.5 Hz, 1H), 4.42 (d, *J* = 11.5 Hz, 1H), 4.26 (s, 1H), 4.08 – 4.02 (m, 4H), 3.85 – 3.75 (m, 3H), 3.67 – 3.57 (m, 2H), 3.52 (s, 3H), 3.38 (s, 3H), 3.00 (q, *J* = 7.4, 1.4Hz, 2H), 2.80 – 2.59 (m, 3H), 2.55 – 2.40 (m, 2H), 1.95 – 1.88 (m, 1H), 1.81 (d, *J* = 1.2 Hz, 3H), 1.74 (d, *J* = 1.3 Hz, 3H), 1.65 (s, 3H), 1.31 (d, *J* = 6.1 Hz, 3H), 1.25 – 1.16 (m, 10H), 1.15 (s, 3H), 0.82 (t, *J* = 7.5 Hz, 3H) ppm; **^{13}C NMR** (126 MHz, acetone-d_6) δ 170.8, 169.4, 167.8, 155.8, 153.8, 145.4, 143.4, 142.7, 136.9, 136.1, 135.9, 133.9, 128.1, 126.4, 125.3, 124.0, 114.5, 110.6, 108.1, 101.8, 96.1, 93.0, 81.6, 78.2, 77.6, 75.4, 75.2, 72.8, 72.3, 71.2, 70.6 (2C), 70.0, 67.6, 63.4, 61.7, 59.1, 42.0, 37.2, 28.9, 28.4, 26.5, 26.2, 20.6, 18.2, 17.7, 17.5, 15.2, 14.4, 13.8, 11.1 ppm; **HRMS** ESI(+), (MeOH) calculated for $C_{51}H_{72}O_{19}Cl_2Na$ $[M+Na]^+$: 1081.39426, found: 1081.39396.

7ag

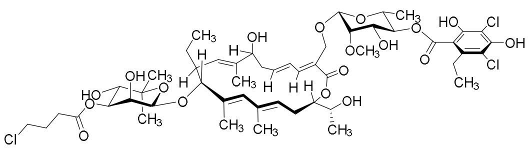

4-Chlorobutyryl chloride (9.35 µL, 81.9 µmol, 3.5 eq.) was added to a stirred mixture of **bisallyl-OP-1118** (**5**) (35.6 mg, 23.4 µmol, 1.0 eq.), boronic acid (679 µg, 2.93 µmol, 12.5 mol%) and DIPEA (15.8 µL, 93.6 µmol, 4.0 eq.) in 1,4-dioxane (125 µL) under ambient atmosphere. The reaction mixture was stirred at 40 °C for 16 h and the reaction was allowed to reach room temperature. $Pd(PPh_3)_4$ (2.70 mg, 2.34 µmol, 10 mol%) followed by morpholine (8.15 µL, 93.6 µmol, 4.0 eq.) were added to the crude mixture under ambient atmosphere and the reaction was stirred at room temperature for 45 min. The solvent was removed under reduced pressure and the crude residue was purified by preparative RP-HPLC [Gemini NX, C18, 5 µ, 110 Å, 250 mm × 21.2 mm, solvent A: H_2O + 0.1% HCOOH, solvent B: MeCN + 0.1% HCOOH, 20 mL/min LC time program (min –% B): 12 min – 50%, 60 min – 55%, 70 min – 100%] to afford, after lyophilization, **7ag** (t_R = 23.5 min, 16.8 mg, 15.4 µmol, 66%) as a slightly yellow solid.

Specific Rotation $[\alpha]_D^{25\,°C}$ = −15.73 (c = 0.39, $CHCl_3$); **FT-IR** $\tilde{\nu}$ (film) 2976, 2034, 1693, 1242, 1066, 771 cm^{-1}; **^{1}H NMR** (500 MHz, acetone-*d*6) δ 7.24 (d, *J* = 11.5 Hz, 1H), 6.68 – 6.46 (m, 1H), 5.96 (ddd, *J* = 14.7, 9.6, 4.6 Hz, 1H), 5.88 (s, 1H), 5.65 (t, *J* = 8.2 Hz, 1H), 5.20 (d, *J* = 1.6 Hz, 1H), 5.10 (t, *J* = 9.7 Hz, 1H), 4.84 – 4.79 (m, 1H), 4.79 (s, 1H), 4.78 – 4.71 (m, 1H), 4.68 (s, 1H), 4.60 (d, *J* = 11.5 Hz, 1H), 4.42 (d, *J* = 11.5 Hz, 1H), 4.26 (s, 1H), 4.06 – 4.00 (m, 2H), 3.84 – 3.75 (m, 3H), 3.69 (t, *J* = 6.6 Hz, 2H), 3.64 – 3.59 (m, 2H), 3.52 (s, 3H), 3.00 (q, *J* = 7.3 Hz, 2H), 2.81 – 2.74 (m, 1H), 2.73 – 2.60 (m, 2H), 2.54 (t, *J* = 7.2 Hz, 2H), 2.52 – 2.42 (m, 2H), 2.12 – 2.06 (m, 2H), 1.95 – 1.88 (m, 1H), 1.81 (s, 3H), 1.74 (d, *J* = 1.3 Hz, 3H), 1.65 (d, *J* = 1.2 Hz, 3H), 1.31 (d, *J* = 6.1 Hz, 3H), 1.28 – 1.17 (m, 10H), 1.14 (s, 3H), 0.82 (t, *J* = 7.4 Hz, 3H) ppm; **^{13}C NMR** (126 MHz, acetone-*d*6) δ 172.9, 169.4, 167.8, 155.8, 153.8, 145.4, 143.4, 142.7, 136.9, 136.1, 135.9, 133.9, 128.1, 126.4, 125.3, 124.0, 114.5, 110.6, 108.2, 101.8, 96.1, 93.0, 81.6, 78.2, 77.6, 75.3, 75.2, 72.8, 72.3, 71.3, 70.6 (2C), 67.7, 63.4, 61.7, 45.0, 42.0, 37.3, 32.0, 28.9, 28.8, 28.4, 26.5, 26.2, 20.7, 18.2, 17.7, 17.5, 15.2, 14.4, 13.8, 11.1 ppm; **HRMS** ESI(+), (MeOH) calculated for $C_{52}H_{73}O_{18}Cl_3Na$ $[M+Na]^+$: 1113.37602, found: 1113.37442.

7ah

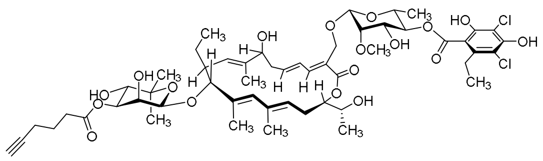

Hex-5-ynoyl chloride (10.7 mg, 81.9 µmol, 3.5 eq.) was added to a stirred mixture of **bisallyl-OP-1118** (**5**) (35.6 mg, 23.4 µmol, 1.0 eq.), boronic acid (679 µg, 2.93 µmol, 12.5 mol%) and DIPEA (15.8 µL, 93.6 µmol, 4.0 eq.) in 1,4-dioxane (125 µL) under ambient atmosphere. The reaction mixture was stirred at 30 °C for 16 h and the reaction was allowed to reach room temperature. $Pd(PPh_3)_4$ (2.70 mg, 2.34 µmol, 10 mol%) followed by morpholine (8.15 µL, 93.6 µmol, 4.0 eq.) were added to the crude mixture under ambient atmosphere and the reaction was stirred at room temperature for 45 min. The solvent was removed under reduced pressure and the crude residue was purified by preparative RP-HPLC [Gemini NX, C18, 5 µ, 110 Å, 250 mm × 21.2 mm, solvent A: H_2O + 0.1% HCOOH, solvent B: MeCN + 0.1% HCOOH, 20 mL/min LC time program (min –% B): 12 min – 50%, 60 min – 60%, 70 min – 100%] to afford, after lyophilization, **7ah** (t_R = 21.8 min, 17.5 mg, 16.2 µmol, 69%) as a slightly yellow solid.

Specific Rotation $[\alpha]_D^{25\,°C}$ = −38.70 (c = 0.24, $CHCl_3$); **FT-IR** $\tilde{\nu}$ (film) 3453, 2975, 2934, 1692, 1589, 1379, 1312, 1243, 1144, 1066, 1021, 899, 800, 758, 638, 514 cm^{-1}; **^{1}H NMR** (400 MHz, acetone-*d*6) δ 7.24 (d, *J* = 11.4 Hz, 1H), 6.63 (dd, *J* = 15.0, 11.5 Hz, 1H), 5.97 (ddd, *J* = 14.6, 9.5, 4.6 Hz, 1H), 5.88 (s, 1H), 5.65 (t, *J* = 8.3 Hz, 1H), 5.22 (d, *J* = 10.5 Hz, 1H), 5.10 (t, *J* = 9.7 Hz, 1H), 4.83 – 4.77 (m, 2H), 4.77 – 4.71 (m, 1H), 4.68 (s, 1H), 4.60 (d, *J* = 11.4 Hz, 1H), 4.42 (d, *J* = 11.5 Hz, 1H), 4.26 (s, 1H), 4.12 – 3.96 (m, 2H), 3.84 – 3.74 (m, 3H), 3.66 – 3.57 (m, 2H), 3.52 (s, 3H), 3.06 – 2.96 (m, 2H), 2.84 – 2.59 (m, 3H), 2.56 – 2.41 (m, 4H), 2.37 (t, *J* = 2.7 Hz, 1H), 2.27 (td, *J* = 7.1, 2.6 Hz, 2H), 1.95 – 1.88 (m, 1H), 1.87 – 1.78 (m, 5H), 1.74 (s, 3H), 1.65 (s, 3H), 1.31 (d, *J* = 6.2 Hz, 3H), 1.26 – 1.17 (m, 10H), 1.14 (s, 3H), 0.82 (t, *J* = 7.4 Hz, 3H) ppm; **^{13}C NMR** (126 MHz, acetone-*d*6) δ 173.2, 169.6, 167.8, 156.1, 154.5, 145.4, 143.3, 142.6, 136.9, 136.1, 135.9, 133.9, 128.2, 126.4, 125.3, 124.0, 114.8, 109.9, 108.2, 101.8, 96.2, 93.0, 84.3, 81.7, 78.2, 77.5, 75.2, 75.2, 72.8, 72.3, 71.3, 70.6 (2C), 70.4, 67.7, 63.4, 61.7, 42.0, 37.3, 33.6, 28.9, 28.4, 26.5, 26.3, 24.8, 20.7, 18.2, 18.2, 17.7, 17.5, 15.2, 14.4, 13.8, 11.2 ppm; **HRMS** ESI(+), (MeOH) calculated for $C_{54}H_{74}O_{18}Cl_2Na$ $[M+Na]^+$: 1103.41444, found: 1103.41499.

7ai

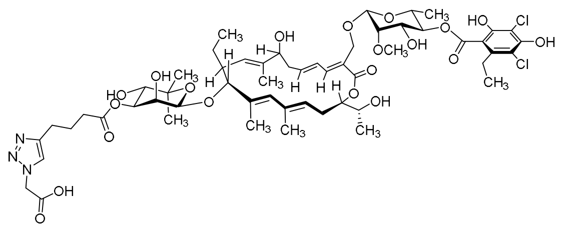

A vial under an inert atmosphere was charged with **7ah** (10 mg, 9.25 µmol, 1.0 eq.) and **Dinuclear Copper Catalyst** (0.65 mg, 0.92 µmol, 0.1 eq.). The solids were dissolved in DCM (100 µL) and a solution of azidoacetic acid (1.38 µL, 18.5 µmol, 2 eq.) in THF (100 µL) was added at room temperature. The reaction mixture was stirred at room temperature until full conversion was observed (UHPLC-MS).The solvent was removed under reduced pressure and the crude residue was purified by preparative RP-HPLC [Gemini NX, C18, 5 µ, 110 Å, 250 mm × 21.2 mm, solvent A: H_2O + 0.1% HCOOH, solvent B: MeCN + 0.1% HCOOH, 20 mL/min LC time program (min –% B): 12 min – 50%, 60 min – 60%, 61 min – 100%] to afford, after lyophilization, **7ai** (t_R = 10.3 min, 10.0 mg, 8.45 µmol, 91%) as a slightly yellow solid.

Specific Rotation $[\alpha]_D^{25\,°C}$ = −8.39 (c = 0.37, MeOH); **FT-IR** $\tilde{\nu}$ (film) 3425, 2974, 2934, 1710, 1379, 1312, 1242, 1142, 1066, 1022, 900, 800, 772, 600 cm^{-1}; **^{1}H NMR** (500 MHz, acetone-*d*$_6$) δ 7.81 (s, 1H), 7.24 (d, J = 11.5 Hz, 1H), 6.63 (dd, J = 15.0, 11.5 Hz, 1H), 5.97 (ddd, J = 14.7, 9.6, 4.6 Hz, 1H), 5.88 (s, 1H), 5.65 (t, J = 8.3 Hz, 1H), 5.28 (s, 2H), 5.22 (d, J = 10.5 Hz, 1H), 5.10 (t, J = 9.7 Hz, 1H), 4.84 – 4.79 (m, 1H), 4.79 (s, 1H), 4.74 (q, J = 5.3 Hz, 1H), 4.68 (s, 1H), 4.60 (d, J = 11.4 Hz, 1H), 4.42 (d, J = 11.5 Hz, 1H), 4.26 (s, 1H), 4.06 – 4.00 (m, 2H), 3.86 – 3.79 (m, 2H), 3.77 (d, J = 9.8 Hz, 1H), 3.67 – 3.58 (m, 2H), 3.52 (s, 3H), 3.05 – 2.96 (m, 2H), 2.83 – 2.73 (m, 3H), 2.73 – 2.68 (m, 1H), 2.67 – 2.60 (m, 1H), 2.54 – 2.42 (m, 2H), 2.39 (td, J = 7.3, 2.1 Hz, 2H), 2.02 – 1.95 (m, 2H), 1.91 (m, 1H), 1.81 (s, 3H), 1.74 (s, 3H), 1.65 (s, 3H), 1.31 (d, J = 6.1 Hz, 3H), 1.26 – 1.17 (m, 10H), 1.15 (s, 3H), 0.81 (t, J = 7.4 Hz, 3H) ppm; **^{13}C NMR** (126 MHz, acetone-*d*$_6$) δ 173.4, 169.4, 168.7, 167.8, 155.7, 153.7, 147.5, 145.5, 143.5, 142.7, 136.8, 136.1, 136.0, 133.9, 128.1, 126.3, 125.3, 124.0, 123.9, 114.5, 110.6, 108.1, 101.7, 96.2, 93.0, 81.6, 78.2, 77.6, 75.2, 75.2, 72.8, 72.2, 71.2, 70.6 (2C), 67.6, 63.4, 61.7, 51.1, 42.0, 37.2, 33.9, 28.9, 28.4, 26.5, 26.2, 25.7, 25.2, 20.6, 18.2, 17.8, 17.5, 15.2, 14.4, 13.8, 11.2 ppm; **HRMS** ESI(+), (MeOH) calculated for $C_{56}H_{77}O_{20}Cl_2N_3Na$ [M+Na]$^+$: 1204.43697, found: 1204.43724.

Experimental Procedures Tsuji-Trost Functionalizations

9

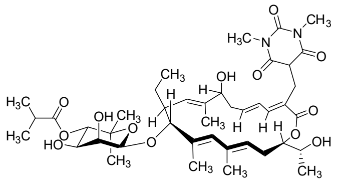

In a flame-dried microwave tube, a mixture of fidaxomicin (**1**, 30.0 mg, 28.4 µmol, 1.0 eq.), 1,3-dimethylbarbituric acid (17.7 mg, 0.114 mmol, 4.0 eq.), $Pd(OAc)_2$ (3.19 mg, 14.2 µmol, 50 mol%) and Xantphos (16.4 mg, 28.4 µmol, 1.0 eq.) was dissolved in dry toluene (500 µL, degassed by freeze-pump-thaw (3x)). The microwave tube was evacuated, flushed with argon several times and sealed. The mixture was heated to 90 °C for 5 h and then diluted with EtOAc (2 mL) and washed with H_2O (3 x 2 mL). The organic phase was dried over $MgSO_4$ and the solvent was evaporated under reduced pressure. The crude residue was purified by preparative RP-HPLC [Gemini NX, C18, 10 µ, 110 Å, 250 mm × 21.2 mm, solvent A: H_2O + 0.1% HCOOH, solvent B: MeCN + 0.1% HCOOH, 20 mL/min; LC time program (min –% B): 0.0 min – 5%, 10 min – 5%, 12 min – 40%, 55 min – 60%, 56 min – 100%] to afford, after lyophilization, barbituric acid **9** (t_R = 30.1 min, 9.5 mg, 12 µmol, 42%) as a slightly yellow solid.

TLC (pentane:acetone, 2:3 v/v) = 0.13; **Specific Rotation** $[\alpha]_D^{26\,°C}$ = +32.42 (c = 0.29, MeOH); **FT-IR** $\tilde{\nu}$ (film) 3386, 2972, 2970, 2874, 2857, 1677, 1585, 1446, 1424, 1381, 1292, 1253, 1198, 1147, 1074, 1031, 951, 899, 842, 797, 756, 717, 674, 584, 505, 472 cm^{-1}; **^{1}H NMR** (500 MHz, CD_3OD) δ 7.14 (d, *J* = 11.4 Hz, 1H), 6.40 (dd, *J* = 14.6, 12.2 Hz, 1H), 5.89 (ddd, *J* = 14.6, 9.4, 4.7 Hz, 1H), 5.81 (s, 1H), 5.54 (t, *J* = 8.3 Hz, 1H), 5.13 (dt, *J* = 10.5, 1.6 Hz, 1H), 5.01 (d, *J* = 10.2 Hz, 1H), 4.71 (s, 1H), 4.63 (dt, *J* = 6.9, 5.0 Hz, 1H), 4.22 (m, 1H), 3.95 (quint, *J* = 6.4 Hz, 1H), 3.92 (d, J = 3.1 Hz, 1H), 3.75 – 3.68 (m, 3H), 3.18 (s, 3H), 3.17 (s, 3H), 3.12 (d, *J* = 14.3 Hz, 1H), 3.05 (d, *J* = 14.2 Hz, 1H), 2.74 – 2.63 (m, 3H), 2.59 (sept, *J* = 7.0 Hz, 1H), 2.48 (ddd, *J* = 16.2, 9.4, 4.4 Hz, 1H), 2.39 (ddd, *J* = 13.9, 9.0, 4.5 Hz, 1H), 2.05 – 1.96 (m, 1H), 1.79 (d, *J* = 1.3 Hz, 3H), 1.70 (d, *J* = 1.3 Hz, 3H), 1.65 (s, 3H), 1.33 – 1.22 (m, 1H), 1.19 – 1.10 (m, 15H), 0.88 (t, *J* = 7.5 Hz, 3H) ppm; **^{13}C NMR** (126 MHz, CD_3OD) δ 178.4, 170.4, 170.2, 169.3, 153.4, 144.9, 143.0, 136.94, 136.92, 136.3, 134.6, 128.5, 126.5, 124.6, 124.4, 97.2, 94.3, 78.7, 75.9, 74.6, 73.5, 73.2, 70.5, 68.2, 49.9, 42.5, 37.2, 35.4, 29.1, 28.8, 28.73, 28.70, 28.4, 26.9, 20.4, 19.5, 19.1, 18.7, 17.5, 15.4, 13.9, 11.3 ppm; **HRMS** ESI(+), (MeOH) calculated for $C_{42}H_{62}N_2O_{13}Na$ $[M+Na]^+$: 825.41441, found: 825.41465.

10

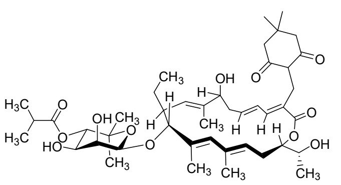

In a flame-dried microwave tube, a mixture of fidaxomicin (**1**, 20.3 mg, 19.2 µmol, 1.0 eq.), dimedone (10.6 mg, 75.6 µmol, 4.0 eq.) and $Pd(PPh_3)_4$ (5.0 mg, 4.3 µmol, 25 mol%) was dissolved in dry toluene (100 µL, degassed by freeze-pump-thaw (3x)). The microwave tube was evacuated and flushed with argon several times and the tube was sealed. The mixture was heated to 90 °C for 5 h and then diluted with EtOAc (2 mL) and washed with H_2O (3 x 2 mL). The organic phase was dried over $MgSO_4$ and the solvent was evaporated under reduced pressure. The crude residue was purified by preparative RP-HPLC [Gemini NX, C18, 5 µ, 110 Å, 250 mm × 21.2 mm, solvent A: H_2O + 0.1% HCOOH, solvent B: MeCN + 0.1% HCOOH, 20 mL/min; LC time program (min –% B): 0.0 min – 5%, 10 min – 5%, 12 min – 40%, 55 min –60%, 56 min – 100%] to afford, after lyophilization, dimedone **10** (t_R = 38.2 min, 4.10 mg, 5.2 µmol, 27%) as a slightly yellow solid.

TLC (pentane:acetone, 1:1 v/v) = 0.82; **Specific Rotation** $[\alpha]_D^{24\,°C}$ = −12.86 (c = 0.22, MeOH); **FT-IR** $\tilde{\nu}$ (film) 3421, 2971, 2933, 2874, 1732, 1698, 1656, 1637, 1607, 1470, 1385, 1370, 1355, 1292, 1257, 1208, 1150, 1078, 1034, 897, 797, 712 cm^{-1}; **^{1}H NMR** (500 MHz, CD_3OD) δ 6.86 (d, *J* = 11.3 Hz, 1H), 6.65 – 6.55 (m, 1H), 5.83 (s, 1H), 5.71 (ddd, *J* = 14.7, 9.4, 4.9 Hz, 1H), 5.53 (t, *J* = 8.2 Hz, 1H), 5.09 (dt, *J* = 10.4, 1.6 Hz, 1H), 5.01 (d, *J* = 10.3 Hz, 1H), 4.70 (s, 1H), 4.66 (q, *J* = 6.2 Hz, 1H), 4.17 (s, 1H), 4.04 (quint, *J* = 6.3 Hz, 1H), 3.91 (d, *J* = 2.8 Hz, 1H), 3.72 (dd, *J* = 10.2, 3.2 Hz, 1H), 3.69 (d, *J* = 9.8 Hz, 1H), 3.32 (d, *J* = 14.5 Hz, 1H), 3.25 (d, *J* = 14.6 Hz, 1H), 2.77 – 2.54 (m, 4H), 2.48 – 2.33 (m, 2H), 2.23 (s, 4H), 2.01 (m, 1H), 1.78 (d, *J* = 1.3 Hz, 3H), 1.76 (d, *J* = 1.4 Hz, 3H), 1.63 (d, *J* = 1.3 Hz, 3H), 1.34 – 1.22 (m, 1H), 1.20 – 1.09 (m, 15H), 1.00 (s, 6H), 0.87 (t, *J* = 7.4 Hz, 3H) ppm; **^{13}C NMR** (126 MHz, CD_3OD) δ 208.4 (HMBC), 178.4, 172.6, 142.0, 140.0, 137.1, 136.9, 136.2, 134.7, 130.1, 129.6, 127.1, 124.6, 114.0, 97.1, 94.3, 79.1, 76.0, 74.5, 73.7, 73.2, 70.6, 68.3, 48.3 (HMBC), 42.6, 37.37, 35.4, 32.8, 28.7, 28.5, 28.3, 28.2, 26.9, 20.2, 19.5, 19.1, 18.7, 17.4, 15.5, 13.9, 11.3 ppm; **HRMS** ESI(+), (MeOH) calculated for $C_{44}H_{66}O_{12}Na$ $[M+Na]^+$: 809.44465, found: 809.44499.

11

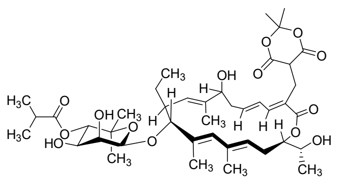

A flame-dried microwave tube was charged with fidaxomicin (**1**, 100 mg, 94.5 µmol, 1.0 eq.), Meldrum's acid (54.5 mg, 0.378 mmol, 4.0 eq.) and $Pd(PPh_3)_4$ (27.3 mg, 23.6 µmol, 25 mol%). The tube was sealed and evacuated and flushed with argon several times. Then, the solids were dissolved in dry toluene (1.9 mL, degassed by freeze-pump-thaw (3x)) and the mixture was stirred for 3 h at 70 °C. After evaporation of the solvent the crude mixture was purified by RP-HPLC [Gemini NX, C18, 5 µ, 110 Å, 250 mm × 21.2 mm, solvent A: H_2O + 0.1% HCOOH, solvent B: MeCN + 0.1% HCOOH, 20 mL/min; LC time program (min –% B): 0.0 min – 5%, 10 min – 5%, 11 min – 55%, 55 min – 60%, 56 min – 100%] to afford, after lyophilization, Meldrum's acid **11** (t_R = 19.7 min, 19.5 mg, 24.6 µmol, 26%) as a colorless solid.

TLC (pentane:acetone, 1:1 v/v) = 0.30; **Specific Rotation** $[\alpha]_D^{24\,°C}$ = +36.52 (c = 0.69, MeOH); **FT-IR** $\tilde{\nu}$ (film) 3452, 2976, 2934, 2876, 1782, 1744, 1690, 1641, 1573, 1438, 1385, 1299, 1254, 1205, 1151, 1075, 1031, 948, 898, 798, 718 cm^{-1}; **^{1}H NMR** (500 MHz, CD_3OD) δ 7.10 (d, *J* = 11.4 Hz, 1H), 6.54 (dd, *J* = 14.9, 11.9 Hz, 1H), 5.87 (ddd, *J* = 14.6, 9.6, 4.9 Hz, 1H), 5.83 (s, 1H), 5.54 (t, *J* = 8.1 Hz, 1H), 5.13 (dt, *J* = 10.5 Hz, 1.6 Hz, 1H), 5.02 (d, *J* = 10.3 Hz, 1H), 4.71 (d, *J* = 1.2 Hz, 1H), 4.66 (td, *J* = 6.1, 4.3 Hz, 1H), 4.21 (m, 1H), 3.99 (quint, *J* = 6.3 Hz, 1H), 3.92 (dd, *J* = 3.3, 1.1 Hz, 1H), 3.76 – 3.68 (m, 2H), 3.12 (d, *J* = 15.3 Hz, 1H), 3.03 (d, *J* = 15.4 Hz, 1H), 2.76 – 2.64 (m, 3H), 2.59 (sept, *J* = 7.0 Hz, 1H), 2.49 (ddd, *J* = 14.9, 9.5, 4.5 Hz, 1H), 2.40 (ddd, *J* = 13.8, 8.6, 4.4 Hz, 1H), 2.07 – 1.95 (m, 1H), 1.81 (s, 3H), 1.80 (s, 3H), 1.76 (s, 3H), 1.74 (s, 3H), 1.66 (s, 3H), 1.33 – 1.24 (m, 1H), 1.19 – 1.10 (m, 15H), 0.88 (t, *J* = 7.4 Hz, 3H) ppm; **^{13}C NMR** (126 MHz, CD_3OD) δ 178.4, 170.0, 167.3, 167.2, 143.6, 141.5, 137.1, 137.0, 136.2, 134.7, 129.0, 126.9, 125.3, 124.6, 106.3, 97.1, 94.3, 78.6, 76.0, 74.5, 73.6, 73.2, 70.55, 68.3, 45.8, 42.6, 37.3, 35.4, 28.8, 28.7, 28.4, 26.9, 26.5, 24.6, 20.2, 19.5, 19.1, 18.7, 17.5, 15.4, 14.0, 11.3 ppm; **HRMS** ESI(+), (MeOH) calculated for $C_{42}H_{62}O_{14}Na$ $[M+Na]^+$: 813.40375, found: 813.40318.

Baylis-Hillman type mechanism – Control experiment

A flame-dried microwave tube was charged with fidaxomicin (**1**, 5 mg, 4.72 µmol, 1.0 eq.), Meldrum's acid (2.72 mg, 0.018 mmol, 4.0 eq.) and PPh_3 (0.62 mg, 2.36 µmol, 50 mol% or 2.5 mg, 9.44 µmol, 200 mol%). The tube was sealed and evacuated and flushed with argon several times. Then, the solids were dissolved in dry toluene (100 □L, degassed by freeze-pump-thaw (3x)) and the mixture was stirred for 3 h at 70 °C. After evaporation of the solvent, the crude mixture was analyzed by UPLC/MS analysis and ^{1}H NMR. In both cases, mainly starting material was recovered with slight degradation.

(*E*)-13, (*Z*)-13 and 12

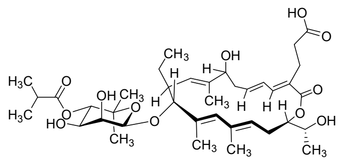

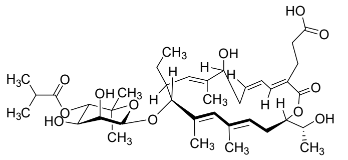

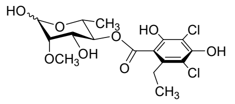

A flame-dried microwave tube was charged with fidaxomicin (**1**, 500 mg, 0.473 mmol, 1.0 eq.), Meldrum's acid (136 mg, 0.946 mmol, 2.0 eq.) and $Pd(PPh_3)_4$ (137 mg, 0.118 mmol, 25 mol%). The tube was sealed and evacuated and flushed with argon several times. Then, the solids were dissolved in dry toluene (4.5 mL, degassed by freeze-pump-thaw (3x)) and the mixture was stirred for 3 h at 70 °C. The solvent was evaporated and the residue was dissolved in DMF/H_2O (9:1, 4.5 mL) and heated to 100 °C for 5 h. The reaction was filtered over Celite[a] and the solvent was evaporated. The crude mixture was purified by RP-HPLC [Gemini NX, C18, 5 µ, 110 Å, 250 mm × 21.2 mm, solvent A: H_2O + 0.1% HCOOH, solvent B: MeCN + 0.1% HCOOH, 20 mL/min; LC time program (min –% B): 0.0 min – 35%, 15 min – 35%, 37.5 min – 40%, 38 min – 100%] to afford, after lyophilization, sugar **12** (α/β = 4:1, t_R = 9 min, 137 mg, 0.333 mmol, 70%) carboxylic acid (*E*)-**13** (t_R = 22.5 min, 122 mg, 0.173 mmol, 37%) and carboxylic acid (*Z*)-**13** (t_R = 26 min, 42.9 mg, 60.6 µmol, 13%)

(*E*)-13

TLC (pentane:acetone, 1:1 v/v) = 0.20; **Specific Rotation** $[\alpha]_D^{22°C}$ = +33.75 (c = 0.40, MeOH); **FT-IR** $\tilde{\nu}$ (film) 3417, 2975, 2934, 2875, 1700, 1641, 1602, 1447, 1386, 1370, 1353, 1295, 1250, 1207, 1152, 1082, 1033, 976, 951, 898, 798, 764 cm^{-1}; **^{1}H NMR** (400 MHz, CD_3OD) δ 7.06 (d, *J* = 11.4 Hz, 1H), 6.46 (dd, *J* = 14.6, 11.4 Hz, 1H), 5.85 (ddd, *J* = 14.6, 9.6, 4.4, 1H), 5.82 (s, 1H), 5.55 (t, *J* = 8.2 Hz, 1H), 5.12 (dt, *J* = 10.5, 1.6 Hz, 1H), 5.02 (d, *J* = 10.2 Hz, 1H), 4.71 (d, *J* = 1.1 Hz, 1H), 4.67 (td, *J* = 6.2, 4.5 Hz, 1H), 4.22 (m, 1H), 4.02 (quint, *J* = 6.2 Hz, 1H), 3.92 (d, *J* = 3.1 Hz, 1H), 3.76 – 3.66 (m, 2H), 2.75 – 2.54 (m, 6H),2.49 (ddd, *J* = 15.0, 9.6, 4.4 Hz, 1H), 2.45 – 2.33 (m, 3H), 2.04 – 1.95 (m, 1H), 1.81 (d, *J* = 1.3 Hz, 3H), 1.74 (d, *J* = 1.3 Hz, 3H), 1.66 (d, *J* = 1.2 Hz, 3H), 1.34 – 1.23 (m, 1H), 1.19 – 1.10 (m, 15H), 0.88 (t, *J* = 7.4 Hz, 3H); **^{13}C NMR** (126 MHz, CD_3OD) δ 178.4, 177.0, 169.8, 142.7, 141.1, 137.1, 136.9, 136.2, 134.7, 128.84, 128.80, 126.9, 124.5, 97.1, 94.3, 78.5, 76.0, 74.5, 73.6, 73.2, 70.6, 68.2, 42.5, 37.3, 35.4, 34.7, 28.7, 28.2, 26.9, 23.8, 20.2, 19.5, 19.1, 18.7, 17.5, 15.4, 13.9, 11.3 ppm; **HRMS** ESI(+), (MeOH) calculated for $C_{38}H_{58}O_{12}Na$ $[M+Na]^+$: 729.38150, found: 729.38205.

(*Z*)-13

TLC (pentane:acetone, 1:1) = 0.20; **Specific Rotation** $[\alpha]_D^{25\,°C}$ = +57.57 (c = 1.00, MeOH); **FT-IR** $\tilde{\nu}$ (film) 3453, 2976, 2934, 2875, 1704, 1633, 1593, 1385, 1260, 1203, 1149, 1078, 1034, 1035, 878, 796 cm^{-1}; **^{1}H NMR** (500 MHz, CD_3OD) δ 7.38 (d, *J* = 11.8 Hz, 1H), 6.48 (t, *J* = 11.3, 1H), 6.01 (dt, *J* = 9.4, 5.6 Hz, 1H), 5.74 (s, 1H), 5.55 (t, *J* = 7.7 Hz, 1H), 5.14 (dt, *J* = 10.9, 1.4 Hz, 1H), 5.02 (d, *J* = 10.2 Hz, 1H), 4.84 – 4.78 (m, 1H), 4.70 (d, *J* = 1.2 Hz, 1H), 4.04 – 3.95 (m, 2H), 3.93 (d, *J* = 3.0 Hz, 1H), 3.73 (dd, *J* = 10.2, 3.2 Hz, 1H), 3.69 (d, *J* = 9.4 Hz, 1H), 2.74 – 2.64 (m, 3H), 2.63 – 2.54 (m, 2H), 2.49 (m, 1H), 2.44 – 2.33 (m, 3H), 2.25–2.14 (m, 1H), 2.07 – 1.97 (m, 1H), 1.62 (s, 3H), 1.61 (s, 3H), 1.59 (s, 3H), 1.29 – 1.22 (m, 1H), 1.19 – 1.10 (m, 15H), 0.89 (t, *J* = 7.4 Hz, 3H); **^{13}C NMR** (126 MHz, CD_3OD) δ 178.4, 177.4, 168.9, 140.0, 139.9, 136.9, 136.5, 135.4, 133.5, 131.4, 125.5, 125.3, 123.9, 97.4, 94.5, 78.6, 75.9, 74.9, 74.6, 73.0, 70.5, 67.9, 42.8, 37.2, 35.4, 35.0, 28.8, 28.7, 26.8, 23.7, 20.4, 19.5, 19.1, 18.8, 18.0, 16.0, 14.1, 11.8 ppm; **HRMS** ESI(+), (MeOH) calculated for $C_{38}H_{58}O_{12}Na$ $[M+Na]^+$: 729.38150, found: 729.38250.

12

TLC (acetone:pentane, 3:2 v/v) = 0.43; **FT-IR** (film) $\tilde{\nu}$ =3401, 2986, 2939, 1655, 1560, 1456, 1411, 1390, 1374, 1314, 1290, 1242, 1195, 1145, 1128, 1100, 1073, 1039, 1018, 996, 910, 834, 799, 759, 732, 710, 688, 648, 596, 519 cm^{-1}; α: **^{1}H NMR** (500 MHz, $CDCl_3$) δ 5.38 (d, *J* = 1.5 Hz, 1H), 5.20 (t, *J* = 9.9 Hz, 1H), 4.16–4.03 (m, 2H), 3.61 (dd, *J* = 3.7, 1.5 Hz, 1H); 3.53 (s, 3H), 3.17–2.97 (m, 2H), 1.27–1.18 (m, 6H) ppm; **^{13}C NMR** (126 MHz, $CDCl_3$) δ 170.26, 157.7, 152.6, 143.3, 113.8, 107.4, 107.1, 90.8, 80.5, 76.7, 69.1, 65.9, 59.0, 26.2, 17.8, 14.0 ppm; β: **^{1}H NMR** (500 MHz, $CDCl_3$) δ 5.15 (t, *J* = 9.8 Hz, 1H), 4.85 (d, *J* = 1.3 Hz, 1H), 3.80 (dd, *J* = 9.9, 3.5 Hz, 1H), 3.74 (s, 1H), 3.67 (dd, *J* = 3.6, 1.3 Hz, 1H); 3.58–3.55 (m, 1H), 3.17–2.97 (m, 2H), 1.31 (d, *J* = 6.2 Hz, 1H), 1.27–1.18 (m, 3H) ppm; **^{13}C NMR** (126 MHz, $CDCl_3$) δ 170.34, 157.6, 152.7, 143.1, 113.9, 107.3, 107.2, 94.7, 81.4, 76.2, 72.8, 69.9, 63.2, 26.2, 17.8, 14.0 ppm; **HRMS** ESI(–) (MeOH) calculated for $C_{16}H_{19}Cl_2O_8$ $[M–H]^-$: 409.04625, found: 409.04625.

S1

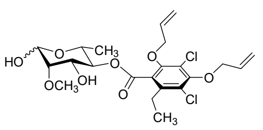

The Rhamnosyl-resorcylate moiety (**12**, 100 mg, 0.243 mmol, 1.0 eq.) and K_2CO_3 (134 mg, 0.972 mmol, 4.0 eq.) were dissolved in dry DMF (4.8 mL) and allyl bromide (63 µL, 0.73 mmol, 3.0 eq.) was added dropwise. The mixture was warmed to 45 °C and it was stirred for 3 h. After complete conversion as indicated by TLC (EtOAc/pentane 1:1), the reaction mixture was diluted with EtOAc (10 mL) and washed with aq. sat. NH_4Cl (3 x 10 mL). The combined organic layers were dried over $MgSO_4$ and the solvent was evaporated under reduced pressure. The crude was purified by silica-gel column chromatography (EtOAc/penante 1:2) to afford the desired allyl-protected rhamnosyl-resorcylate **S1** (a/b = 6:1, 112 mg, 0.229 mmol, 94%) as a colorless oil.

TLC (EtOAc:pentane, 1:1 v/v) = 0.60; **FT-IR** (film) $\tilde{\nu}$ =3452, 2939, 1734, 1651, 1568, 1456, 1403, 1351, 1315, 1252, 1193, 1103, 1065, 1042, 1019, 933, 799, 749, 601 cm^{-1}; α**:** **^{1}H NMR** (400 MHz, $CDCl_3$) δ 6.19–5.95 (m, 2H), 5.46–5.18 (m, 4H), 5.33 (s, 1H), 5.09 (t, *J* = 9.8 Hz, 1H), 4.62–4.44 (m, 4H), 4.08–3.92 (m, 2H), 3.59–3.57 (m, 1H), 3.53 (s, 3H), 3.1 (d, *J* = 3.5 Hz, 1H), 2.89–2.70 (m, 2H), 2.54 (d, *J* = 10.7 Hz, 1H), 1.30 (d, *J* = 6.3 Hz, 3H), 1.19 (t, *J* = 7.5 Hz, 3H) ppm; **^{13}C NMR** (126 MHz, $CDCl_3$) δ 166.6, 153.1, 151.0, 139.2, 132.9, 132.8, 127.3, 125.6, 121.6, 119.1, 119.0, 91.0, 80.7, 76.6, 75.9, 74.4, 69.2, 66.0, 59.1, 25.3, 17.7, 14.1 ppm; β**:** **^{1}H NMR** (400 MHz, $CDCl_3$) 6.19–5.95 (m, 2H), 5.46–5.18 (m, 4H), 5.05 (t, *J* = 9.8 Hz, 1H), 4.76 (d, *J* = 1.4 Hz, 1H), 4.62–4.44 (m, 4H), 3.78–3.75 (m, 1H), 3.74 (s, 3H), 3.63 (dd, *J* = 3.4, 1.3 Hz, 1H), 3.51–3.44 (m, 1H), 2.89–2.70 (m, 2H), 1.35 (d, *J* = 6.2 Hz, 3H), 1.19 (t, *J* = 7.5 Hz, 3H) ppm; **^{13}C NMR** (126 MHz, $CDCl_3$) δ166.7, 153.3, 150.9, 138.9, 132.8, 132.5, 127.0, 125.7, 121.6, 119.5, 119.2, 94.4, 81.3, 76.3, 76.0, 73.2, 70.0, 65.9, 62.8, 25.3, 17.7, 14.1 ppm; **HRMS** ESI(–) (MeOH) calculated for $C_{23}H_{29}Cl_2O_{10}$ [M+HCOO]$^-$: 535.11433, found: 535.11416.

14

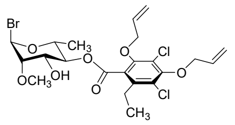

Allyl-protected sugar **S2** (50.0 mg, 0.102 mmol, 1.0 eq.) was dissolved in dry CH_2Cl_2 (500 µL) and cooled to 0 °C. Then, HBr (33 wt% in AcOH, 51 µL, 0.31 mmol, 3.0 eq.) was added dropwise and the mixture was allowed to warm to room temperature over 1 h and stirred for an additional 1 h at room temperature. The reaction was monitored by TLC (EtOAc/pentane 1:2). Upon completion, the reaction was poured onto a water-ice mixture (5 mL) and stirred for 15 min. Next, CH_2Cl_2 (5 mL) was added and the layers were separated. The organic layer was washed with aq. Sat. $NaHCO_3$ (5 mL) and brine (5 mL). The organic layer was dried over $MgSO_4$, filtered and the solvent was evaporated. The bromo-sugar **14** (46.3 mg, 83.5 mmol, 82%) was obtained as slightly yellow oil and was used without further purification.

TLC (EtOAc:pentane, 1:2 v/v) = 0.79; **FT-IR** (film) $\tilde{\nu}$ = 3531, 2981, 2938, 1738, 1568, 1458, 1403, 1371, 1314, 1277, 1247, 1126, 1116, 1076, 1046, 1001, 974, 932, 842, 792, 767, 748, 680 cm^{-1}; **^{1}H NMR** (500 MHz, $CDCl_3$) δ 6.56 (m, 2H), 6.15 (ddt, *J* = 16.5, 10.3, 5.9 Hz, 1H), 6.04 (ddt, *J* = 16.5, 10.3, 5.9 Hz, 1H), 5.45 (dd, *J* = 17.1, 1.3 Hz, 1H), 5.37 (dd, *J* = 17.1, 1.3 Hz, 1H), 5.31 (dd, *J* = 10.2, 1.3 Hz, 1H), 5.26 (dd, *J* = 10.2, 1.3 Hz, 1H), 5.16 (t, J = 9.9 Hz, 1H), 4.62 – 4.45 (m, 4H), 4.31 (dd, *J* = 9.9 Hz, 3.6 Hz, 1H), 4.01 (dq, *J* = 9.9, 6.2 Hz, 1H), 3.81 (dd, *J* = 3.6, 1.4 Hz, 1H), 3.55 (s, 3H), 2.81 (m, 2H), 1.35 (d, *J* = 6.2 Hz, 3H), 1.21 (t, *J* = 7.5 Hz, 3H) ppm; **^{13}C NMR** (126 MHz, $CDCl_3$) δ 166.4, 153.4, 151.1, 139.2, 132.90, 132.85, 127.0, 125.7, 121.7, 119.1, 119.0, 85.9, 83.7, 75.9, 75.6, 74.5, 70.9, 68.5, 59.1, 25.3, 17.2, 14.2 ppm; **HRMS** ESI(+) (MeOH) calculated for $C_{22}H_{27}BrCl_2O_7Na$ $[M+Na]^+$: 575.02094, found: 575.02045.

15

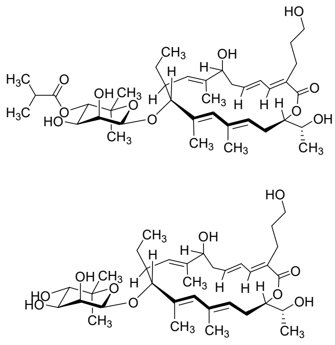

In a flame dried flask under an atmosphere of argon, carboxylic acid **(*E*)-13** (117 mg, 0.166 mmol, 1.0 eq.) was dissolved in dry THF (500 µL) and *N*-methylmorpholine (36 µL, 0.33 mmol, 2.0 eq.) was added. The mixture was cooled to 0 °C and isobutyl chloroformate (43 µL, 0.33 mmol, 2.0 eq.) was added. The reaction mixture was to stirred for 1 h at 0 °C before it was filtered over a plug of Celite[a] into a solution of $NaBH_4$ (7.5 mg, 0.198 mmol, 1.2 eq.) in H_2O (400 µL) at 0 °C. It was stirred for 1 h at 0 °C and was then allowed warm to room temperature over 1 h. H_2O (2 mL) was added and the reaction was extracted with EtOAc (3 x 2 mL). The crude product was purified by silica-gel column chromatography (MeOH/CH_2Cl_2 1:19 to 1:9) to obtain alcohol **15** (68.3 mg, 98.6 µmol, 60%) as a colorless solid. Hydrolyzed **S2** (7.0 mg, 11 µmol, 20%) was obtained as a by-product.

15

TLC (MeOH:CH_2Cl_2, 1:9 v/v) = 0.44; **Specific Rotation** $[\alpha]_D^{28\,°C}$ = +68.17 (c = 0.35, MeOH); **FT-IR** $\tilde{\nu}$ (film) 3420, 2974, 2932, 2875, 1732, 1696, 1641, 1447, 1385, 1250, 1198, 1151, 1072, 1033, 898, 796, 511 cm^{-1}; **^{1}H NMR** (500 MHz, CD_3OD) δ 7.06 (d, *J* = 11.3 Hz, 1H), 6.49 – 6.40 (m, 1H), 5.87 – 5.77 (m, 2H), 5.56 (t, *J* = 8.3 Hz, 1H), 5.13 (dt, *J* = 10.5, 1.6 Hz, 1H), 5.02 (d, *J* = 10.2 Hz, 1H), 4.71 (d, *J* = 1.2 Hz, 1H), 4.67 (q, *J* = 5.5 Hz, 1H), 4.21 (m, 1H), 4.00 (quint, *J* = 6.4 Hz, 1H), 3.92 (d, *J* = 2.9 Hz, 1H), 3.78 – 3.66 (m, 2H), 3.53 (t, *J* = 6.4 Hz, 2H), 2.75 – 2.63 (m, 3H), 2.59 (sept, *J* = 6.9 Hz, 1H), 2.50−2.43 (m, 4H), 2.06 – 1.97 (m, 1H), 1.81 (d, *J* = 1.3 Hz, 3H), 1.73 (d, *J* = 1.3 Hz, 3H), 1.67 – 1.65 (m, 3H), 1.65 – 1.58 (m, 2H), 1.33 – 1.24 (m, 1H), 1.20 – 1.09 (m, 15H), 0.88 (t, *J* = 7.4 Hz, 3H) ppm; **^{13}C NMR** (126 MHz, CD_3OD) δ 178.4, 170.1, 142.4, 140.8, 137.00, 136.95, 136.2, 134.7, 129.7, 128.8, 126.7, 124.5, 97.2, 94.3, 78.5, 75.9, 74.5, 73.6, 73.2, 70.5, 68.2, 62.3, 42.5, 37.2, 35.4, 33.2, 28.7, 28.3, 26.9, 24.0, 20.3, 19.5, 19.1, 18.7, 17.5, 15.4, 13.9, 11.3 ppm; **HRMS** ESI(+) (MeOH) calculated for $C_{38}H_{60}O_{11}Na$ $[M+Na]^+$: 715.40250, found: 715.40278.

S2

TLC (MeOH:CH_2Cl_2, 1:9 v/v) = 0.15; **Specific Rotation** $[\alpha]_D^{28\,°C}$ = +71.2 (c = 0.49, MeOH); **FT-IR** $\tilde{\nu}$ (film) 3382, 2972, 2931, 2874, 1683, 1640, 1381, 1249, 1209, 1160, 1070, 1029, 977, 895, 787 cm^{-1}; **^{1}H NMR** (500 MHz, CD_3OD) δ 7.07 (d, J = 11.4 Hz, 1H), 6.48 – 6.40 (m, 1H), 5.86 – 5.78 (m, 2H), 5.56 (t, J = 8.2 Hz, 1H), 5.13 (dd, J = 10.4, 1.8 Hz, 1H), 4.68 (q, J = 5.6 Hz, 1H), 4.65 (s, 1H), 4.23 – 4.19 (m, 1H), 4.01 (quint, J = 6.4 Hz, 1H), 3.88 (d, J = 3.2 Hz, 1H), 3.69 (d, J = 9.7 Hz, 1H), 3.56 – 3.50 (m, 3H), 3.47 (d, J = 10.0 Hz, 1H), 2.75 – 2.62 (m, 4H), 2.50 – 2.34 (m, 4H), 2.06 – 1.95 (m, 1H), 1.81 (s, 3H), 1.74 (s, 3H), 1.65 (s, 3H), 1.64 – 1.58 (m, 2H), 1.32 – 1.26 (m, 1H), 1.24 (s, 3H), 1.17 (d, J = 6.3 Hz, 3H), 1.07 (s, 3H), 0.88 (t, J = 7.4 Hz, 3H) ppm; **^{13}C NMR** (126 MHz, CD_3OD) δ 170.1, 142.4, 140.7, 137.0, 136.9, 136.1, 134.7, 129.7, 128.9, 126.7, 124.6, 97.0, 94.0, 78.5, 75.9, 74.8, 73.6, 73.1, 72.2, 68.3, 62.3, 42.6, 37.2, 33.2, 30.7, 28.8, 28.4, 26.9, 23.4, 20.2, 17.8, 17.5, 15.4, 13.9, 11.3 ppm; **HRMS** ESI(+) (MeOH) calculated for $C_{34}H_{54}O_{10}Na$ $[M+Na]^+$: 645.36092, found: 645.36072.

S3

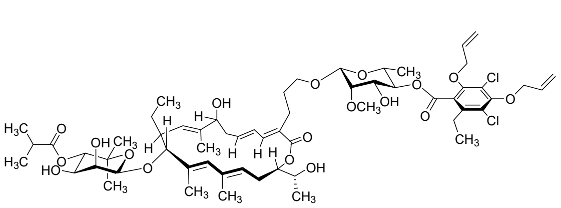

In a flame dried flask under an atmosphere of argon, to a solution alcohol **15** (63.7 mg, 91.9 µmol, 1.0 eq.) and bromide **14** (56.5 mg, 0.102 mmol, 1.1 eq.) in dry CH_2Cl_2 (8.0 mL), molecular sieves (4Å, 800 mg) was added and the suspension was stirred for 30 min at room temperature. Then, Ag_2CO_3 (253 mg, 0.919 mmol, 10 eq.) was added and the reaction mixture was stirred for 24 h at room temperature. It was filtered over Celiteâ and the solvent was evaporated. The crude product was purified by RP-HPLC [Gemini NX, C18, 5 µ, 110 Å, 250 mm × 21.2 mm, solvent A: H_2O + 0.1% HCOOH, solvent B: MeCN + 0.1% HCOOH, 20 mL/min; LC time program (min –% B): 0.0 min – 70%, 15 min – 70%, 45 min – 90%, 45.20 min –100%] to afford, after lyophilization, allyl-protected C2-elongated fidaxomicin **S3** (t_R = 25.5 min, 15.0 mg, 12.8 µmol, 14%) as a colorless solid.

TLC (MeOH:CH_2Cl_2, 1:19 v/v) = 0.79; **Specific Rotation** $[\alpha]_D^{24\,°C}$ = +6.16 (c = 0.75, MeOH); **FT-IR** (film) $\tilde{\nu}$ = 3464, 2975, 2931, 2876, 1736, 1699, 1641, 1568, 1455, 1402, 1384, 1369, 1352, 1316, 1249, 1199, 1135, 1070, 1025, 995, 975, 932, 901, 791, 775, 750, 713 cm^{-1}; **^{1}H NMR** (500 MHz, acetone-d_6) δ 7.10 (d, *J* = 11.4 Hz, 1H), 6.51–6.44 (m, 1H), 6.17 (ddt, *J* = 17.2, 10.4, 5.8 Hz, 1H), 6.08 (ddt, *J* = 17.1, 10.4, 5.8 Hz, 1H), 5.90–5.82 (m, 2H), 5.61 (t, *J* = 8.4 Hz, 1H), 5.49–5.37 (m, 2H), 5.30–5.23 (m, 2H), 5.21 (dt, *J* = 10.5, 1.5 Hz, 1H), 5.03 (t, *J* = 9.7 Hz, 1H), 4.99 (d, *J* = 10.1 Hz, 1H), 4.77 (d, *J* = 1.3 Hz, 1H), 4.71–4.66 (m, 1H), 4.64–4.57 (m, 4H), 4.64–4.57 (m, 1H), 4.29–4.21 (m, 1H), 4.07–4.00 (m, 2H), 3.97–3.94 (m, 1H), 3.90−3.85 (m, 2H), 3.81 (d, *J* = 3.6 Hz, 1H), 3.79−3.69 (m, 3H), 3.68 (d, *J* = 4.1 Hz, 1H), 3.65−3.63 (m, 1H), 3.64 (s, 3H), 3.52−3.46 (m, 1H), 3.46−3.40 (m, 1H), 2.92–2.71 (m, 2H), 2.71–2.60 (m, 3H), 2.56 (sept, *J* = 7.0 Hz, 1H), 2.51−2.35 (m, 4H), 1.97–1.91 (m, 1H), 1.81 (d, *J* = 1.3 Hz, 3H), 1.79–1.67 (m, 2H), 1.73 (d, *J* = 1.4 Hz, 3H), 1.65 (d, *J* = 1.3 Hz, 3H), 1.29 (d, *J* = 6.1 Hz, 3H), 1.26–1.18 (m, 1H), 1.18–1.13 (m, 15H), 1.13 (s, 3H), 0.81 (t, *J* = 7.4 Hz, 3H) ppm; **^{13}C NMR** (126 MHz acetone-d_6) δ 176.8, 168.7, 166.5, 153.9, 151.9, 141.6, 140.6, 140.0, 136.8, 136.2, 136.0, 134.13, 134.07, 133.9, 129.3, 128.7, 128.5, 126.6, 126.0, 124.0, 122.3, 118.8, 118.7, 102.6, 96.7, 93.3, 82.0, 78.2, 77.6, 76.4, 75.7, 75.0, 73.7, 73.0, 72.8, 72.3, 70.6, 70.2, 69.4, 67.7, 61.9, 42.1, 37.3, 34.8, 30.1, 28.7, 28.2, 26.5, 25.6, 24.1, 20.6, 19.4, 19.2, 18.6, 18.3, 17.4, 15.2, 14.4, 13.8, 11.2 ppm; **HRMS** ESI(+) (MeOH) calculated for $C_{60}H_{86}Cl_2O_{18}Na$ $[M+Na]^+$: 1187.50834, found: 1187.50826

17

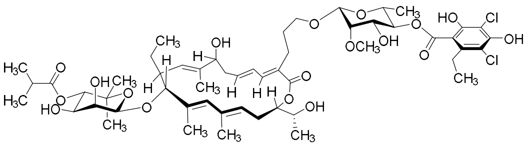

In a flame-dried flask under argon atmosphere, to a solution of allyl-protected C2-elongated fidaxomicin (**S3**, 15.0 mg, 12.9 µmol, 1.0 eq.) in dry THF (1.2 mL) was added morpholine (1 µL, 13 µmol, 1.0 eq.). After cooling the mixture to 0 °C, $Pd(PPh_3)_4$ (1.5 mg, 1.3 µmol, 10 mol%) was added and it was stirred for 10 min. Then, the reaction mixture was diluted with EtOAc (2 mL) and washed with sat. aq. NH_4Cl (2 mL). The phases were separated and the aqueous layer was extracted with EtOAc (3 x 3 mL). The combined organic layers were dried over $MgSO_4$, filtered and the solvent was evaporated under reduced pressure. The crude residue was further purified by RP-HPLC [Gemini NX, C18, 5 µ, 110 Å, 250 mm × 21.2 mm, solvent A: H_2O + 0.1% HCOOH, solvent B: MeCN + 0.1% HCOOH, 20 mL/min; LC time program (min –% B): 0.0 min – 60%, 5.0 min – 60%, 30.0 min – 70%, 30.5 min –100%] to afford, after lyophilization, C2-elongated fidaxomicin **17** (t_R = 13 min, 9.1 mg, 8.4 µmol, 65%) as a colorless solid.

TLC (MeOH:CH_2Cl_2, 1:19 v/v) = 0.16; **Specific Rotation** $[\alpha]_D^{24\,°C}$ = +8.24 (c = 0.46, MeOH); **FT-IR** (film) $\tilde{\nu}$ = 3447, 2976, 2935, 2876, 1734, 1697, 1590, 1406, 1384, 1369, 1312, 1247, 1198, 1146, 1111, 1070, 1024, 975, 901, 873, 799, 760, 738, 720, 695, 582, 504, 477 cm^{-1}; **^{1}H NMR** (500 MHz, acetone-d_6) δ 7.10 (d, *J* = 11.4 Hz, 1H), 6.54−6.41 (m, 1H), 5.91−5.82 (m, 2H), 5.61 (t, *J* = 8.3 Hz, 1H), 5.21 (dt, *J* = 10.6, 1.5 Hz, 1H), 5.10 (t, *J* = 9.7 Hz, 1H), 4.99 (d, *J* = 10.1 Hz, 1H), 4.77 (d, *J* = 1.2 Hz, 1H), 4.71−4.66 (m, 1H), 4.65 (d, *J* = 0.8 Hz, 1H), 4.28−4.23 (m, 1H), 4.07−3.99 (m, 1H), 3.95 (d, *J* = 3.3 Hz, 1H), 3.91−3.79 (m, 3H), 3.76−3.65 (m, 3H), 3.63 (s, 3H), 3.62−3.56 (m, 1H), 3.50−3.43 (m, 1H), 3.04−2.96 (m, 2H), 2.79−2.61 (m, 3H), 2.56 (sept, *J* = 6.7 Hz, 1H), 2.51−2.36 (m, 4H), 1.99−1.90 (m, 1H), 1.81 (d, *J* = 1.3 Hz, 3H), 1.79−1.67 (m, 2H), 1.73 (d, *J* = 1.4 Hz, 3H), 1.65 (s, 3H), 1.27 (d, *J* = 6.1 Hz, 3H), 1.26−1.22 (m, 1H), 1.21 (t, *J* = 7.3 Hz, 3H), 1.17−1.12 (m, 12H), 1.09 (s, 3H), 0.83 (t, *J* = 7.4 Hz, 3H) ppm; **^{13}C NMR** (126 MHz, acetone-d_6) δ 176.8, 169.4, 168.7, 155.7, 153.8, 142.6, 141.6, 140.7, 136.8, 136.2, 136.0, 133.9, 129.3, 128.4, 126.5, 124.0, 114.5, 110.8, 108.2, 102.6, 96.7, 93.3, 81.8, 78.2, 77.7, 75.7, 73.7, 72.9, 72.8, 72.2, 70.5, 70.1, 69.4, 67.6, 61.8, 42.1, 37.3, 34.8, 30.1, 28.7, 28.2, 26.5, 26.2, 24.0, 20.6, 19.4, 19.2, 18.6, 18.1, 17.4, 15.2, 14.4, 13.8, 11.2 ppm; **HRMS** ESI(+) (MeOH) calculated for $C_{60}H_{86}Cl_2O_{18}Na$ $[M+Na]^+$: 1107.44574, found: 1107.44501.

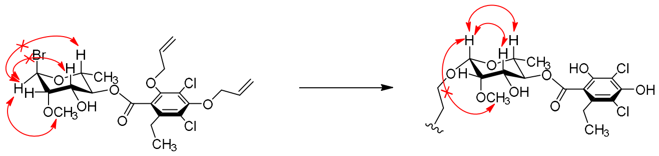

Supplementary Figure 1. Observed NOESY correlations in glycosyl donor **14** and C2-elongated **17**.

16

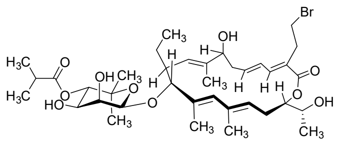

In a flame-dried flask under argon atmosphere, carboxylic acid **(*E*)-13** (122 mg, 0.173 mmol, 1.0 eq.) and HOTT (95.8 mg, 0.258 mmol, 1.5 eq.) were dissolved in dry CH_2Cl_2 (1.8 mL). Next, DIPEA (71 µL, 0.43 mmol, 2.5 eq.) was added and flask was covered in aluminum foil. The reaction mixture was stirred at room temperature for 1 h, the aluminum foil was removed and CCl_3Br (1.2 mL) was added. The reaction was allowed to stir for 15 h at room temperature, the solvent was evaporated and the residue was purified by silica-gel column chromatography (EtOAc/pentane 3:2). The bromide **16** (79.1 mg, 0.107 mmol, 62%) was obtained as a slightly yellow solid.

TLC (EtOAc:pentane, 1:1 v/v) = 0.13; **Specific Rotation** $[\alpha]_D^{26\,°C}$ = +17.7 (c = 0.38, MeOH); **FT-IR** (film) $\tilde{\nu}$ = 3441, 2974, 2933, 2873, 1735, 1698, 1640, 1446, 1385, 1297, 1249, 1198, 1153, 1072, 1033, 898, 844, 798, 504 cm^{-1}; **^{1}H NMR** (500 MHz, acetone-d_6) δ 7.18 (d, *J* = 11.4 Hz, 1H), 6.56−6.38 (m, 1H), 5.92 (ddd, *J* = 14.6, 9.7, 4.5 Hz, 1H), 5.82 (s, 1H), 5.61 (t, *J* = 8.2 Hz, 1H), 5.21 (dt, *J* = 10.7, 1.6 Hz, 1H), 4.99 (d, *J* = 10.1 Hz, 1H), 4.78 (d, *J* = 1.2 Hz, 1H), 4.73−4.65 (m, 1H), 4.33−4.21 (m, 1H), 4.09−3.99 (m, 2H), 3.98−3.94 (m, 1H), 3.85 (d, *J* = 3.6 Hz, 1H), 3.76−3.70 (m, 2H), 3.68 (d, *J* = 4.2 Hz, 1H), 3.54−3.44 (m, 2H), 3.32 (d, *J* = 9.2 Hz, 1H), 2.99−2.80 (m, 2H), 2.80−2.60 (m, 3H), 2.56 (sept, *J* = 7.0 Hz, 1H), 2.49 (ddd, *J* = 14.8, 9.7, 4.2 Hz, 1H), 2.42 (ddd, *J* = 14.0, 9.1, 4.4 Hz, 1H), 1.99−1.89 (m, 1H), 1.80 (d, *J* = 1.3 Hz, 3H), 1.71 (d, *J* = 1.4 Hz, 3H), 1.65 (s, 3H), 1.29−1.22 (m, 1H), 1.19−1.11 (m, 12H), 1.09 (s, 3H), 0.83 (t, *J* = 7.4 Hz, 3H) ppm; **^{13}C NMR** (126 MHz, acetone-d_6) δ 176.8, 168.2, 143.7, 142.4, 136.8, 136.2, 136.0, 133.9, 128.1, 126.31, 126.25, 123.9, 96.7, 93.3, 78.3, 75.7, 73.8, 72.89, 72.83, 70.1, 67.6, 42.0, 37.2, 34.7, 32.1, 31.2, 28.7, 28.2, 26.5, 20.7, 19.4, 19.2, 18.6, 17.4, 15.2, 13.8, 11.2 ppm; **HRMS** ESI(+) (MeOH) calculated for $C_{37}H_{57}BrO_{10}Na$ $[M+Na]^+$: 763.30273, found: 763.30285.

16.1

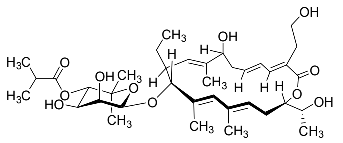

Bromide **16** (52.9 mg, 71.3 μmol, 1.0 eq.) was dissolved in MeCN/H_2O (1:1, 1.4 mL) and AgF (18.1 mg, 0.143 mmol, 2.0 eq.) was added. The reaction mixture was stirred at 50 °C for 14 h before it was filtered over Celite[a] and the solvent was removed under reduced pressure. The residue was redissolved in MeCN (2 mL) and filtered again. The solvent was evaporated and the residue was purified by RP-HPLC [Gemini NX, C18, 5 μ, 110 Å, 250 mm × 21.2 mm, solvent A: H_2O + 0.1% HCOOH, solvent B: MeCN + 0.1% HCOOH, 20 mL/min; LC time program (min – % B): 0.0 min – 35%, 10.0 min – 35%, 45.0 min – 45%, 45.2 min – 100%] to afford, after lyophilization, alcohol **16.1** (t_R = 18 min, 10.7 mg, 15.7 μmol, 44%) as a colorless solid.

TLC (MeOH:CH_2Cl_2, 1:19 v/v) = 0.12; **Specific Rotation** $[\alpha]_D^{26\,°C}$ = +36.0 (c = 0.36, MeOH); **FT-IR** (film) $\tilde{\nu}$ = 3423, 2975, 2933, 2876, 1734, 1695, 1641, 1443, 1386, 1370, 1296, 1252, 1202, 1151, 1085, 1035, 975, 951, 899, 797 cm^{-1}; **^{1}H NMR** (500 MHz, acetone-d_6) 7.11 (d, *J* = 11.4 Hz, 1H), 6.46 (dd, *J* = 15.0, 11.4 Hz, 1H), 5.88–5.80 (m, 2H), 5.59 (t, *J* = 8.3 Hz, 1H), 5.19 (d, *J* = 10.5 Hz, 1H), 4.99 (d, *J* = 10.1 Hz, 1H), 4.77 (s, 1H), 4.67 (q, *J* = 5.5 Hz, 1H), 4.25–4.22 (m, 1H), 4.02 (quint, *J* = 6.3 Hz, 1H), 3.95 (d, *J* = 3.2 Hz, 1H), 3.74–3.69 (m, 2H), 3.54 (t, *J* = 6.8 Hz, 2H), 2.78–2.43 (m, 7H), 2.39 (ddd, *J* = 13.8, 9.0, 4.4 Hz, 1H), 1.98–1.91 (m, 1H), 1.80 (s, 3H), 1.72 (s, 3H), 1.65 (s, 3H), 1.31–1.20 (m, 1H), 1.16–1.11 (m, 12H), 1.08 (s, 3H), 0.82 (t, *J* = 7.4 Hz, 3H) ppm; **^{13}C NMR** (126 MHz, acetone-d_6) δ 176.8, 169.1, 142.6, 140.7, 136.9, 136.2, 135.9, 133.9, 128.6, 126.7, 126.6, 123.9, 96.7, 93.3, 78.2, 75.7, 73.7, 72.9, 72.8, 70.2, 67.6, 61.9, 42.1, 37.2, 34.8, 31.4, 28.7, 28.2, 26.5, 20.6, 19.4, 19.1, 18.6, 17.4, 15.2, 13.8, 11.2 ppm; **HRMS** ESI(+) (MeCN) calculated for $C_{37}H_{58}O_{11}Na$ $[M+Na]^+$: 701.38713, found: 701.38334.

S4

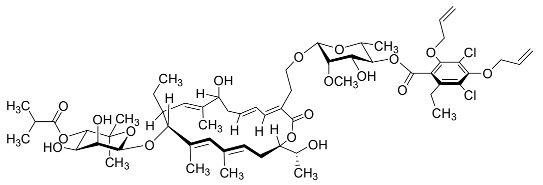

In a flame-dried flask under argon atmosphere, molecular sieves (4Å, 200 mg) were added to a solution of alcohol **16.1** (15.8 mg, 23.3 µmol, 1.0 eq.) and bromide **14** (33.0 mg, 59.5 µmol, 2.5 eq.) in dry CH_2Cl_2 (2.0 mL), and the suspension was stirred for 30 min at room temperature. Next, Ag_2CO_3 (64.2 mg, 0.233 mmol, 10 eq.) was added and the reaction mixture was stirred for 3 h at 40 °C. Subsequently, it was diluted with CH_2Cl_2 (2 mL) and filtered over Celite[a]. The crude product was purified by RP-HPLC [Gemini NX, C18, 5 µ, 110 Å, 250 mm × 21.2 mm, solvent A: H_2O + 0.1% HCOOH, solvent B: MeCN + 0.1% HCOOH, 20 mL/min; LC time program (min –% B): 0.0 min – 68%, 15.0 min – 68%, 45.0 min – 73%, 45.2 min –100%] to afford, after lyophilization, allyl-protected C1-elongated fidaxomicin **S4** (t_R = 25.8 min, 19.7 mg, 4.6 µmol, 20%) as a colorless solid.

TLC (MeOH:CH_2Cl_2, 1:19) = 0.28; **Specific Rotation** $[\alpha]_D^{25\,°C}$ = −7.02 (c = 0.26, MeOH); **FT-IR** (film) $\tilde{\nu}$ = 3462, 2976, 2934, 2877, 1735, 1698, 1642, 1568, 1403, 1385, 1369, 1353, 1318, 1250, 1199, 1135, 1073, 1028, 975, 900 cm^{-1}; **^{1}H NMR** (500 MHz, acetone-d_6) δ 7.17 (d, *J* = 11.4 Hz, 1H), 6.50 (dd, *J* = 14.9, 11.8 Hz, 1H), 6.17 (ddt, *J* = 17.3, 10.4, 5.8 Hz, 1H), 6.08 (ddt, *J* = 17.1, 10.6, 5.8 Hz, 1H), 5.88 (ddd, *J* = 14.7, 9.5, 4.7 Hz, 1H), 5.82 (s, 1H), 5.62 (t, *J* = 8.3 Hz, 1H), 5.49 – 5.36 (m, 2H), 5.31 – 5.23 (m, 2H), 5.21 (dt, *J* = 10.7, 1.8 Hz, 1H), 5.01 (t, *J* = 9.7 Hz, 1H), 4.98 (d, *J* = 10.1 Hz, 1H), 4.77 (d, *J* = 1.3 Hz, 1H), 4.69 (q, *J* = 5.2 Hz, 1H), 4.63 – 4.58 (m, 4H), 4.54 – 4.49 (m, 1H), 4.28 – 4.23 (m, 1H), 4.07 – 4.00 (m, 2H), 3.97 – 3.94 (m, 1H), 3.89 – 3.82 (m, 3H), 3.76 – 3.67 (m, 4H), 3.61 – 3.55 (m, 2H), 3.54 (s, 3H), 3.53 – 3.45 (m, 1H), 3.29 (d, *J* = 9.4 Hz, 1H), 2.90−2.77 (m, 2H), 2.76 – 2.60 (m, 5H), 2.56 (sept, *J* = 7.0 Hz, 1H), 2.48 (ddd, *J* = 14.8, 9.5, 4.1 Hz, 1H), 2.41 (ddd, *J* = 13.9, 9.2, 4.4 Hz, 1H), 1.98 – 1.89 (m, 1H), 1.80 (d, *J* = 1.3 Hz, 3H), 1.72 (s, 3H), 1.65 (s, 3H), 1.29 (d, *J* = 6.1 Hz, 3H), 1.27 – 1.21 (m, 1H), 1.17 – 1.12 (m, 15H), 1.09 (s, 3H), 0.83 (t, *J* = 7.4 Hz, 3H) ppm; ; **^{13}C NMR** (126 MHz, acetone-d_6) δ 176.8, 168.7, 166.5, 153.9, 151.9, 143.0, 141.2, 140.0, 136.9, 136.2, 136.0, 134.13, 134.05, 133.9, 128.7, 128.6, 126.4, 126.1, 126.0, 123.9, 122.3, 118.8, 118.7, 102.5, 96.8, 93.3, 81.8, 78.2, 77.5, 76.4, 75.7, 75.0, 73.8, 72.9, 72.8, 72.3, 70.6, 70.2, 69.1, 67.6, 61.8, 42.0. 37.2, 34.8, 28.7, 28.2, 28.1, 26.5, 25.6, 20.7, 19.4, 19.2, 18.6, 18.3, 17.5, 15.2, 14.4, 13.8, 11.2 ppm; **HRMS** ESI(−) (MeCN) calculated for $C_{60}H_{85}Cl_2O_{20}$ $[M+HCOO]^-$: 1195.50167, found: 1195.50218.

18

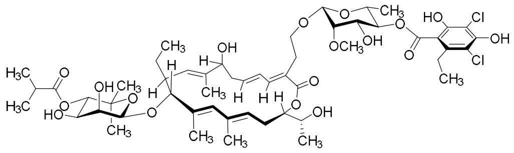

In a flame-dried flask under argon atmosphere, morpholine (0.4 µL, 4.5 µmol, 1.0 eq.) was added to a solution of allyl-protected C1-elongated fidaxomicin **S4** (5.2 mg, 4.5 µmol, 1.0 eq.) in dry THF (450 µL). This mixture was cooled to 0 °C and $Pd(PPh_3)_4$ (0.5 mg, 0.4 µmol, 10 mol%) was added and the reaction was stirred for 20 min. Next, the reaction mixture was diluted with EtOAc (2 mL) and washed with sat. aq. NH_4Cl (2 mL). The phases were separated and the aqueous layer was extracted with EtOAc (3 x 2 mL). The combined organic layers were dried over $MgSO_4$, filtered and the solvent was evaporated under reduced pressure. The crude residue was further purified by RP-HPLC [Gemini NX, C18, 5 µ, 110 Å, 250 mm × 21.2 mm, solvent A: H_2O + 0.1% HCOOH, solvent B: MeCN + 0.1% HCOOH, 20 mL/min; LC time program (min –% B): 0.0 min – 60%, 5.0 min – 60%, 30.0 min – 70%, 30.5 min – 100%] to afford, after lyophilization, C1-elongated fidaxomicin **18** (t_R = 10.5 min, 3.6 mg, 3.4 µmol, 75%) as a colorless solid.

Specific Rotation $[\alpha]_D^{24\,°C}$ = −6.94 (c = 0.72, MeOH); **FT-IR** (film) $\tilde{\nu}$ = 3432, 2976, 2933, 2876, 1733, 1697, 1589, 1385, 1370, 1313, 1250, 1200, 1073, 1028, 899, 799, 761 cm^{-1}; **^{1}H NMR** (500 MHz, acetone-d_6) δ 7.17 (d, *J* = 11.4 Hz, 1H), 6.51 (dd, *J* = 15.0, 11.4 Hz, 1H), 5.89 (ddd, *J* = 14.6, 9.4, 4.7 Hz, 1H), 5.82 (s, 1H), 5.62 (t, *J* = 8.3 Hz, 1H), 5.22 (dt, *J* = 10.6, 1.7 Hz, 1H), 5.09 (t, *J* = 9.7 Hz, 1H), 4.99 (d, *J* = 10.1 Hz, 1H), 4.77 (d, *J* = 1.2 Hz, 1H), 4.70 (q, *J* = 5.2 Hz, 1H), 4.67 (s, 1H), 4.29 – 4.23 (m, 1H), 4.07 – 3.98 (m, 2H), 3.95 (d, *J* = 3.5 Hz, 1H), 3.90 – 3.84 (m, 1H), 3.83 – 3.78 (m, 2H), 3.74 – 3.68 (m, 3H), 3.63 – 3.56 (m, 3H), 3.54 (s, 3H), 3.00 (q, *J* = 7.3 Hz, 2H), 2.76 – 2.60 (m, 5H), 2.56 (sept, *J* = 7.0 Hz, 1H), 2.52 – 2.38 (m, 2H), 1.98 – 1.89 (m, 1H), 1.80 (s, 3H), 1.72 (s, 3H), 1.65 (s, 3H), 1.27 (d, *J* = 6.2 Hz, 3H), 1.26 – 1.23 (m, 1H), 1.21 (t, *J* = 7.4 Hz, 3H), 1.18 – 1.11 (m, 12H), 1.09 (s, 3H), 0.83 (t, *J* = 7.4 Hz, 3H) ppm; **^{13}C NMR** (126 MHz, acetone-d_6) δ 176.77, 169.41, 168.63, 155.80, 153.81, 143.09, 142.60, 141.28, 136.86, 136.14, 136.04, 133.84, 128.54, 126.30, 126.08, 123.93, 114.51, 110.66, 108.19, 102.53, 96.76, 93.35, 81.61, 78.16, 77.63, 75.70, 73.76, 72.92, 72.82, 72.24, 70.52, 70.15, 69.08, 67.62, 61.77, 42.00, 37.16, 34.78, 28.72, 28.24, 28.09, 26.52, 26.20, 20.74, 19.41, 19.17, 18.59, 18.17, 17.48, 15.19, 14.37, 13.75, 11.17 ppm; **HRMS** ESI(−) (MeCN) calculated for $C_{53}H_{75}Cl_2O_{18}$ $[M-H]^-$: 1069.43359, found: 1069.43427.

19

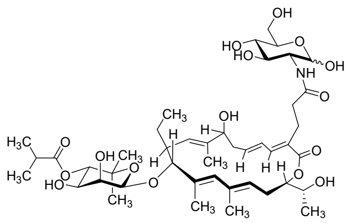

In a flame-dried flask under argon atmosphere, carboxylic acid **(*E*)-13** (18.3 mg, 25.9 µmol, 1.0 eq.), D-glucosamine hydrochlorid (8.5 mg, 39 µmol, 1.5 eq.) and HATU (14.8 mg, 38.8 µmol, 1.5 eq.) were dissolved in dry DMF (0.2 mL). Next, freshly distilled DIPEA (11 µL, 63 µmol, 2.4 eq.) was added and the resulting mixture was stirred at room temperature for 3 h. It was then quenched with water (2 mL) and lyophilized. The crude residue was purified by preparative RP-HPLC [Gemini NX, C18, 5 µ, 110 Å, 250 mm × 21.2 mm, solvent A: H_2O + 0.1% HCOOH, solvent B: MeCN + 0.1% HCOOH, 20 mL/min; LC time program (min –% B): 0.0 min – 30%, 15.0 min – 30%, 45.0 min – 35%, 45.2 min –100%] to afford, after lyophilization, FDX-glucosamide **19** (a/b=3:1, t_R = 16.8 and 18.5 min, 15.8 mg, 18.2 µmol, 70%) as a colorless solid.

TLC (MeOH:CH_2Cl_2. 1:4 v/v) = 0.38; **Specific Rotation** $[\alpha]_D^{25\,°C}$ = +23.70 (c = 0.75, MeOH); **FT-IR** $\tilde{\nu}$ (film) 363, 3404, 2974, 2939, 1680, 1643, 1539, 1386, 1307, 1250, 1212, 1147, 1077, 1037, 849, 559 cm^{-1}; <u>α:</u> **^{1}H NMR** (500 MHz, CD_3OD) δ 7.08 (d, *J* = 11.4 Hz, 1H), 6.52 – 6.44 (m, 1H), 5.87 – 5.80 (m, 2H), 5.55 (t, *J* = 8.3 Hz, 1H), 5.12 (d, *J* = 11.5 Hz, 1H), 5.10 (d, *J* = 3.5 Hz, 1H), 5.02 (d, *J* = 10.2 Hz, 1H), 4.73 – 4.70 (m, 1H), 4.67 (d, *J* = 5.0 Hz, 1H), 4.22 (s, 1H), 4.04 (quint, *J* = 6.3 Hz, 1H), 3.92 (dd, *J* = 3.3, 1.2 Hz, 1H), 3.82 – 3.78 (m, 2H), 3.74 – 3.66 (m, 4H), 3.35 (t, *J* = 9.2 Hz, 1H), 2.76 – 2.55 (m, 6H), 2.51 – 2.43 (m, 1H), 2.43 – 2.31 (m, 3H), 2.05 – 1.98 (m, 1H), 1.82 – 1.79 (m, 3H), 1.75 (s, 3H), 1.66 (s, 3H), 1.33 – 1.24 (m, 1H), 1.20 – 1.11 (m, 15H), 0.88 (t, *J* = 7.4 Hz, 3H) ppm; **^{13}C NMR** (126 MHz, CD_3OD) δ 178.4, 175.5, 169.9, 143.0, 141.3, 137.16, 137.0, 136.1, 134.7, 128.9, 128.64, 126.9, 124.6, 97.2, 94.3, 92.5, 78.6, 76.0, 74.5, 73.6, 73.2, 73.1, 72.6, 72.5, 70.6, 68.2, 62.9, 56.0, 42.5, 37.3, 36.5, 35.4, 28.7, 28.1, 26.9, 24.2, 20.1, 19.5, 19.1, 18.7, 17.5, 15.4, 13.9, 11.3 ppm; <u>β:</u> **^{1}H NMR** (500 MHz, CD_3OD) δ 7.08 (d, *J* = 11.4 Hz, 1H), 6.52 – 6.44 (m, 1H), 5.87 – 5.80 (m, 2H), 5.55 (t, *J* = 8.3 Hz, 1H), 5.12 (d, *J* = 11.5 Hz, 1H), 5.02 (d, *J* = 10.2 Hz, 1H), 4.73 – 4.70 (m, 1H), 4.67 (d, *J* = 5.0 Hz, 1H), 4.58 (d, *J* = 8.3 Hz, 1H), 4.22 (s, 1H), 4.04 (quint, *J* = 6.3 Hz, 1H), 3.92 (dd, *J* = 3.3, 1.2 Hz, 1H), 3.86 (dd, *J* = 12.0, 2.1 Hz, 1H), 3.66 (d, *J* = 5.5 Hz, 1H), 3.62 – 3.54 (m, 1H), 3.46 – 3.41 (m, 1H), 3.30 – 3.27 (m, 1H), 2.76 – 2.55 (m, 6H), 2.51 – 2.43 (m, 1H), 2.43 – 2.31 (m, 3H), 2.05 – 1.98 (m, 1H), 1.82 – 1.79 (m, 3H), 1.75 (s, 3H), 1.66 (s, 3H), 1.33 – 1.24 (m, 1H), 1.20 – 1.11 (m, 15H), 0.88 (t, *J* = 7.4 Hz, 3H) ppm; **^{13}C NMR** (126 MHz, CD_3OD) δ 178.4, 175.5, 169.9, 142.9, 141.3, 137.16, 137.0, 136.1, 134.7, 128.9, 128.58, 126.8, 124.6, 97.2, 97.1, 94.3, 78.6, 76.10, 76.0, 74.5, 73.6, 73.2, 72.2, 70.6, 68.2, 62.9, 58.9, 42.5, 37.3, 36.8, 35.4, 28.7, 28.1, 26.9, 24.2, 20.1, 19.5, 19.1, 18.7, 17.5, 15.4, 13.9, 11.3 ppm; **HRMS** ESI(+), (MeOH) calculated for $C_{44}H_{69}NO_{16}Na$ $[M+Na]^+$: 890.45086, found: 890.45082.

20

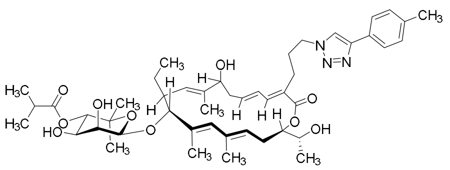

Alcohol **15** (20.0 mg, 28.9 µmol, 1.0 eq.) and Bis(2,4-dichlorophenyl) chlorophosphate (35.2 mg, 86.7 µmol, 3.0 eq.) was dissolved in CH_2Cl_2 (200 µL). Then DIPEA (19 µL, 116 µmol. 4.0 eq.) was added and the reaction mixture was stirred for 1 h at room temperature. Upon completion of the reaction as monitored by TLC ($MeOH/CH_2Cl_2$ 1:19), the reaction was quenched with water and the layers were separated. The organic layer was extracted with water (3x). The combined organic layers were dried over $MgSO_4$, filtered and the solvent was evaporated under a nitrogen flow. The resulting crude phosphate ester was dissolved in DMF (200 µL), NaN_3 (5.6 mg, 87 µmol, 3.0 eq.) was added and the reaction mixture was heated to 80 °C for 1.5 h. Upon completion of the reaction as indicated by UHPLC-MS, the reaction mixture was diluted with CH_2Cl_2 and quenched with water. The layers were separated and the organic layer was washed with brine (2x). The combined organic layers were dried over $MgSO_4$, filtered and the solvent was evaporated under reduced pressure. The resulting azide was dissolved in CH_2Cl_2 (500 µL) and the reaction mixture was degassed by freeze-pump-thaw (3x). Then, Cu(I)-cat. **21**[2] (3.9 mg, 5.5 µmol, 20 mol%) and 1-Ethynyl-4-methylbenzene (7 µL, 55 µmol, 2.0 eq.) were added and the reaction was stirred for 4 h at room temperature. The reaction was diluted with CH_2Cl_2 and washed with brine (3x). The organic layer was dried over $MgSO_4$, filtered and the solvent was evaporated. The crude product was purified by silica-gel column chromatography ($MeOH/CH_2Cl_2$ 1:39) to give triazole **20** (4.9 mg, 5.9 µmol, 21%) as a white solid.

or

Alcohol **15** (10.0 mg, 14.4 µmol, 1.0 eq.) was dissolved in toluene (500 µL) and a solution of DPPA-NO_2[2] (52.6 mg, 144 mol, 10 eq.) and DBU (25.8 µL, 173 µmol, 12 eq.) in toluene (1.5 mL) was added dropwise at room temperature. The reaction mixture was stirred at 50°C until completion of the reaction was observed by UHPLC-MS (2h30). The reaction mixture was concentrated under reduced pressure and the resulting crude mixture was dissolved in CH_2Cl_2 (250 µL) followed by freeze-pump-thaw (3x). Then, Cu(I)-cat. **21**[2] (5.1 mg, 7.2 µmol, 50 mol%) and 1-Ethynyl-4-methylbenzene (3.65 µL, 29 µmol, 2.0 eq.) were added and the reaction was stirred for 4 h at room temperature. The reaction was diluted with CH_2Cl_2 and washed with brine (3x). The layers were separated and the organic layer was washed with brine (2x). The combined organic layers were dried over $MgSO_4$, filtered and the solvent was evaporated under reduced pressure. The crude product was purified by silica-gel column chromatography ($MeOH/CH_2Cl_2$ 1:39) to give triazole **20** (6.3 mg, 8 µmol, 53%) as a slightly yellow solid.

Specific Rotation $[\alpha]_D^{23\,°C}$ = 170.0 (c = 1.15, MeOH); **FT-IR** $\tilde{\nu}$ (film) cm^{-1}; 3452, 2973, 2930, 2874, 1735, 1697, 1641, 1500, 1384, 1368, 1317, 1249, 1202, 1150, 1079, 1034, 900, 820, 797 cm^{-1}; **^{1}H NMR** (500 MHz, acetone-*d*$_6$) δ 8.28 (s, 1H), 7.78 (d, *J* = 8.1 Hz, 2H), 7.25 (d, *J* = 7.9 Hz, 2H), 7.03 (d, *J* = 11.4 Hz, 1H), 6.39 – 6.29 (m, 1H), 5.87 – 5.77 (m, 2H), 5.56 (t, *J* = 8.3 Hz, 1H), 5.18 (d, *J* = 10.6 Hz, 1H), 4.98 (d, *J* = 10.1 Hz, 1H), 4.75 (s, 1H), 4.69 – 4.64 (m, 1H), 4.45 (t, *J* = 7.0 Hz, 2H), 4.25 – 4.21

[2] DPPA-NO_2 was prepared according to the procedure of Shioiri and Yamada.[9]

(m, 1H), 4.12 (d, J = 5.2 Hz, 1H), 4.06 (quint, J = 6.0 Hz, 1H), 3.96 – 3.92 (m, 1H), 3.81 (d, J = 3.6 Hz, 1H), 3.75 – 3.66 (m, 3H), 3.62 – 3.59 (m, 1H), 3.25 (d, J = 9.5 Hz, 1H), 2.78 – 2.73 (m, 2H), 2.67 – 2.36 (m, 6H), 2.35 (s, 3H), 2.19 – 2.08 (m, 2H), 1.99 – 1.90 (m, 1H), 1.78 (d, J = 1.3 Hz, 3H), 1.70 (d, J = 1.3 Hz, 3H), 1.64 (s, 3H), 1.26 – 1.21 (m, 0H), 1.14 (dd, J = 8.5, 6.2 Hz, 12H), 1.07 (s, 3H), 0.82 (t, J = 7.5 Hz, 3H) ppm. **^{13}C NMR** (126 MHz, acetone-d_6) δ 176.77, 168.63, 147.86, 141.87, 141.11, 138.17, 136.90, 136.30, 135.86, 133.96, 130.19, 129.63, 128.31, 128.24, 126.74, 126.20, 123.99, 121.10, 96.68, 93.27, 78.23, 75.72, 73.72, 72.99, 72.81, 70.18, 67.62, 50.39, 42.11, 37.29, 34.78, ~30 (under solvent signal), 28.70, 27.92, 26.47, 24.55, 21.22, 20.43, 19.41, 19.17, 18.61, 17.38, 15.24, 13.83, 11.18 ppm; **HRMS** ESI(+), (MeOH) calculated for $C_{47}H_{68}N_3O_{10}Na$ [M+H]$^+$: 834.48992, found: 834.49000.

Determination of the Minimum Inhibitory Concentration

General procedure for the determination of MIC values of *M. tuberculosis*

MIC determination was carried out by Daniel Schäfle in the research group of Prof. Dr. Peter Sander (University of Zurich). MIC determination was essentially conducted as described recently.[3] Briefly, the Green-Fluorescent Protein (GFP) expressing recombinant *Mycobacterium tuberculosis* H37Rv *rpsL*[4] transformed with pOLYG-Pr-GFP[5] was grown in Middlebrook 7H9-OADC with 0.05% Tween 80 until mid-log phase (optical density at 600 nm OD_{600} = 0.3 – 1.0), diluted to an OD_{600} of 0.04 and 20 µl of the suspension were added to an equal volume of 12-point 2-fold serial dilutions of the compounds in 7H9-OADC-Tween in 384-well plates in triplicates. Compound concentrations were in the range of 62.5 to 0.031 µM. Fluorescence was measured immediately after inoculation (background) and after 10 days of incubation at 37 °C. Dose response curves were fitted with a 4-parameter log-normal model. P_{MIN} [-,-] and P_{MAX} [-, 120] are the minimum and the maximum, respectively, P_{Hill} [0,-] indicates the steepness, and EC_{50} [-,-] the log-back transformed Minimal Effective Concentration 50. The computational and statistical analysis was conducted with R (3.0.1 – 3.1.1; https://www.r-project.org/). Dose response curves were fitted with the ´drc´ package. The inhibitory potency I was calculated with the equation I = 100-[100●(S-P)/N-P)]. S is the sample's fluorescence while P and N derive from growth inhibition with the control drug (Kanamycin A) and solvent growth control measurements (DMSO 1.25% vol./vol.), respectively. A fluorescence reduction of 90% as compared to the no-drug control was reported as Minimal Inhibitory Concentration (MIC_{90}).

General procedure for the determination of MIC values of *C. difficile*

MIC determination was carried out by Micromyx, LLC, 4717 Campus Drive, Kalamazoo, MI, USA 49008. Approximately 5 mg of each of 20 test compounds were provided. These were stored at -20°C until testing. On the day of the assay, the test articles were dissolved in 100% DMSO (dimethyl sulfoxide, Sigma; St. Louis, MO, Cat. No. 472301-500ML, Lot No. SHBH5551V) to a stock concentration of 3232 µg/mL. The concentration range tested for these test agents was 16 – 0.015 µg/mL. The comparator agents, metronidazole and clindamycin were supplied by Micromyx, as shown in the table below:

Comparator Drug	Supplier	Catalog No.	Lot No.	Solvent/Diluent	Testing Range (µg/mL)
Metronidazole	Sigma	M3761-100G	095K0693	DMSO/$_dH_2O$	64 – 0.06
Clindamycin	Sigma	C5269-100MG	021M1533	$_dH_2O$/ $_dH_2O$	32 – 0.03

Test Organisms

Test organisms consisted of reference strains from the American Type Culture Collection (ATCC; Manassas, VA) and clinical isolates from the Micromyx repository (MMX; Kalamazoo, MI). Organisms were initially received at Micromyx and were streaked for isolation. Colonies were picked by sterile swab from the medium and suspended in the appropriate broth containing cryoprotectant. The suspensions were aliquoted into cryogenic vials and maintained at −80ºC.

Prior to testing, all isolates were streaked onto Brucella Agar supplemented with hemin, Vitamin K and 5% sheep blood (Becton Dickinson [BD]; Sparks, MD, Cat. No. 297716, Lot No. 8256909) and incubated anaerobically at 35 − 37°C for 44 – 48 h.
Additionally, *B. fragilis* ATCC 25285 and *C. difficile* ATCC 700057 were tested for purposes of quality control.

Test Medium

The medium employed for anaerobic testing in the broth microdilution MIC assay was Brucella Broth (BD, Cat. No. 211088, Lot No. 7128995), supplemented with hemin (Sigma, Lot No. SLBP5720V), Vitamin K (Sigma, Lot No. MKCG2075) and 5% laked horse blood (LHB, Cleveland Scientific; Bath, OH, Lot No. 474990).

Broth Microdilution Assay

The MIC assay method followed the procedure described by the CLSI[6,7] and employed automated liquid handlers (Multidrop 384, Labsystems, Helsinki, Finland; Biomek 2000 and Biomek FX, Beckman Coulter, Fullerton CA) to conduct serial dilutions and liquid transfers. The wells in columns 2 through 12 in a standard 96-well microdilution plate (Costar) were filled with 150 µL of the appropriate diluent (DMSO for the test agents; $_d$H2O for metronidazole and clindamycin). The drugs (300 µL at 101X the desired top concentration in the test plates) were dispensed into the appropriate well in column 1 of the mother plates. The Biomek 2000 was used to make serial 2-fold dilutions through column 11 in the "mother plate". The wells of column 12 contained no drug and were the organism growth control wells.

The daughter plates for testing of all isolates were loaded with 190 µL per well of supplemented Brucella broth with 5% LHB using the Multidrop 384. The daughter plates were prepared on the Biomek FX instrument which transferred 2 µL of 101X drug solution from each well of a mother plate to the corresponding well of each daughter plate in a single step. The wells of the daughter plates ultimately contained 190 µL of medium, 2 µL of drug solution, and 10 µL of bacterial inoculum prepared in broth.

A standardized inoculum of each organism was prepared per CLSI methods.[6,7] For all bacteria, suspensions were prepared in supplemented Brucella broth supplemented with hemin and Vitamin K to equal the turbidity of a 0.5 McFarland standard. These suspensions were further diluted 1:10 in supplemented Brucella broth with 5% LHB. The inoculum was dispensed into sterile reservoirs (Beckman Coulter) and transferred by hand in the Bactron Anaerobe chamber so that inoculation took place from low to high drug concentration. A 10 µL aliquot of inoculum was delivered into each well. Inoculated daughter plates were stacked and placed in an anaerobic box with GasPak sachets (BD; Lot No. 6309689), covered with a lid on the top plate, and incubated at 35 – 37°C.

The microplates were viewed from the bottom using a plate viewer after 46 h. For each mother plate, an un-inoculated solubility control plate was observed for evidence of drug precipitation. The MIC was read and recorded as the lowest concentration of drug that inhibited visible growth of the organism.

Supplementary References

1. Fulmer, G. R. *et al.* NMR Chemical Shifts of Trace Impurities: Common Laboratory Solvents, Organics, and Gases in Deuterated Solvents Relevant to the Organometallic Chemist. *Organometallics* **29**, 2176–2179 (2010).
2. Berg, R. *et al.* Highly active dinuclear copper catalysts for homogeneous azide-alkyne cycloadditions. *Adv. Synth. Catal.* **354**, 3445–3450 (2012).
3. Dal Molin, M. *et al.* Identification of novel scaffolds targeting *Mycobacterium tuberculosis*. *J. Mol. Med.* **97**, 1601–1613 (2019).
4. Raynaud, C. *et al.* The functions of OmpATb, a pore-forming protein of *Mycobacterium tuberculosis*. *Mol. Microbiol.* **46**, 191–201 (2002).
5. Matt, U. *et al.* Chloroquine enhances the antimycobacterial activity of isoniazid and pyrazinamide by reversing inflammation-induced macrophage efflux. *Int. J. Antimicrob. Agents* **50**, 55–62 (2017).
6. Clinical and Laboratory Standards Institute (CLSI). *Performance Standards for Antimicrobial Susceptibility Testing: 29th Edition*. *Clinical and Laboratory Standards Institute* (2019).
7. Clinical and Laboratory Standards Institute (CLSI). *Methods for Antimicrobial Susceptibility Testing of Anaerobic Bacteria: Ninth Edition*. *Methods for Antimicrobial Susceptibility Testing of Anaerobic Bacteria*, (2018).
8. Shimada, N., Nakamura, Y., Ochiai, T. & Makino, K. Catalytic Activation of *cis*-Vicinal Diols by Boronic Acids: Site-Selective Acylation of Carbohydrates. *Org. Lett.* **21**, 3789–3794 (2019).
9. Shiori, T. & Yamada, S. Amino Acids and Peptides. X. Phosphorus in Organic Synthesis. V. On the Mechanism for the Peptide Synthesis by Diphenyl Phosphorazidate. *Chem. Pharm. Bull. (Tokyo).* **22**, 855–858 (1974).

NMR Spectra

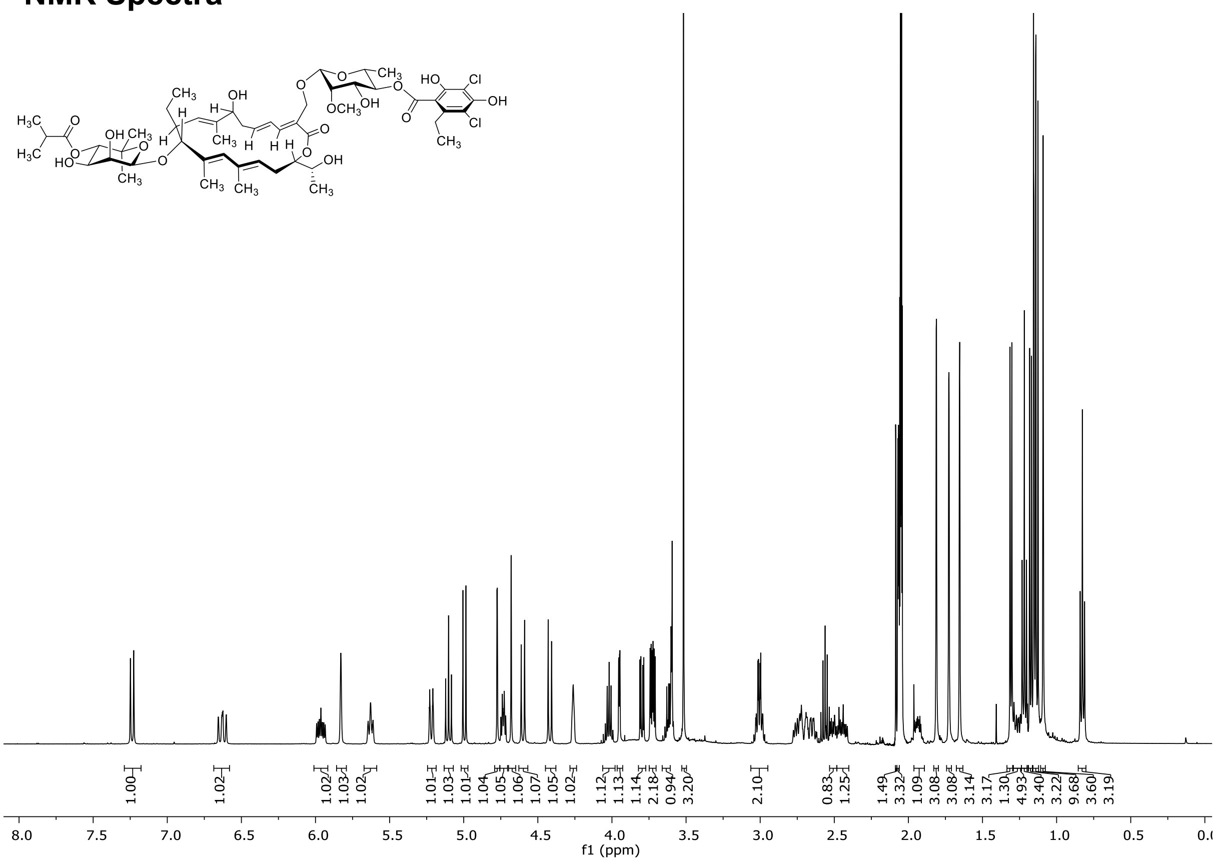

Supplementary Figure 2. ^{1}H NMR (500 MHz, acetone-d_6) of **Fidaxomicin (1)**.

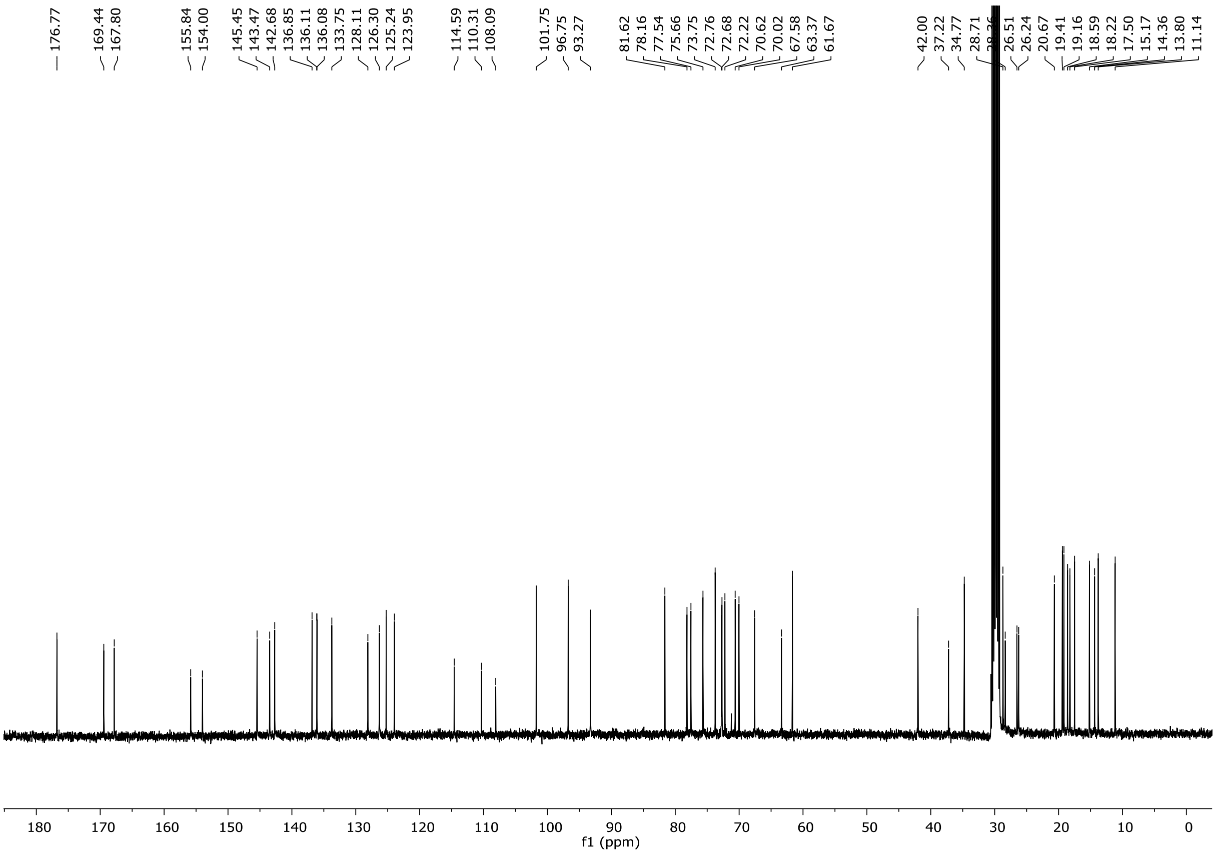

Supplementary Figure 3. ^{13}C NMR (101 MHz, acetone-d_6) of **Fidaxomicin (1)**.

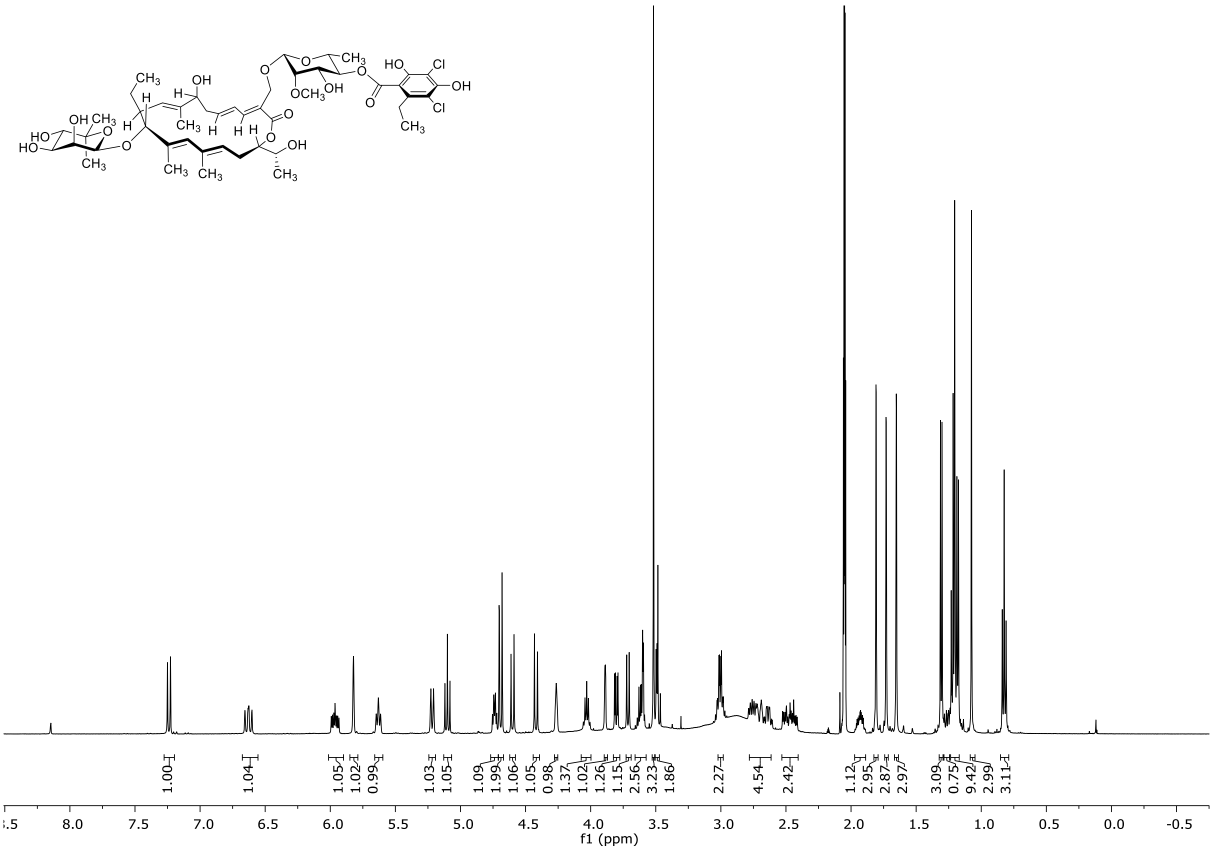

Supplementary Figure 4. ^{1}H NMR (500 MHz, acetone-d_6) of **OP-1118 (4)**.

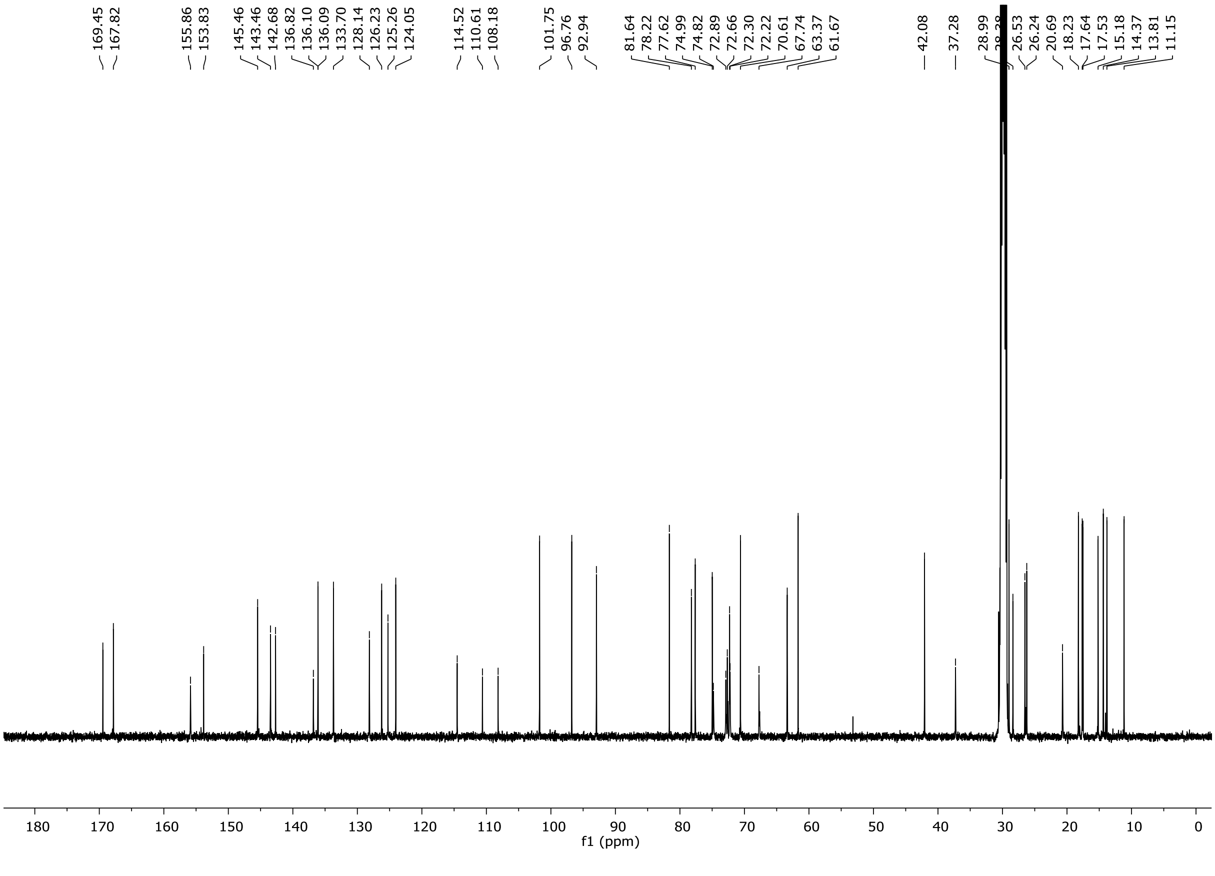

Supplementary Figure 5. ^{13}C NMR (126 MHz, acetone-d_6) of **OP-1118 (4)**.

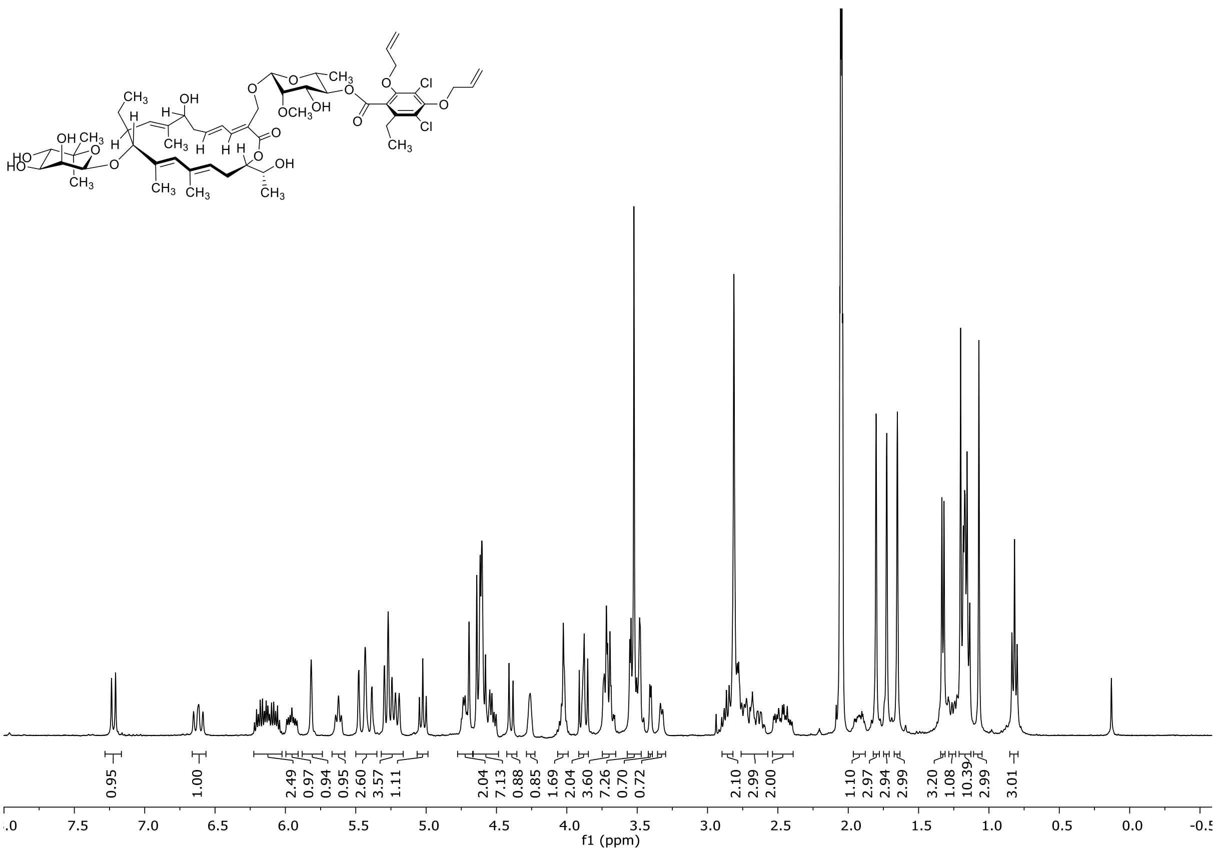

Supplementary Figure 6. ^{1}H NMR (400 MHz, acetone-d_6) of **Bisallyl-OP-1118 (5)**.

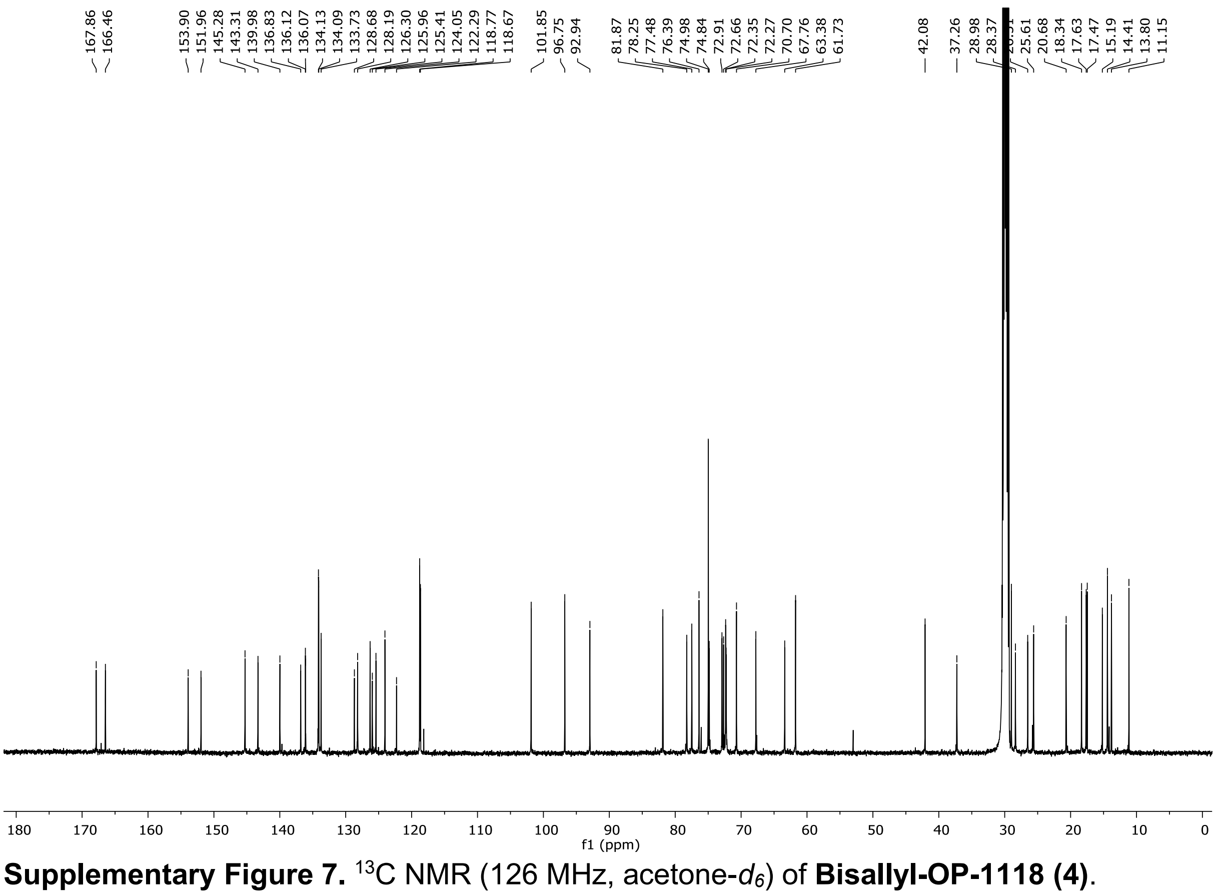

Supplementary Figure 7. ^{13}C NMR (126 MHz, acetone-d_6) of **Bisallyl-OP-1118 (4)**.

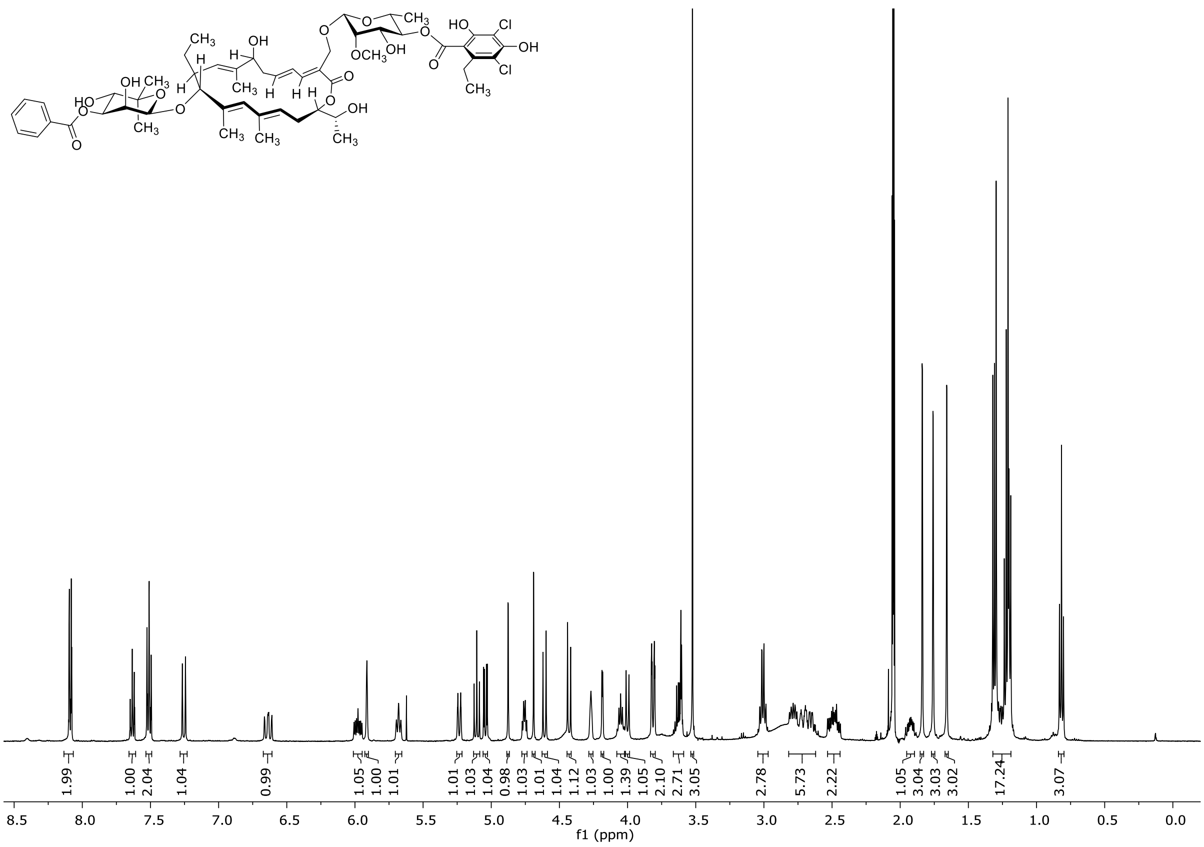

Supplementary Figure 8. ^{1}H NMR (500 MHz, acetone-d_6) of **7a**.

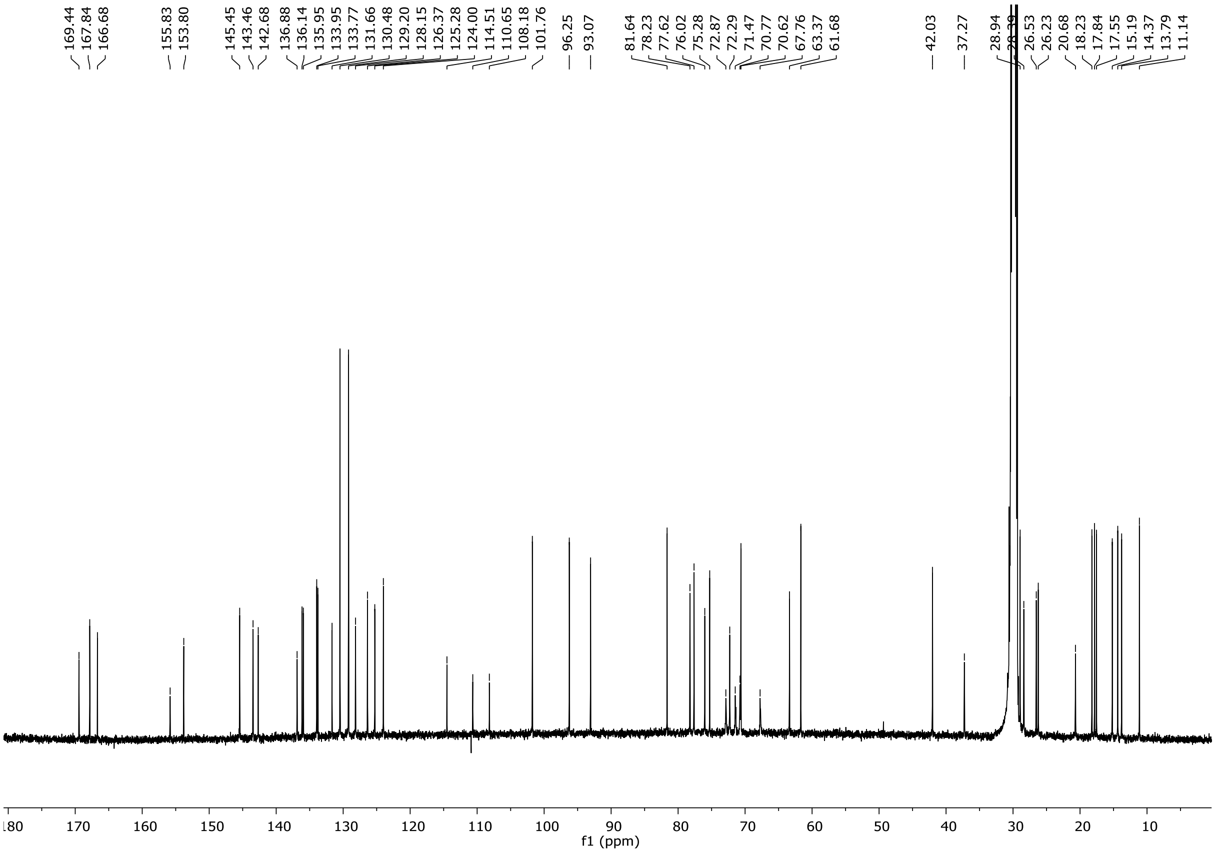

Supplementary Figure 9. ^{13}C NMR (126 MHz, acetone-d_6) of **7a.**

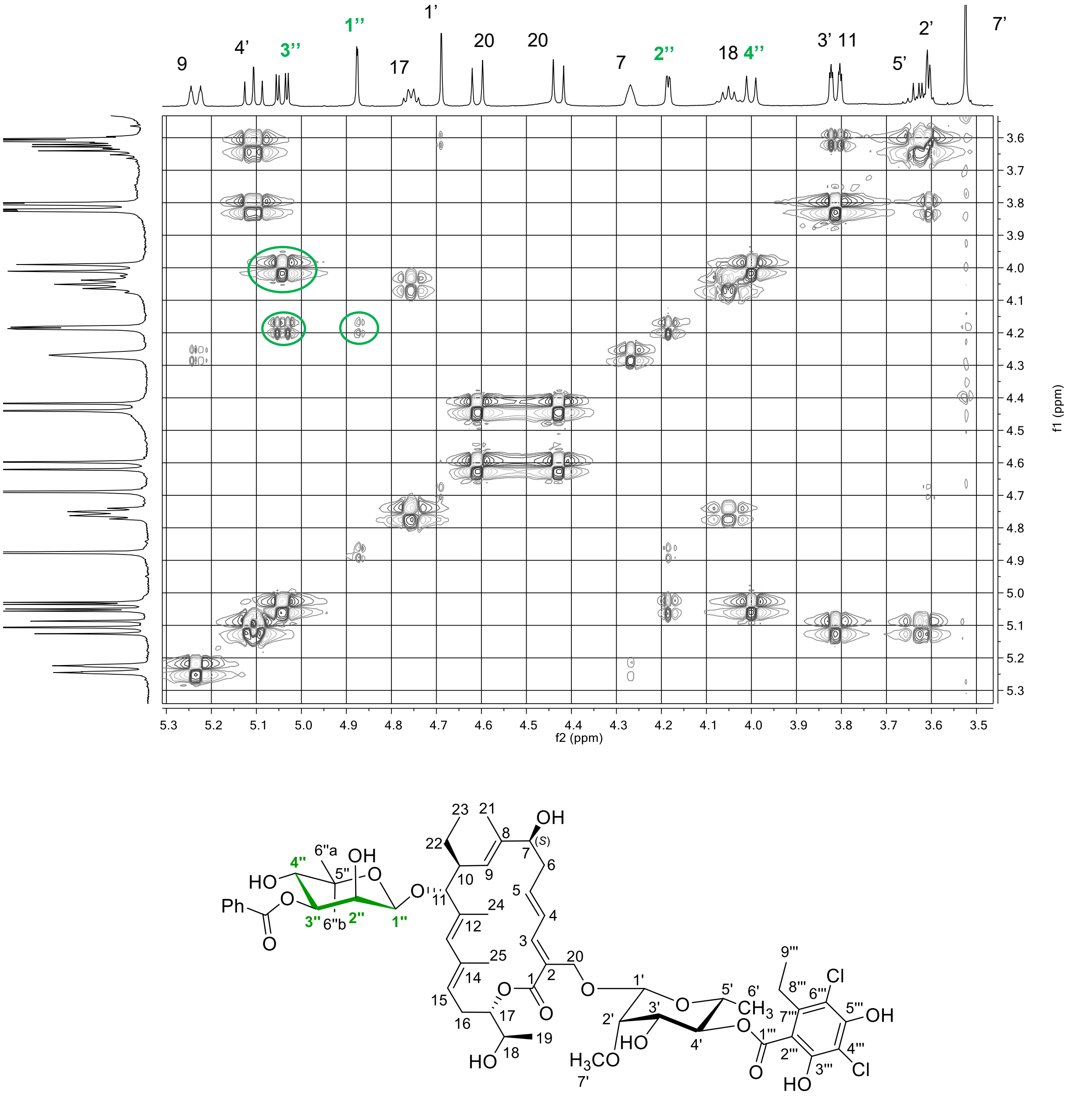

Supplementary Figure 10. COSY NMR (500 MHz, acetone-d_6) of **7a.**

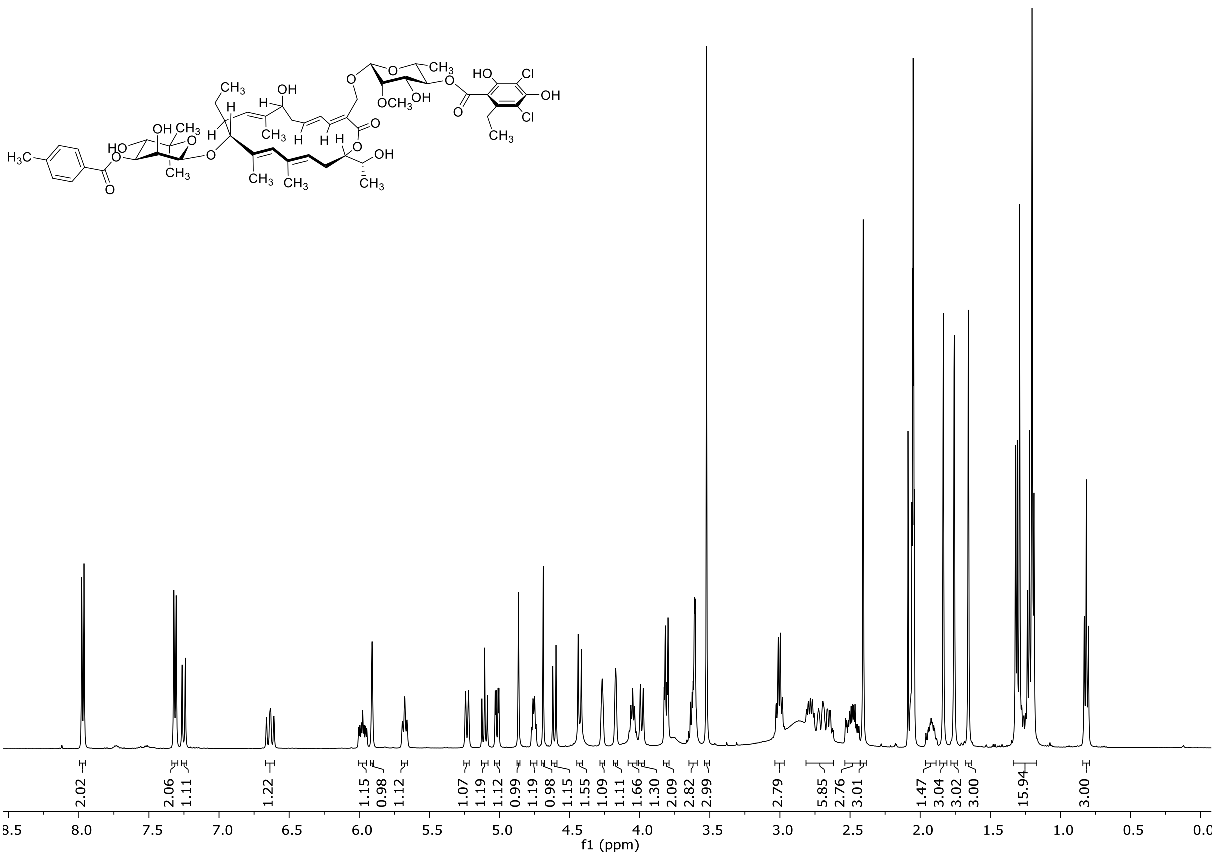

Supplementary Figure 11. ^{1}H NMR (500 MHz, acetone-d_6) of **7b**.

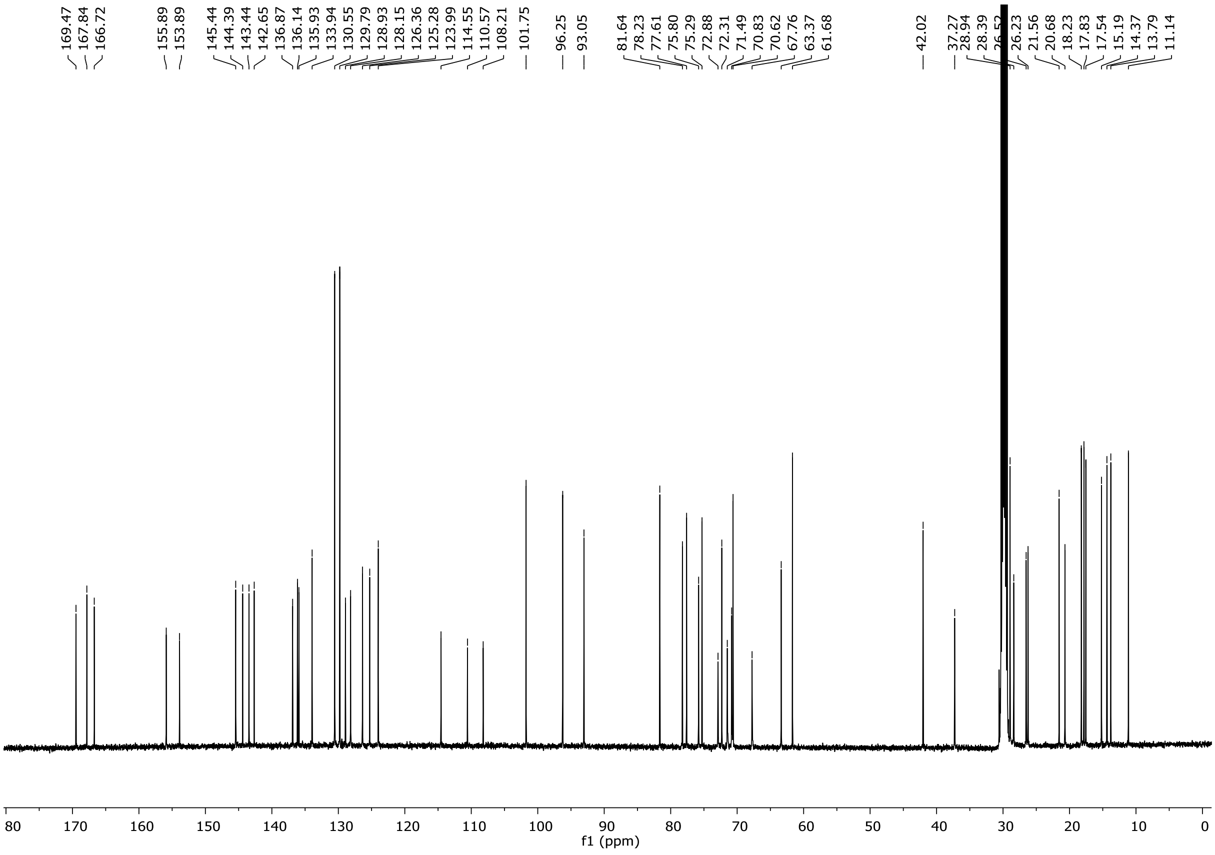

Supplementary Figure 12. ^{13}C NMR (126 MHz, acetone-d_6) of **7b.**

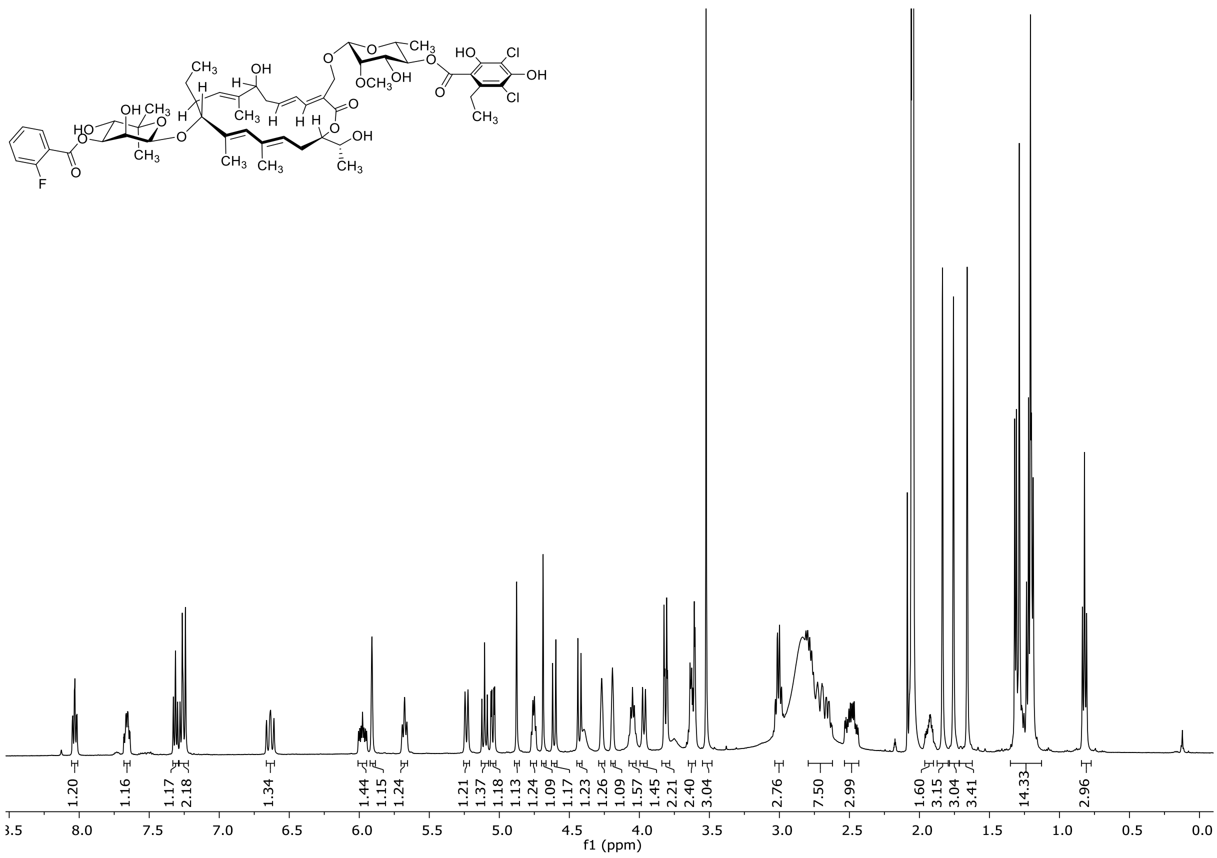

Supplementary Figure 13. ^{1}H NMR (500 MHz, acetone-d_6) of **7c**.

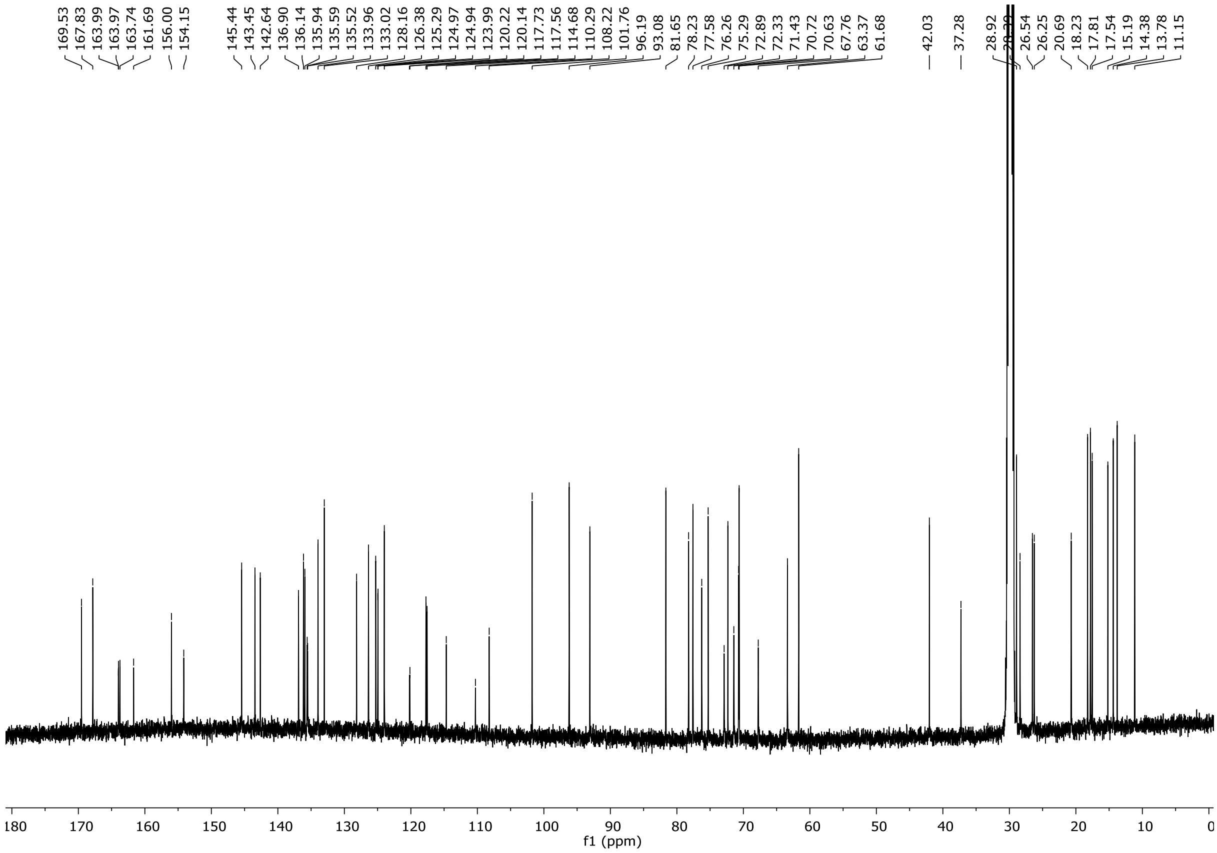

Supplementary Figure 14. ^{13}C NMR (126 MHz, acetone-d_6) of **7c**.

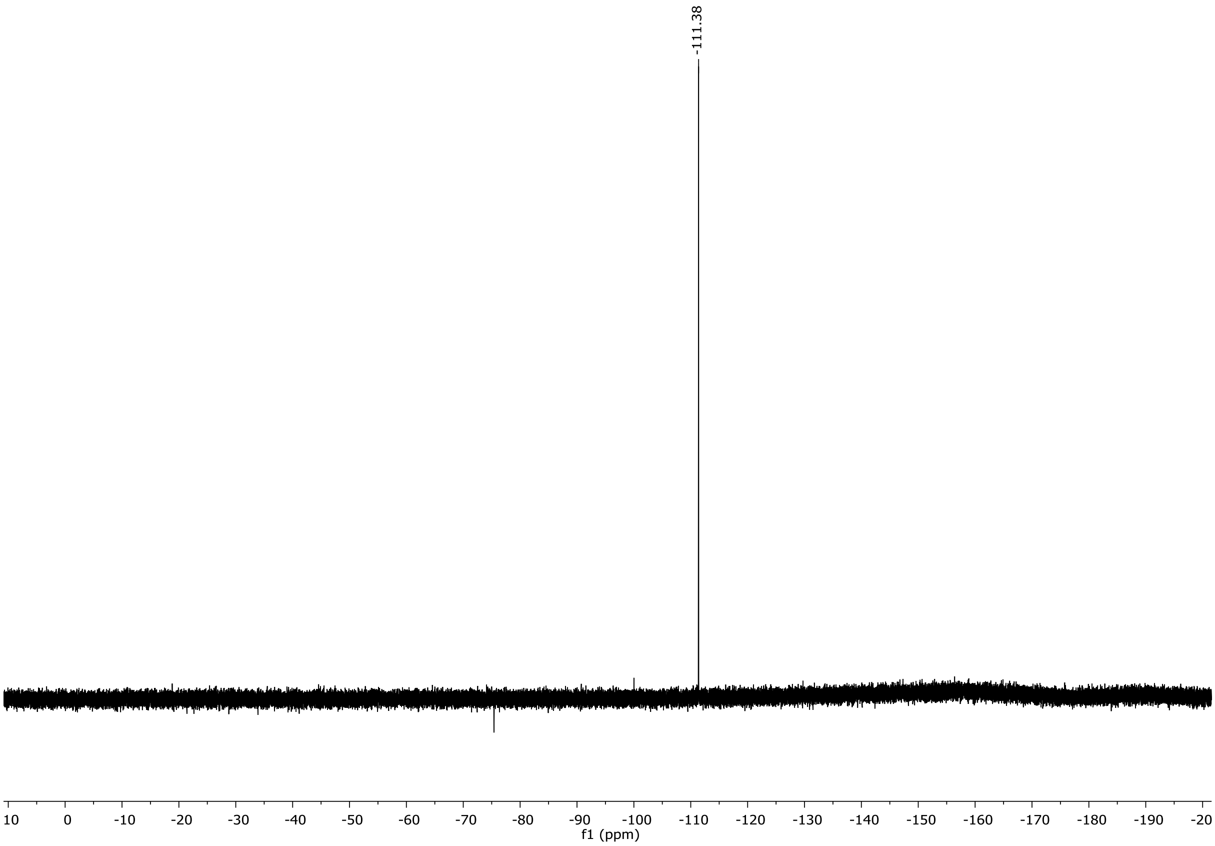

Supplementary Figure 15. ^{19}F NMR (376.5 MHz, acetone-d_6) of **7c**.

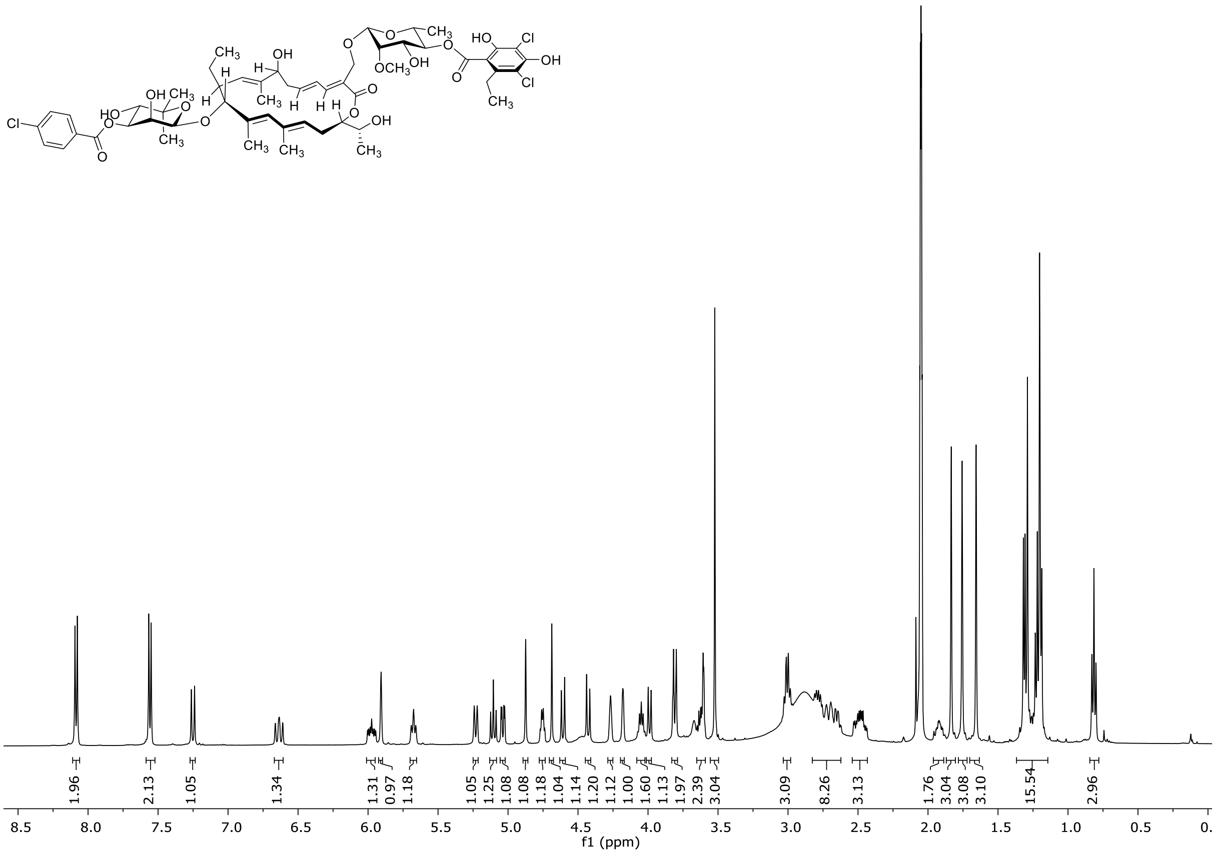

Supplementary Figure 16. ^{1}H NMR (500 MHz, acetone-d_6) of **7d**.

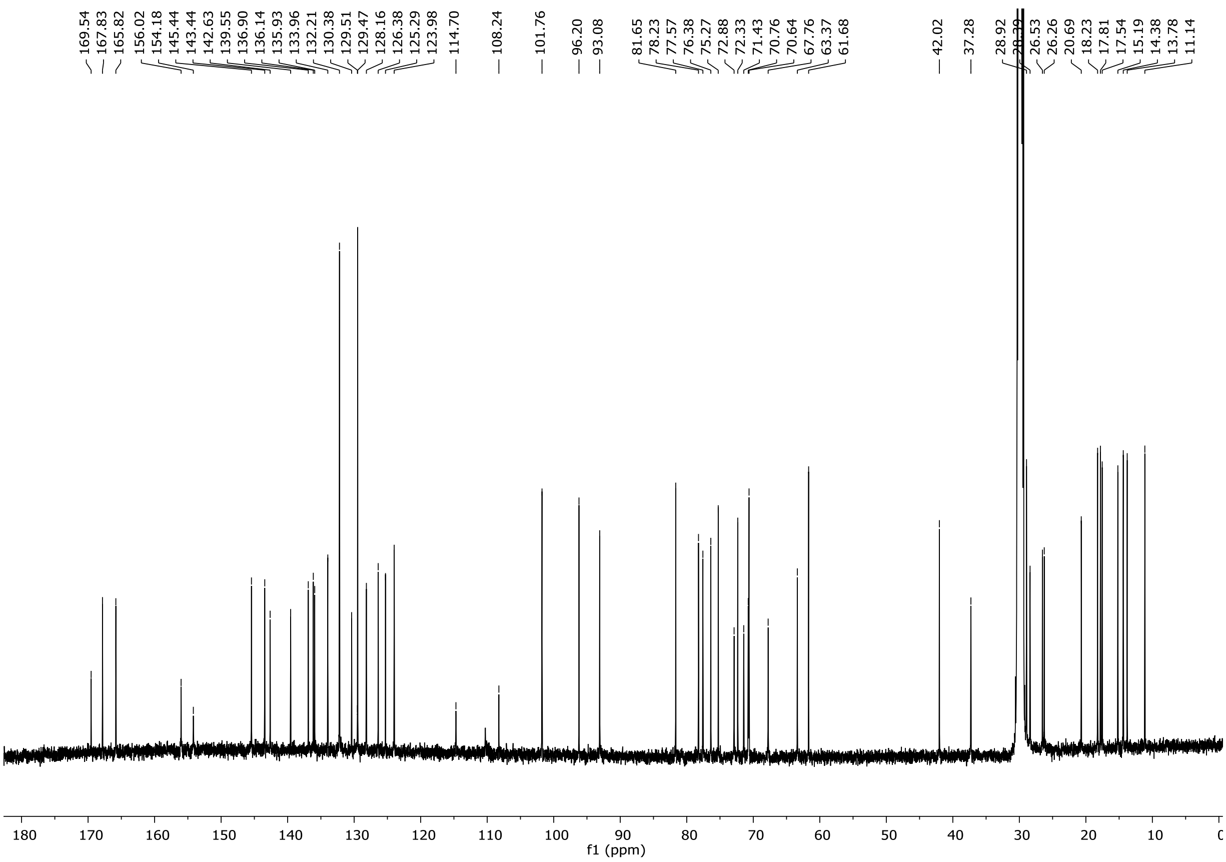

Supplementary Figure 17. ^{13}C NMR (126 MHz, acetone-d_6) of **7d.**

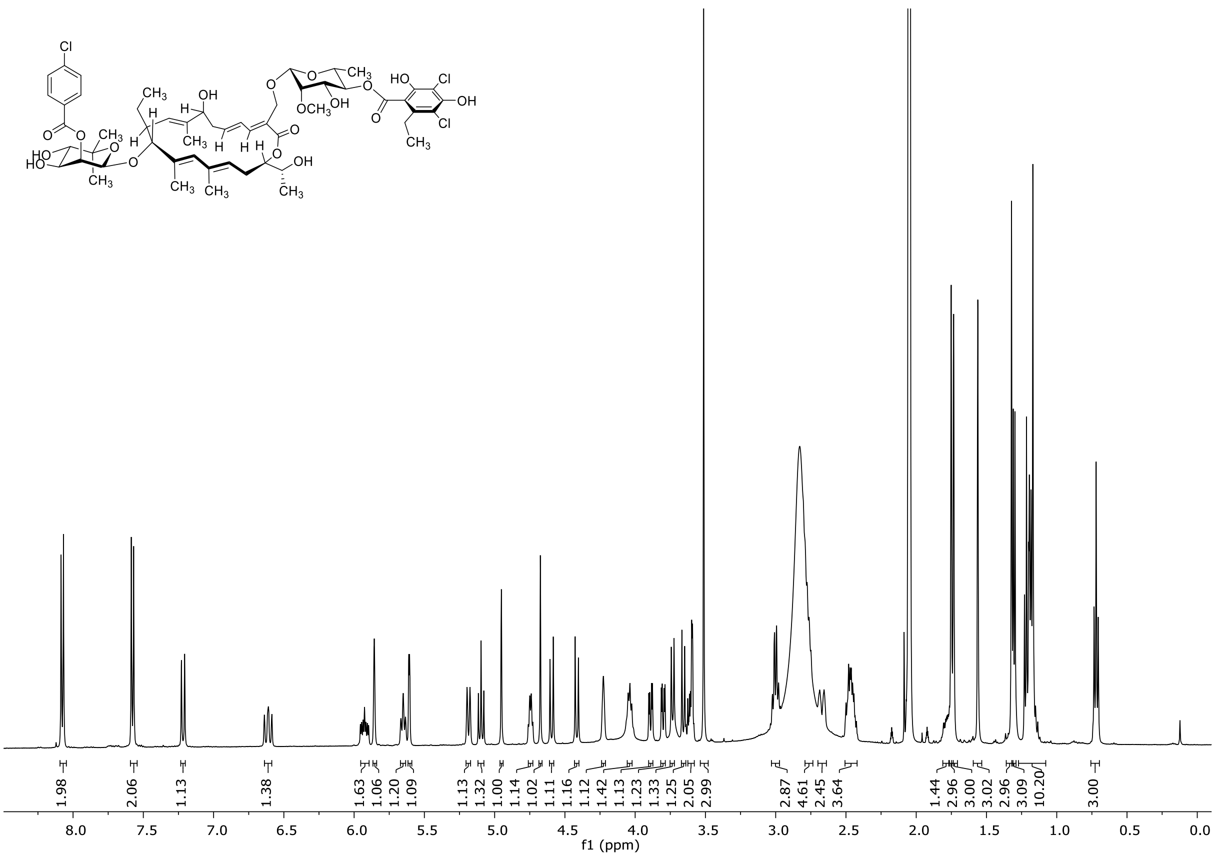

Supplementary Figure 18. ^{1}H NMR (500 MHz, acetone-d_6) of **7d-C2.**

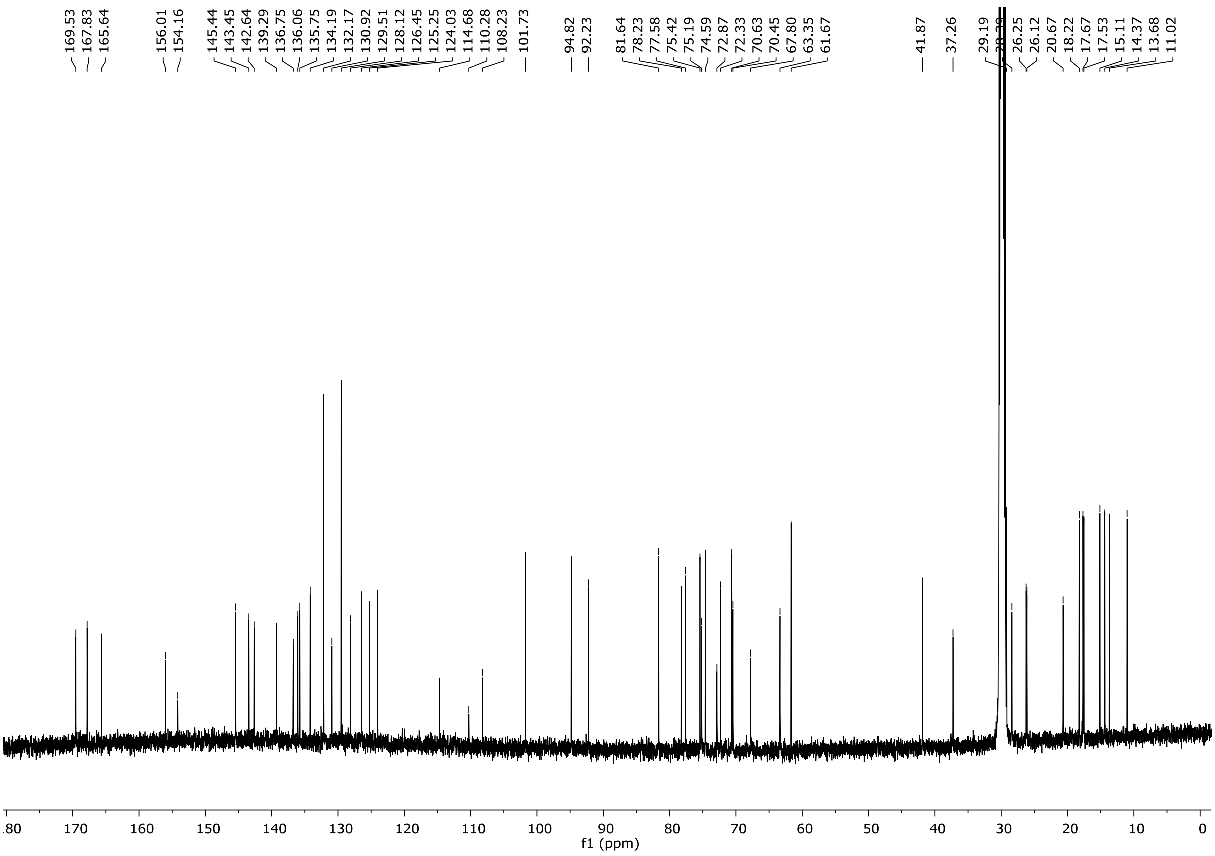

Supplementary Figure 19. ^{13}C NMR (126 MHz, acetone-d_6) of **7d-C2**.

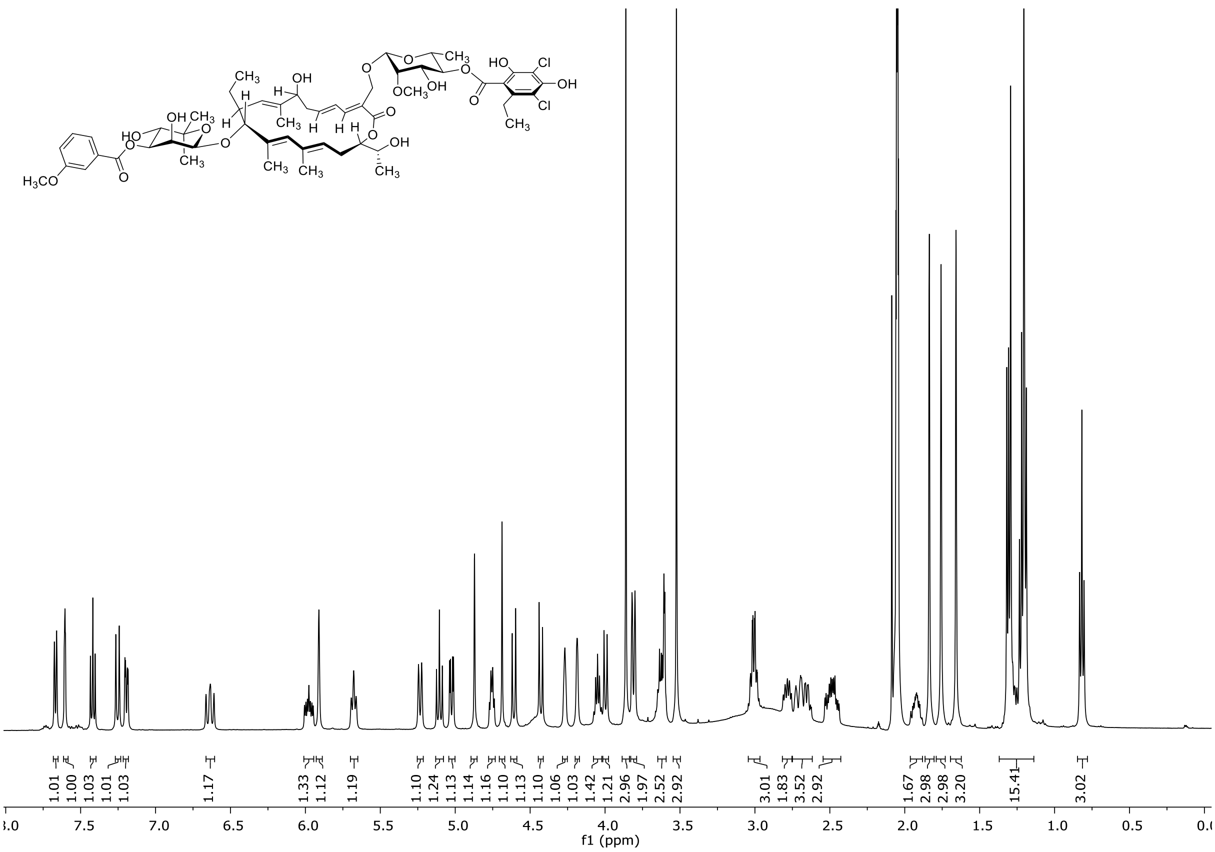

Supplementary Figure 20. ^{1}H NMR (500 MHz, acetone-d_6) of **7e**.

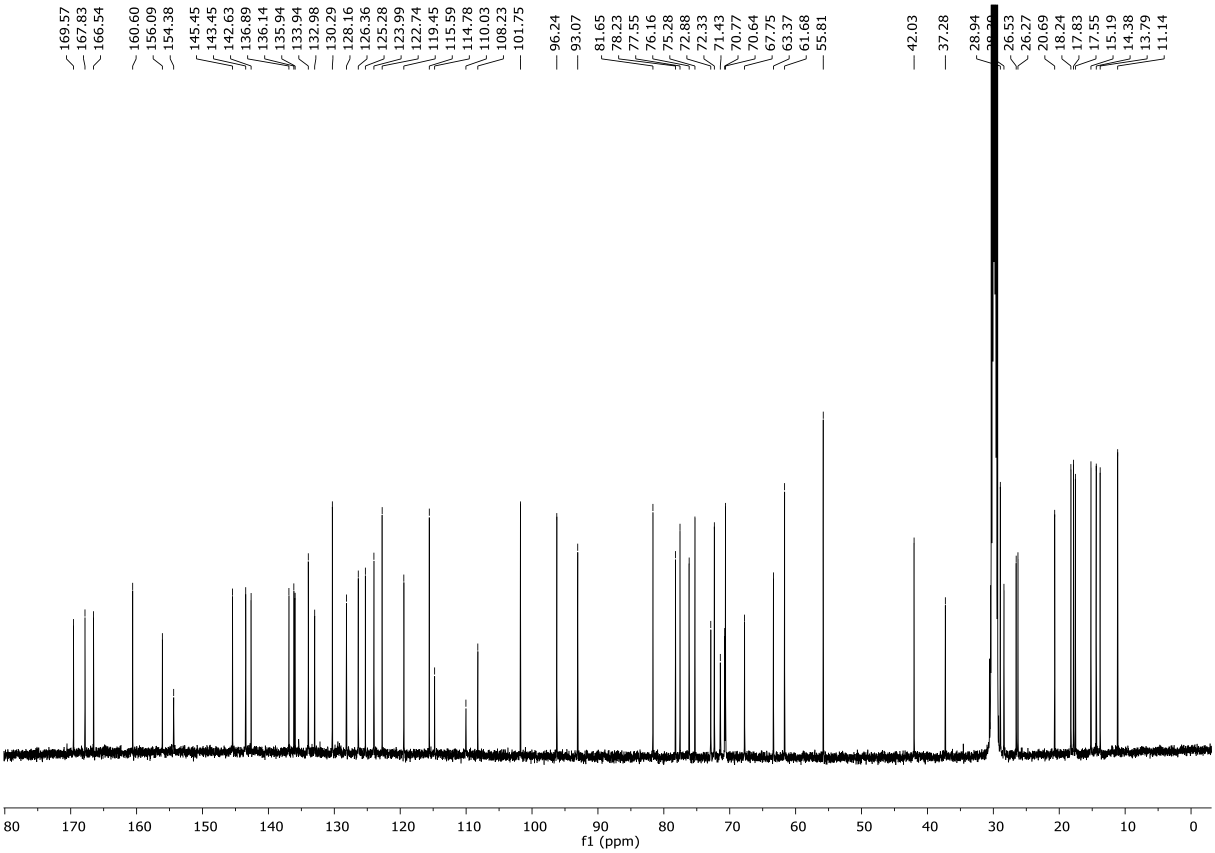

Supplementary Figure 21. ^{13}C NMR (126 MHz, acetone-d_6) of **7e**.

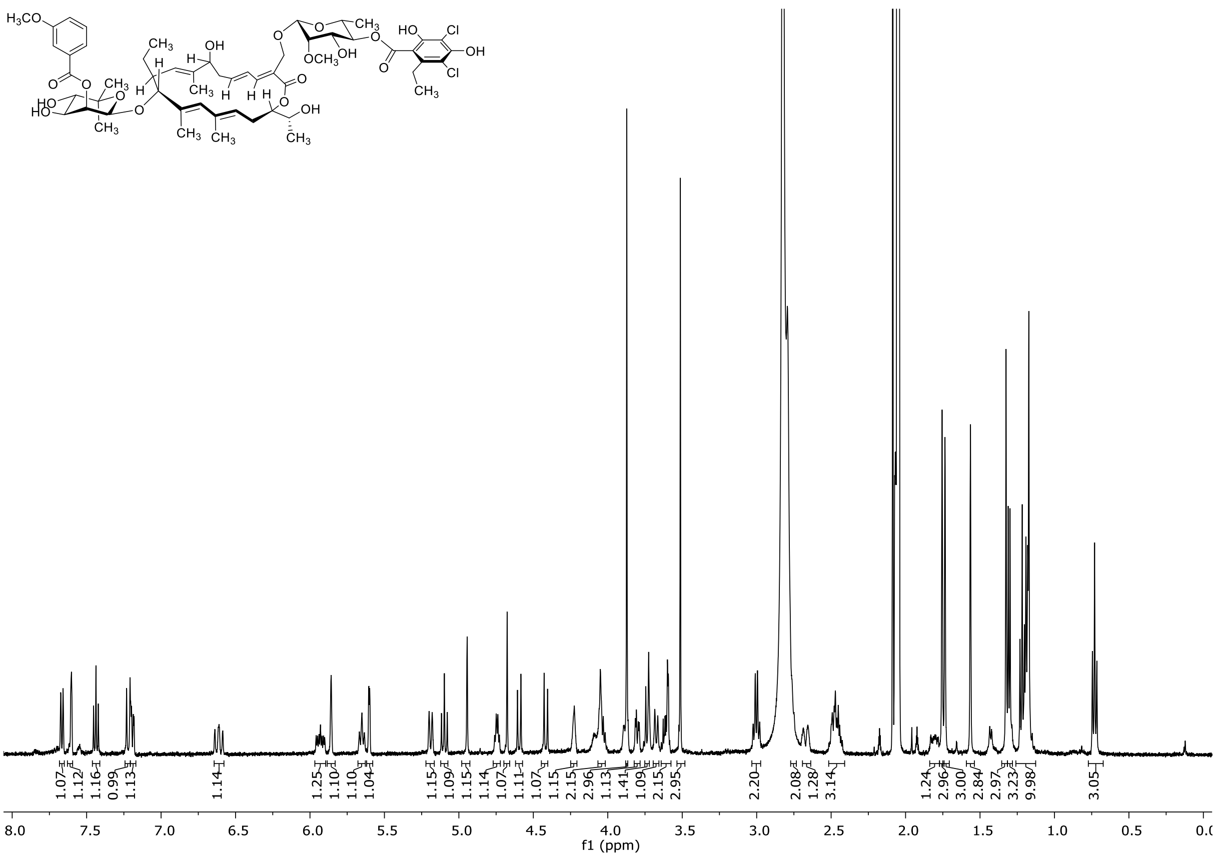

Supplementary Figure 22. ^{1}H NMR (500 MHz, acetone-d_6) of **7e-C2**.

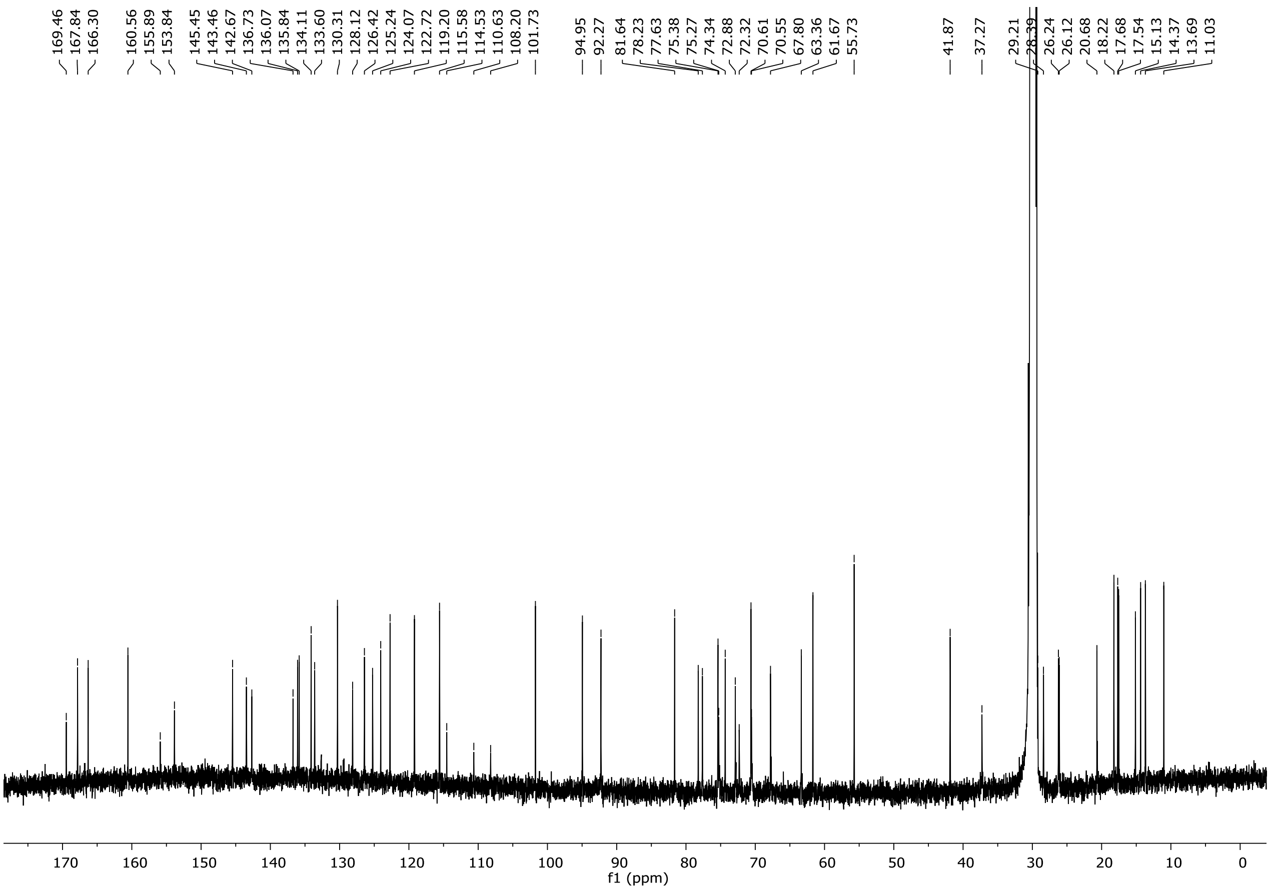

Supplementary Figure 23. ^{13}C NMR (126 MHz, acetone-d_6) of **7e-C2.**

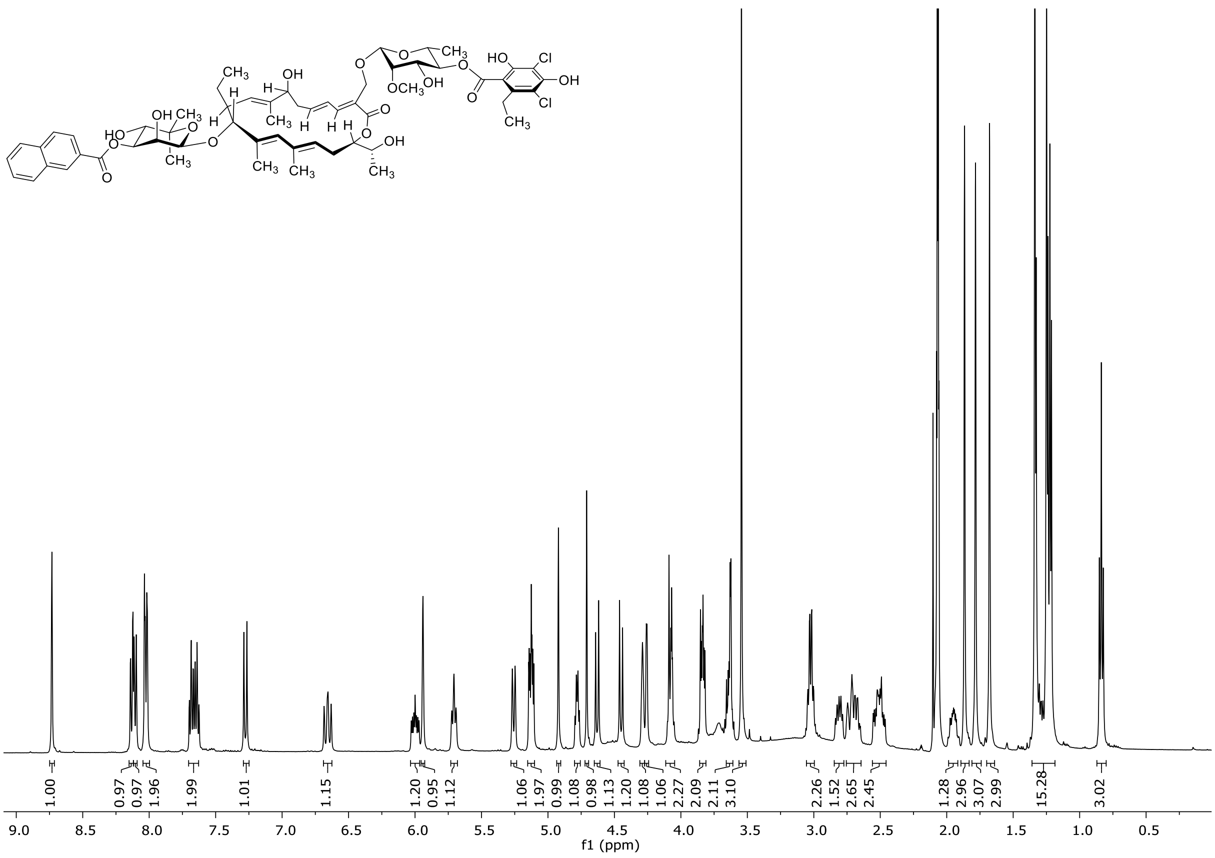

Supplementary Figure 24. ^{1}H NMR (500 MHz, acetone-d_6) of **7f**.

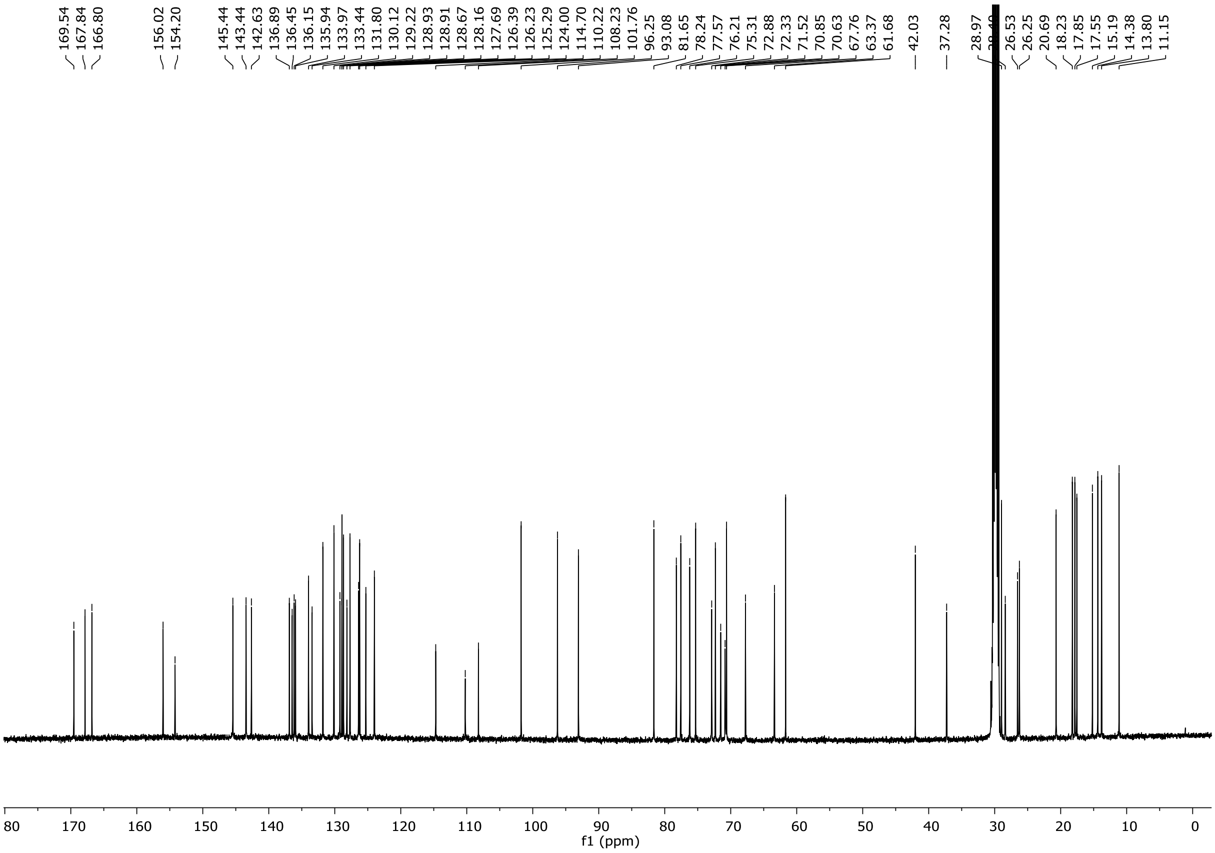

Supplementary Figure 25. ^{13}C NMR (126 MHz, acetone-d_6) of **7f.**

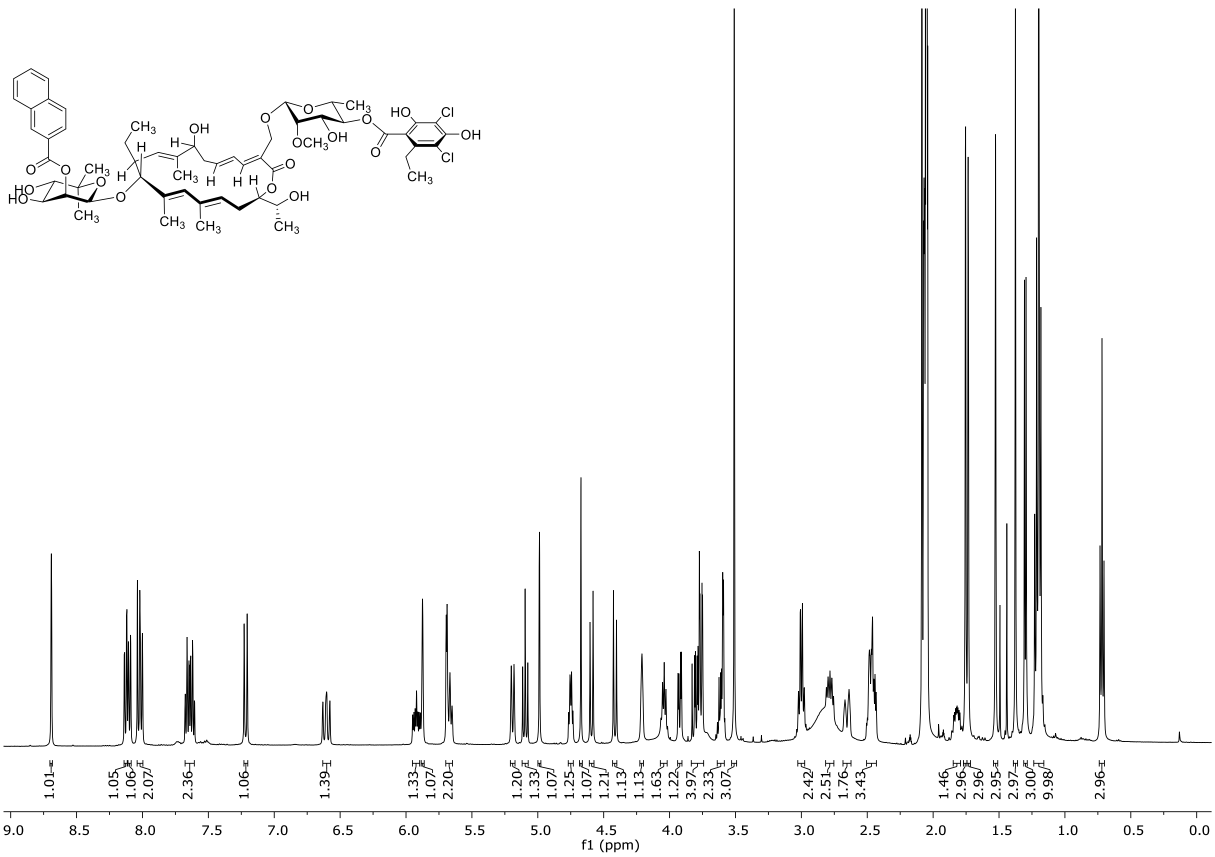

Supplementary Figure 26. ^{1}H NMR (500 MHz, acetone-d_6) of **7f-C2**.

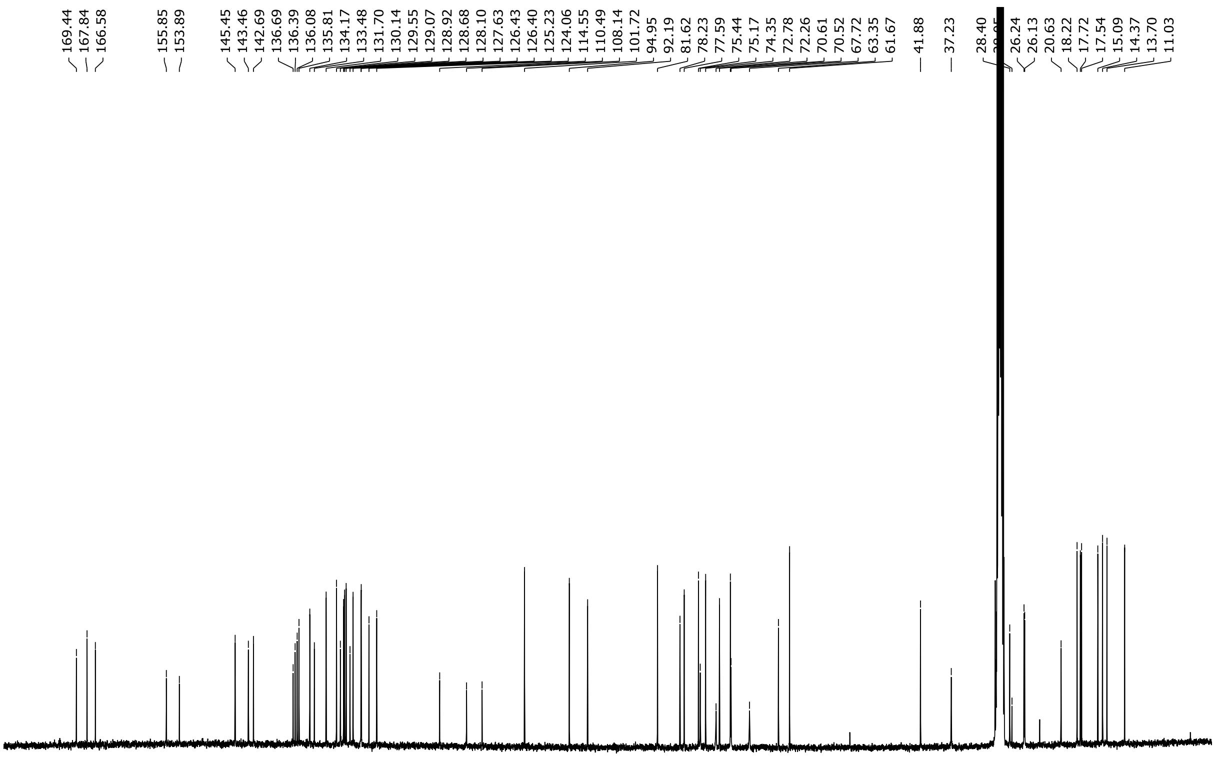

Supplementary Figure 27. ^{13}C NMR (126 MHz, acetone-d_6) of **7f-C2.**

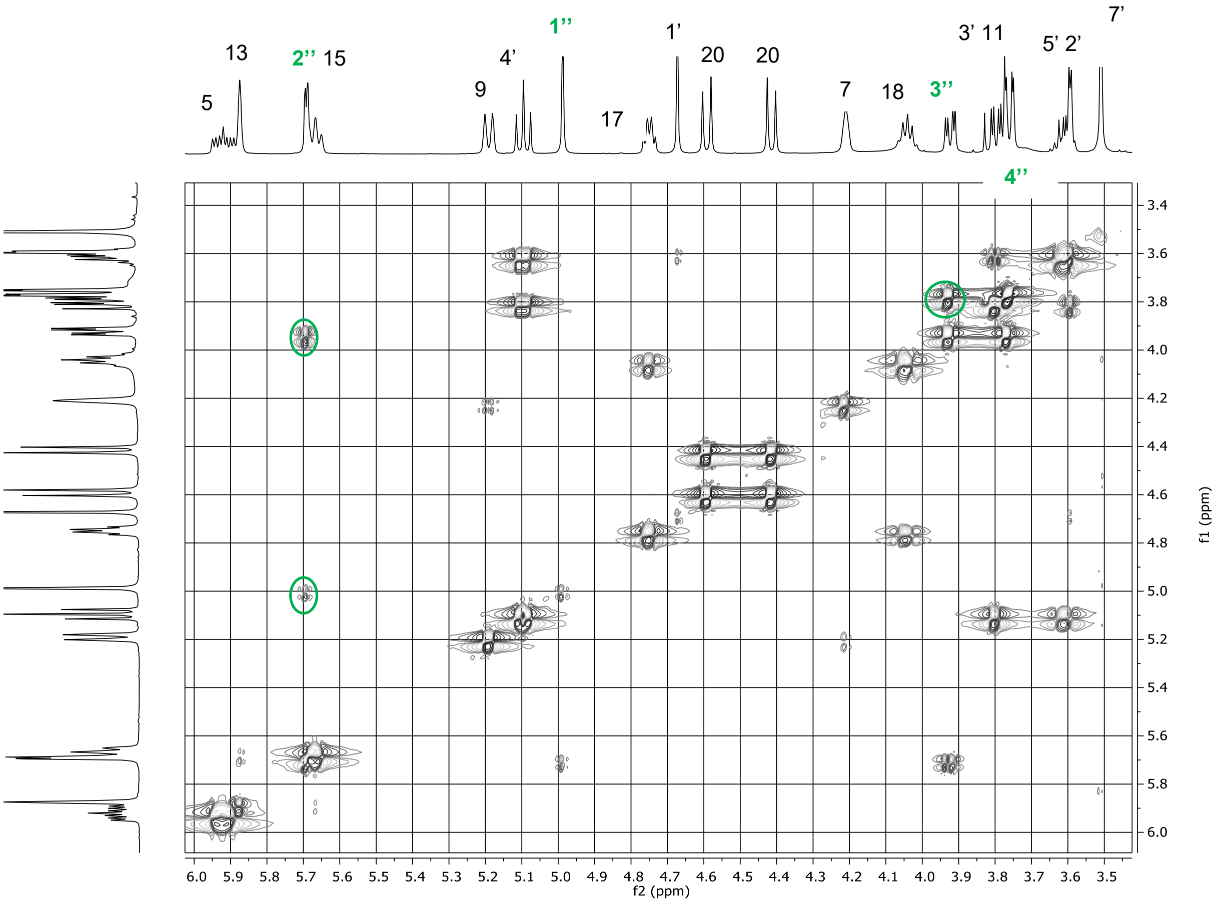

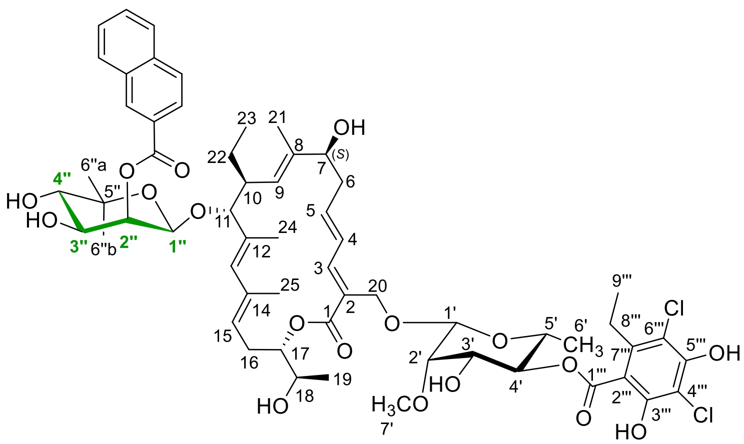

Supplementary Figure 28. COSY NMR (126 MHz, acetone-d_6) of **7f-C2.**

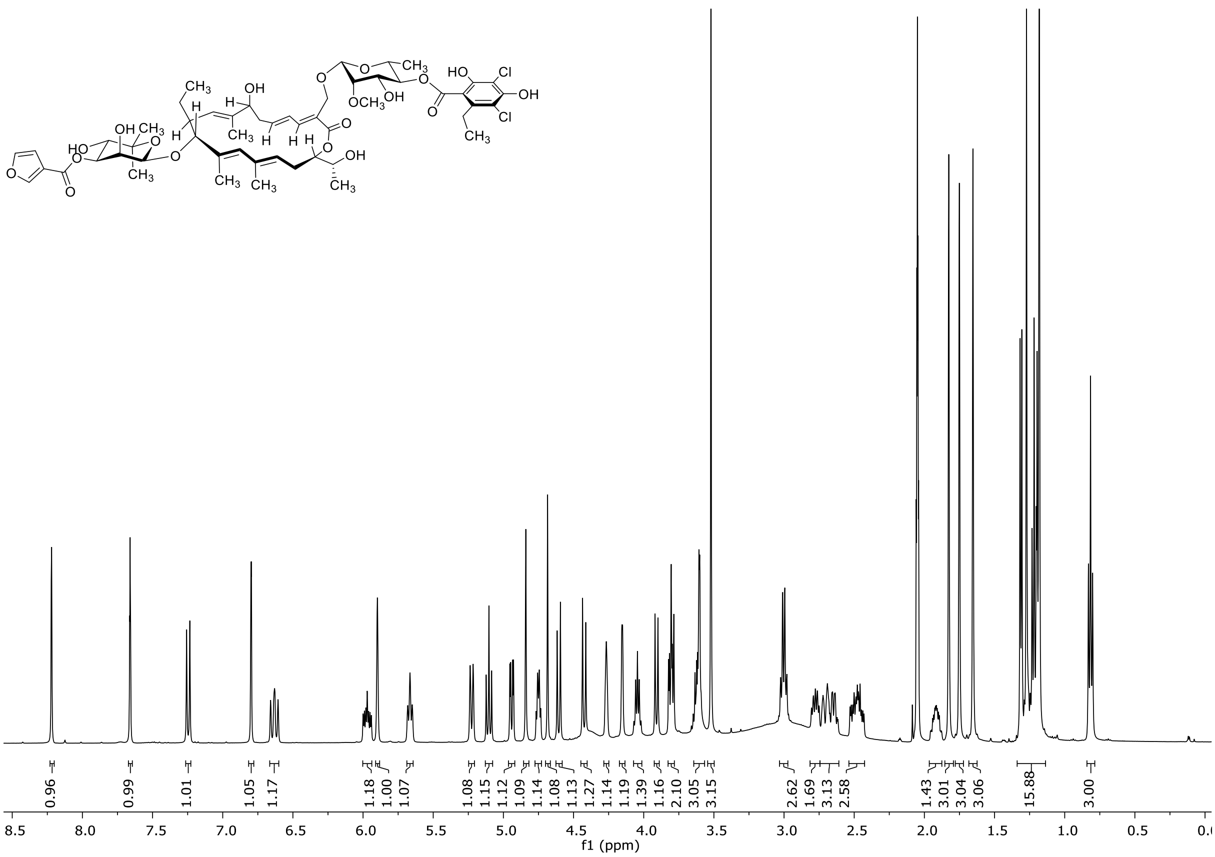

Supplementary Figure 29. ^{1}H NMR (500 MHz, acetone-d_6) of **7j**.

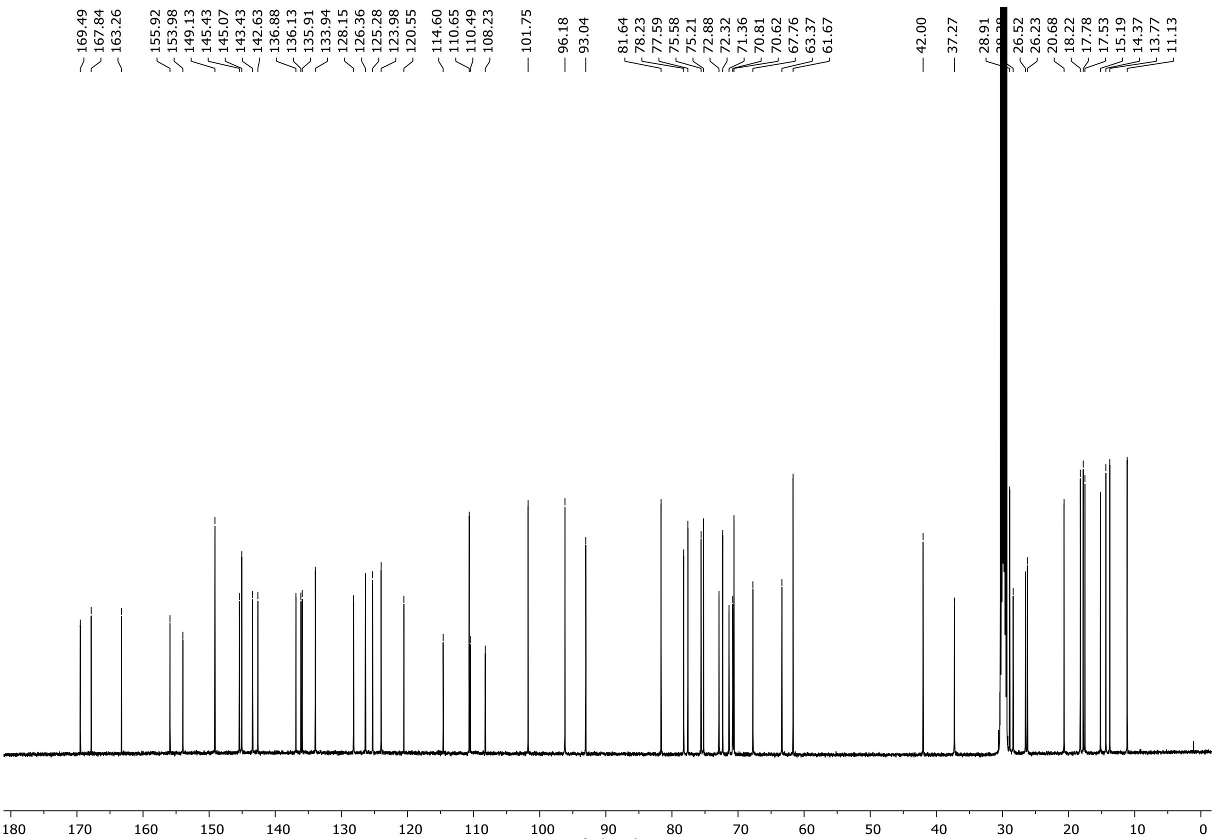

Supplementary Figure 30. ^{13}C NMR (126 MHz, acetone-d_6) of **7j.**

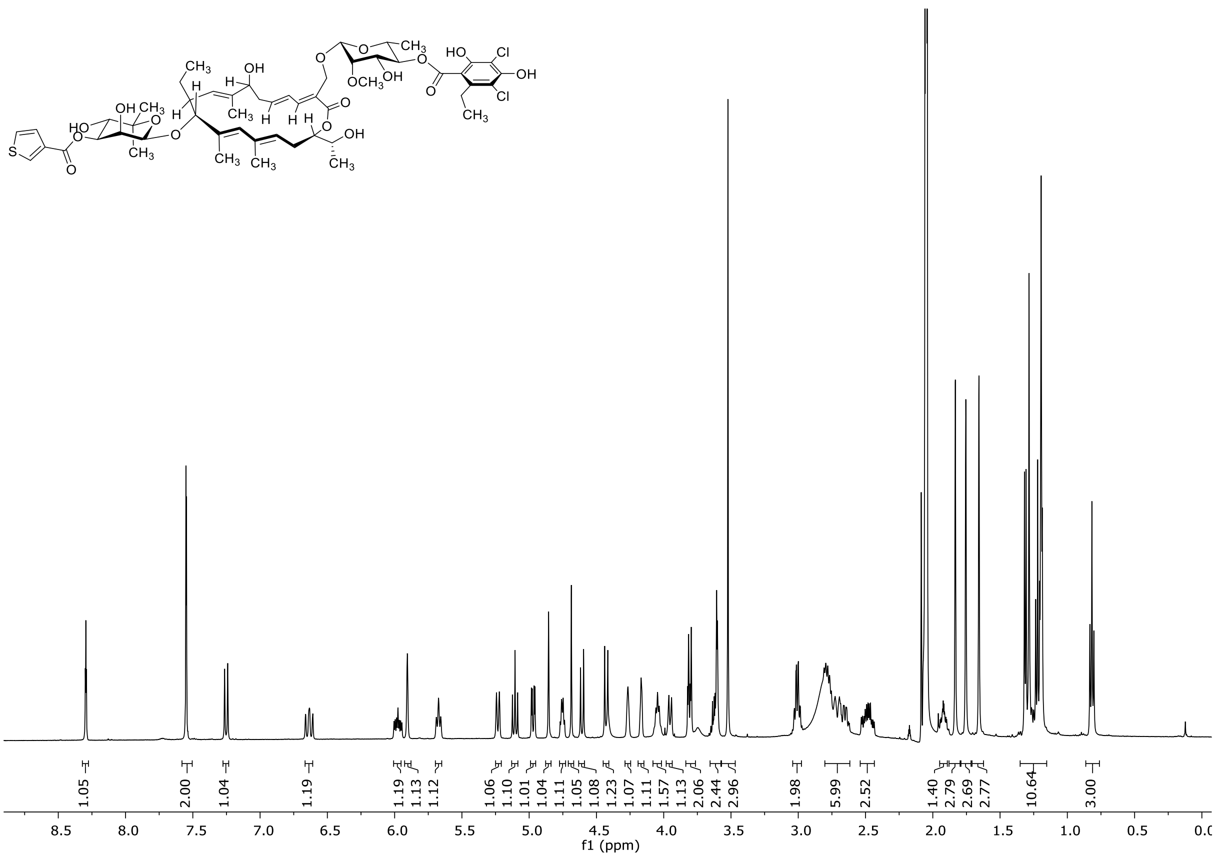

Supplementary Figure 31. ^{1}H NMR (500 MHz, acetone-*d₆*) of **7k.**

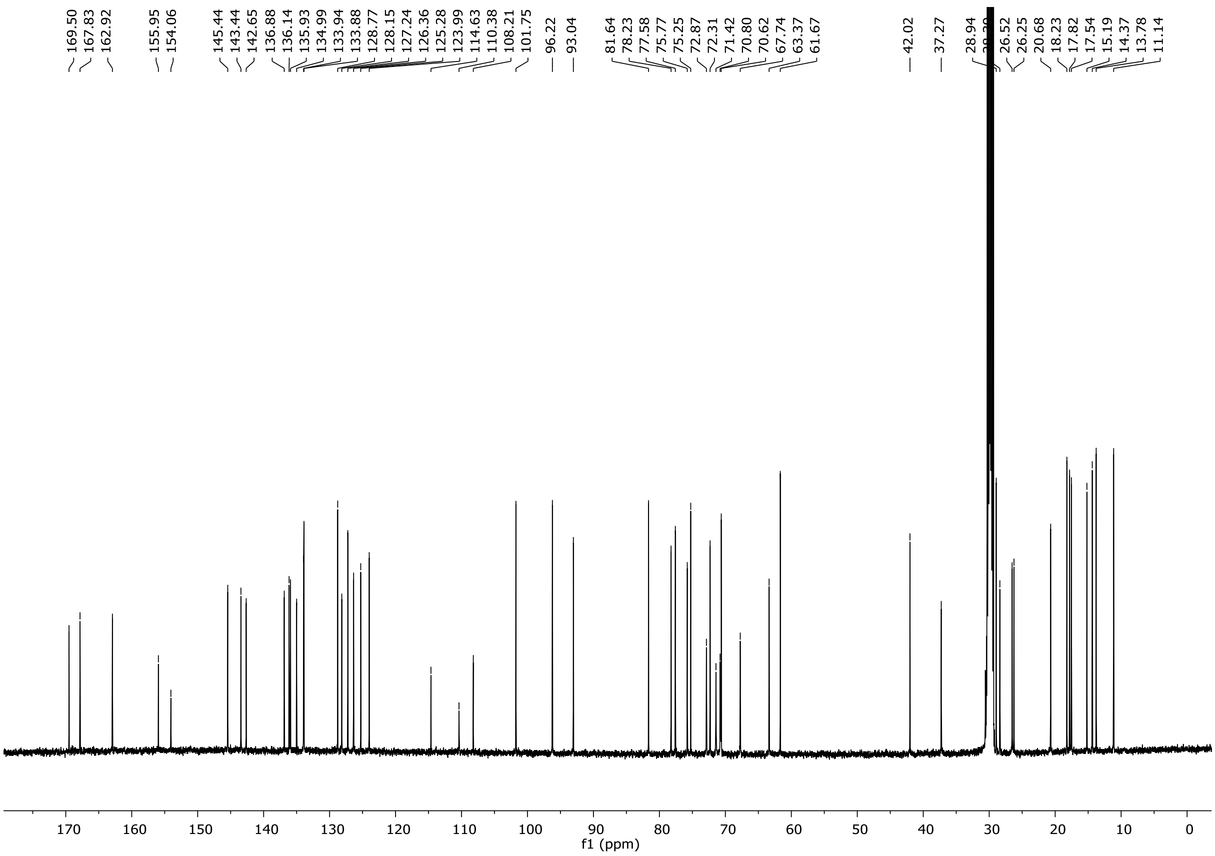

Supplementary Figure 32. ^{13}C NMR (126 MHz, acetone-d_6) of **7k.**

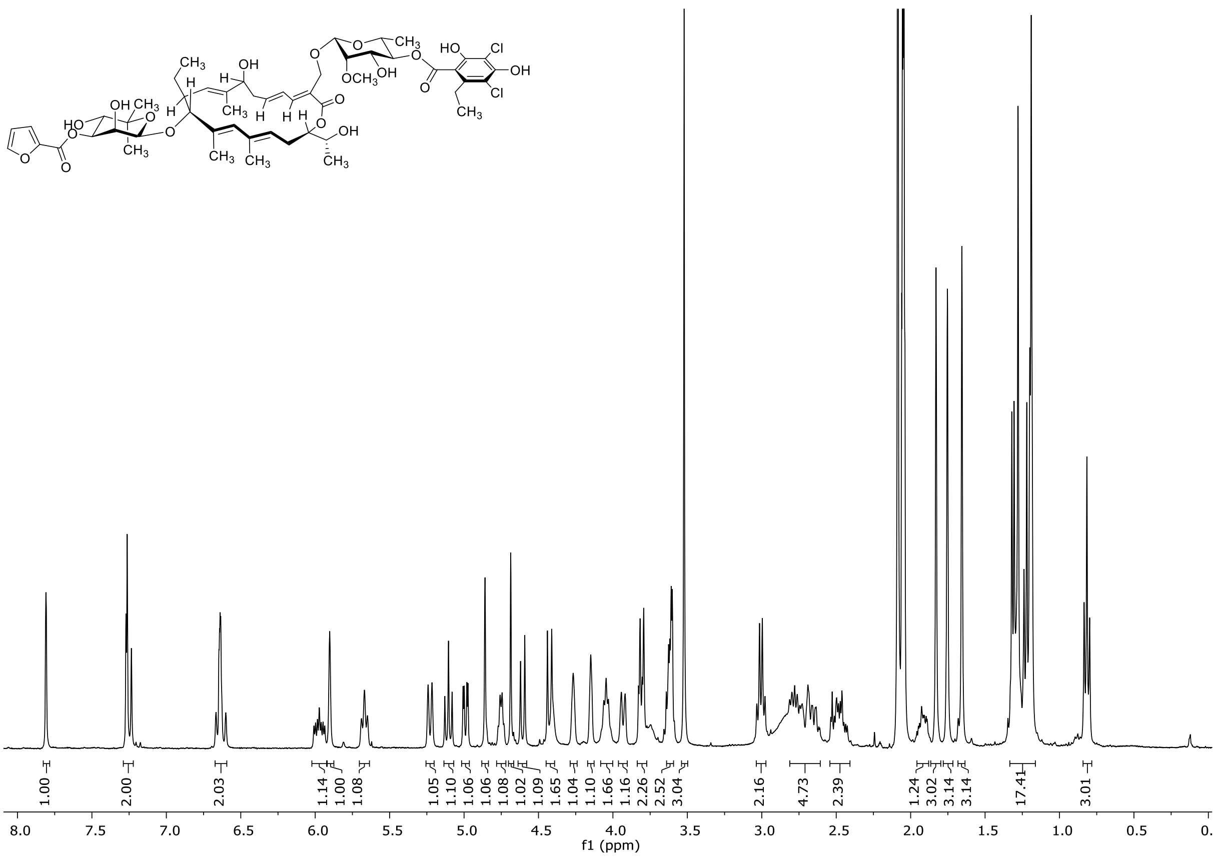

Supplementary Figure 33. ^{1}H NMR (400 MHz, acetone-d_6) of **7l.**

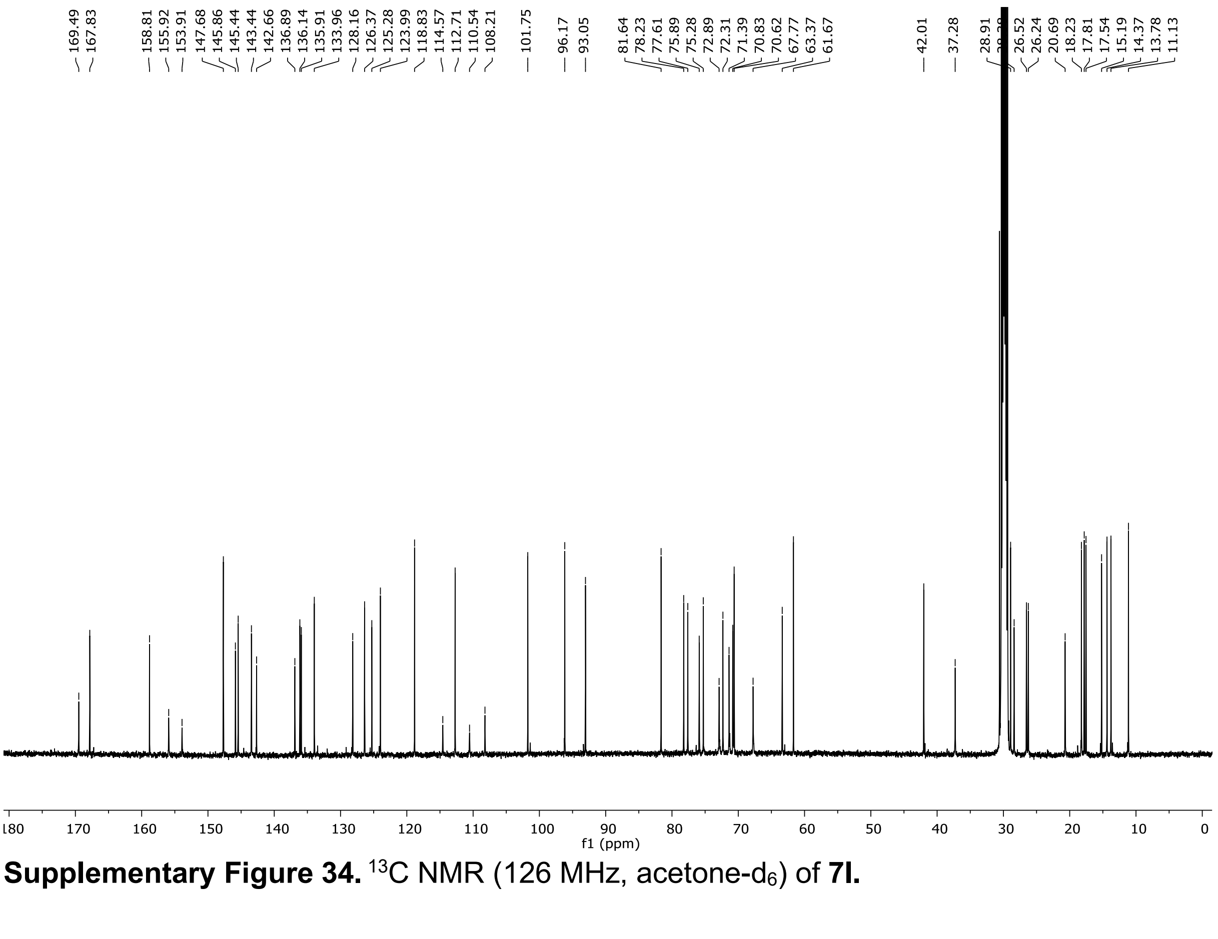

Supplementary Figure 34. ^{13}C NMR (126 MHz, acetone-d_6) of **7l.**

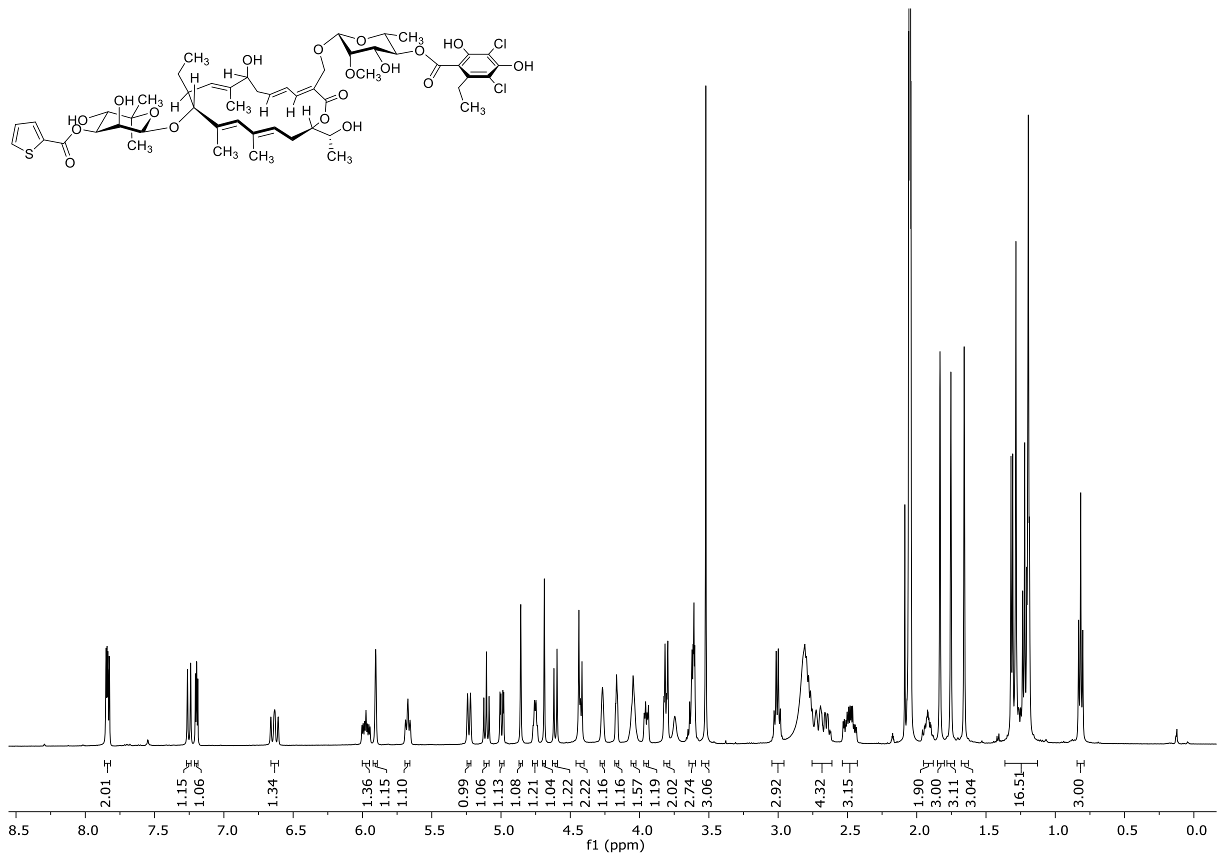

Supplementary Figure 35. ^{1}H NMR (500 MHz, acetone-d_6) of **7m.**

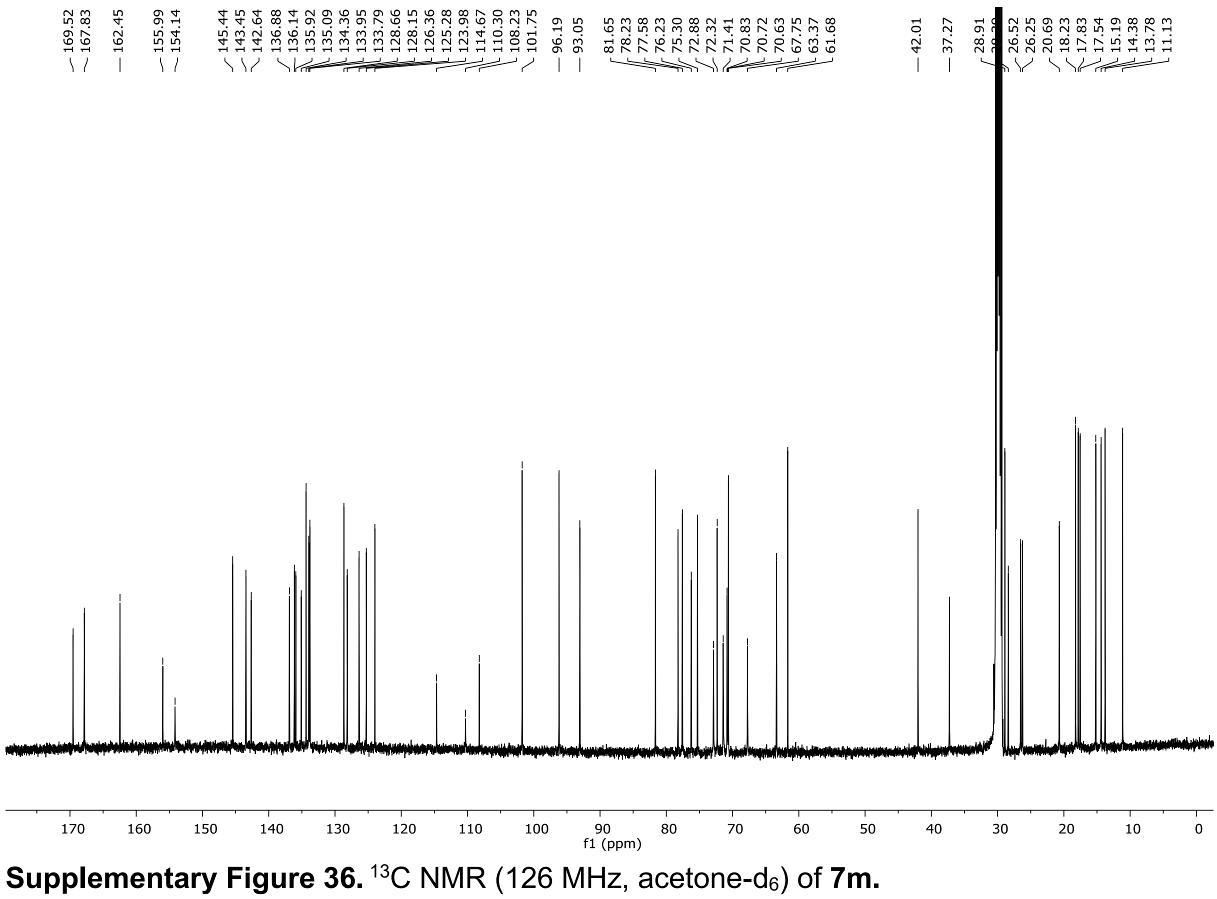

Supplementary Figure 36. ^{13}C NMR (126 MHz, acetone-d_6) of **7m.**

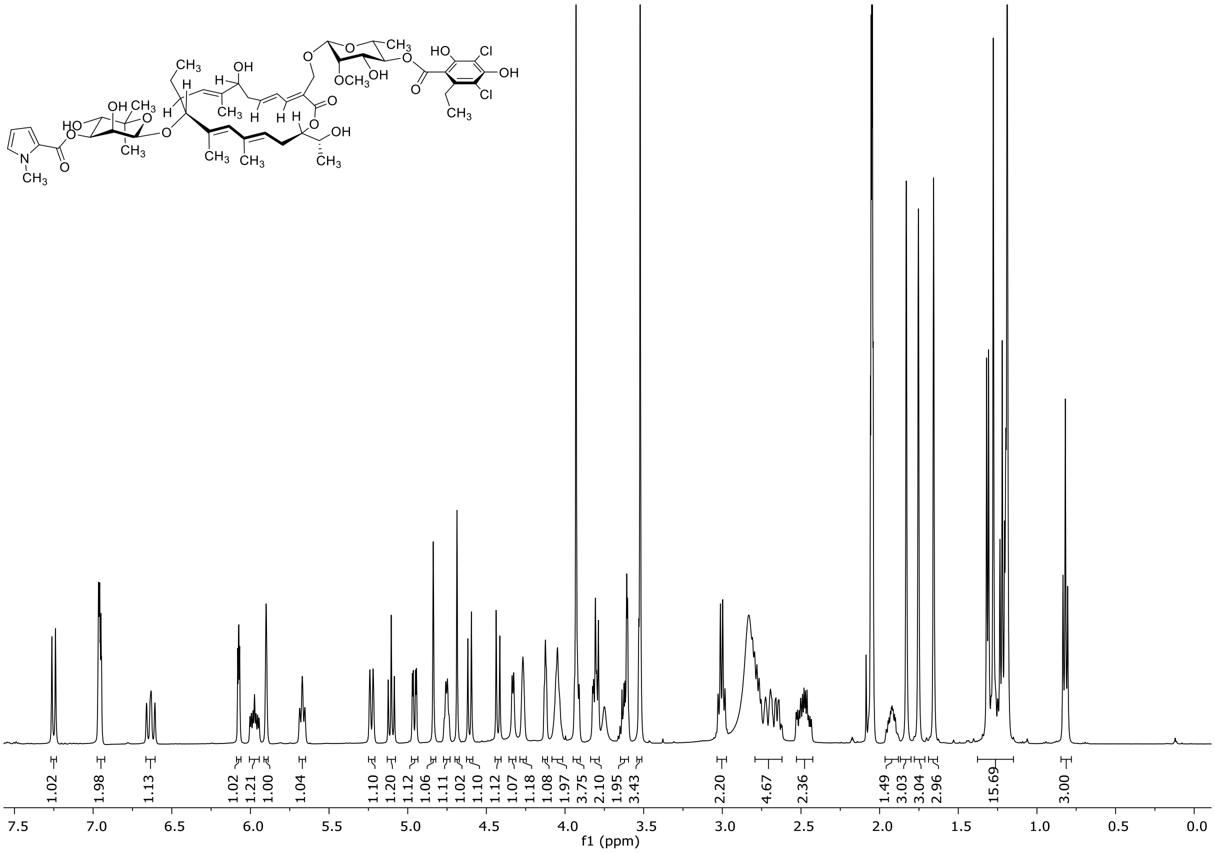

Supplementary Figure 37. ^{1}H NMR (500 MHz, acetone-d_6) of **7n.**

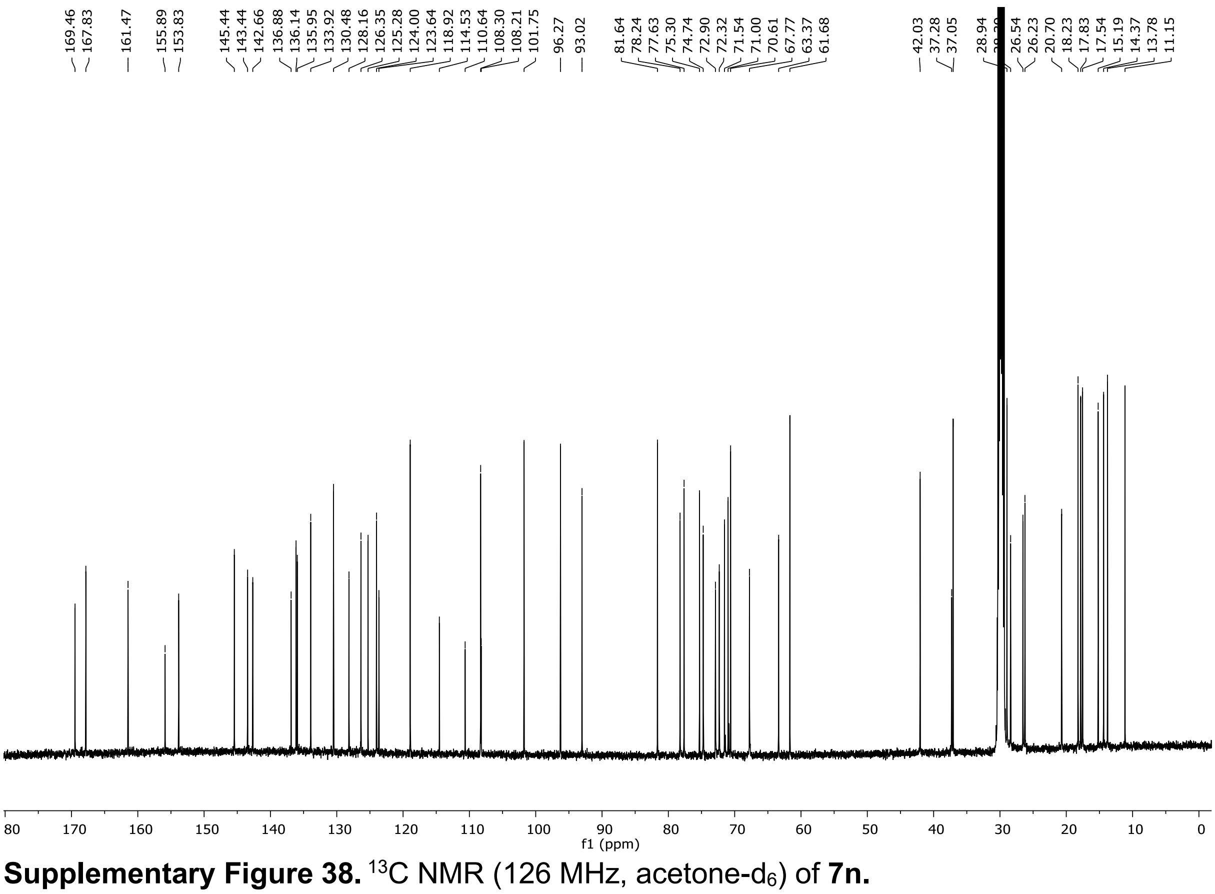

Supplementary Figure 38. ^{13}C NMR (126 MHz, acetone-d_6) of **7n.**

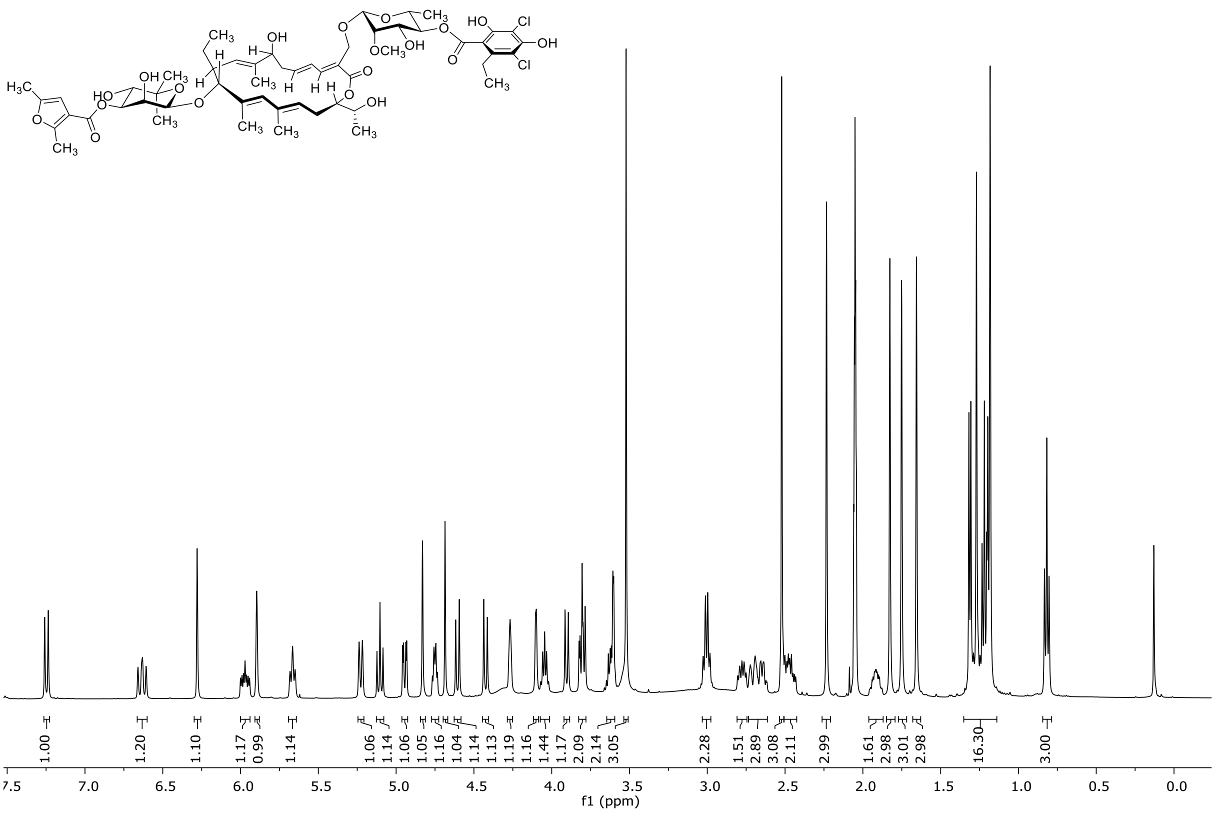

Supplementary Figure 39. ^{1}H NMR (500 MHz, acetone-d_6) of **7p**.

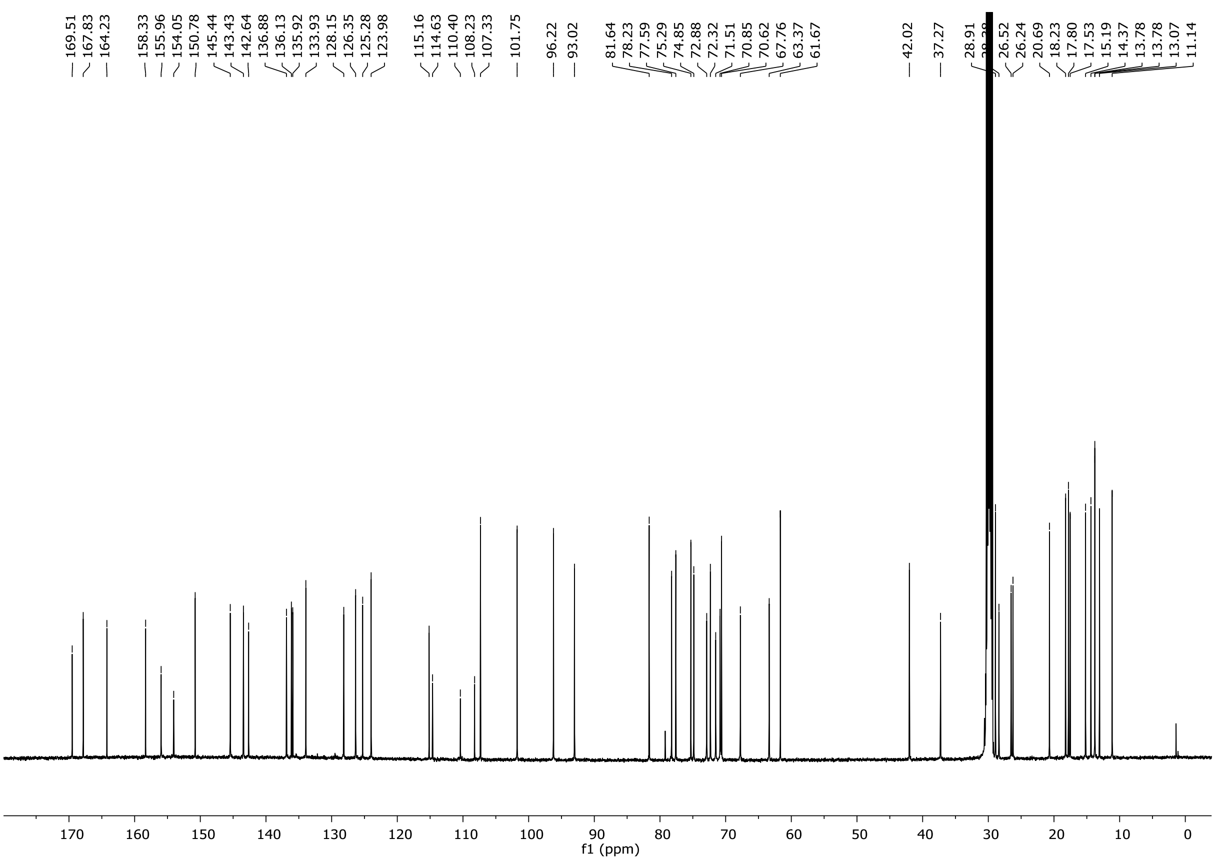

Supplementary Figure 40. ^{13}C NMR (126 MHz, acetone-d_6) of **7p**.

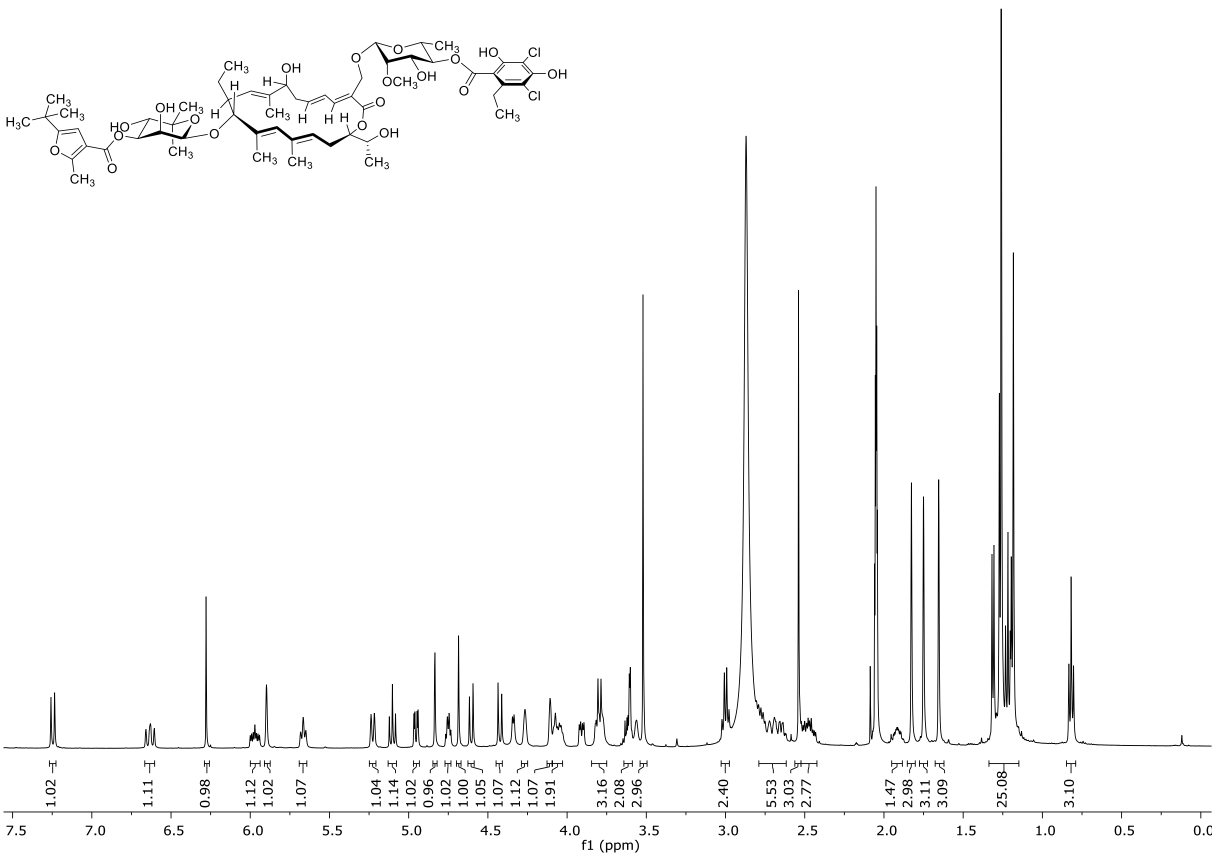

Supplementary Figure 41. ^{1}H NMR (500 MHz, acetone-d_6) of **7q**.

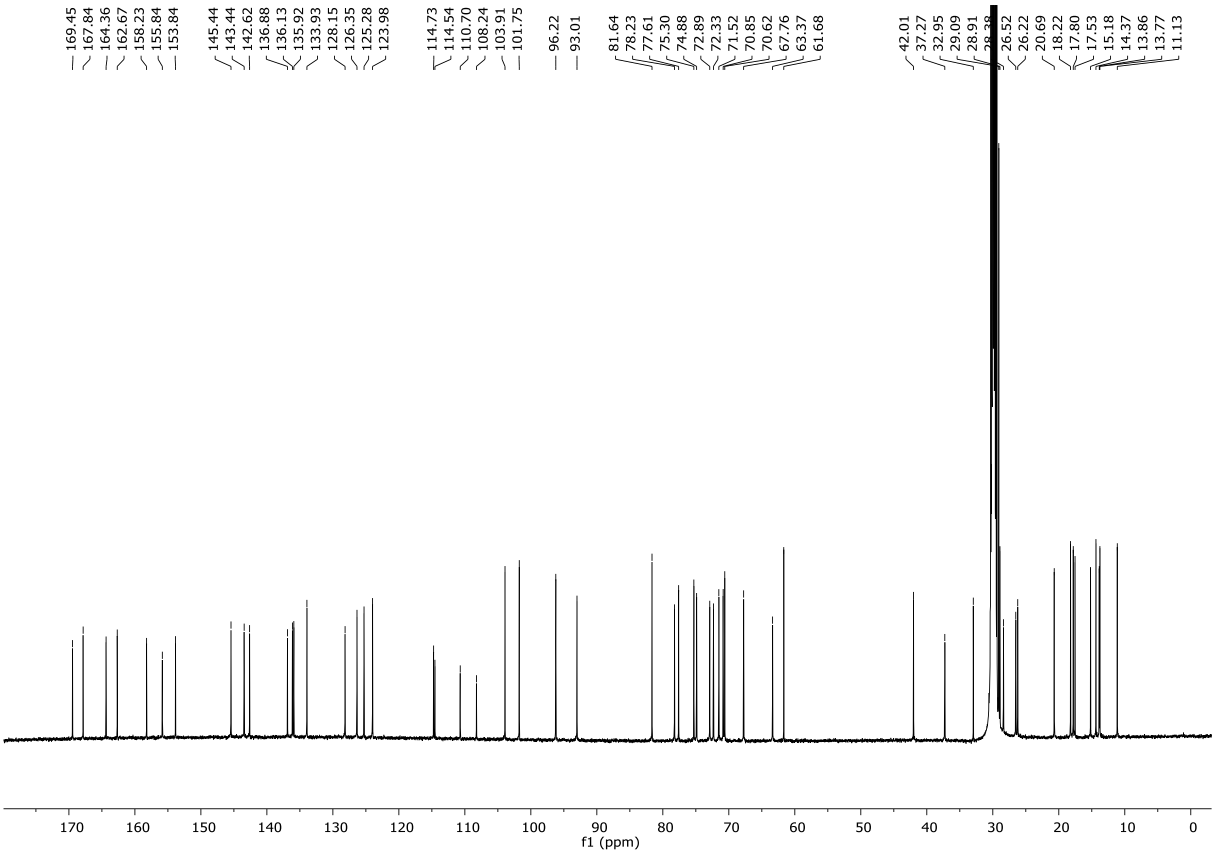

Supplementary Figure 42. ^{13}C NMR (126 MHz, acetone-d_6) of **7q**.

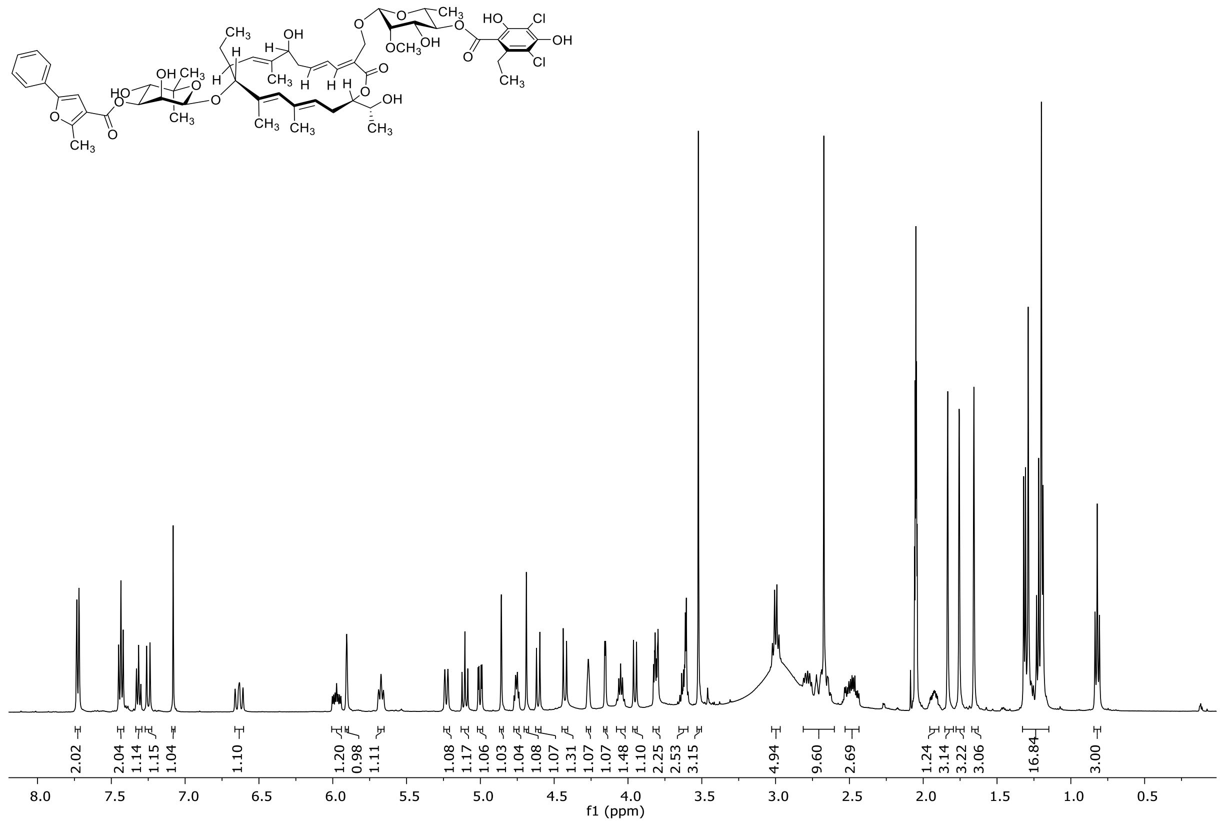

Supplementary Figure 43. ^{1}H NMR (500 MHz, acetone-d_6) of **7r**.

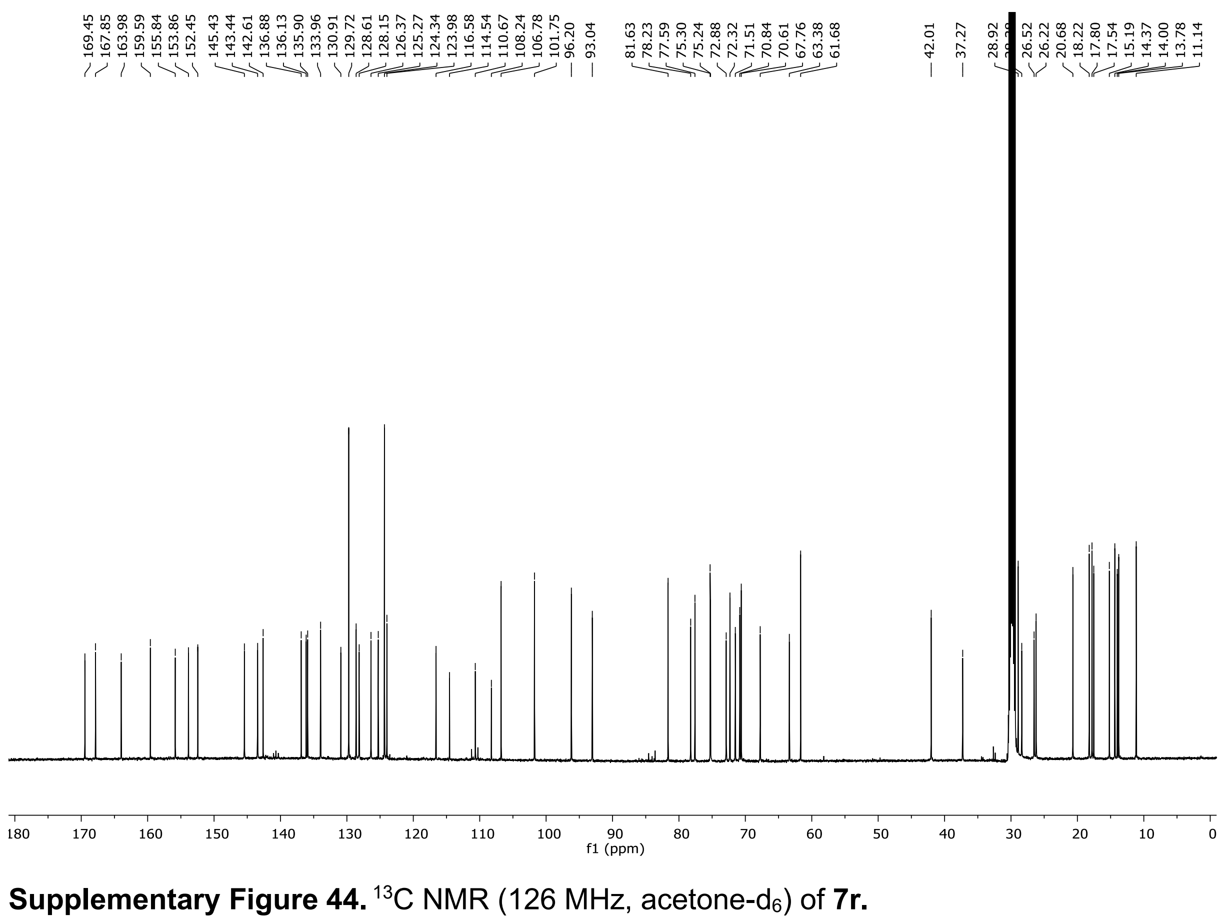

Supplementary Figure 44. ^{13}C NMR (126 MHz, acetone-d_6) of **7r**.

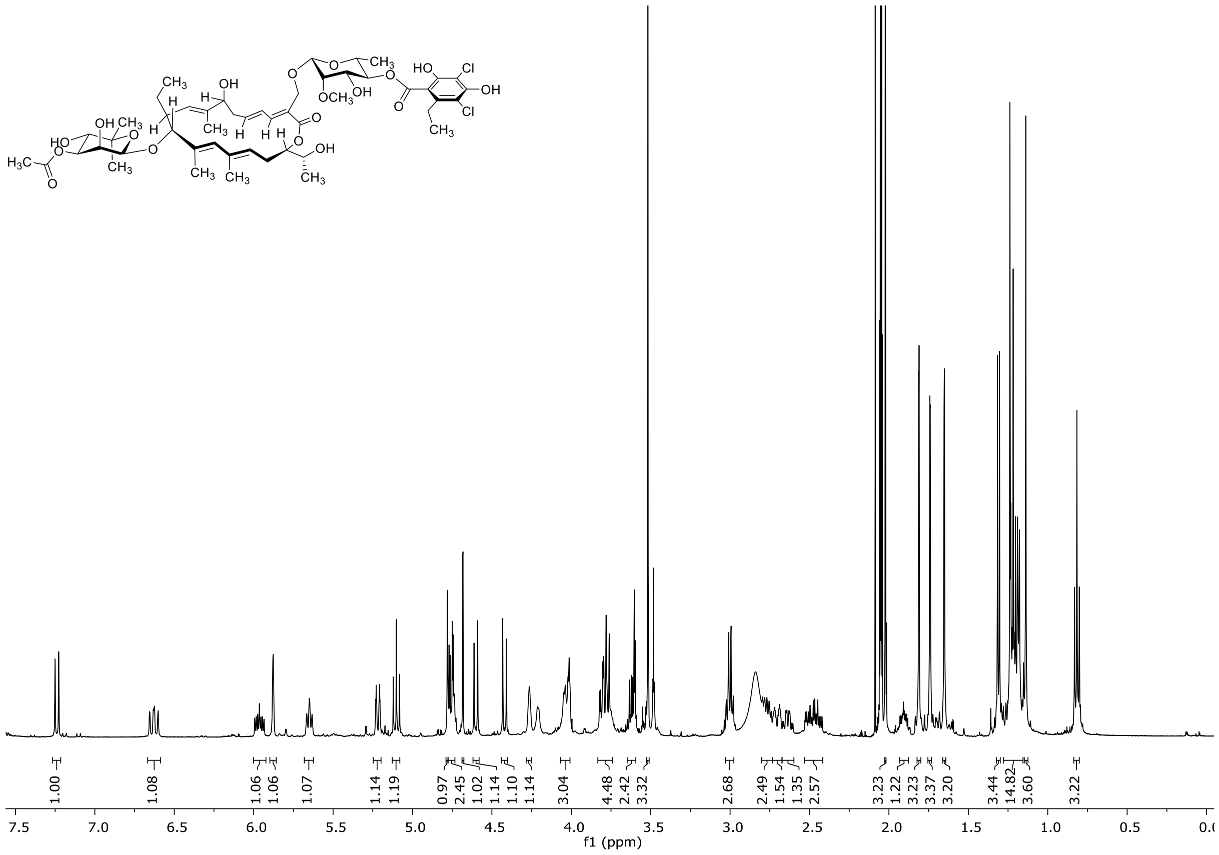

Supplementary Figure 45. ^{1}H NMR (500 MHz, acetone-*d6*) of **7s**.

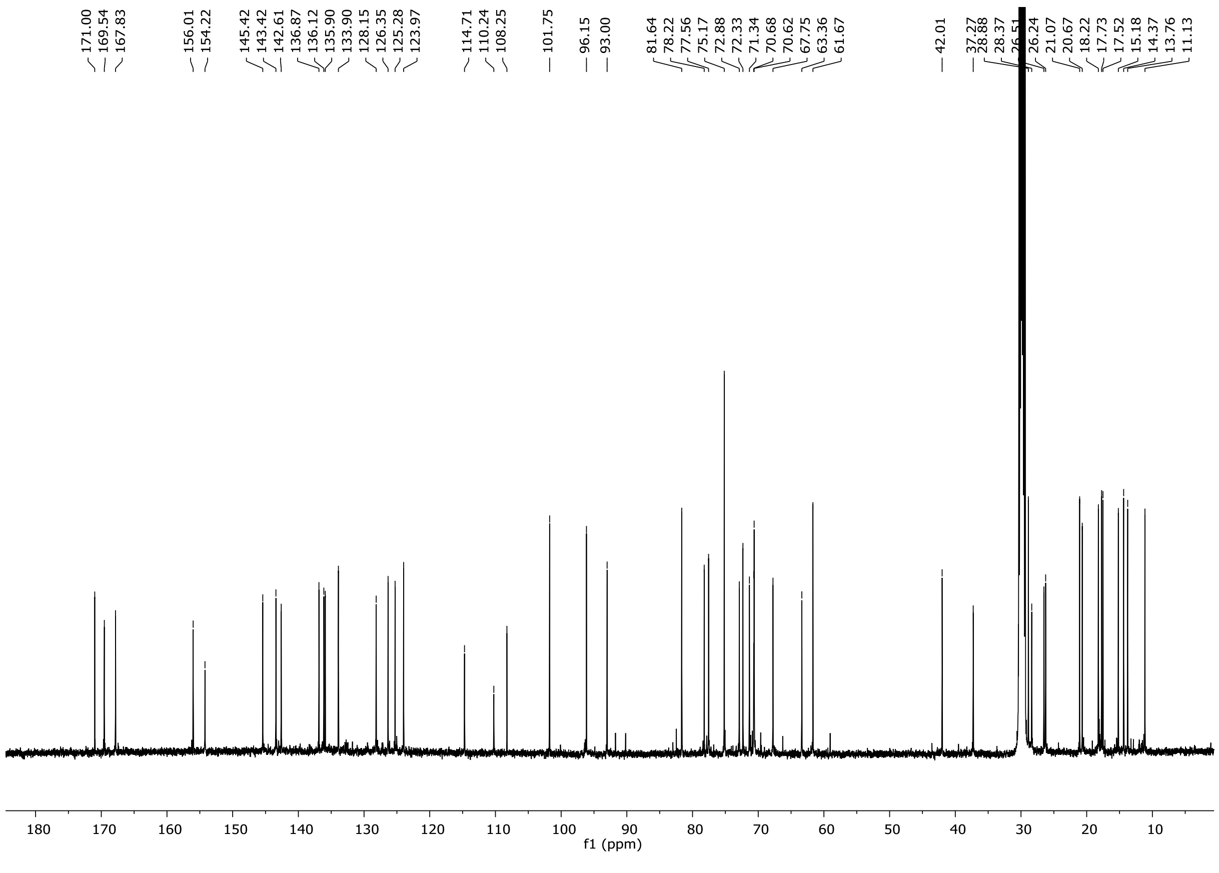

Supplementary Figure 46. ^{13}C NMR (126 MHz, acetone-d_6) of **7s.**

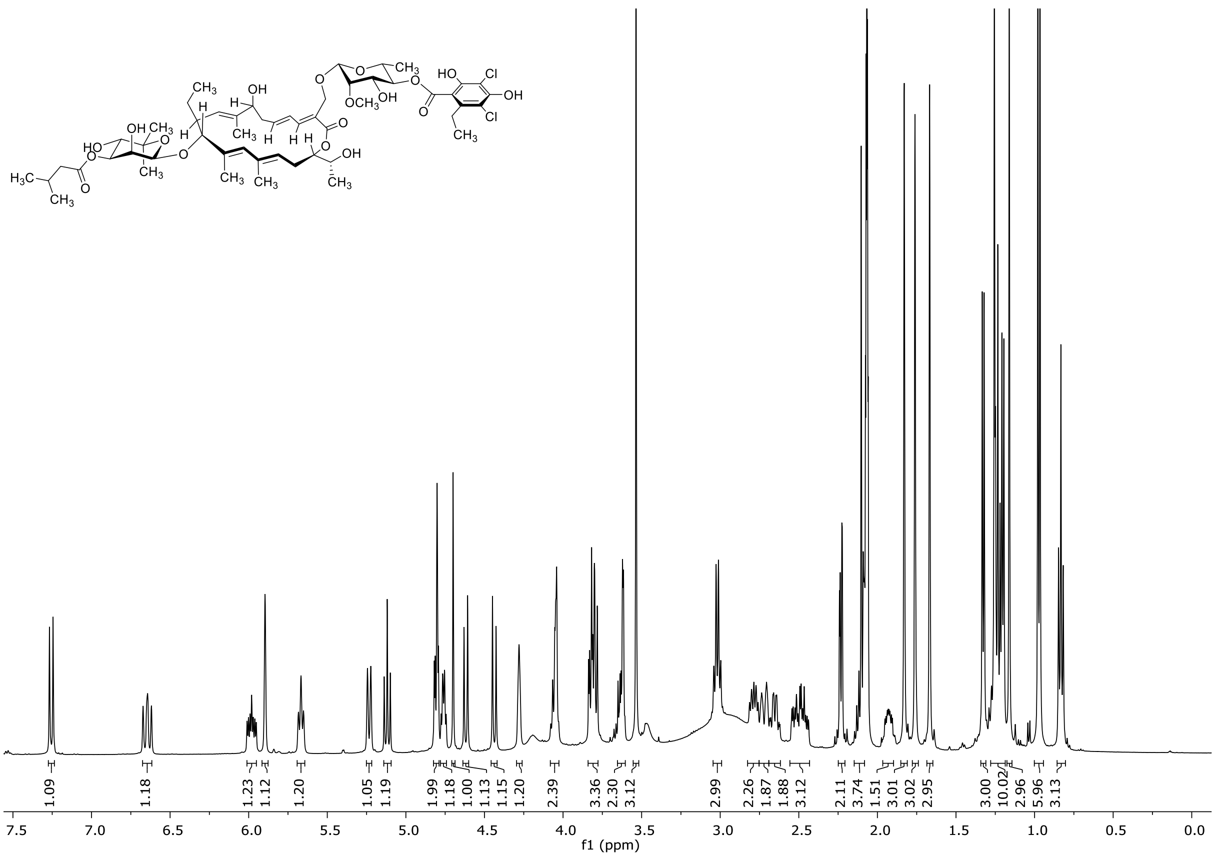

Supplementary Figure 47. ^{1}H NMR (500 MHz, acetone-d_6) of **7t**.

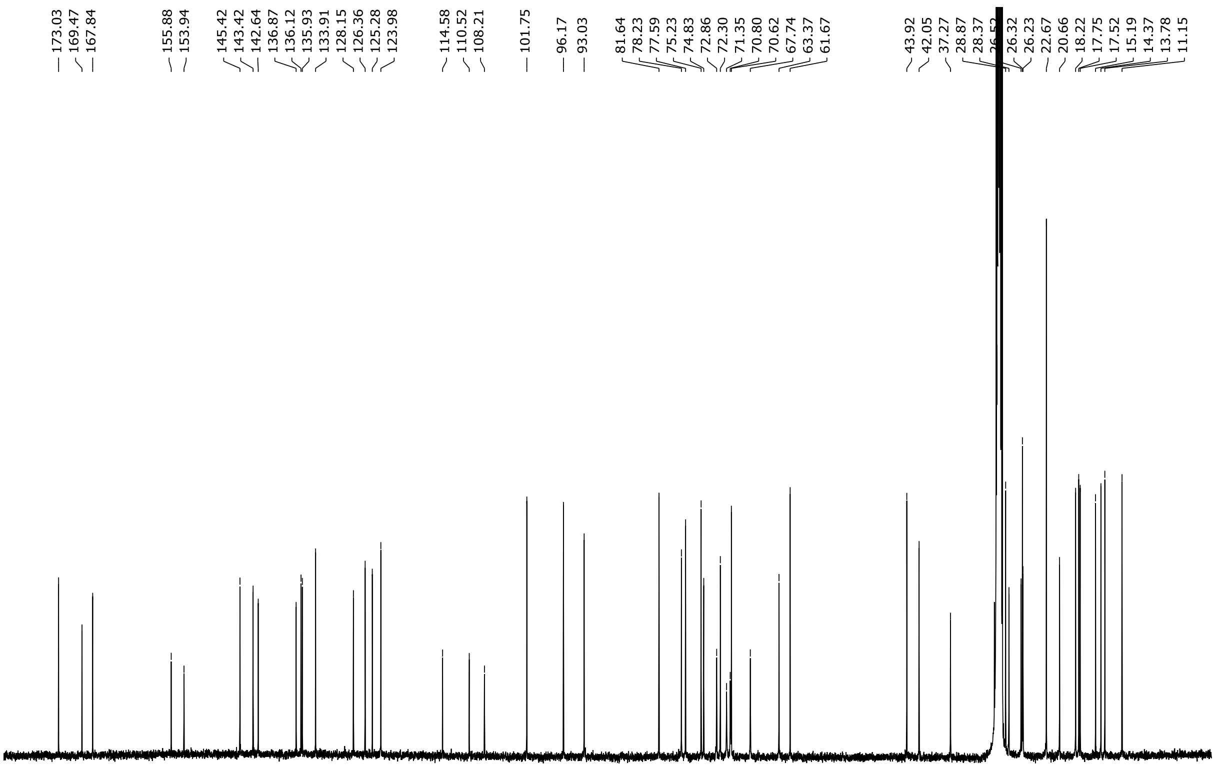

Supplementary Figure 48. ^{13}C NMR (126 MHz, acetone-d_6) of **7t.**

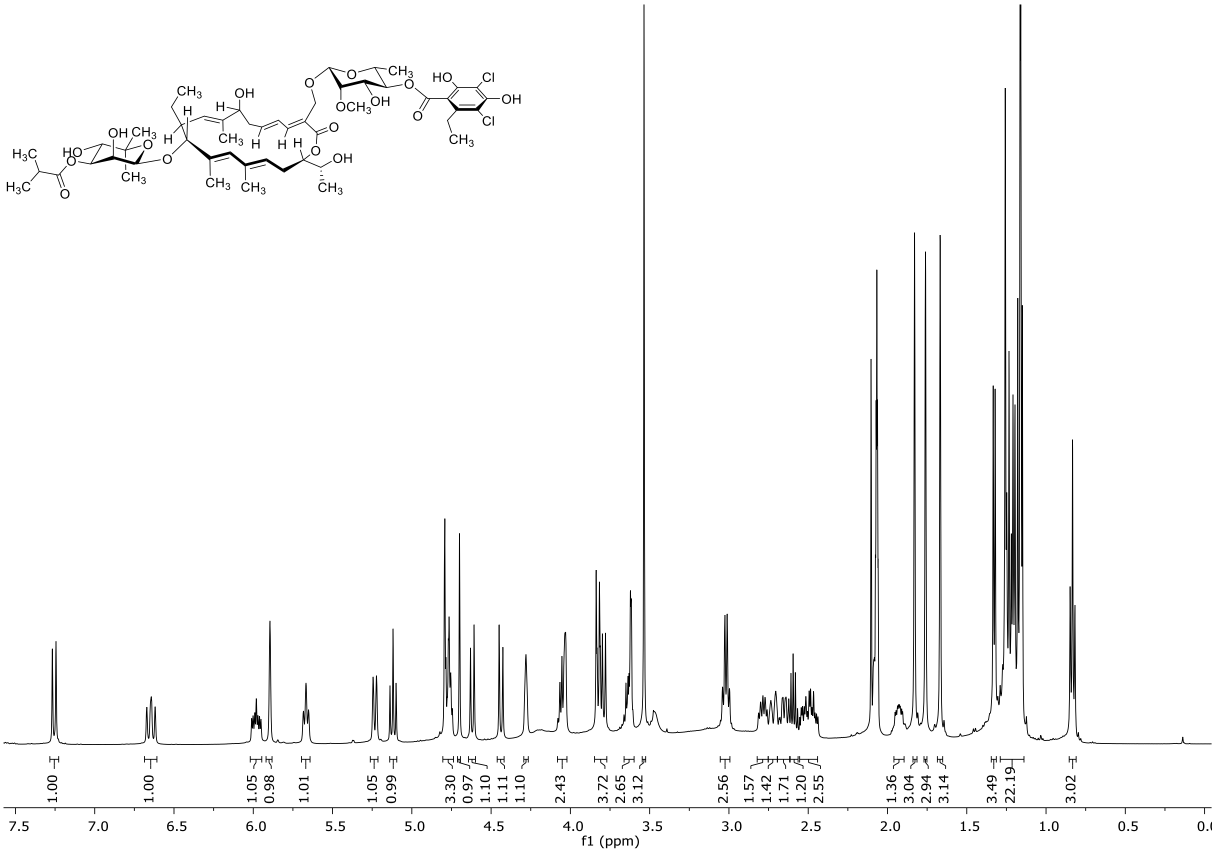

Supplementary Figure 49. ^{1}H NMR (500 MHz, acetone-*d$_6$*) of **7u**.

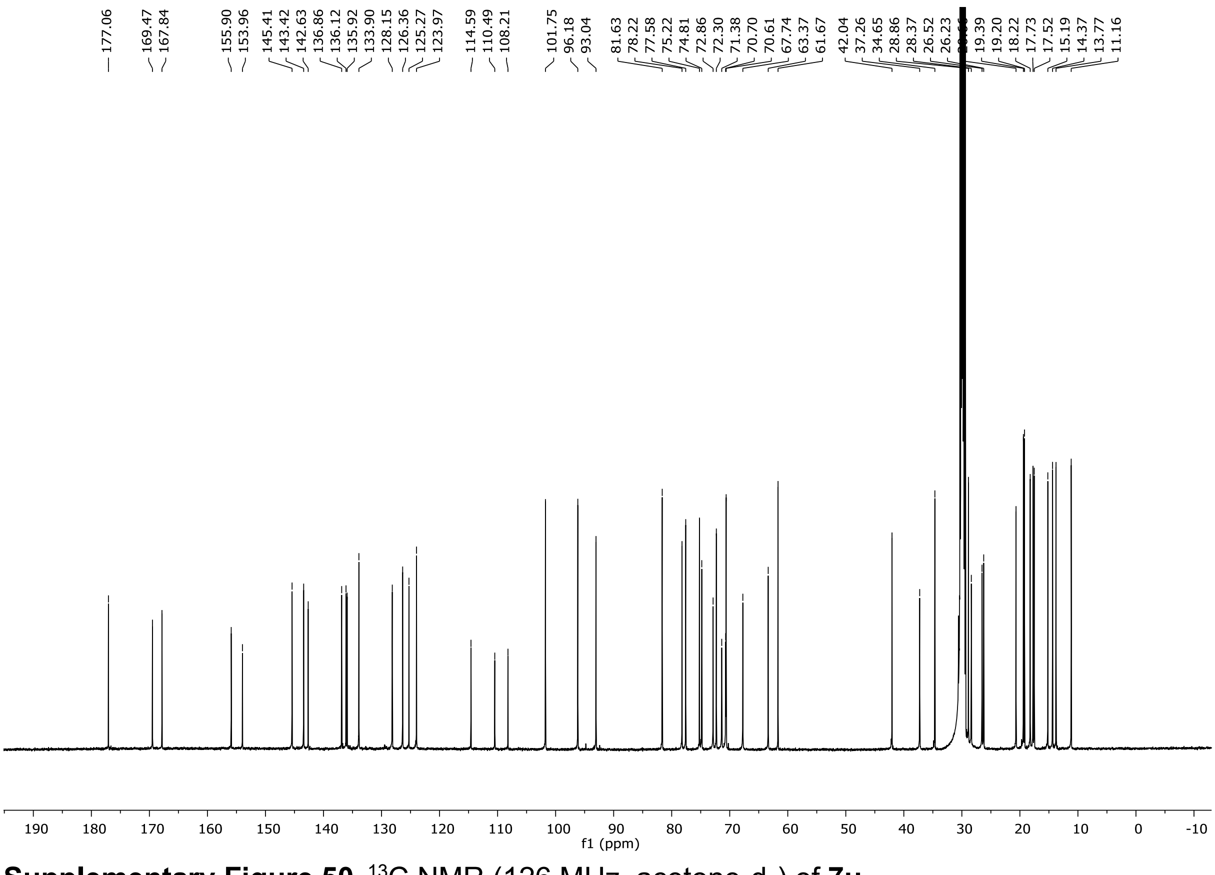

Supplementary Figure 50. ^{13}C NMR (126 MHz, acetone-d$_6$) of **7u.**

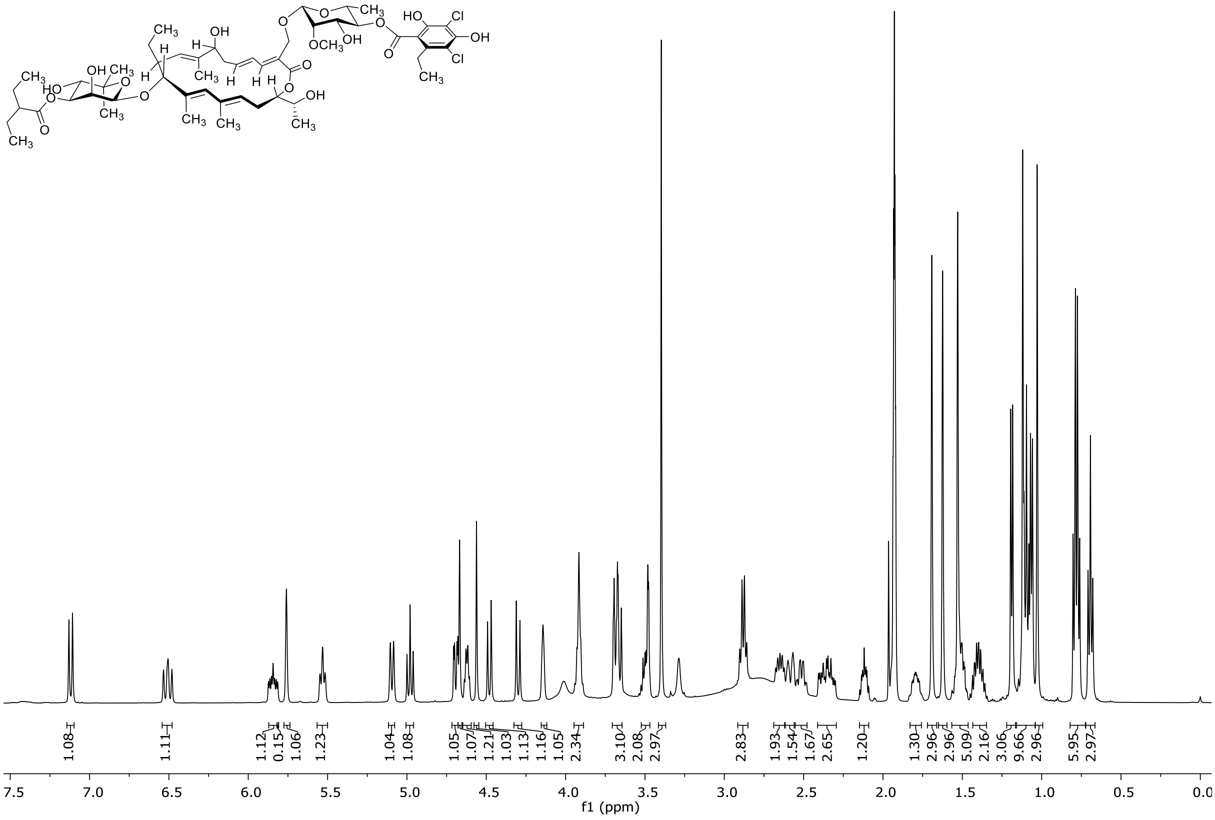

Supplementary Figure 51. ^{1}H NMR (500 MHz, acetone-d_6) of **7w**.

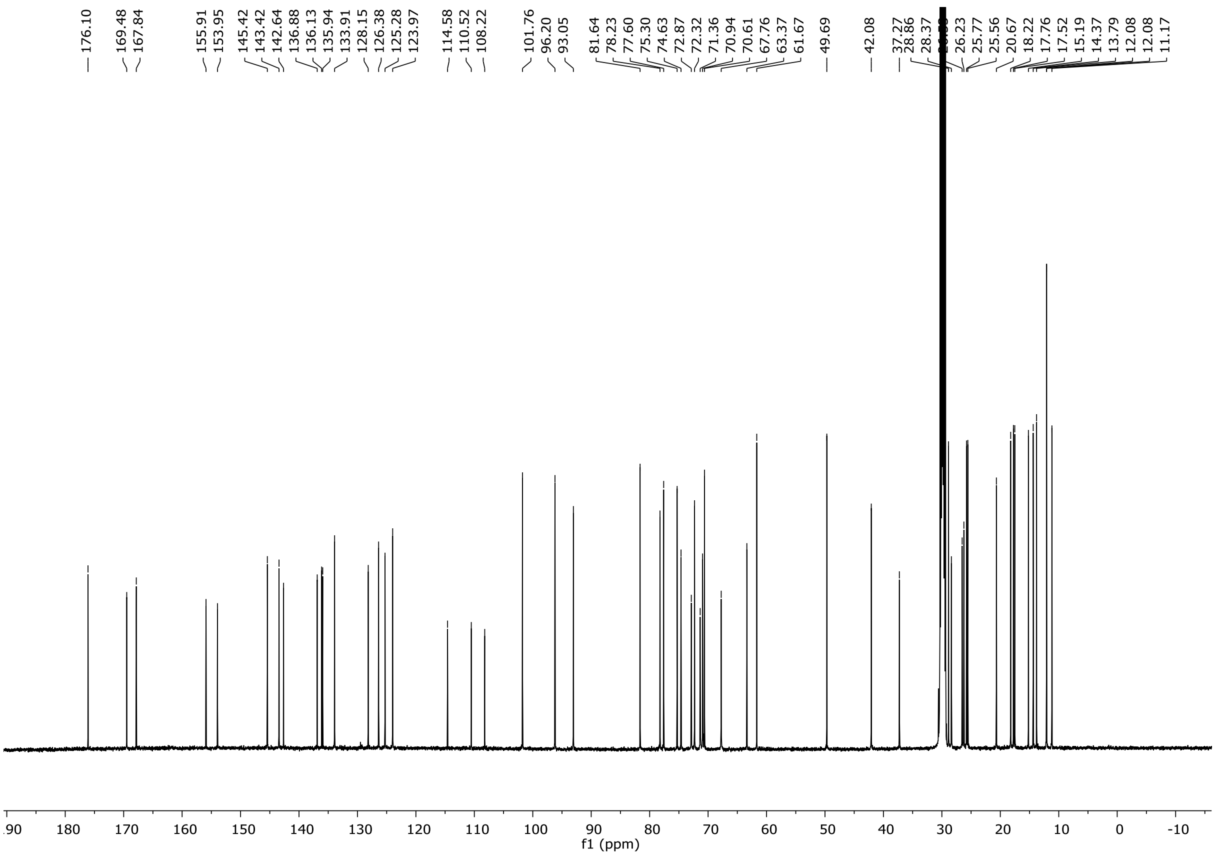

Supplementary Figure 52. ^{13}C NMR (126 MHz, acetone-d$_6$) of **7w.**

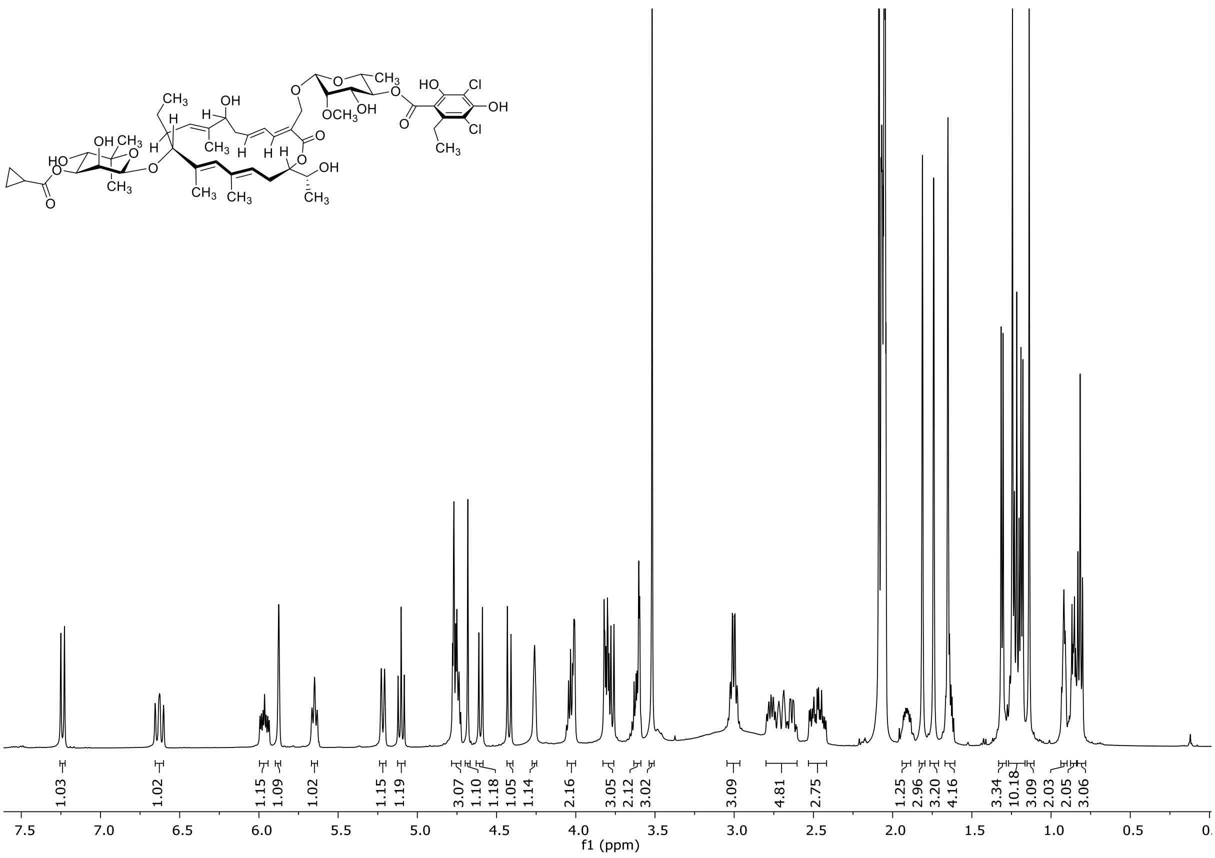

Supplementary Figure 53. ^{1}H NMR (500 MHz, acetone-*d*$_6$) of **7x.**

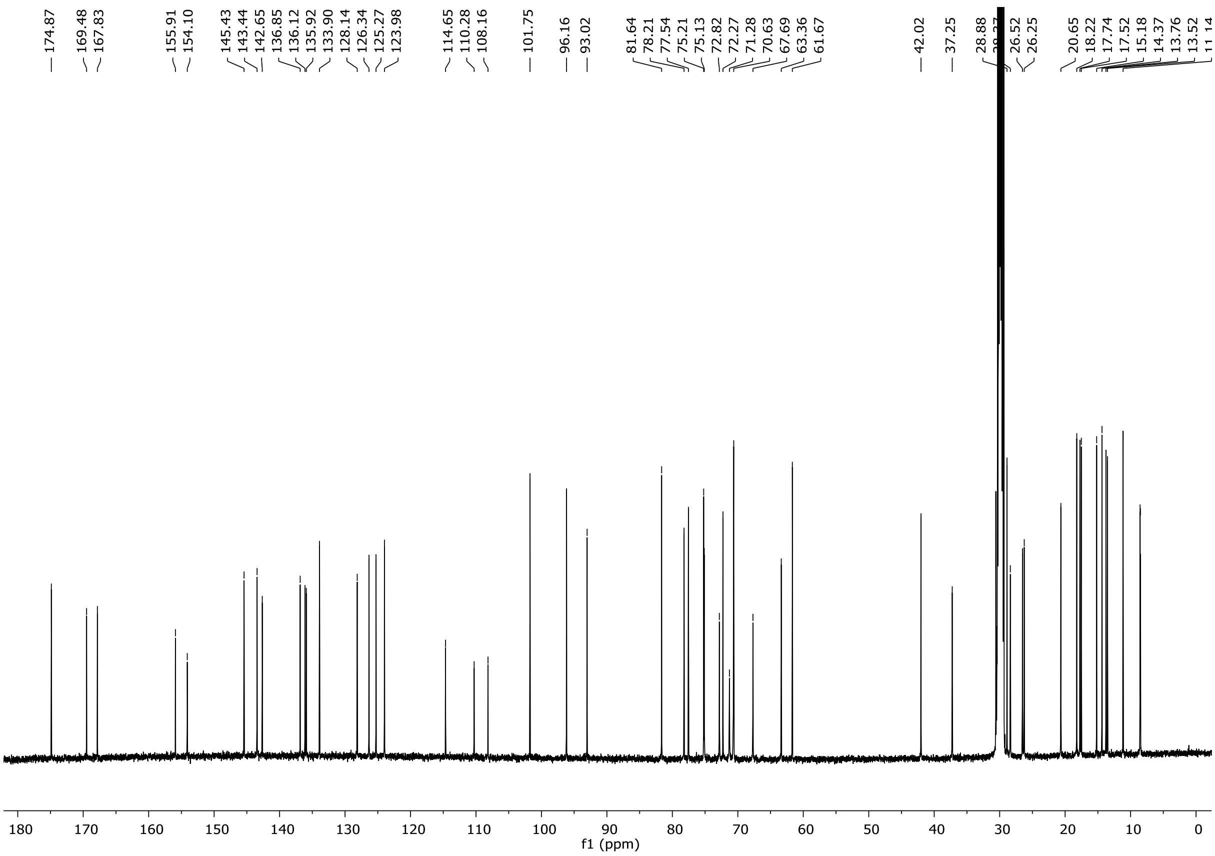

Supplementary Figure 54. ^{13}C NMR (126 MHz, acetone-d$_6$) of **7x.**

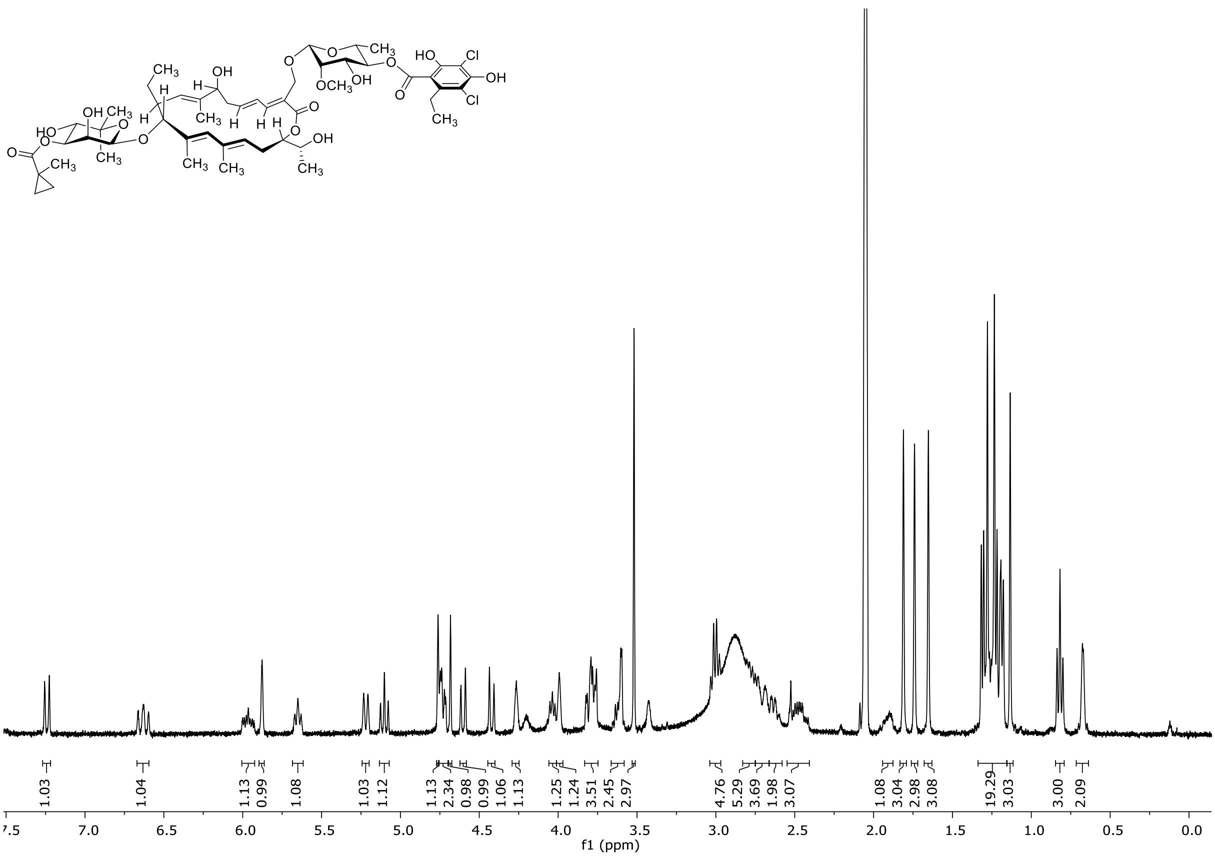

Supplementary Figure 55. ^{1}H NMR (500 MHz, acetone-d_6) of **7y.**

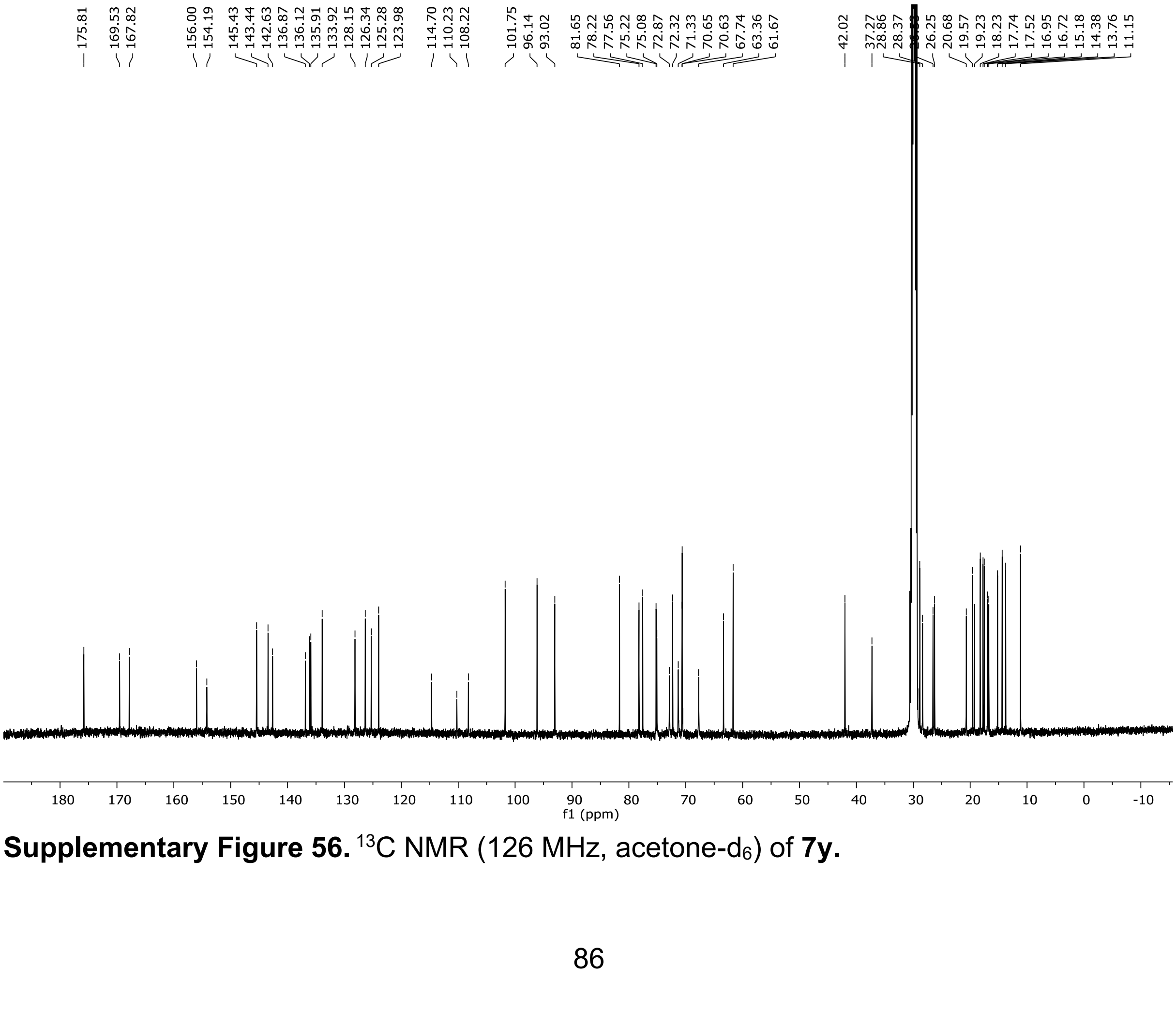

Supplementary Figure 56. ^{13}C NMR (126 MHz, acetone-d_6) of **7y.**

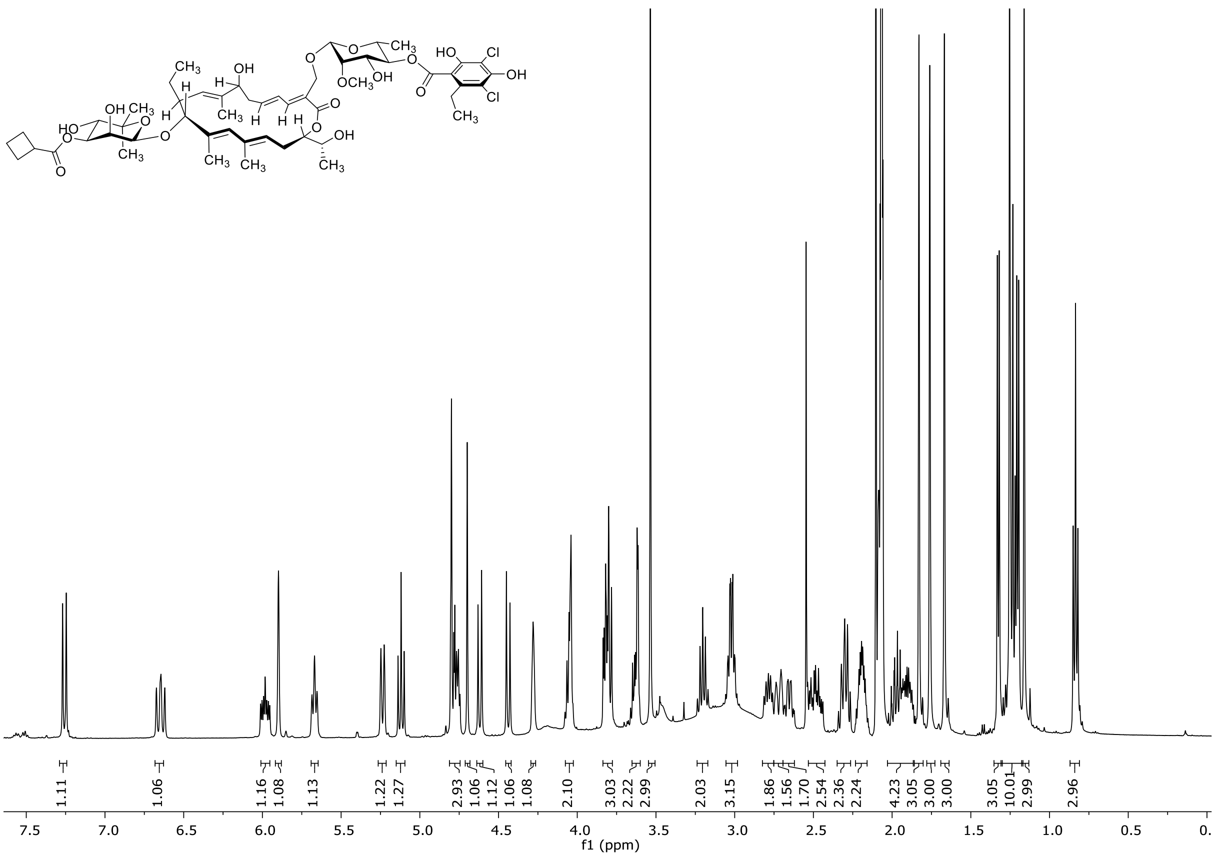

Supplementary Figure 57. ^{1}H NMR (500 MHz, acetone-d_6) of **7z.**

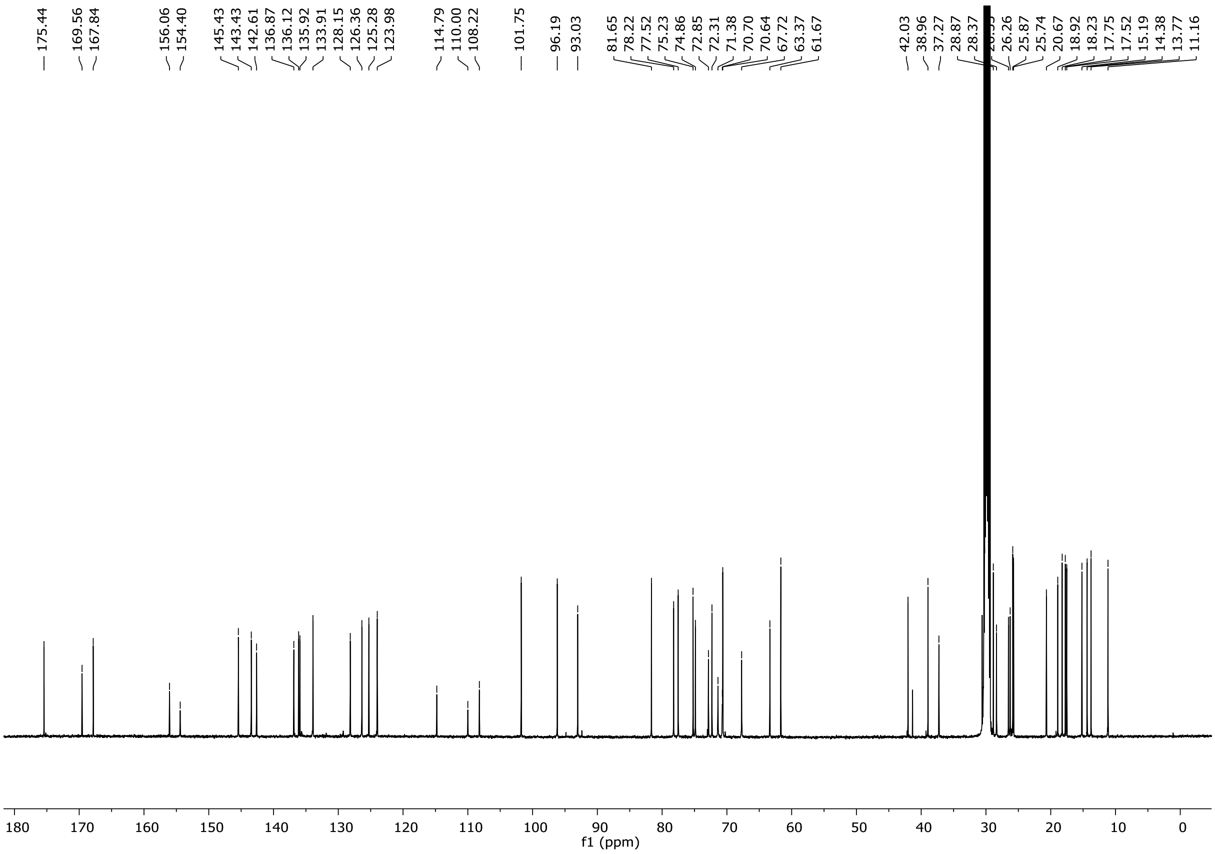

Supplementary Figure 58. ^{13}C NMR (126 MHz, acetone-d_6) of **7z.**

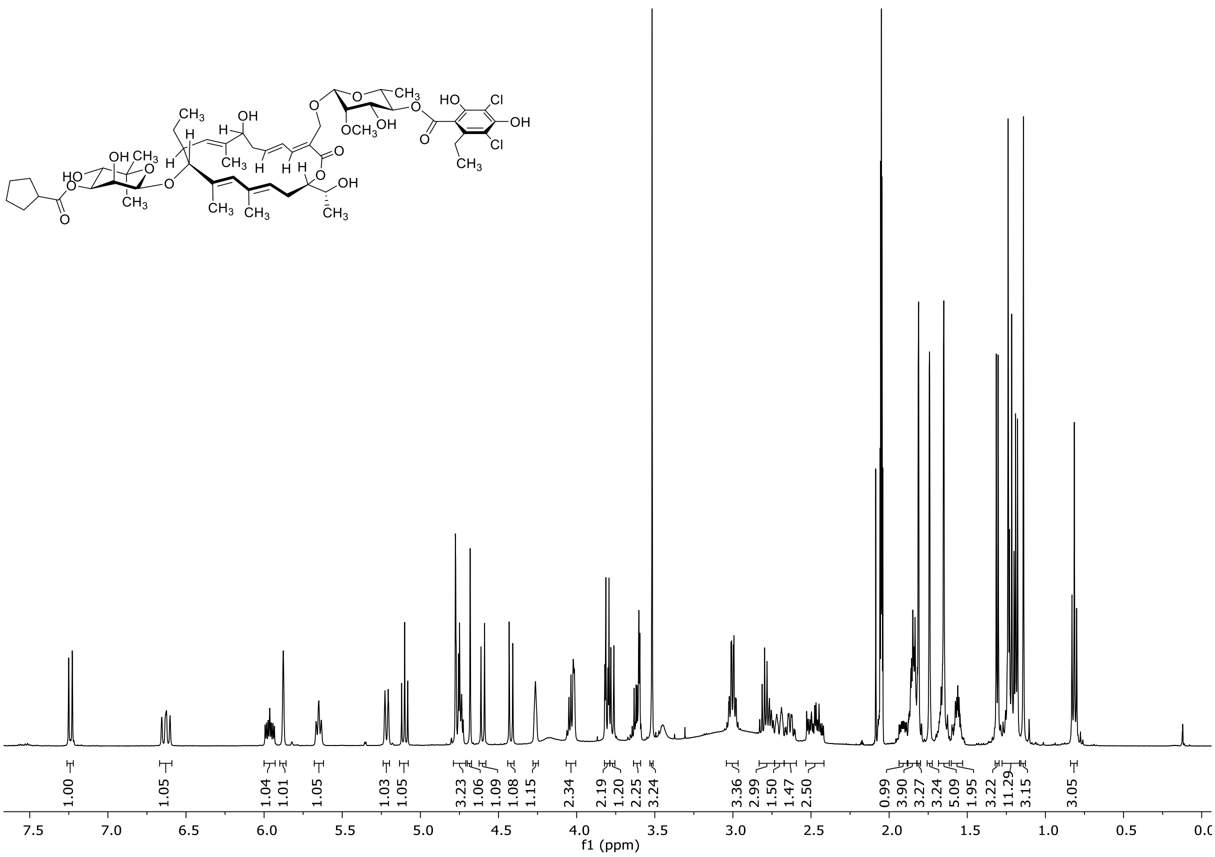

Supplementary Figure 59. ^{1}H NMR (500 MHz, acetone-d_6) of **7aa.**

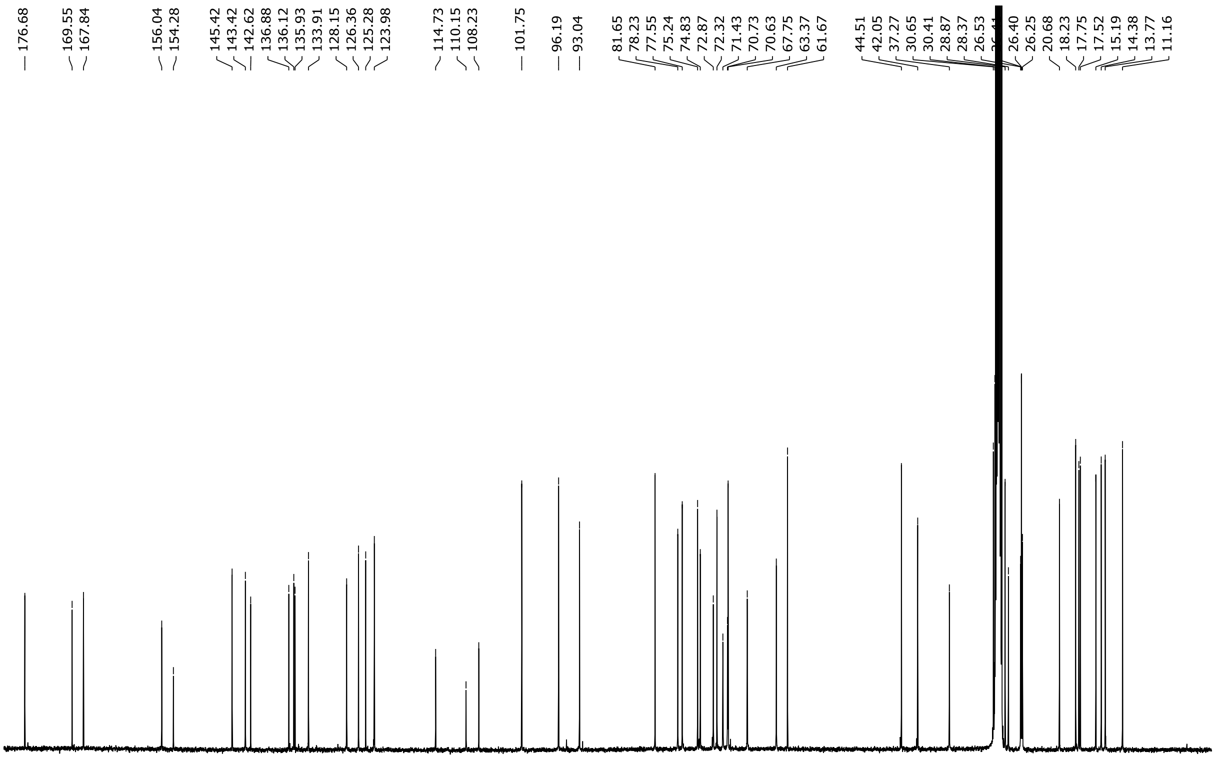

Supplementary Figure 60. ^{13}C NMR (126 MHz, acetone-d_6) of **7aa.**

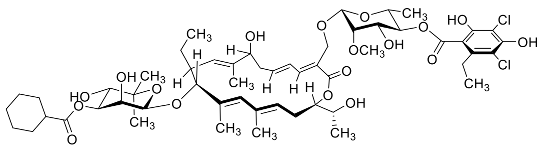

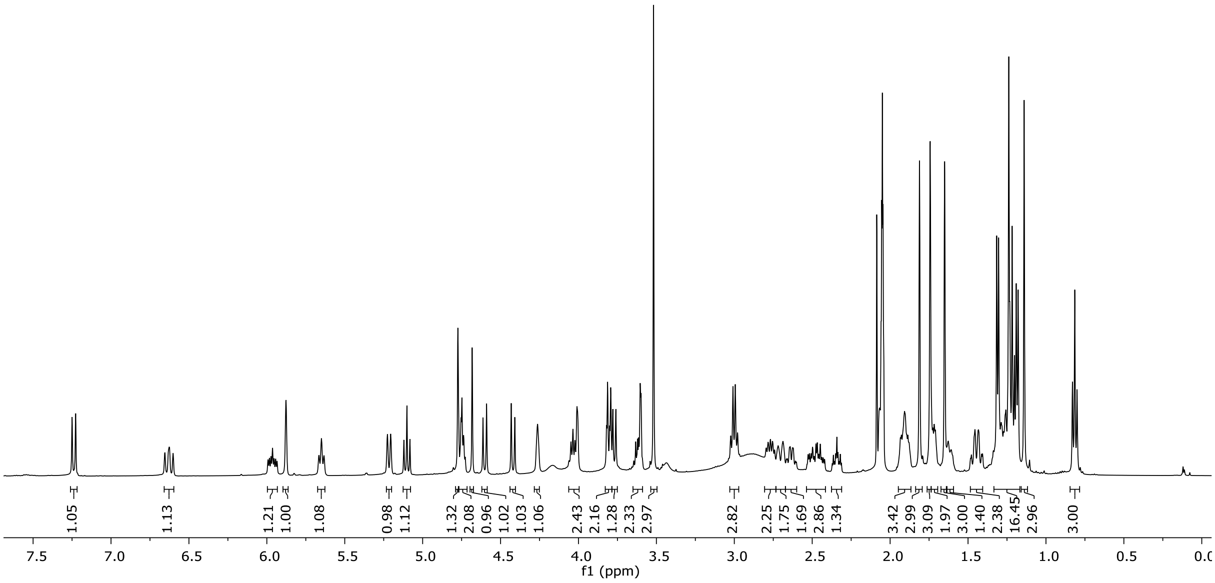

Supplementary Figure 61. ^{1}H NMR (500 MHz, acetone-d_6) of **7ab.**

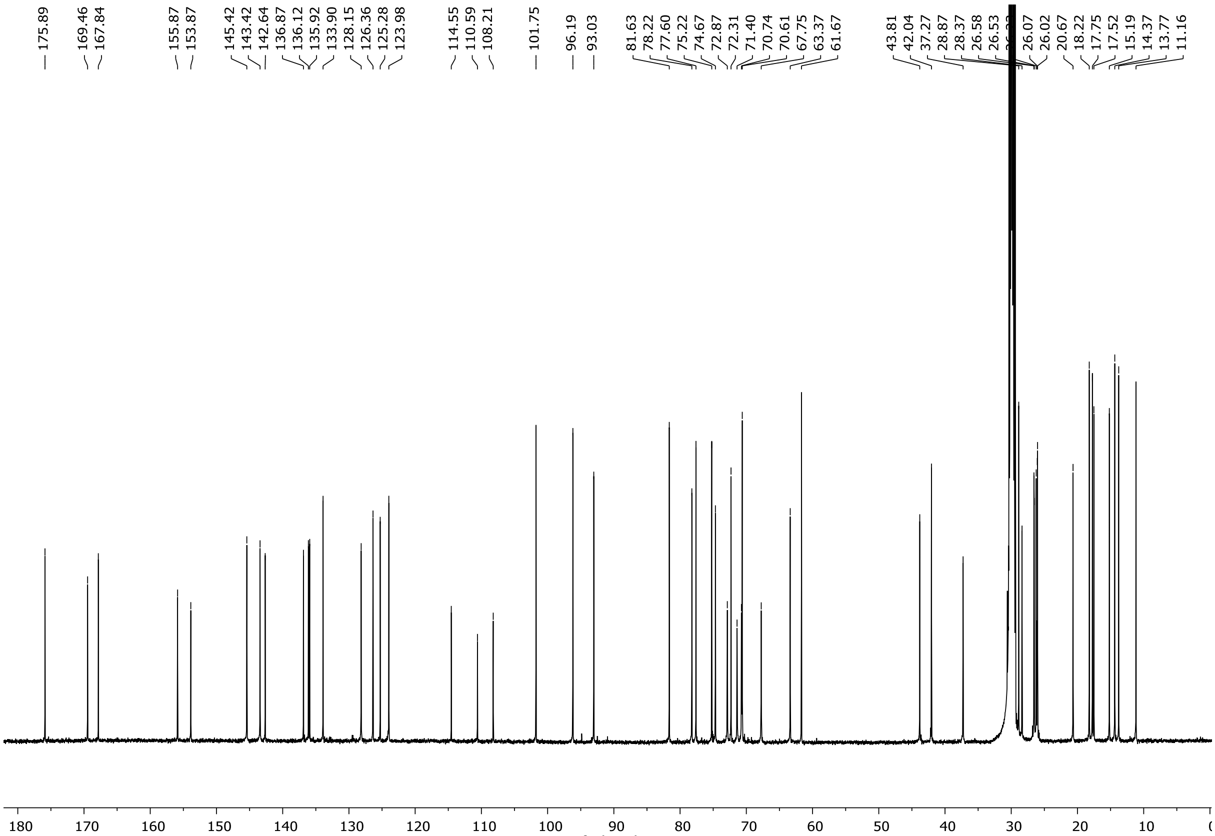

Supplementary Figure 62. ^{13}C NMR (126 MHz, acetone-d_6) of **7ab.**

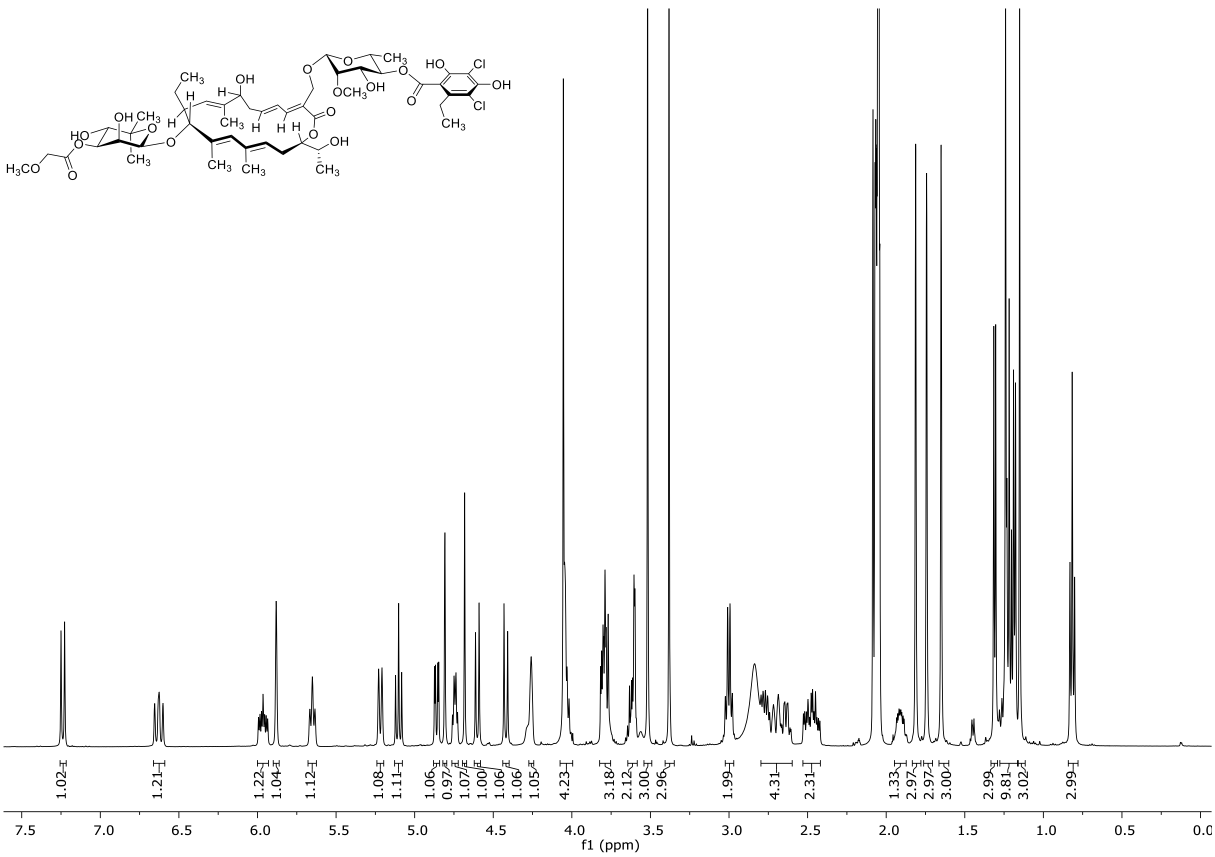

Supplementary Figure 63. ^{1}H NMR (500 MHz, acetone-d_6) of **7ad.**

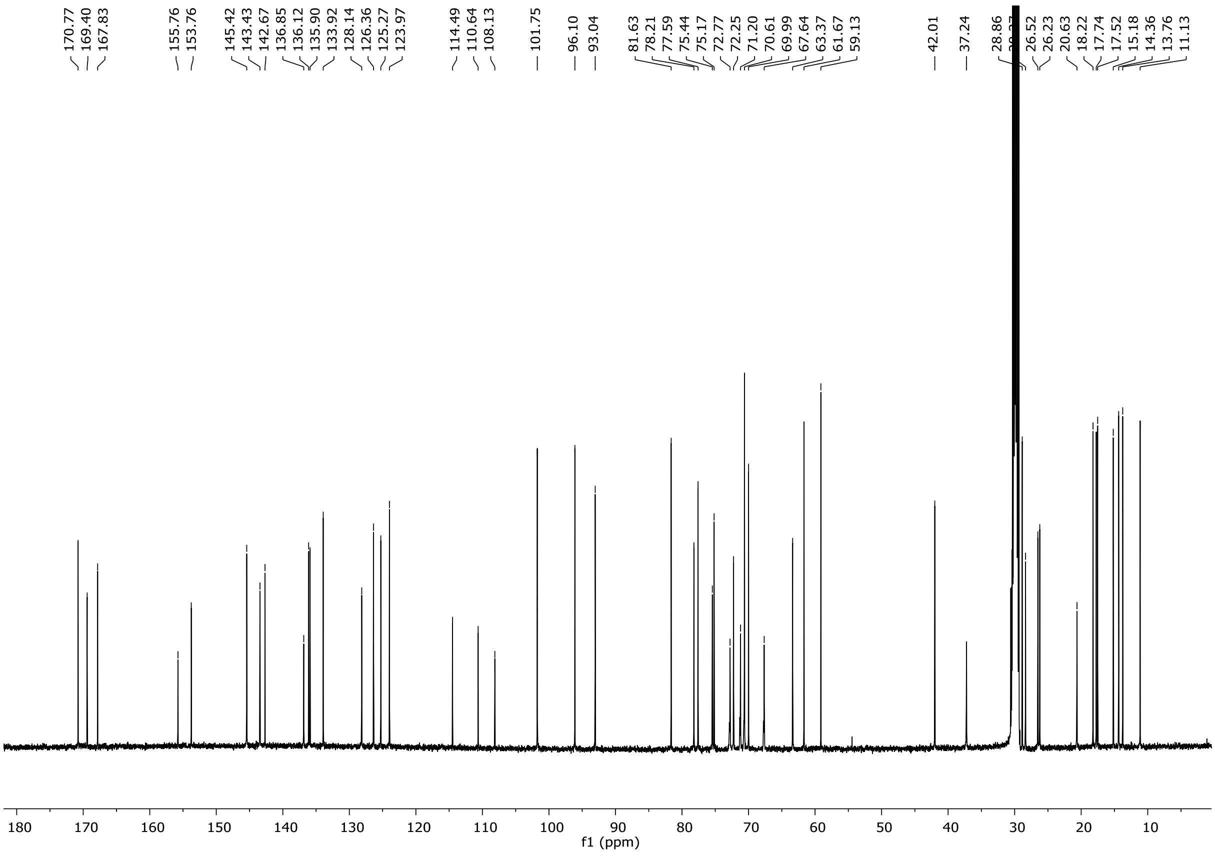

Supplementary Figure 64. ^{13}C NMR (126 MHz, acetone-d_6) of **7ad.**

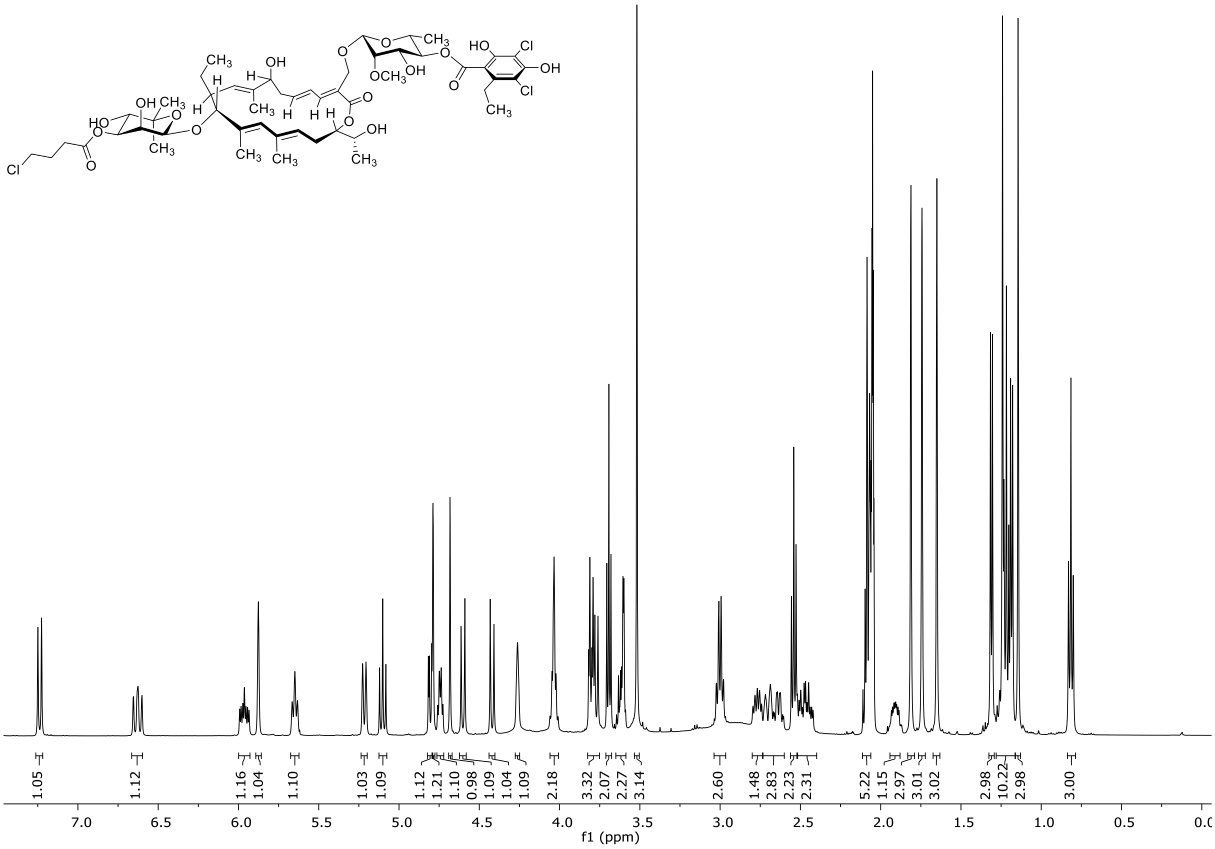

Supplementary Figure 65. ^{1}H NMR (500 MHz, acetone-d_6) of **7ag.**

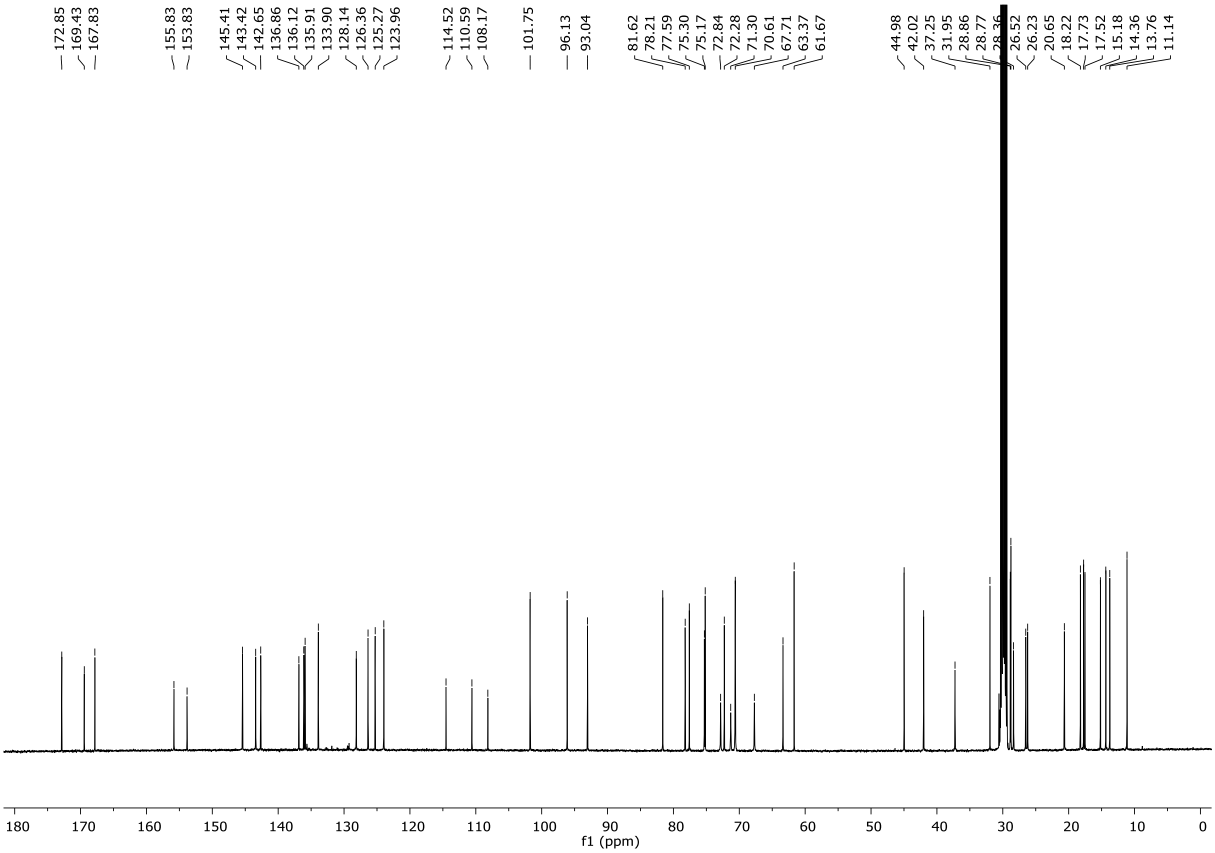

Supplementary Figure 66. ^{13}C NMR (126 MHz, acetone-d_6) of **7ag.**

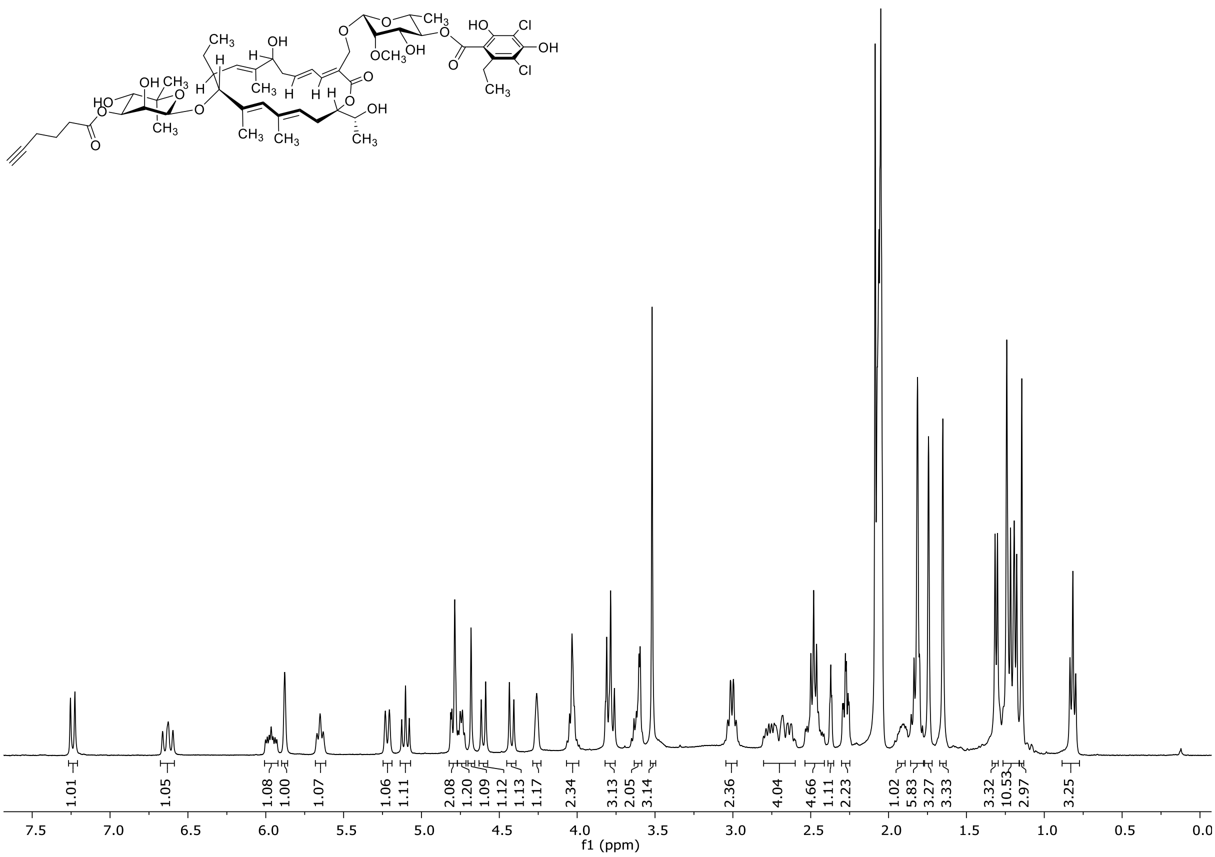

Supplementary Figure 67. ^{1}H NMR (400 MHz, acetone-d_6) of **7ah.**

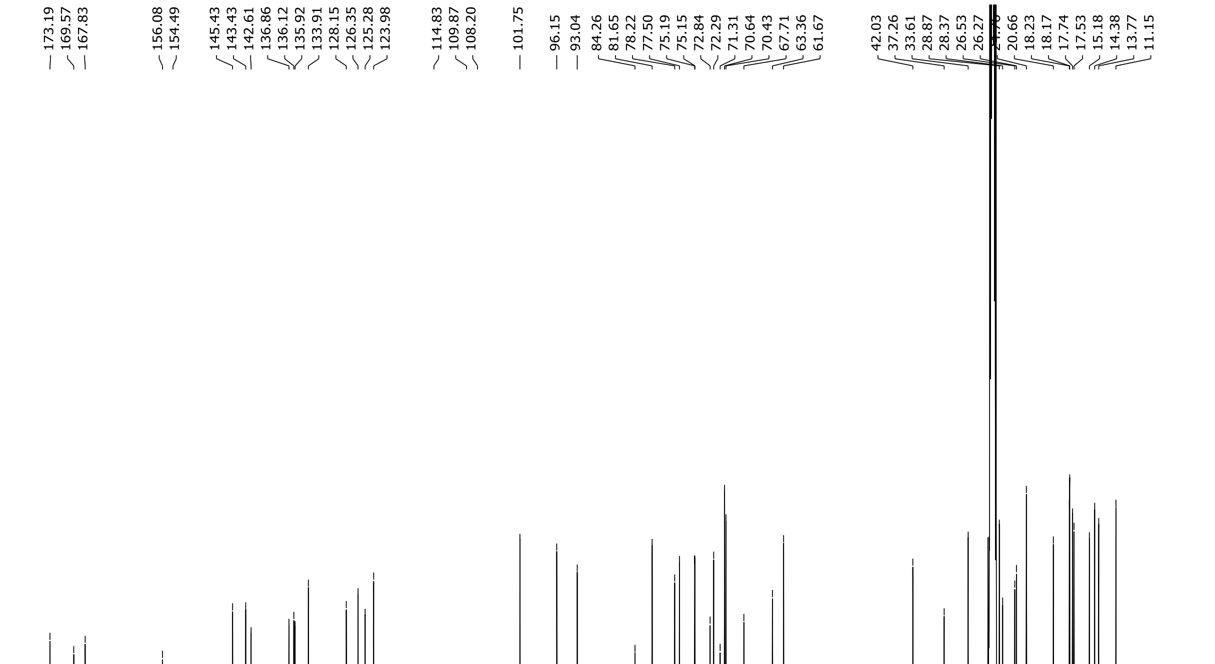

Supplementary Figure 68. ^{13}C NMR (126 MHz, acetone-d_6) of **7ah.**

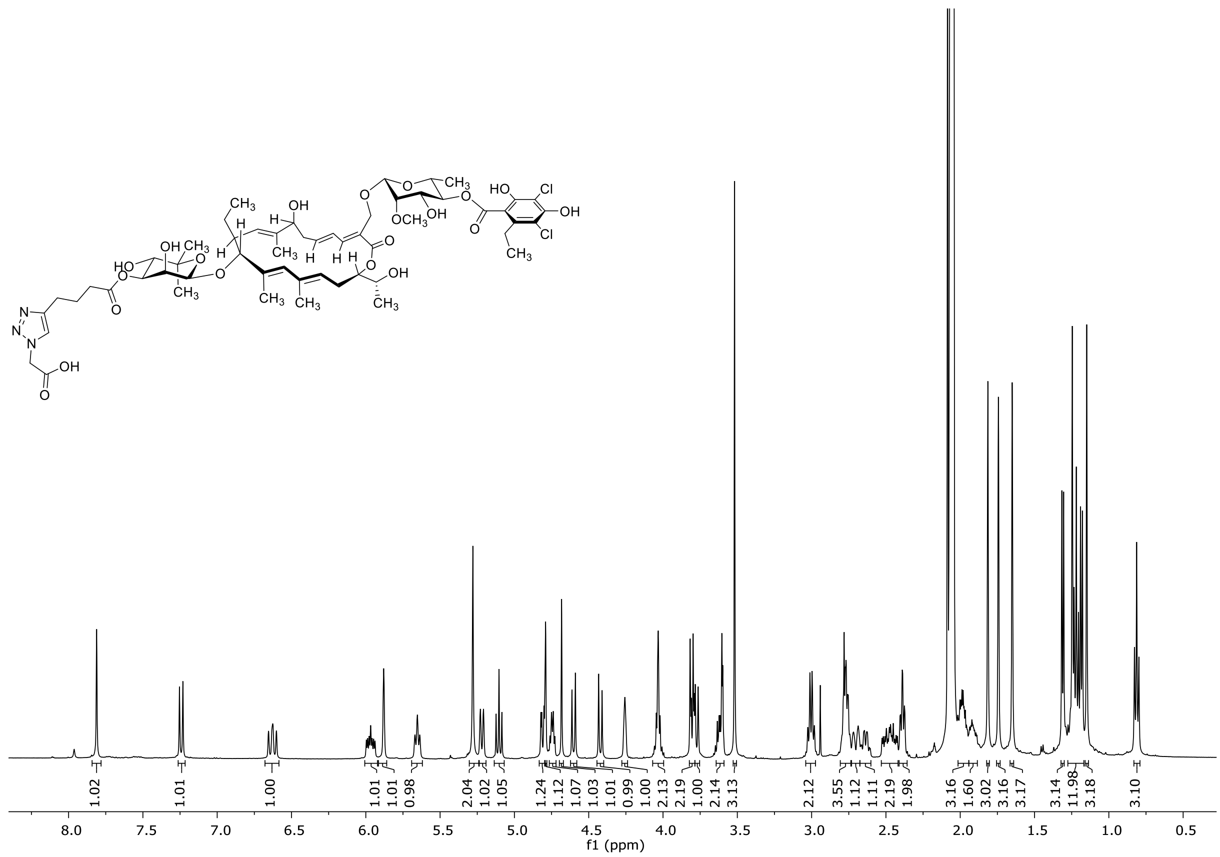

Supplementary Figure 69. ^{1}H NMR (500 MHz, acetone-d_6) of **7ai.**

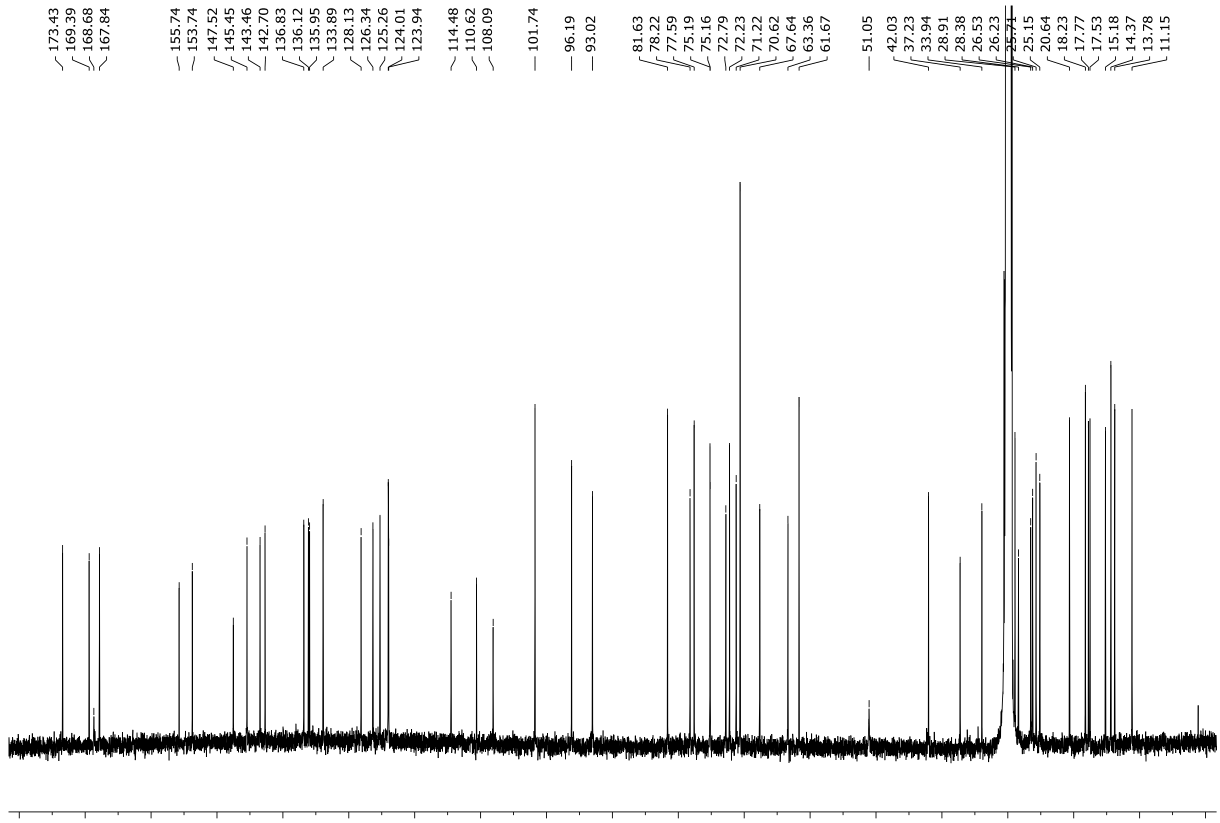

Supplementary Figure 70. ^{13}C NMR (126 MHz, acetone-d_6) of **7ai.**

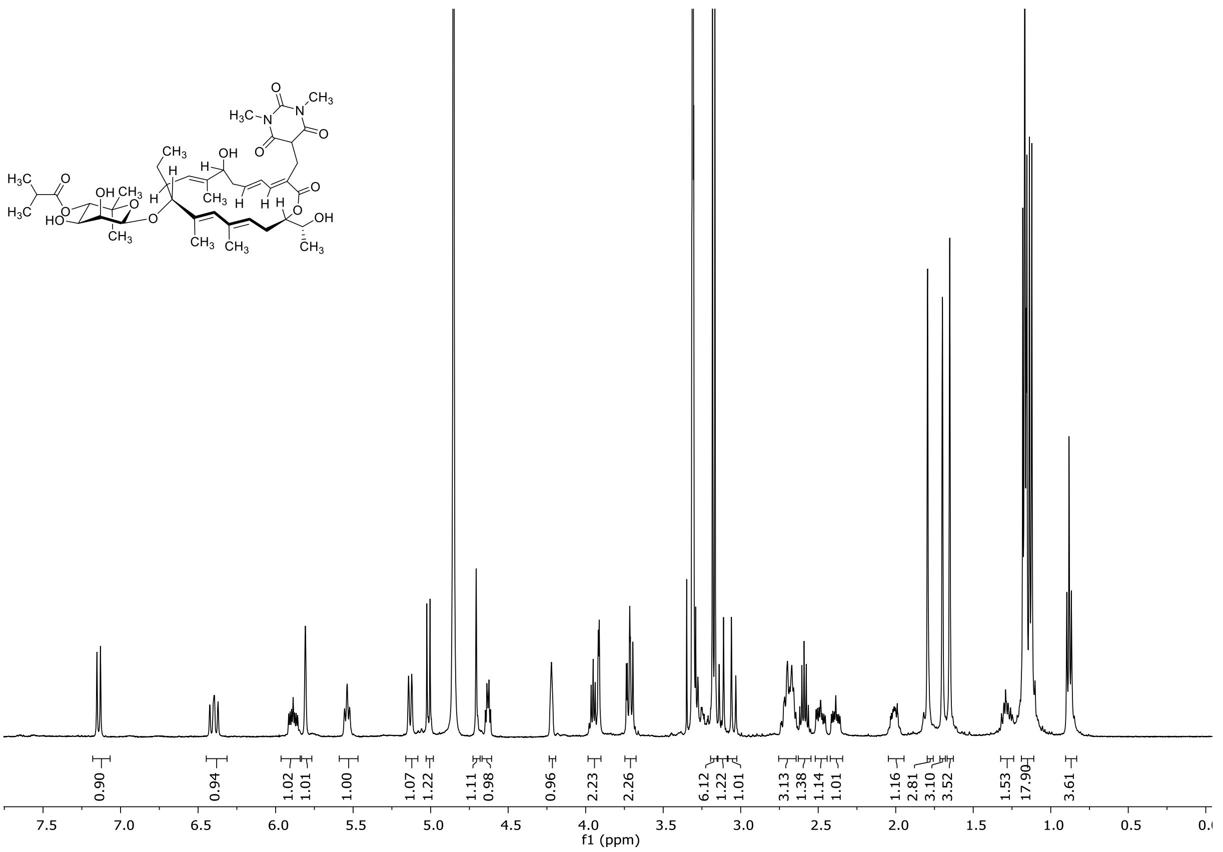

Supplementary Figure 71. ^{1}H NMR (500 MHz, CD_3OD) of **9**.

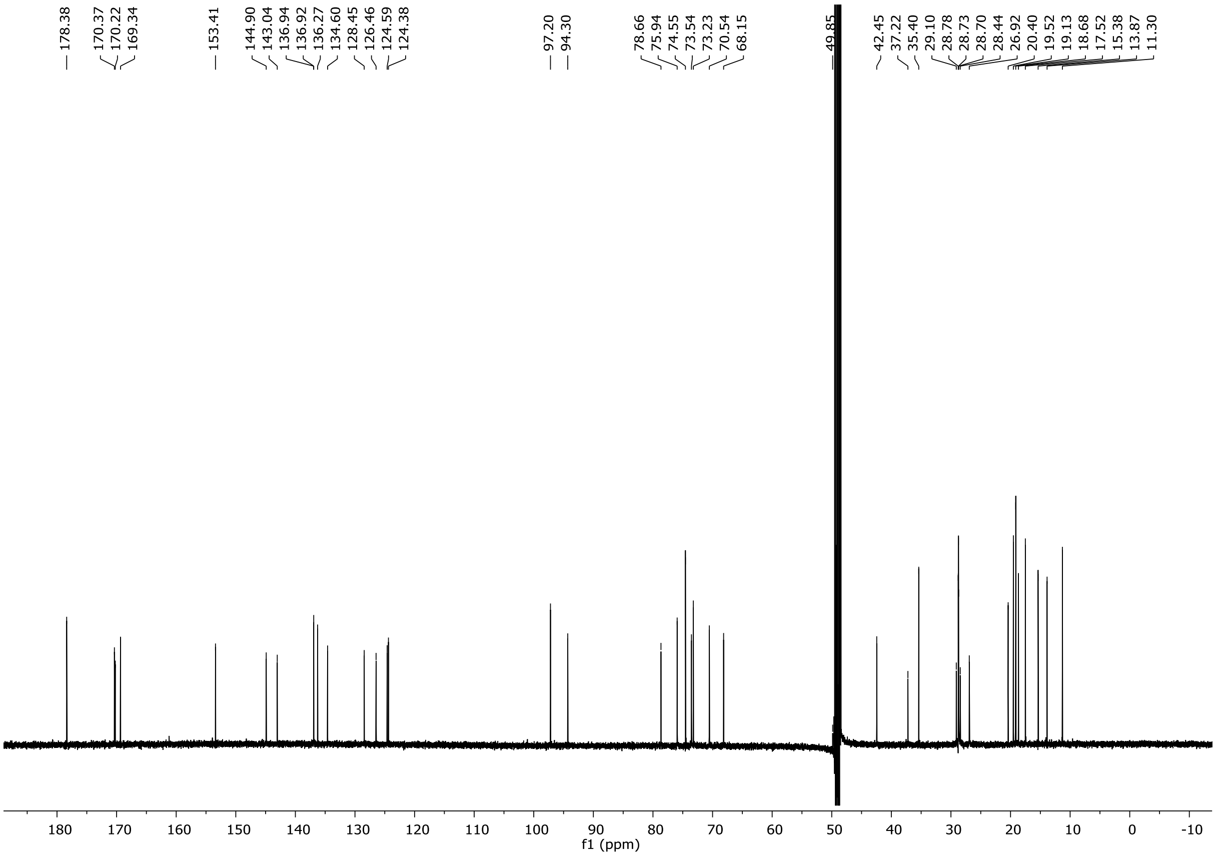

Supplementary Figure 72. ^{13}C NMR (126 MHz, CD_3OD) of **9.**

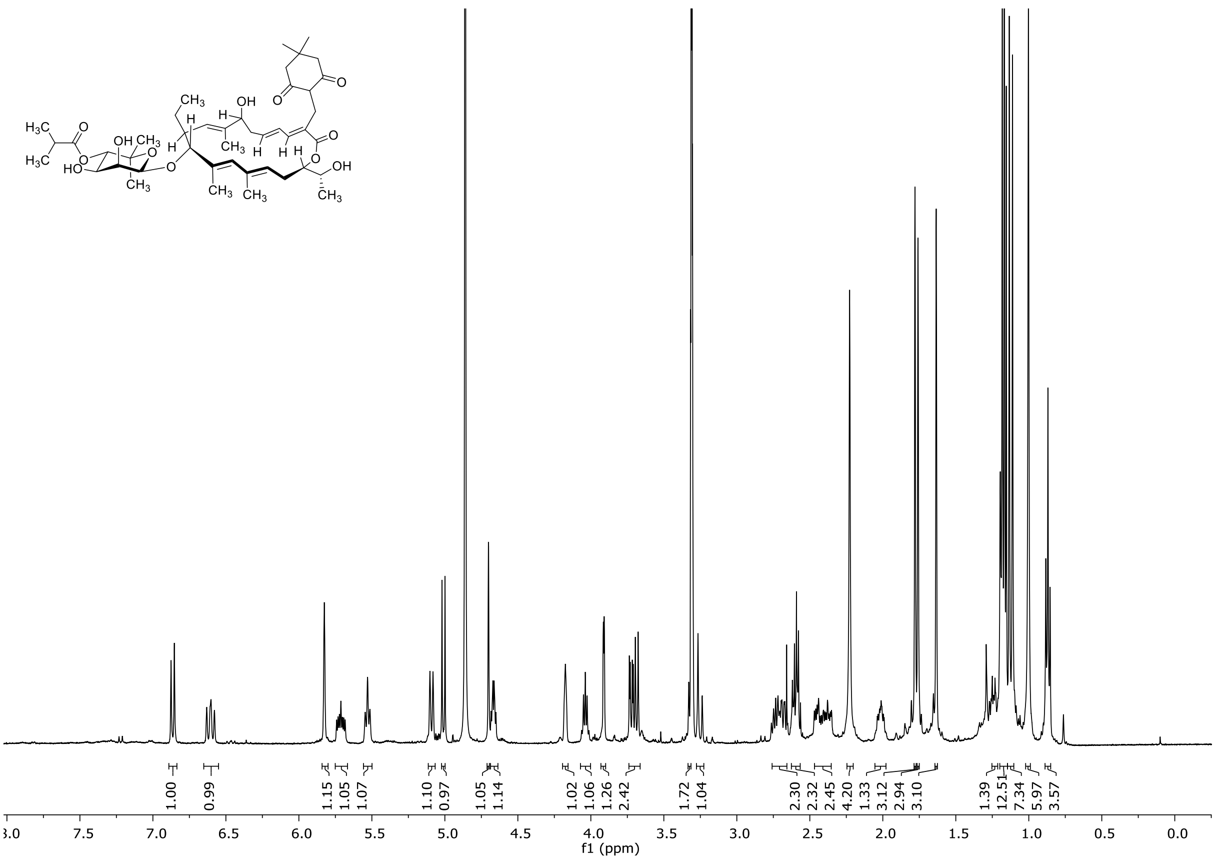

Supplementary Figure 73. ^{1}H NMR (500 MHz, CD_3OD) of **10**.

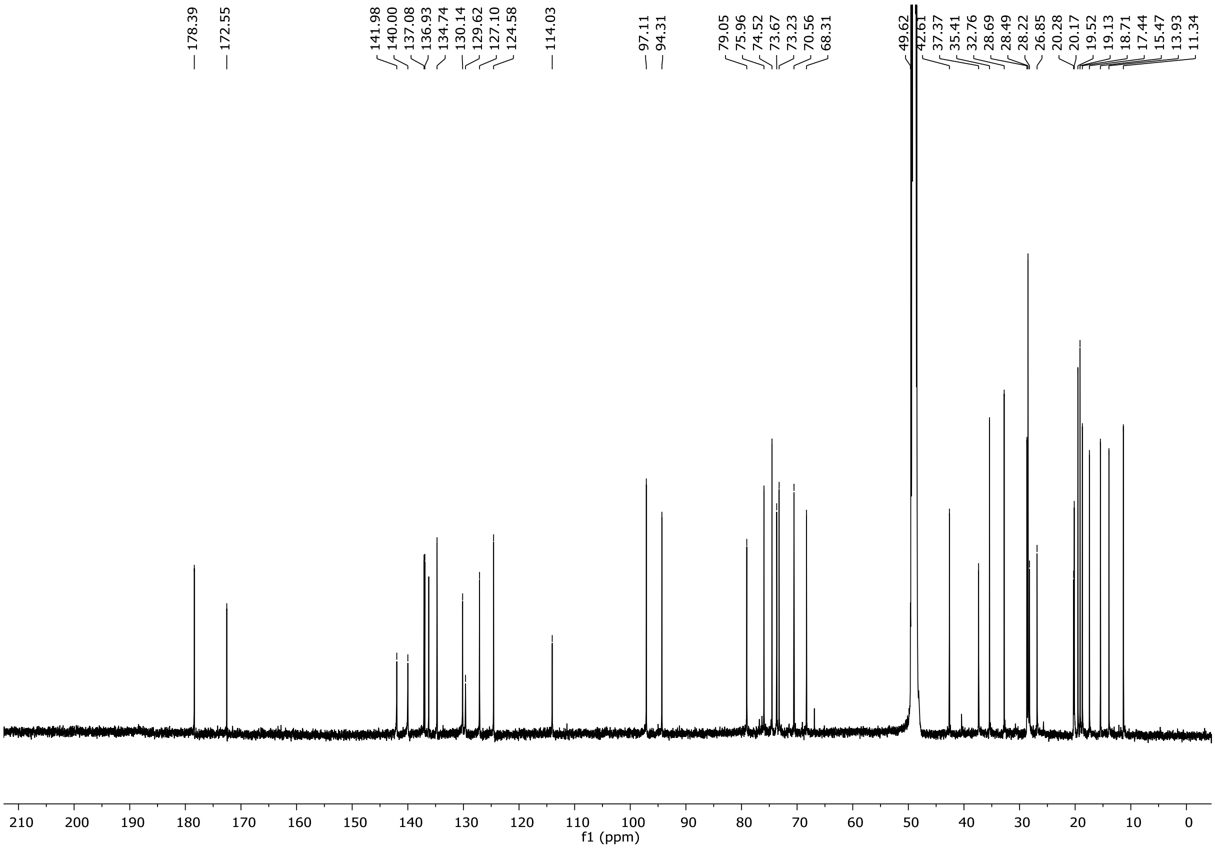

Supplementary Figure 74. ^{13}C NMR (126 MHz, CD_3OD) of **10.**

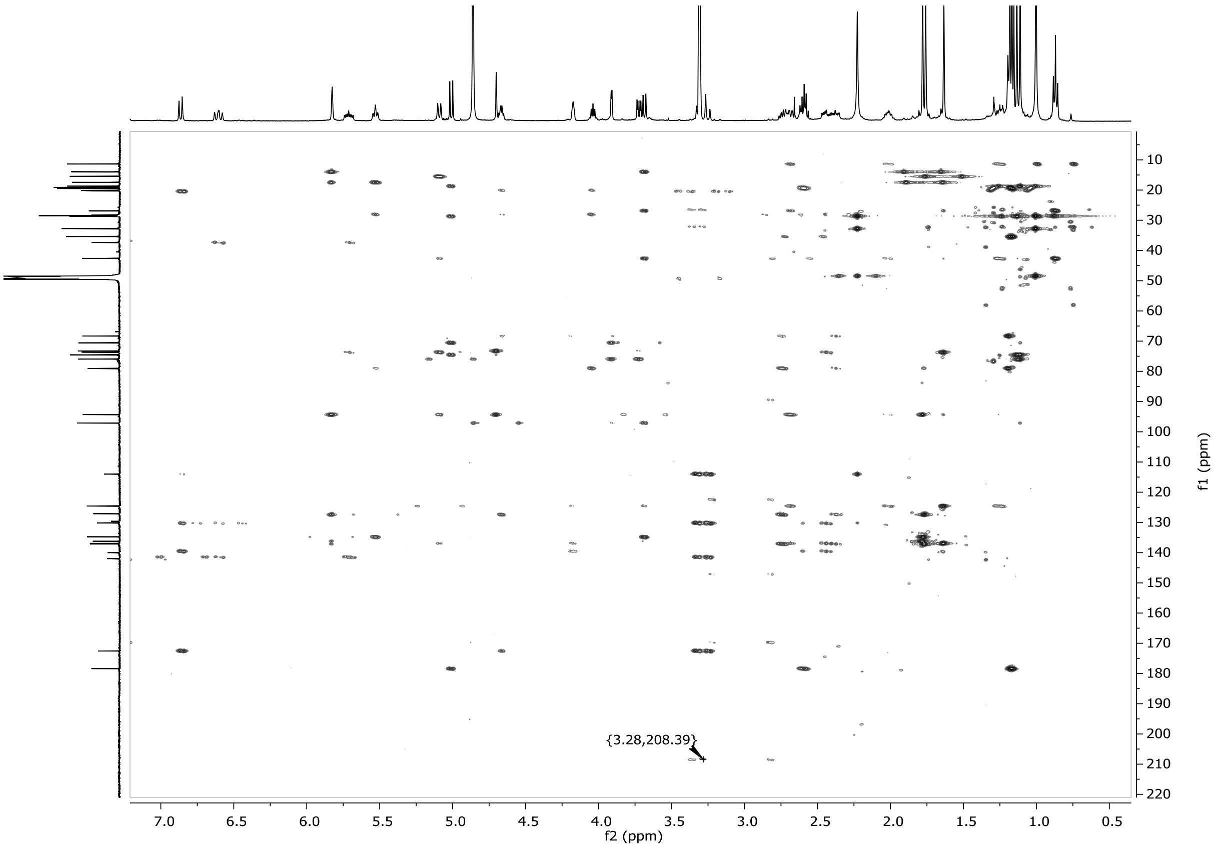

Supplementary Figure 75. HMBC NMR (500 MHz, 126 MHz, CD_3OD) of **10.**

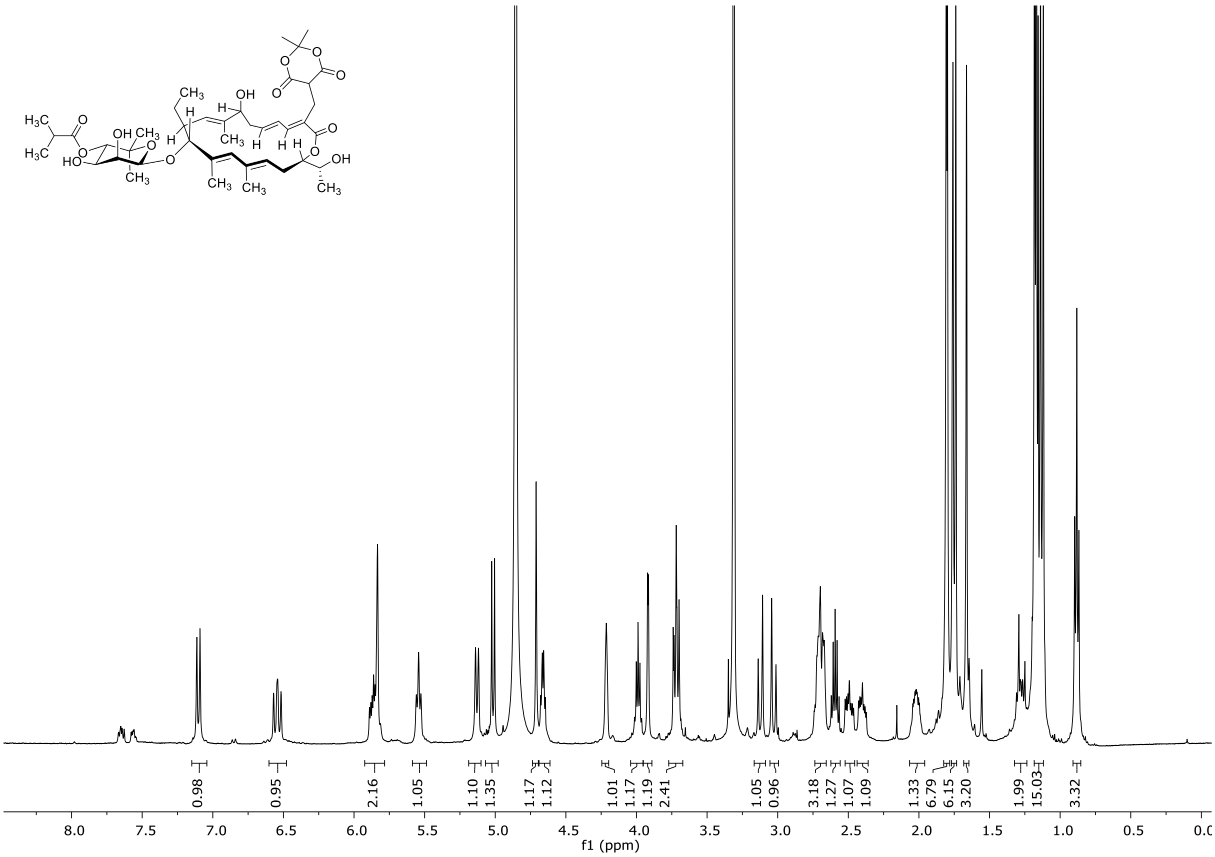

Supplementary Figure 76. ^{1}H NMR (500 MHz, CD_3OD) of **11**.

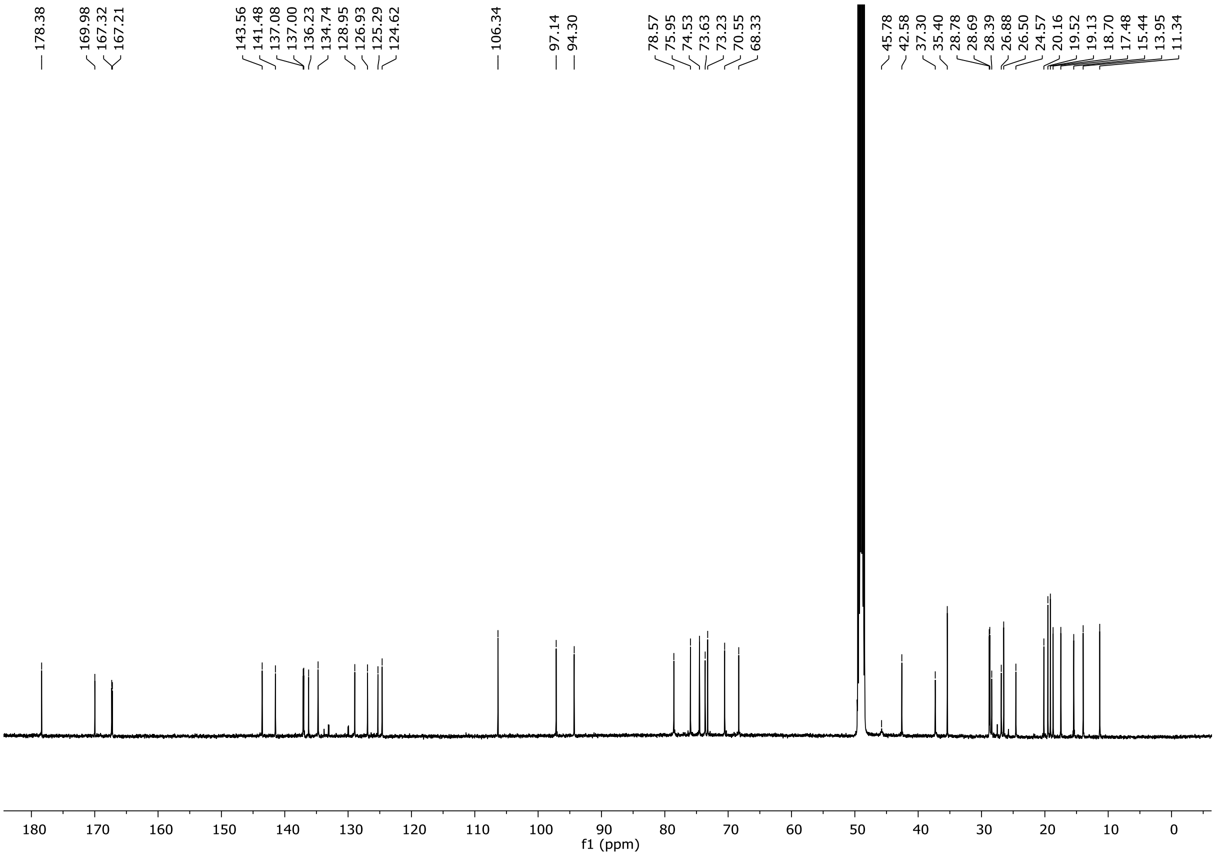

Supplementary Figure 77. ^{13}C NMR (126 MHz, CD_3OD) of **11.**

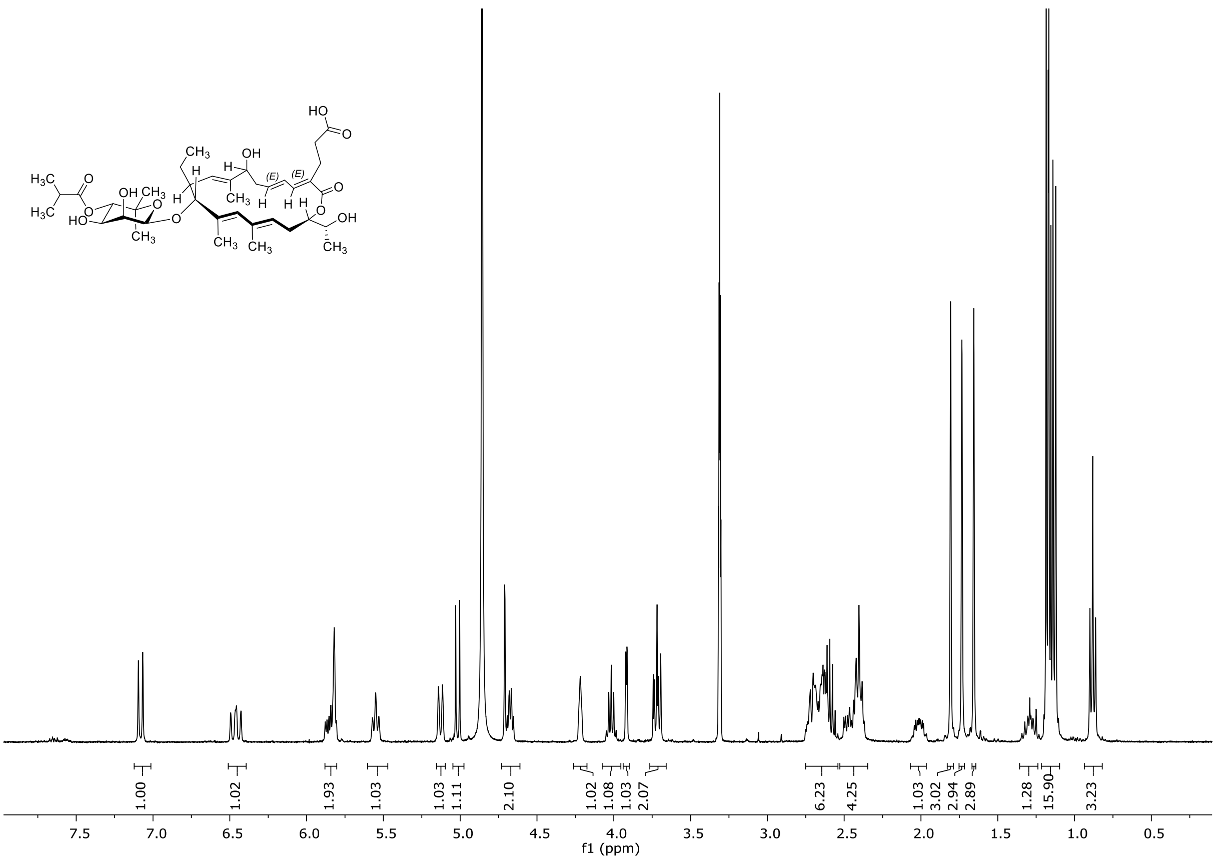

Supplementary Figure 78. ^{1}H NMR (400 MHz, CD_3OD) of ***(E)*-13**.

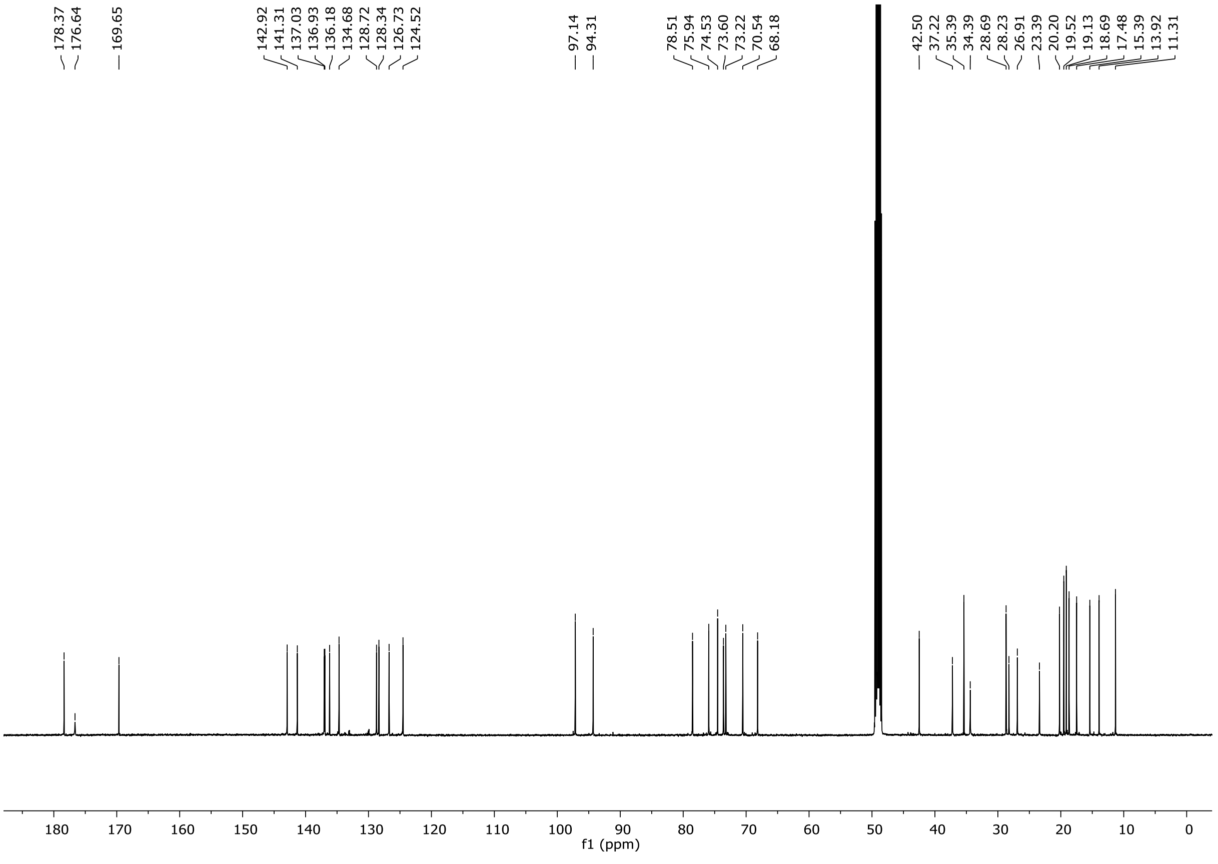

Supplementary Figure 79. ^{13}C NMR (126 MHz, CD_3OD) of ***(E)*-13**.

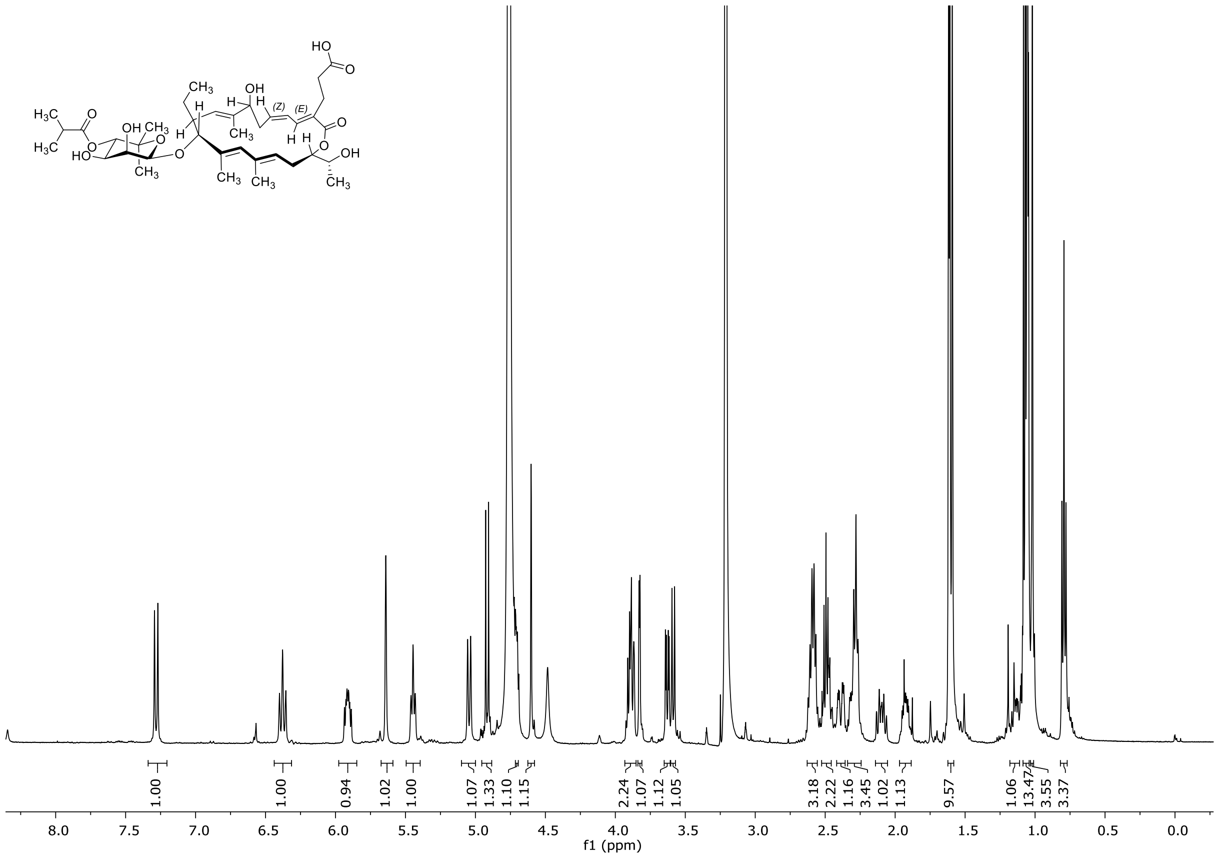

Supplementary Figure 80. ^{1}H NMR (500 MHz, CD_3OD) of ***(Z)*-13**.

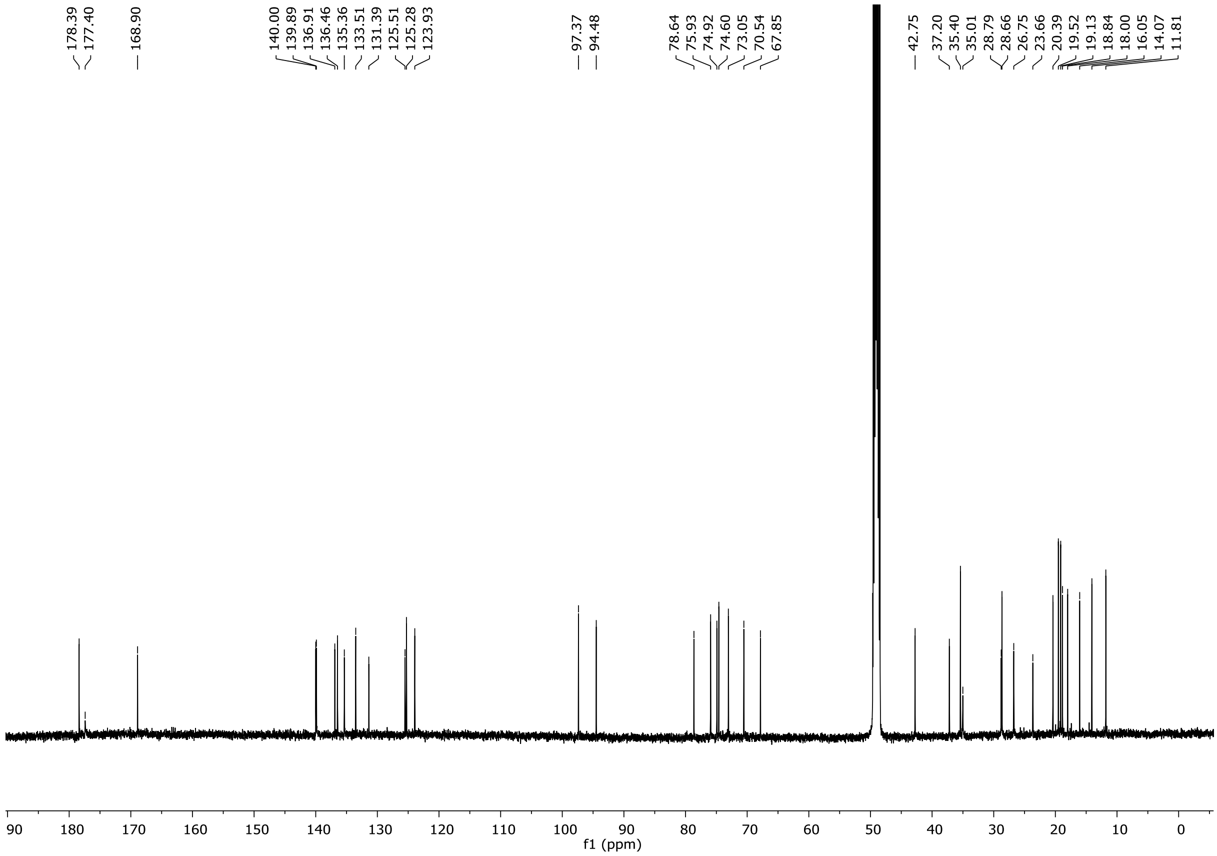

Supplementary Figure 81. ^{13}C NMR (126 MHz, CD_3OD) of ***(Z)*-13**.

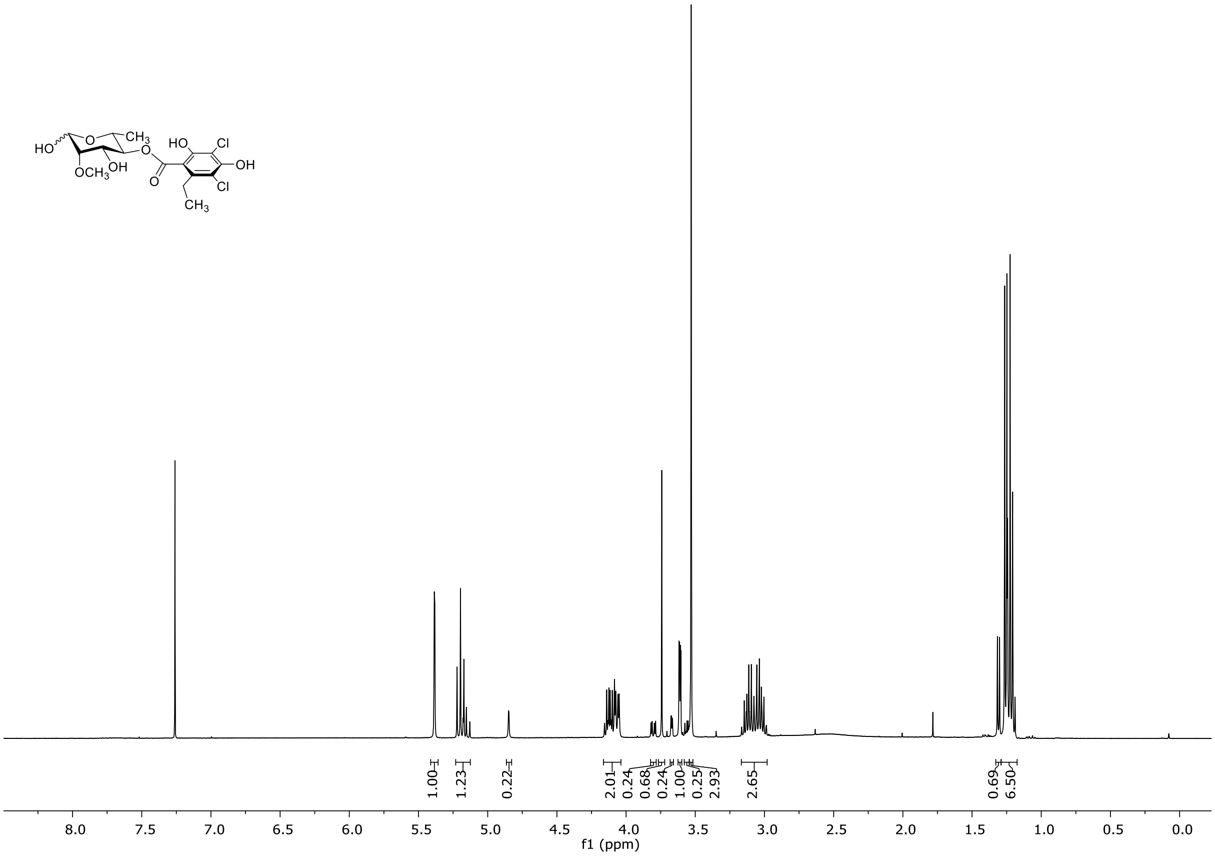

Supplementary Figure 82. ^{1}H NMR (400 MHz, $CDCl_3$) of **12.**

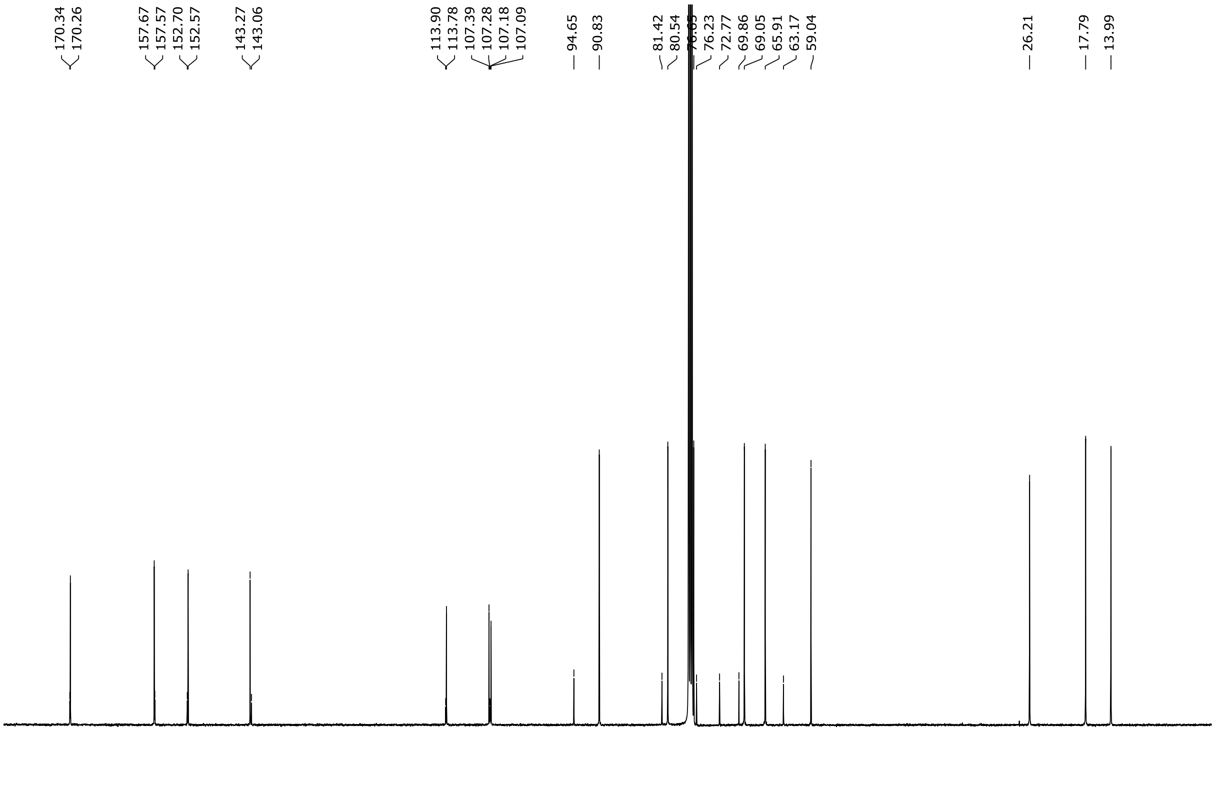

Supplementary Figure 83. ^{13}C NMR (126 MHz, $CDCl_3$) of **12**.

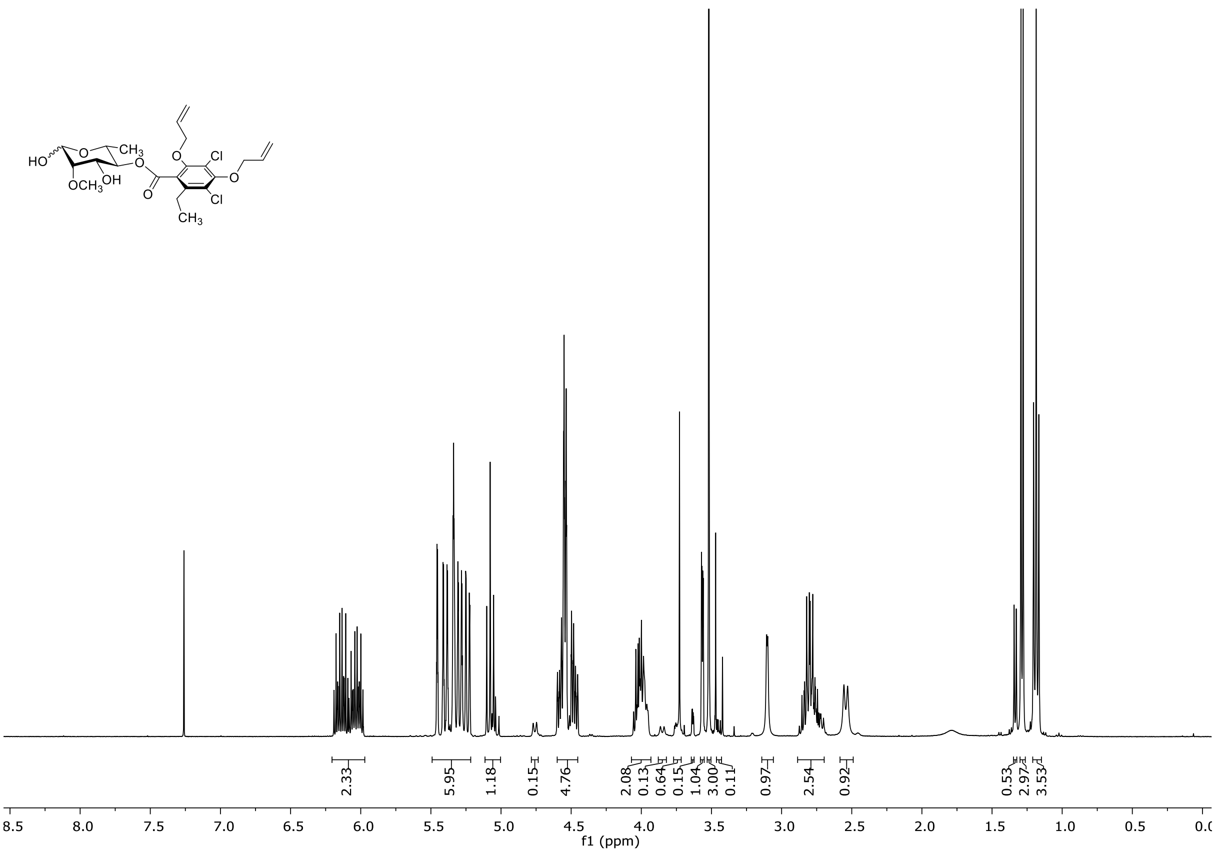

Supplementary Figure 84. ^{1}H NMR (400 MHz, $CDCl_3$) of **S1.**

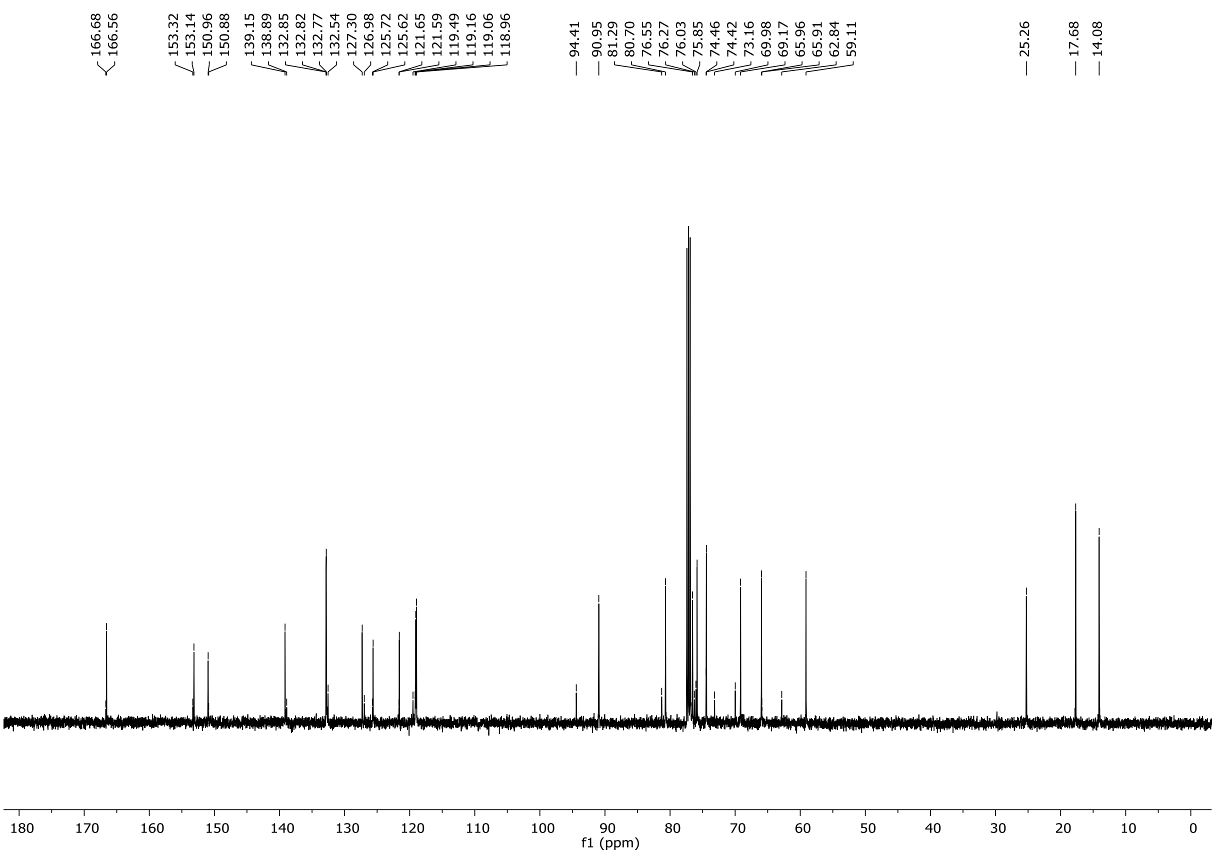

Supplementary Figure 85. ^{13}C NMR (126 MHz, $CDCl_3$) of **S1**.

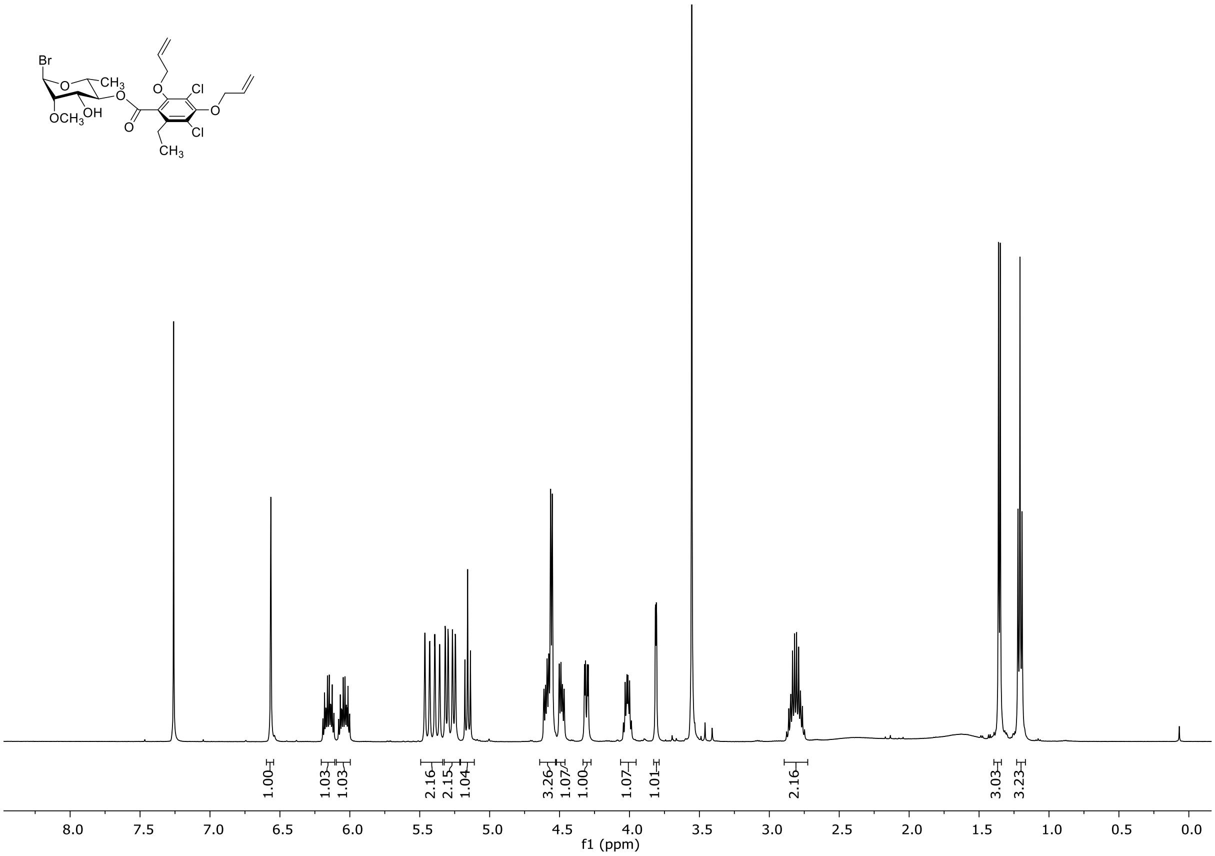

Supplementary Figure 86. ^{1}H NMR (500 MHz, $CDCl_3$) of **14.**

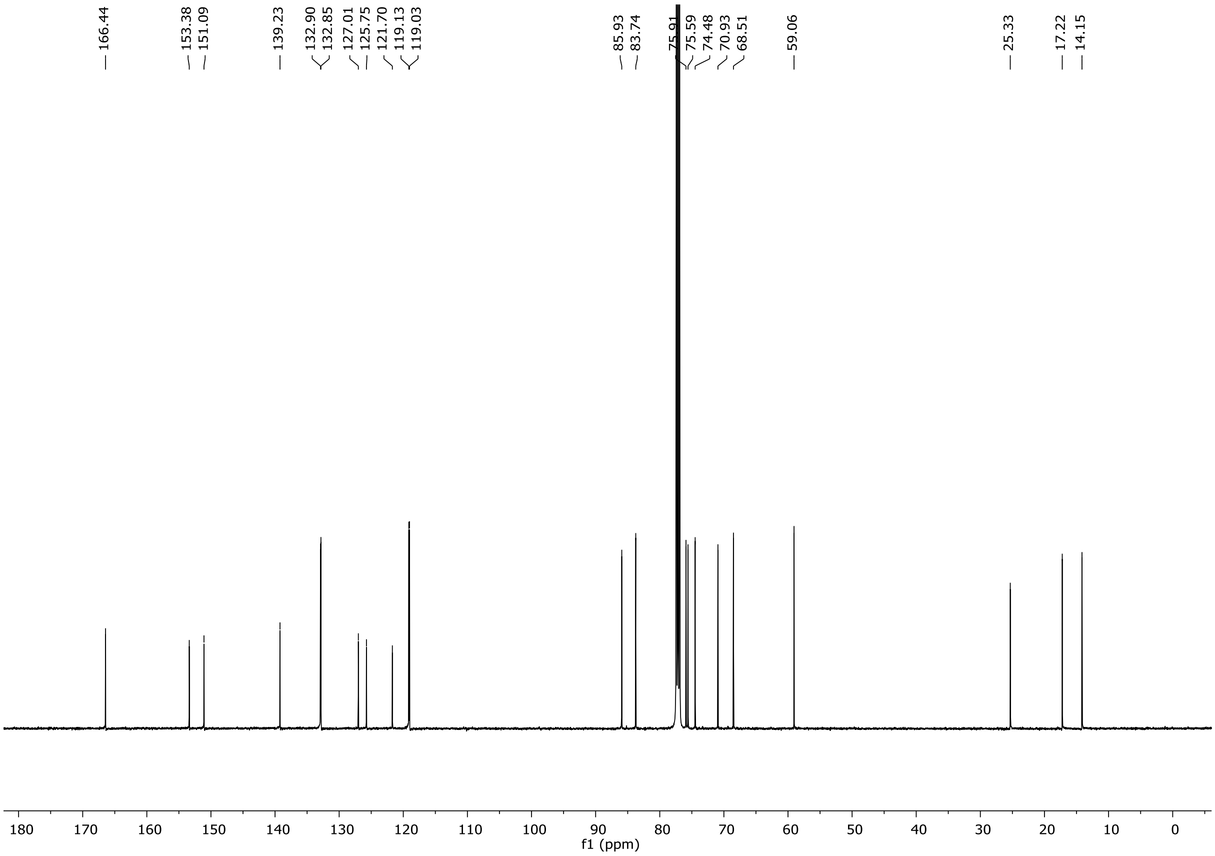

Supplementary Figure 87. ^{13}C NMR (126 MHz, $CDCl_3$) of **14**.

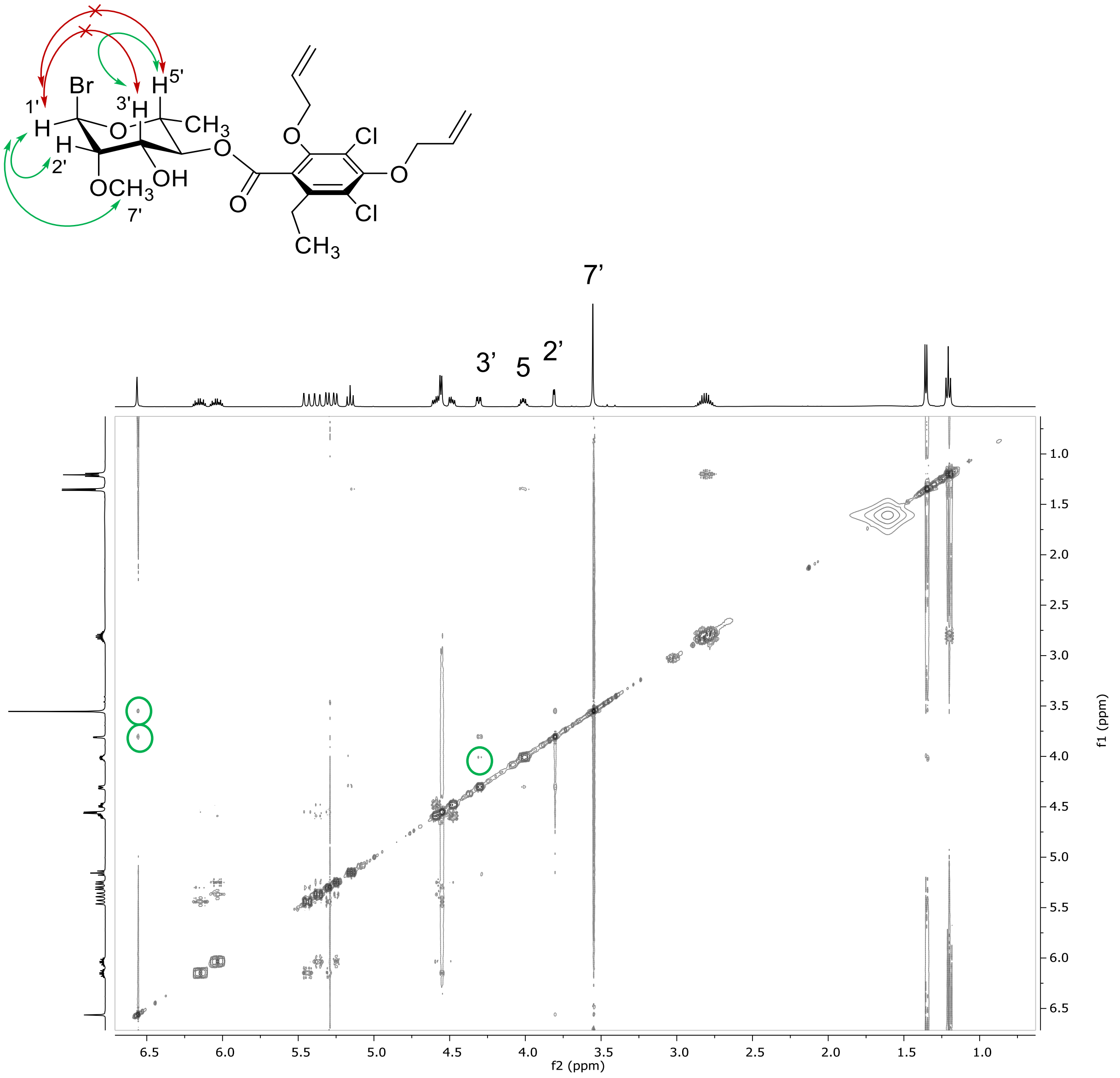

Supplementary Figure 88. NOESY NMR (500 MHz, $CDCl_3$) of **14**.

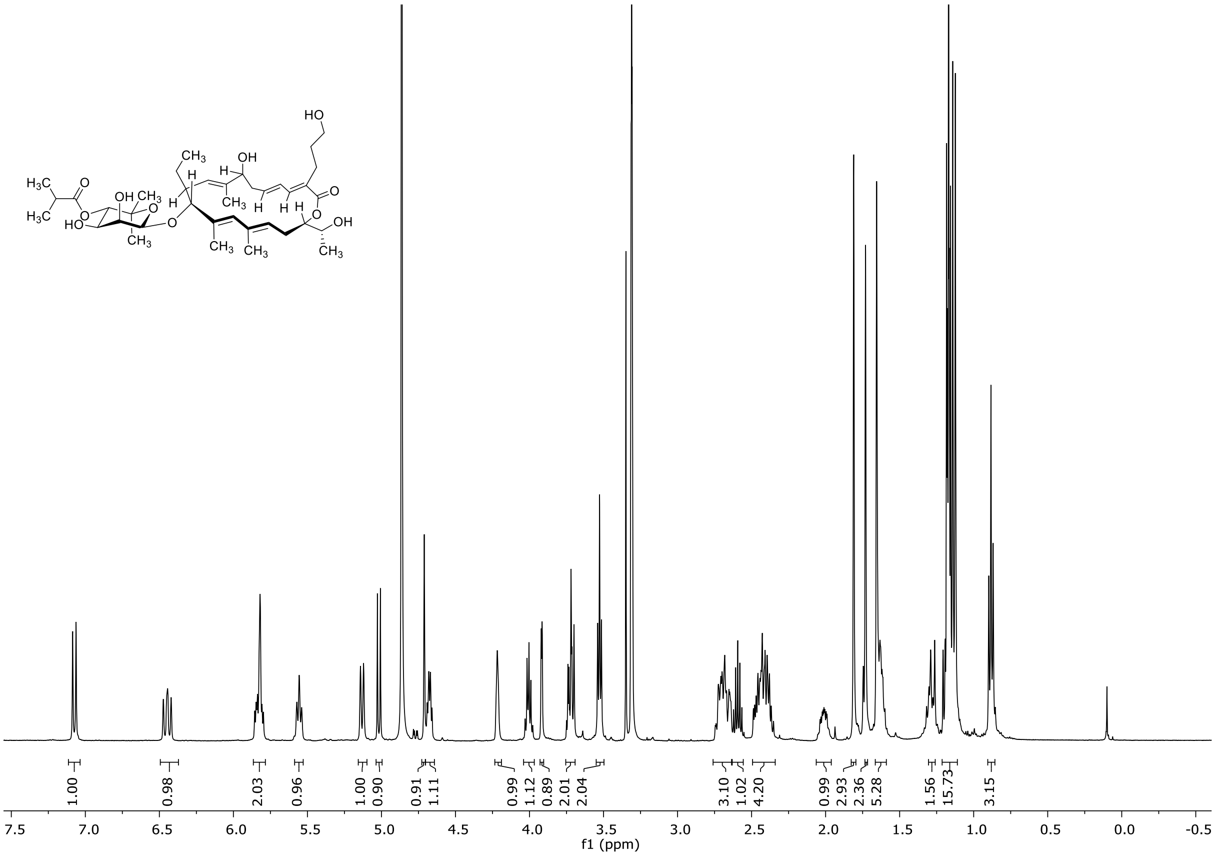

Supplementary Figure 89. ^{1}H NMR (500 MHz, CD_3OD) of **15.**

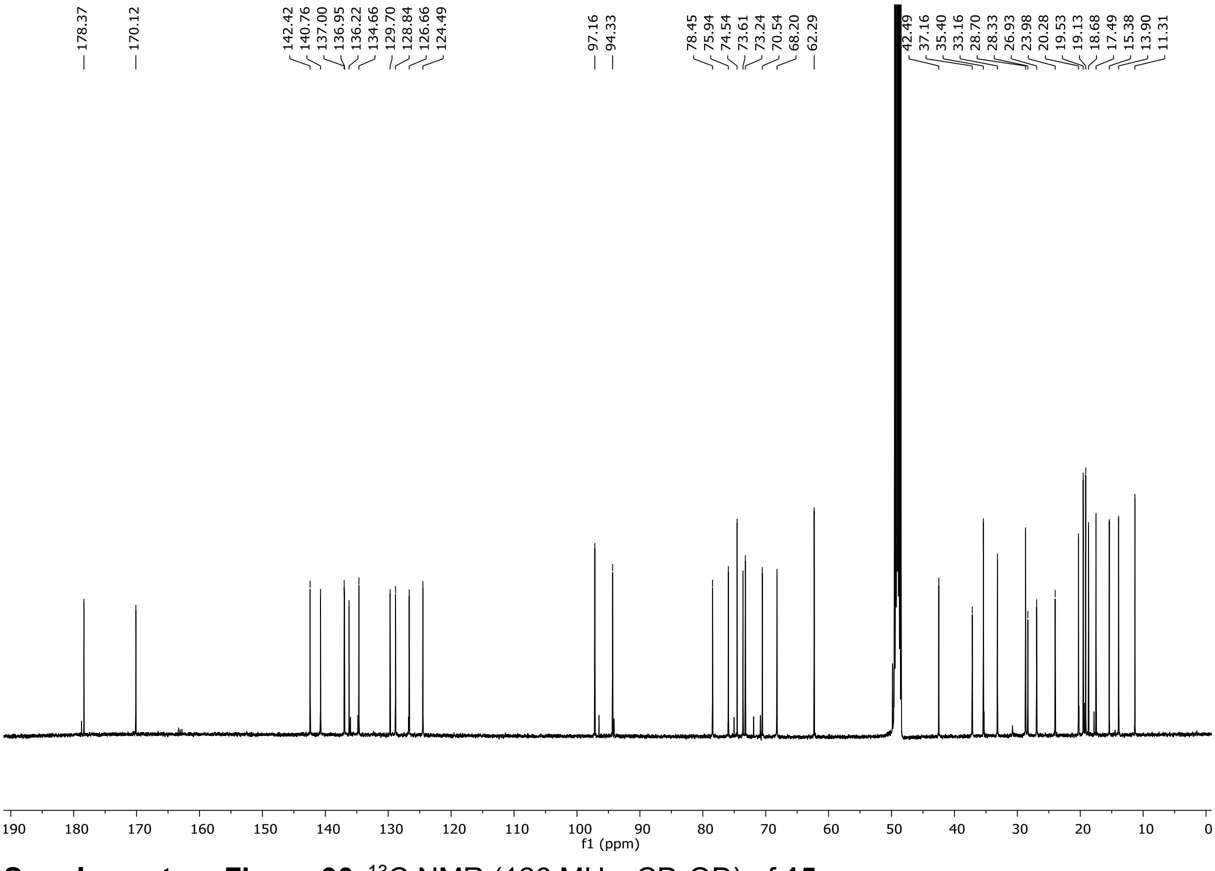

Supplementary Figure 90. ^{13}C NMR (126 MHz, CD_3OD) of **15**.

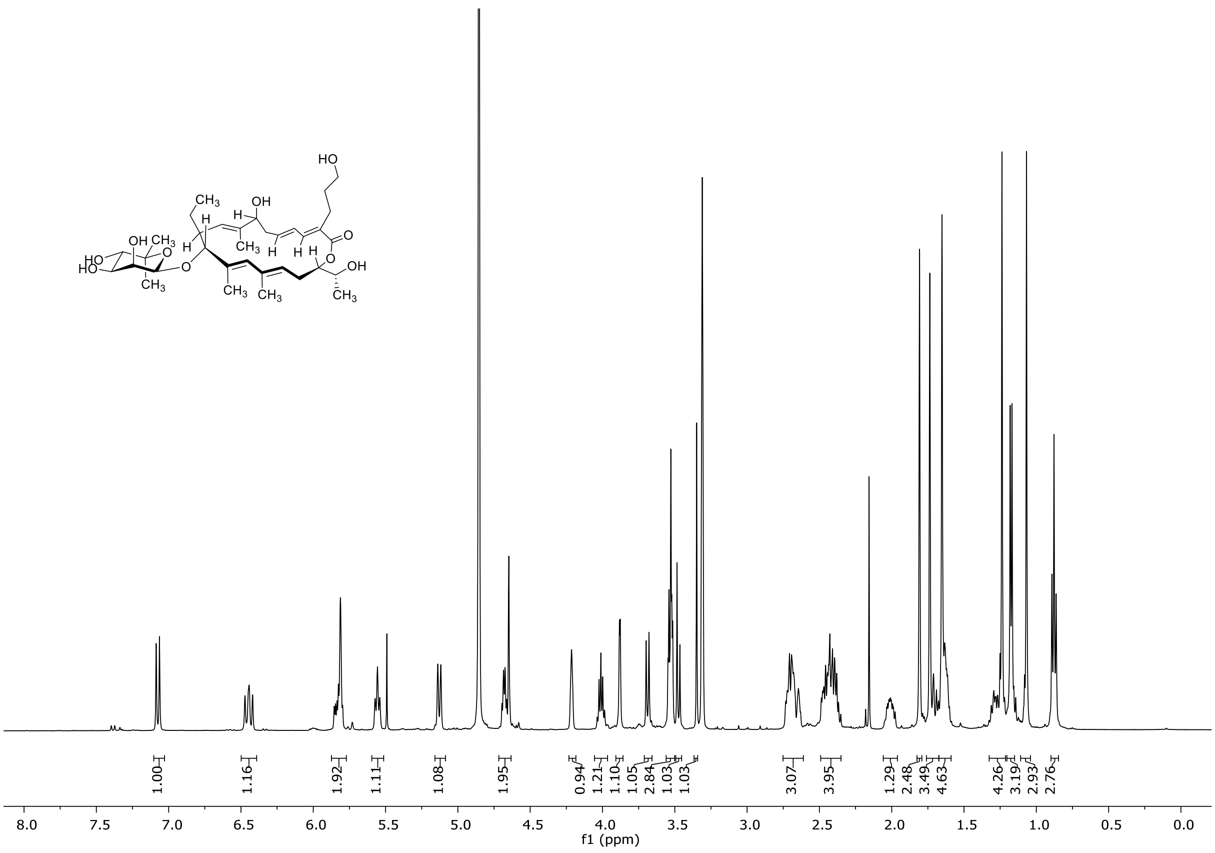

Supplementary Figure 91. ^{1}H NMR (500 MHz, CD_3OD) of **S2.**

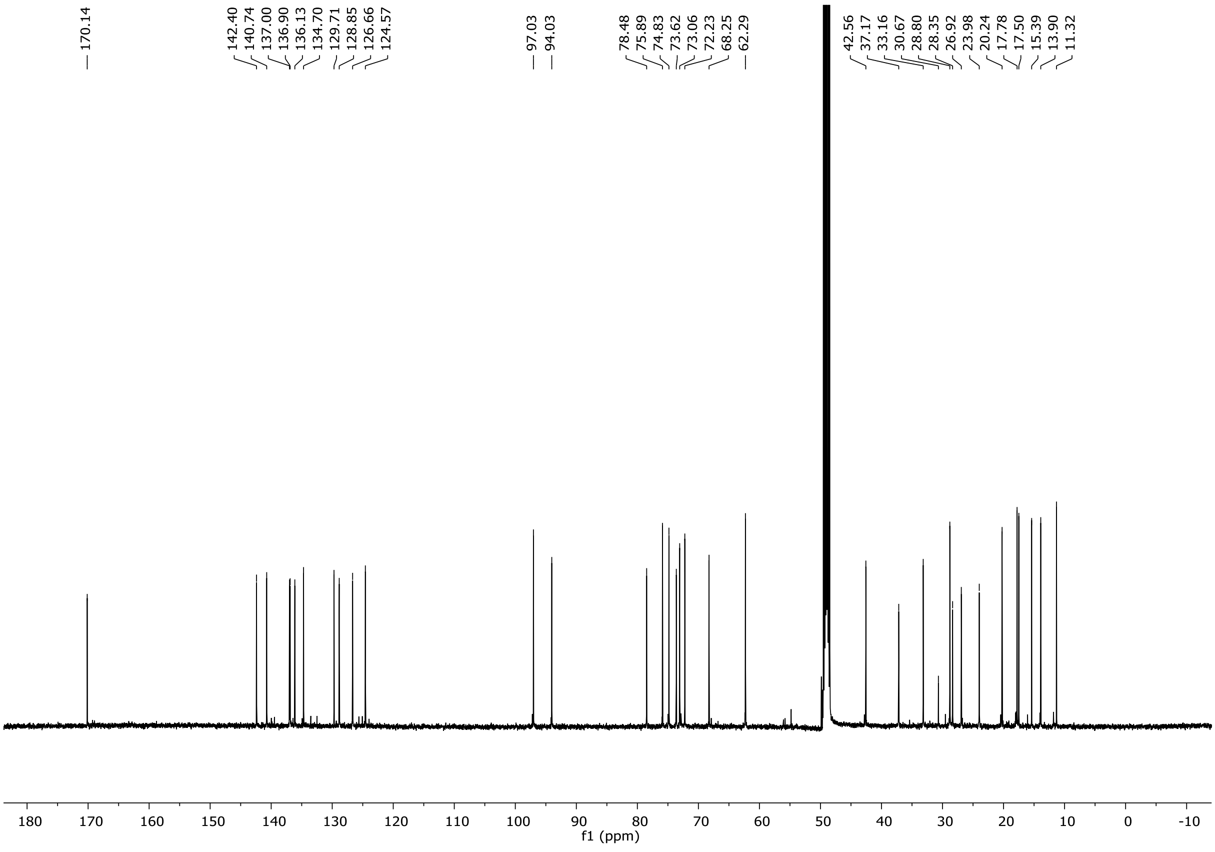

Supplementary Figure 92. ^{13}C NMR (126 MHz, CD_3OD) of **S2**.

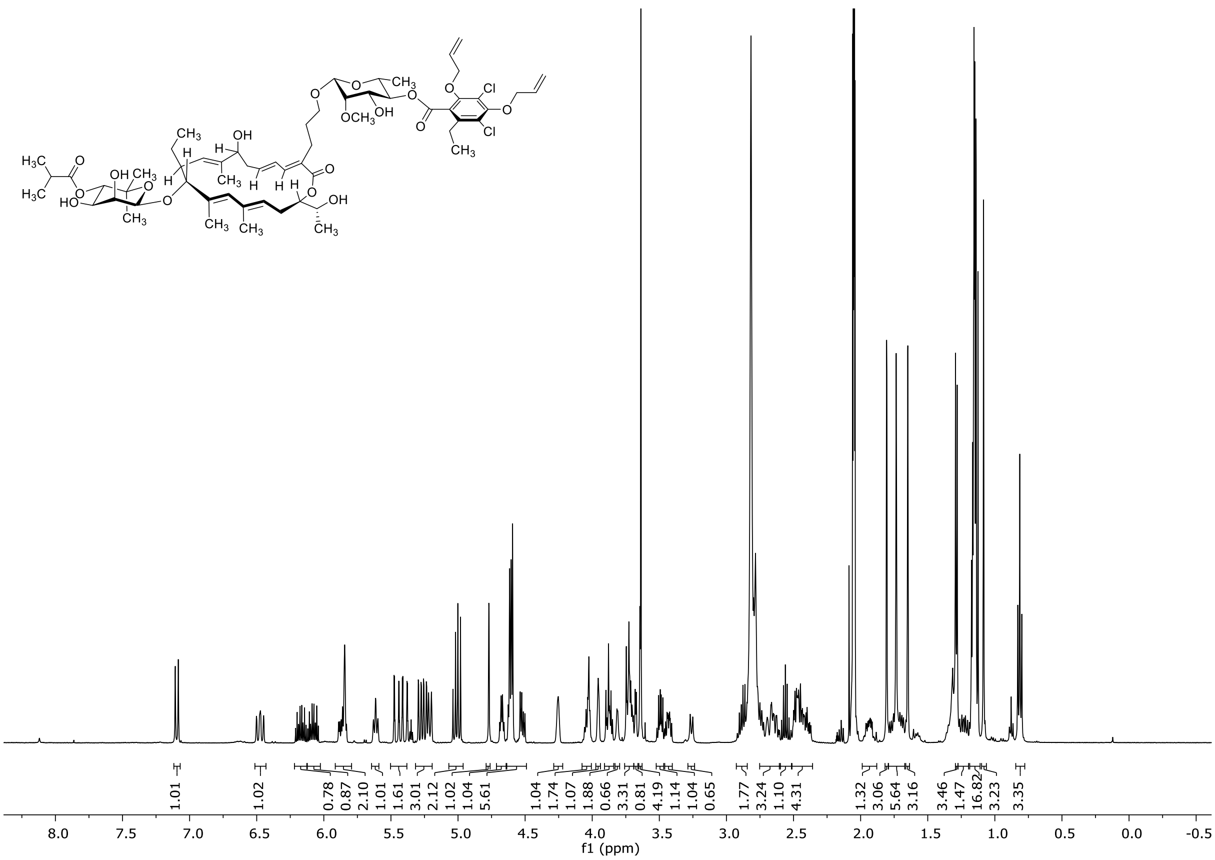

Supplementary Figure 93. ^{1}H NMR (500 MHz, acetone-d_6) of **S3.**

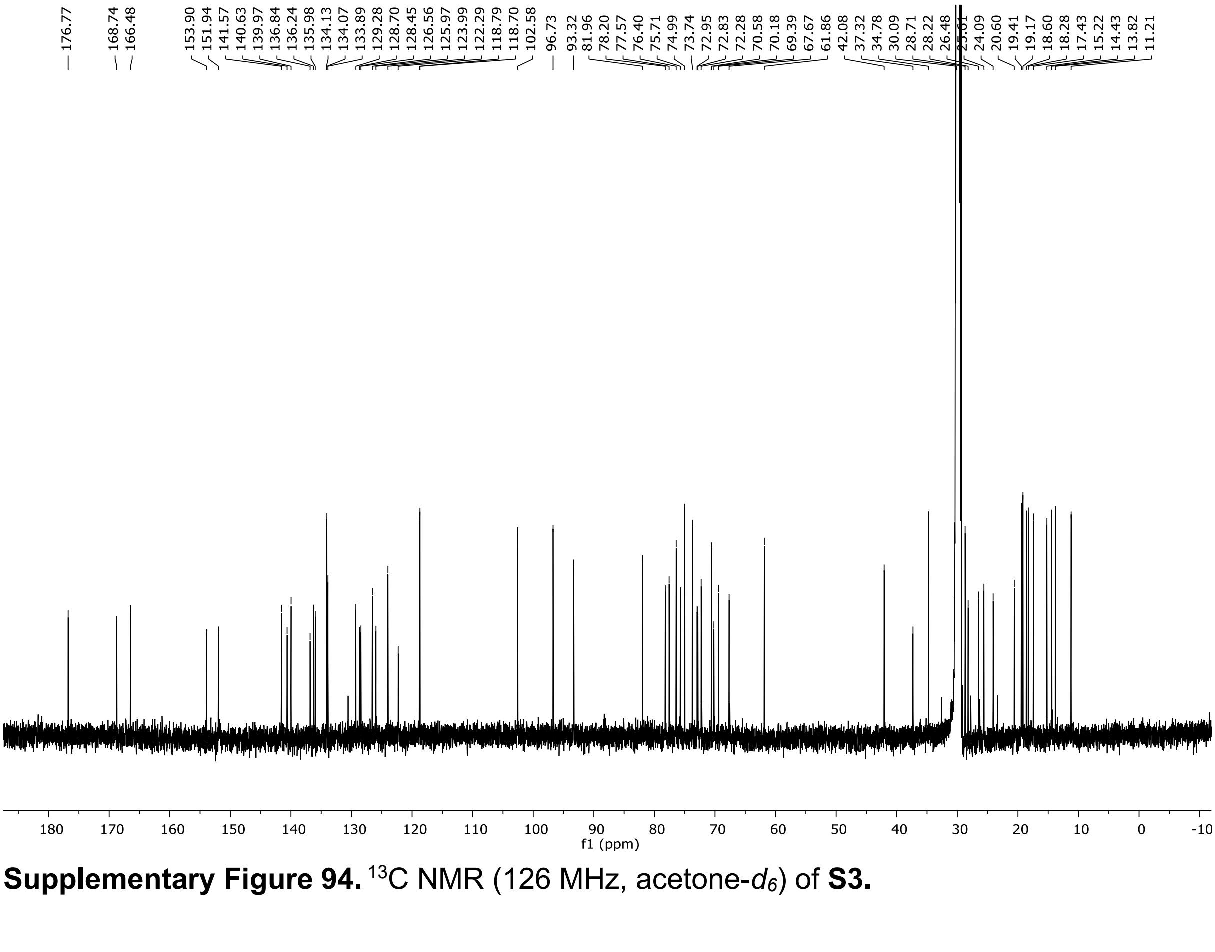

Supplementary Figure 94. ^{13}C NMR (126 MHz, acetone-d_6) of **S3.**

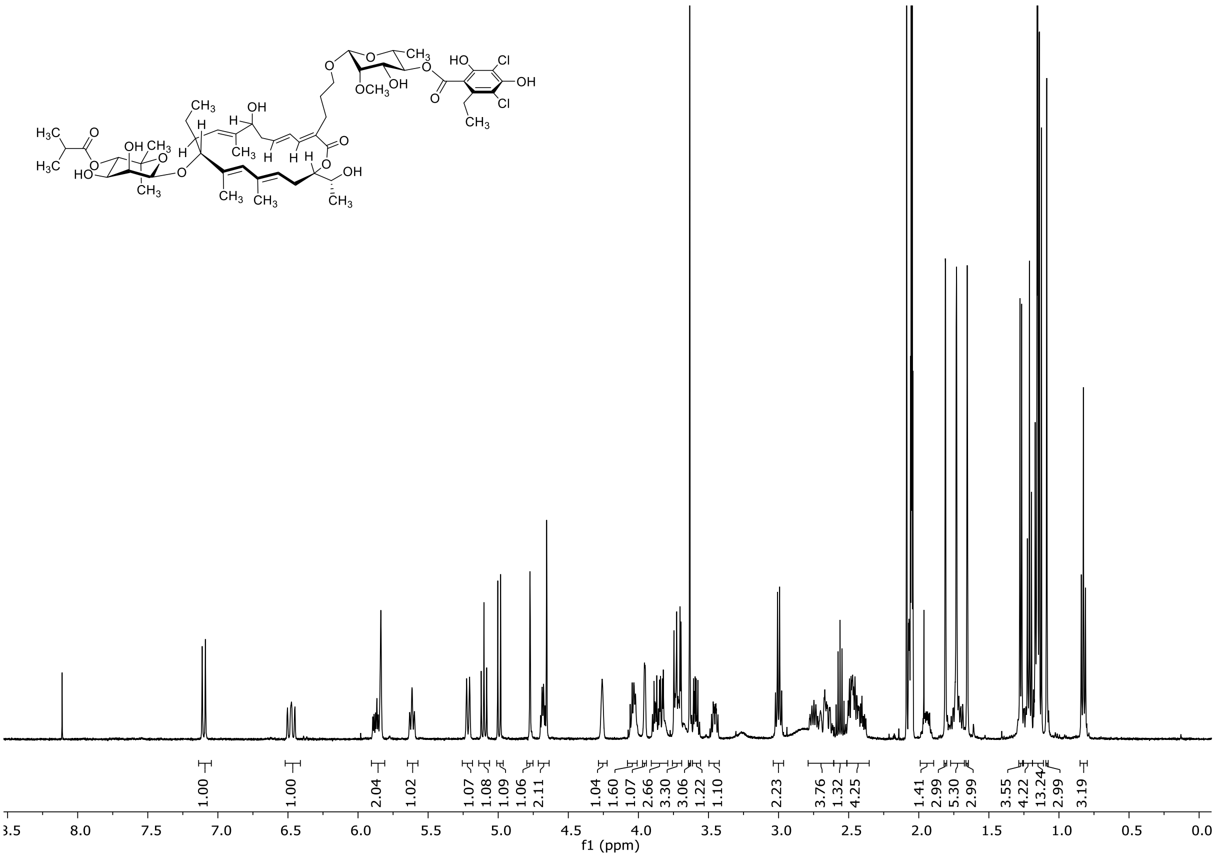

Supplementary Figure 95. ^{1}H NMR (500 MHz, acetone-d_6) of **17**.

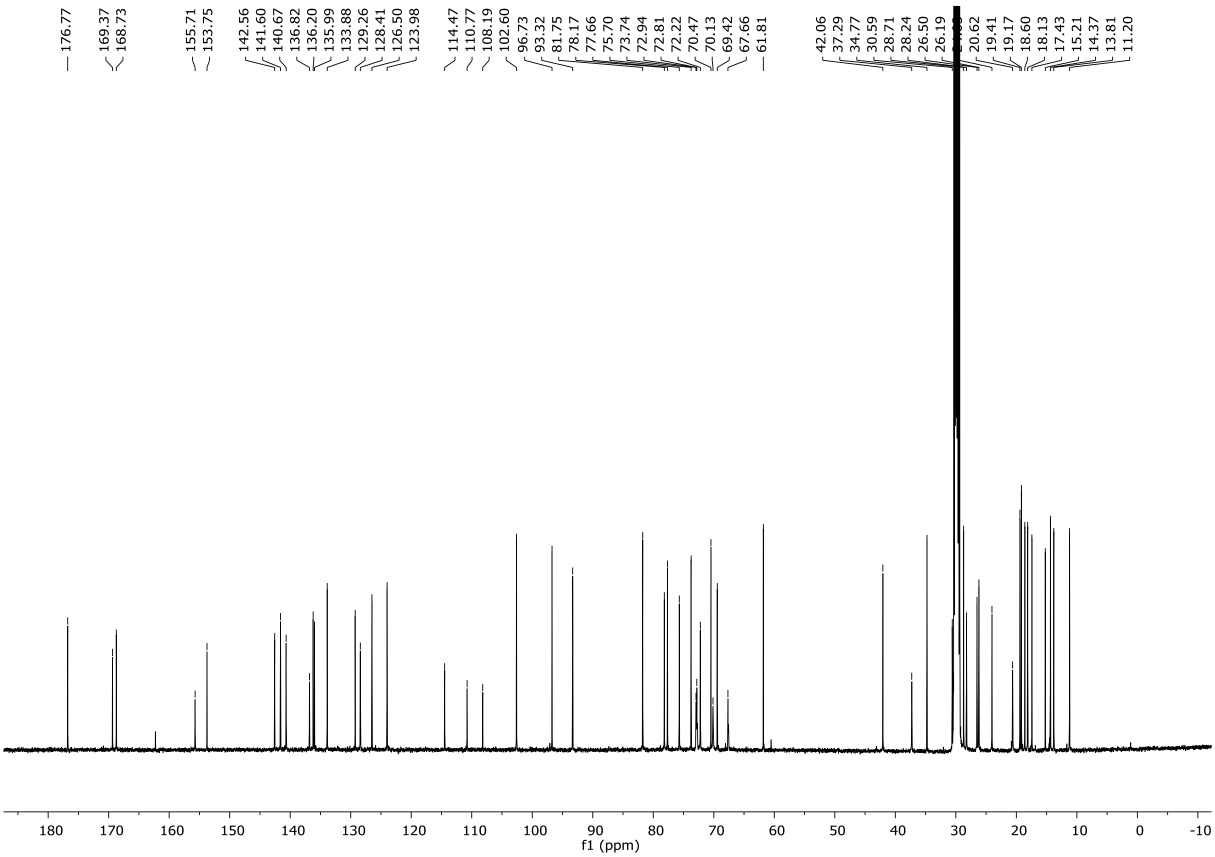

Supplementary Figure 96. ^{13}C NMR (126 MHz, acetone-d_6) of **17**.

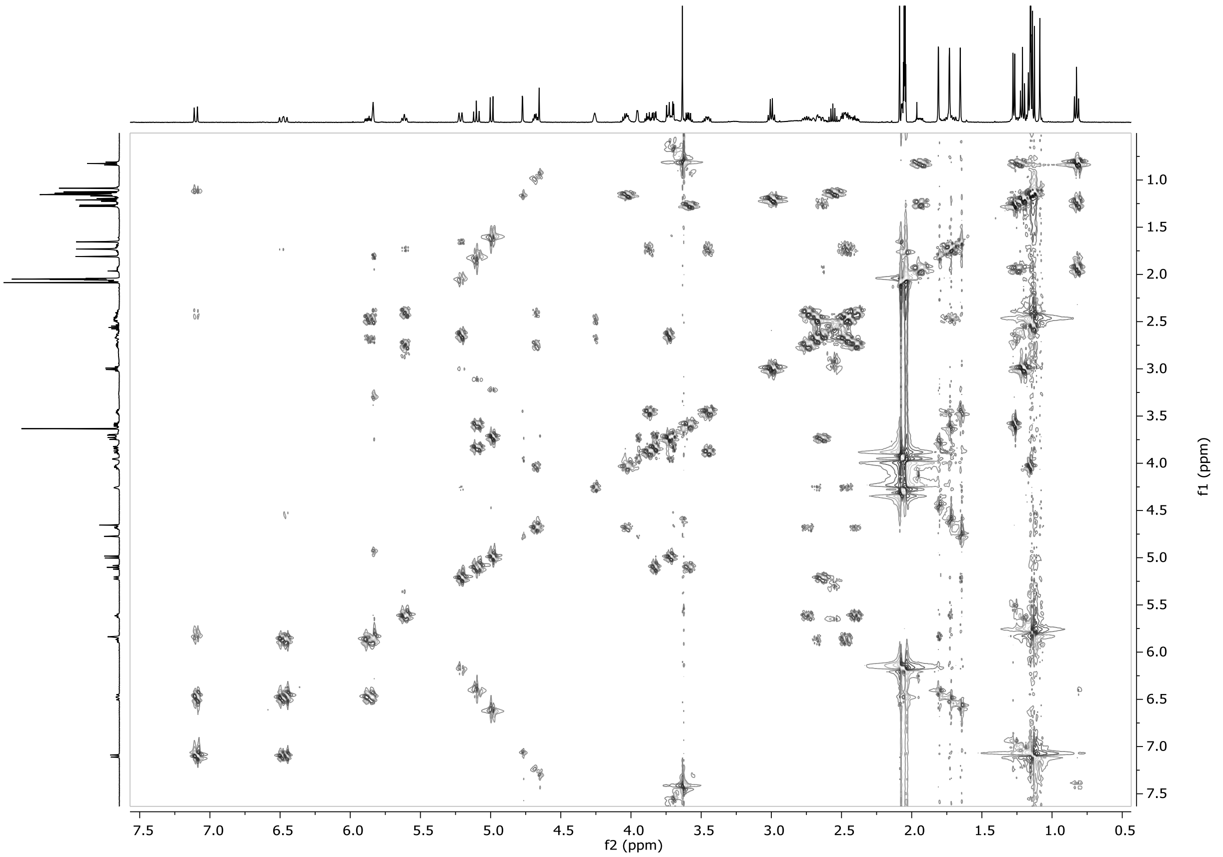

Supplementary Figure 97. COSY NMR (500 MHz, acetone-d_6) of **17**.

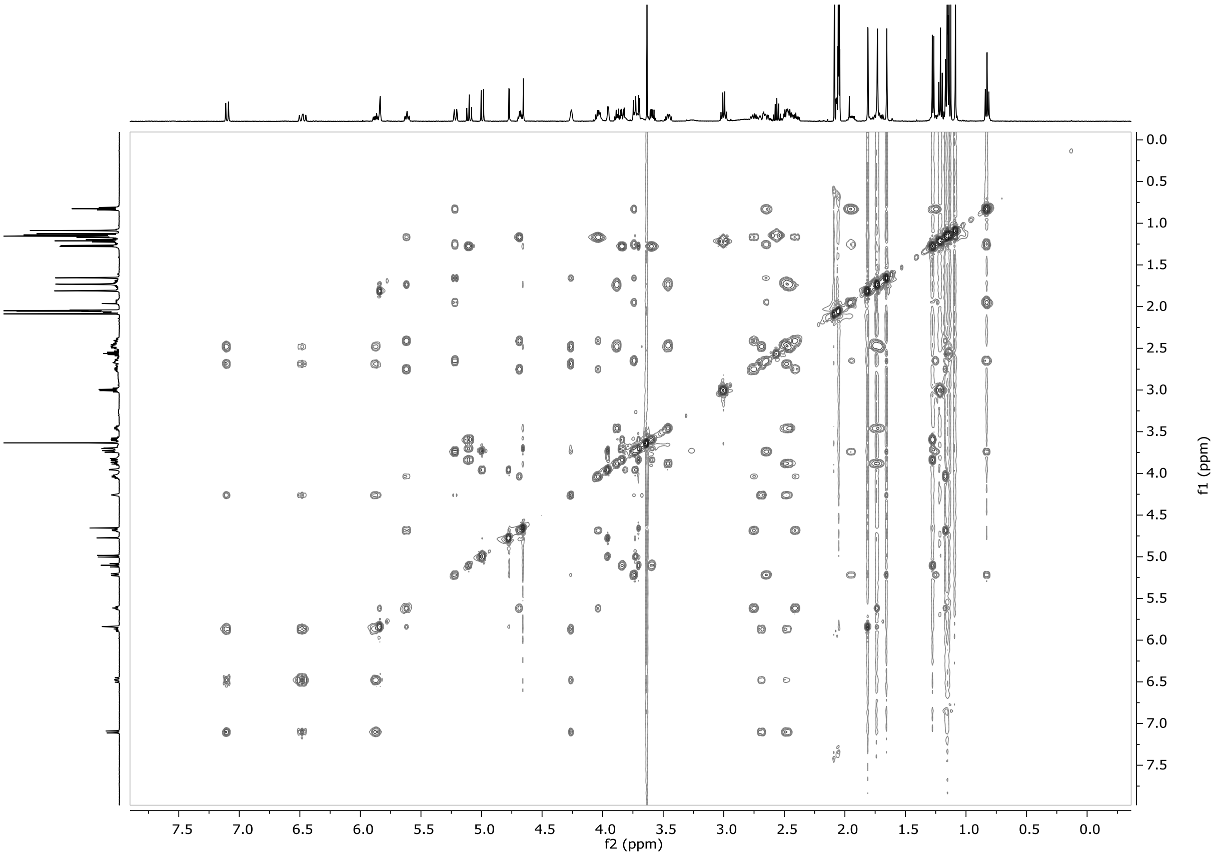

Supplementary Figure 98. TOCSY NMR (500 MHz, acetone-d_6) of **17**.

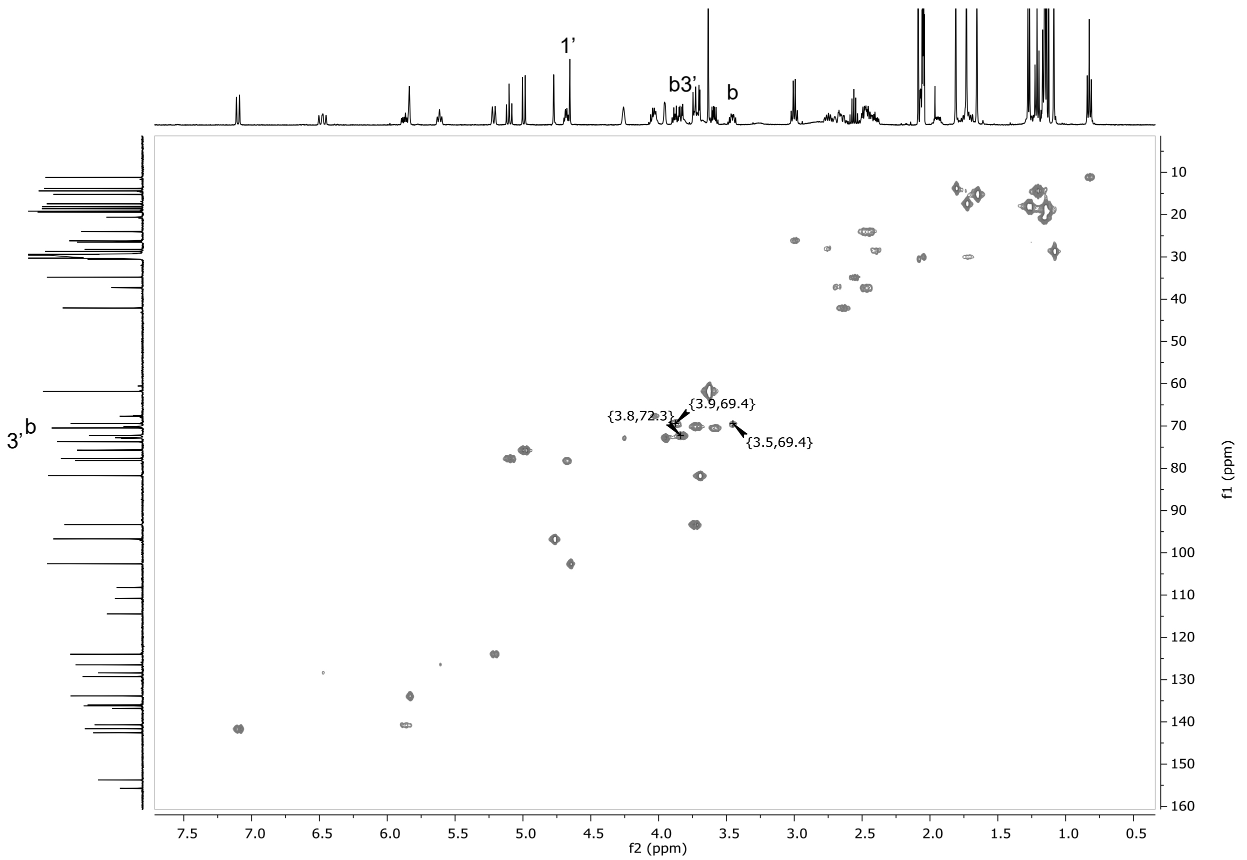

Supplementary Figure 99. HSQC NMR (500 MHz, 126 MHz, acetone-d_6) of **17.**

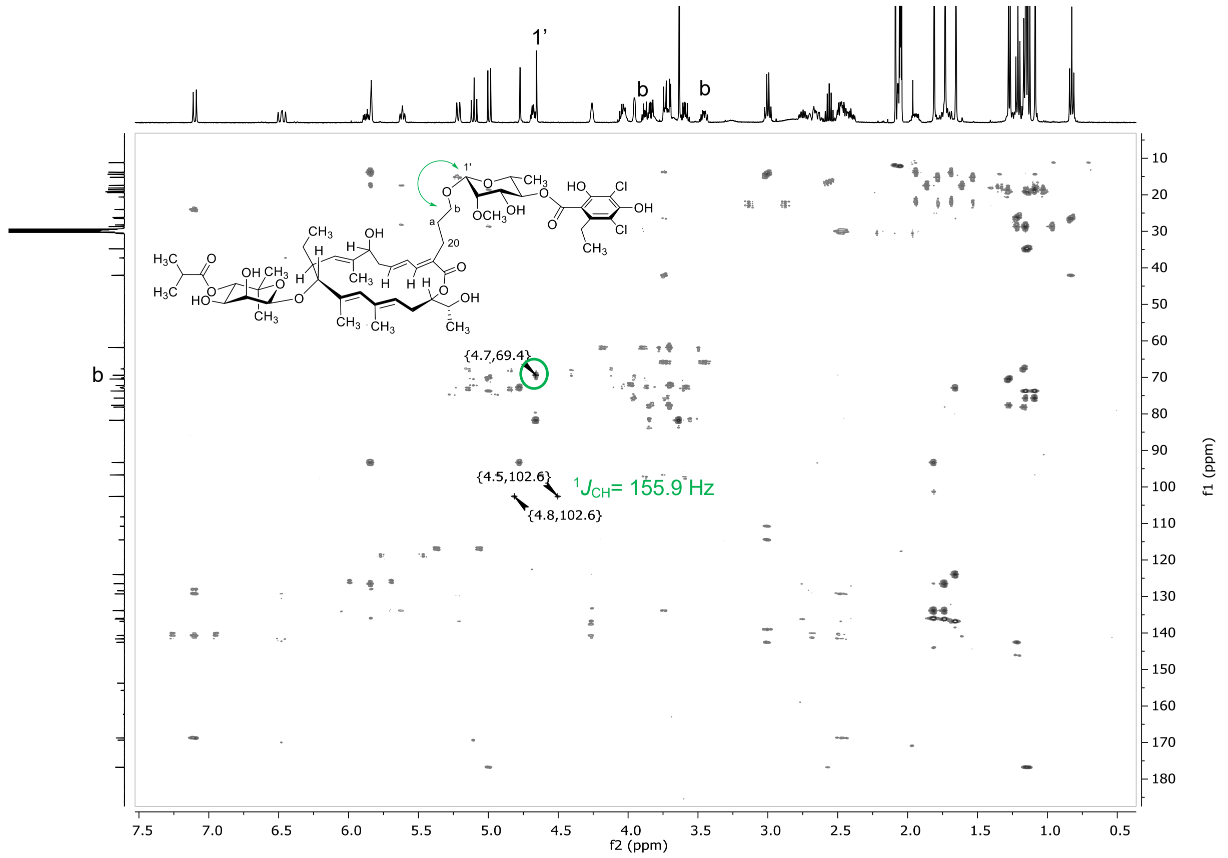

Supplementary Figure 100. HMBC NMR (500 MHz, 126 MHz, acetone-d_6) of **17.**

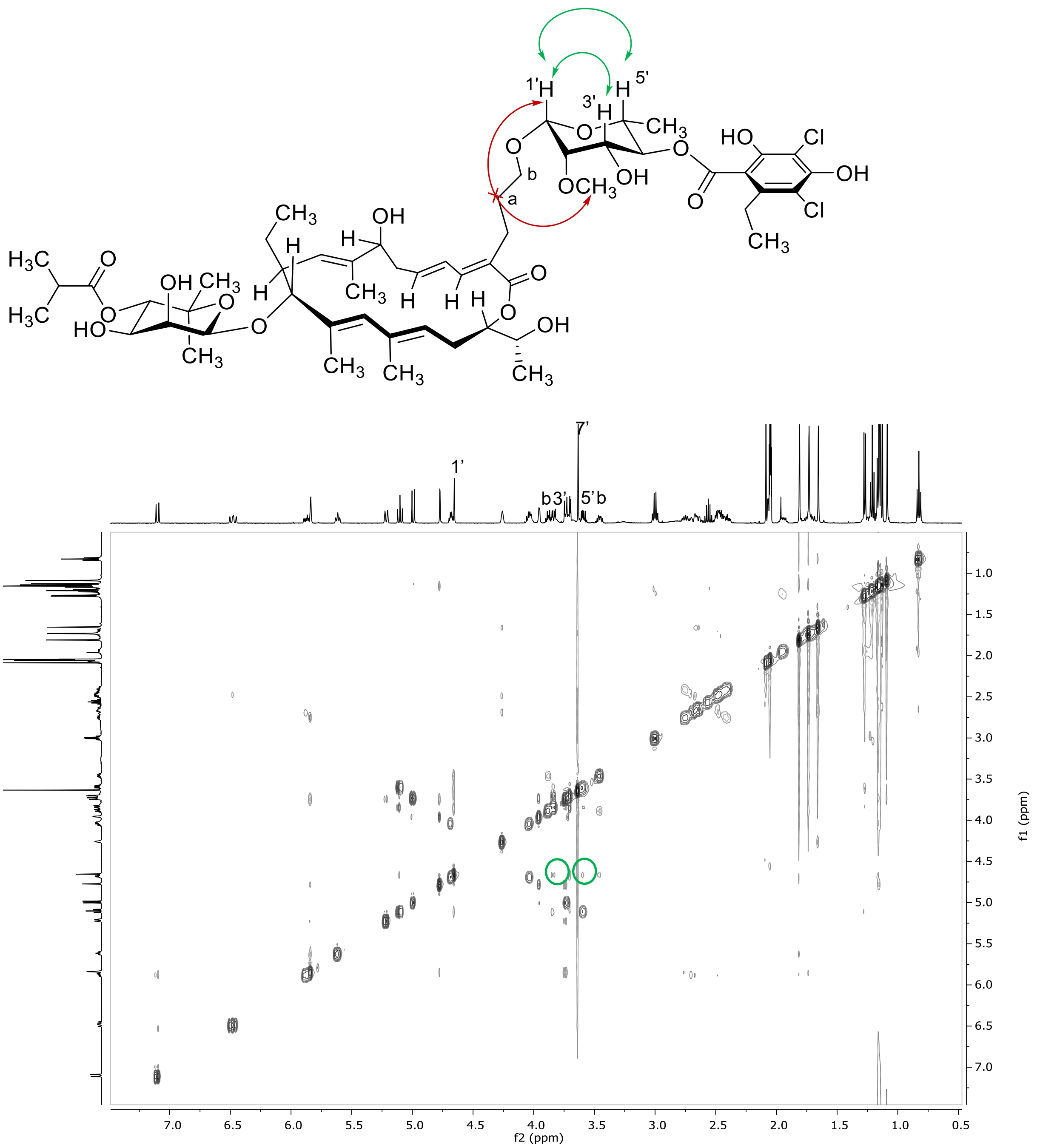

Supplementary Figure 101. ROESY NMR (500 MHz, acetone-d_6) of **17.**

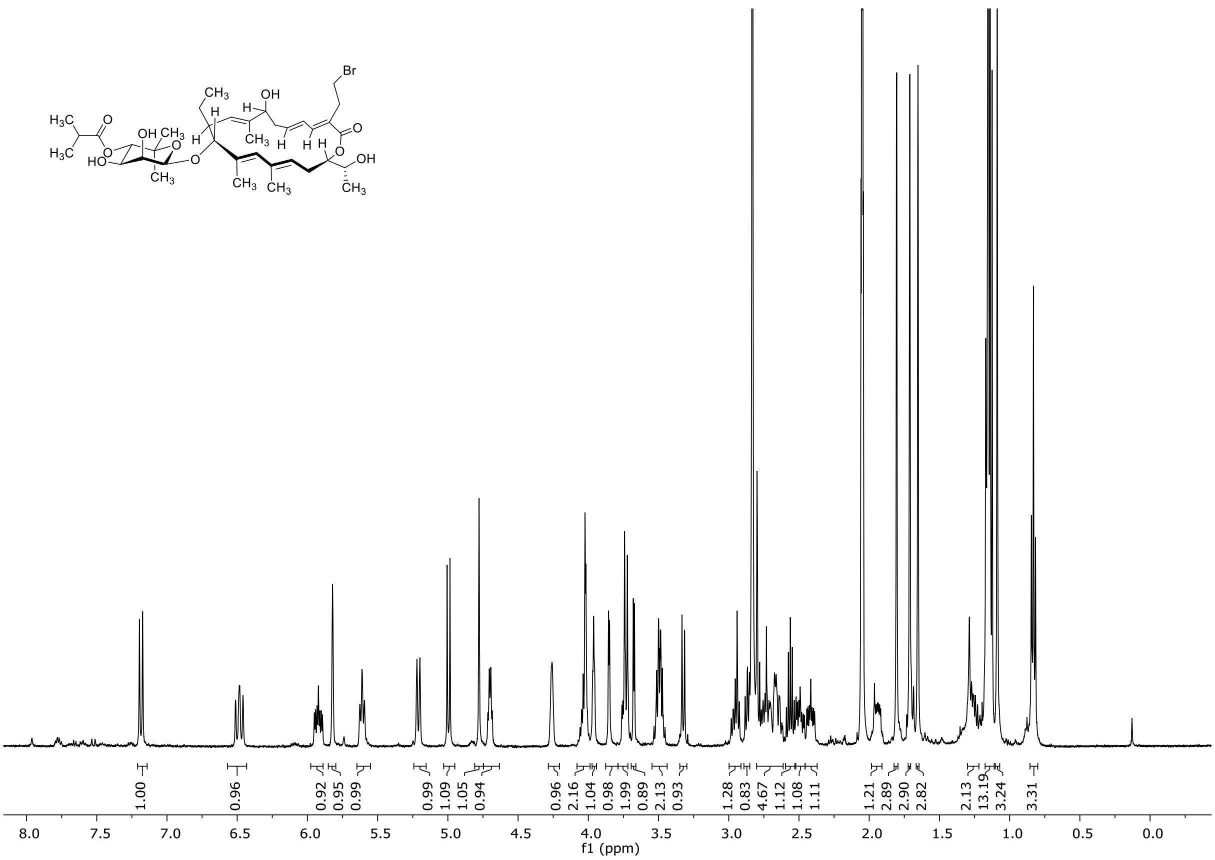

Supplementary Figure 102. ^{1}H NMR (500 MHz, acetone-d_6) of **16.**

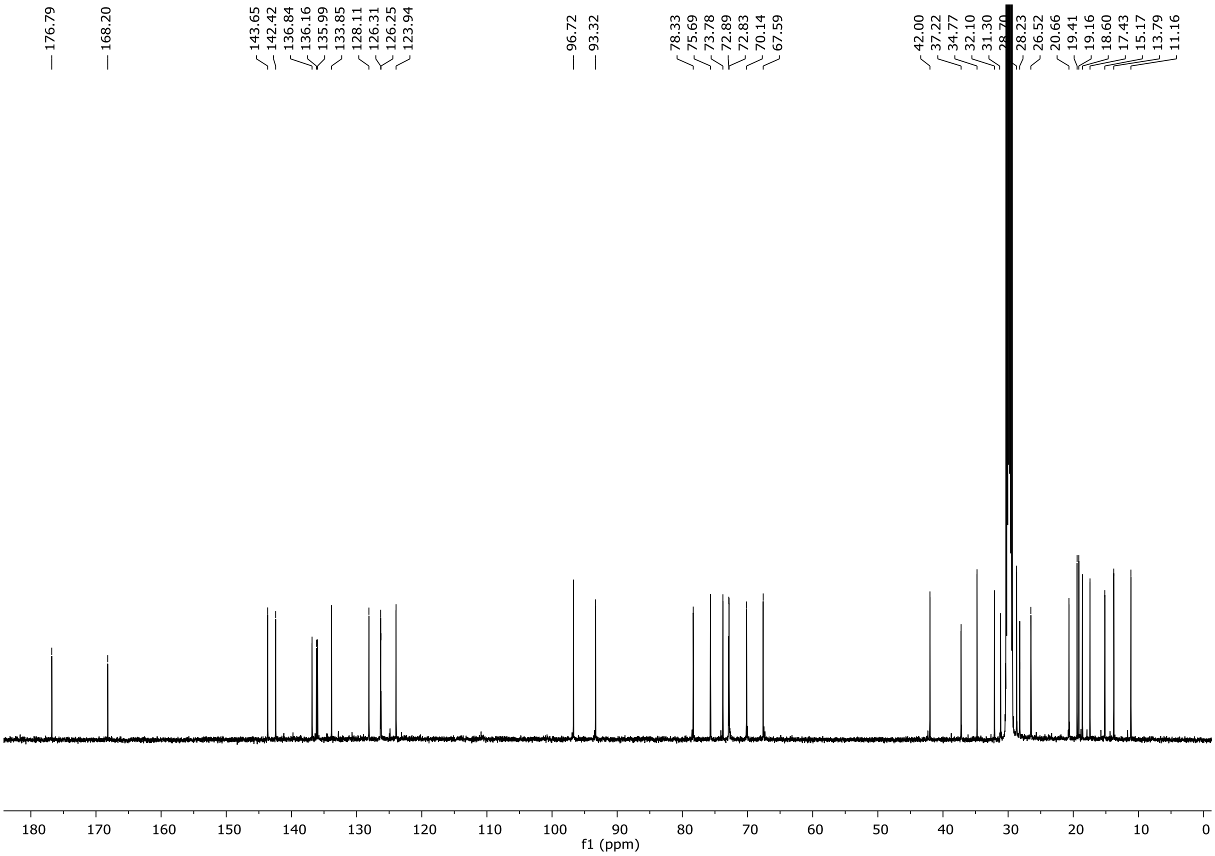

Supplementary Figure 103. ^{13}C NMR (126 MHz, acetone-d_6) of **16.**

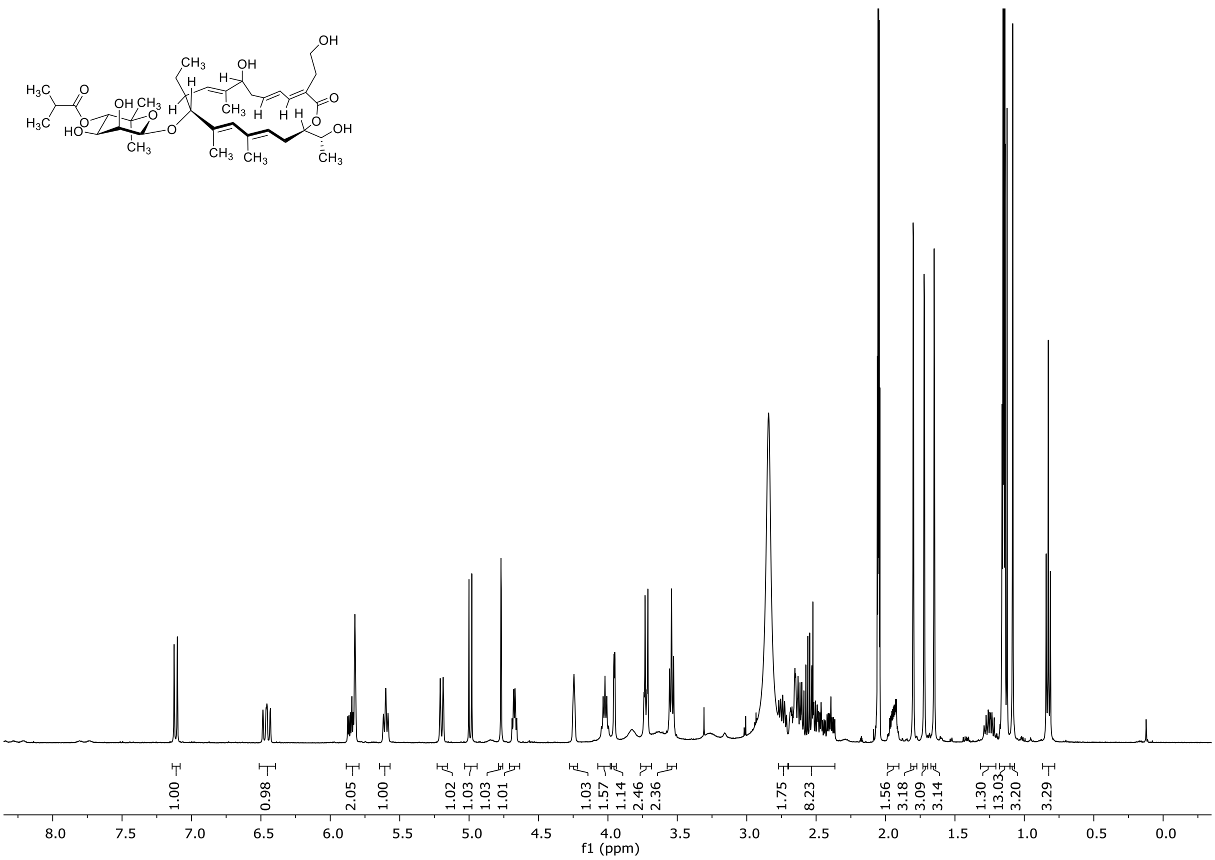

Supplementary Figure 104. ^{1}H NMR (500 MHz, acetone-d_6) of **16.1.**

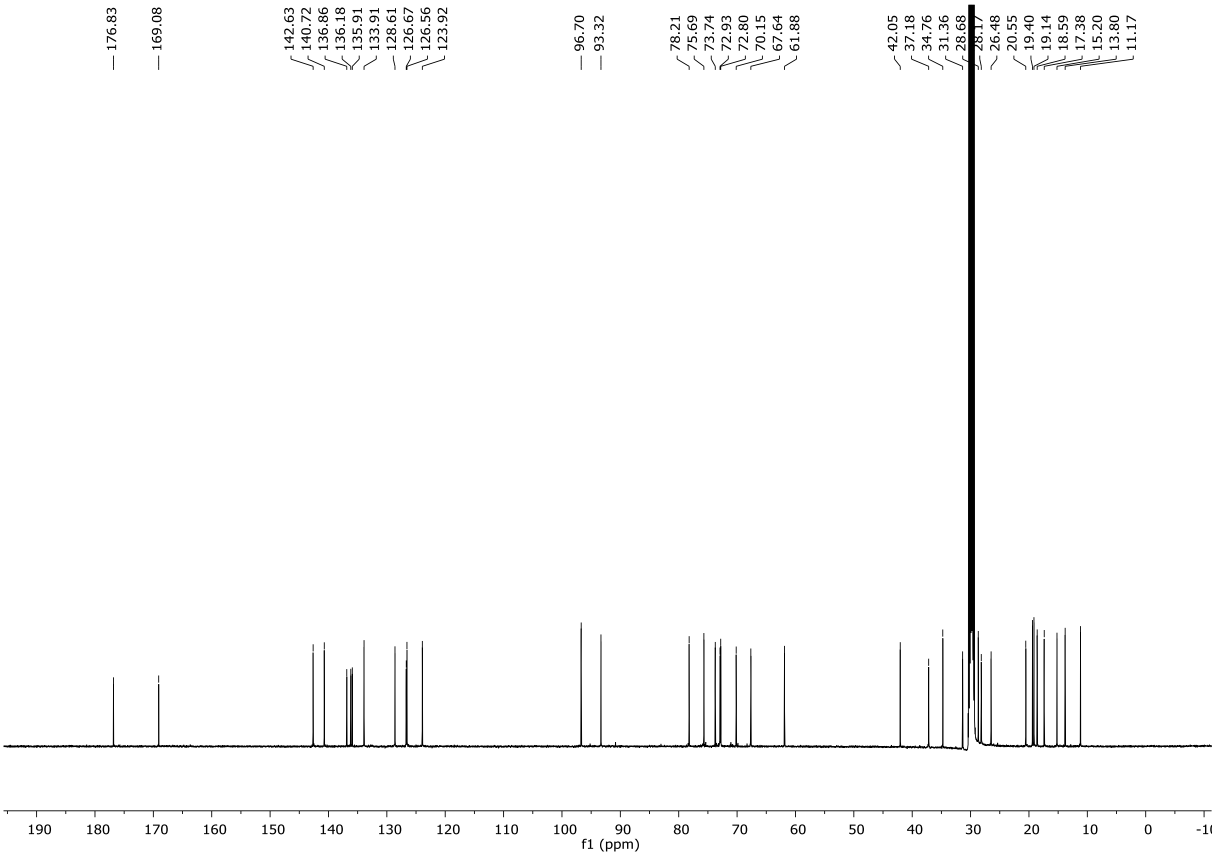

Supplementary Figure 105. ^{13}C NMR (126 MHz, acetone-d_6) of **16.1.**

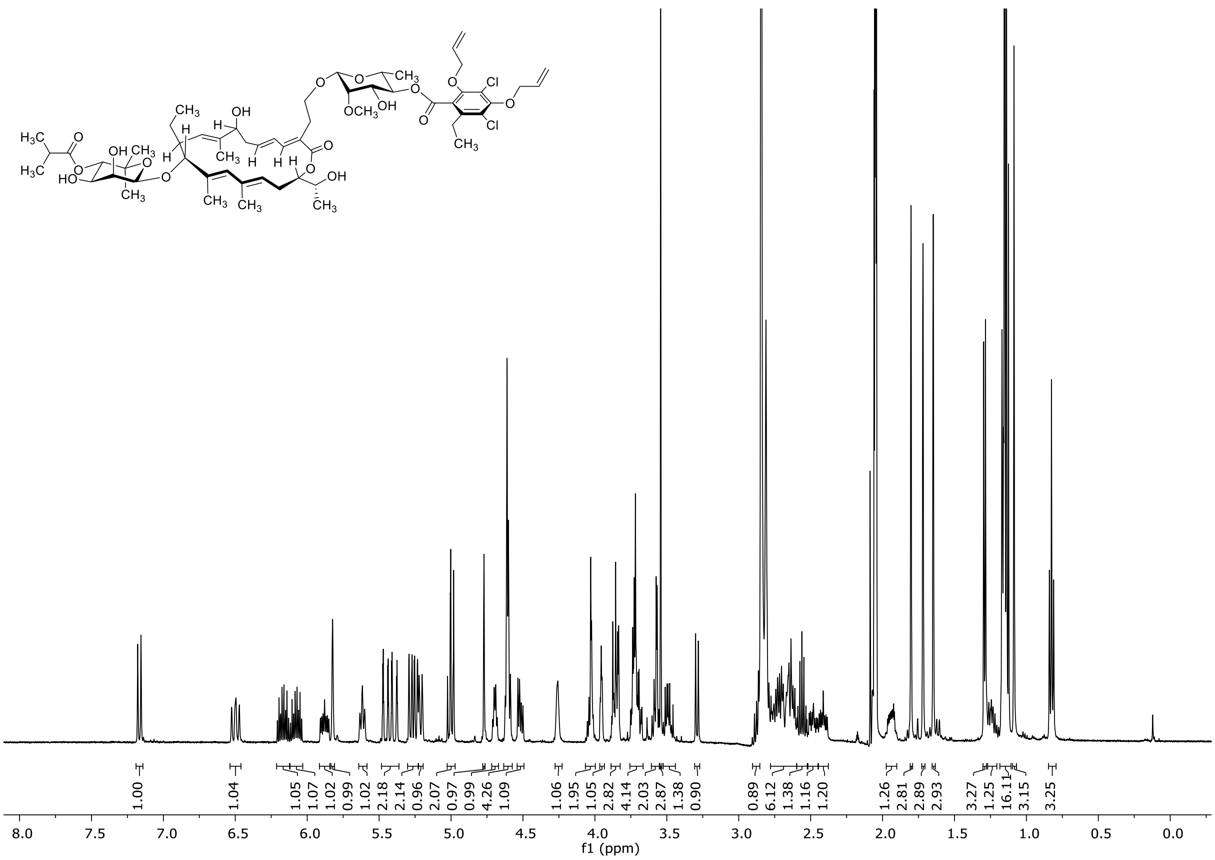

Supplementary Figure 106. ^{1}H NMR (500 MHz, acetone-d_6) of **S4.**

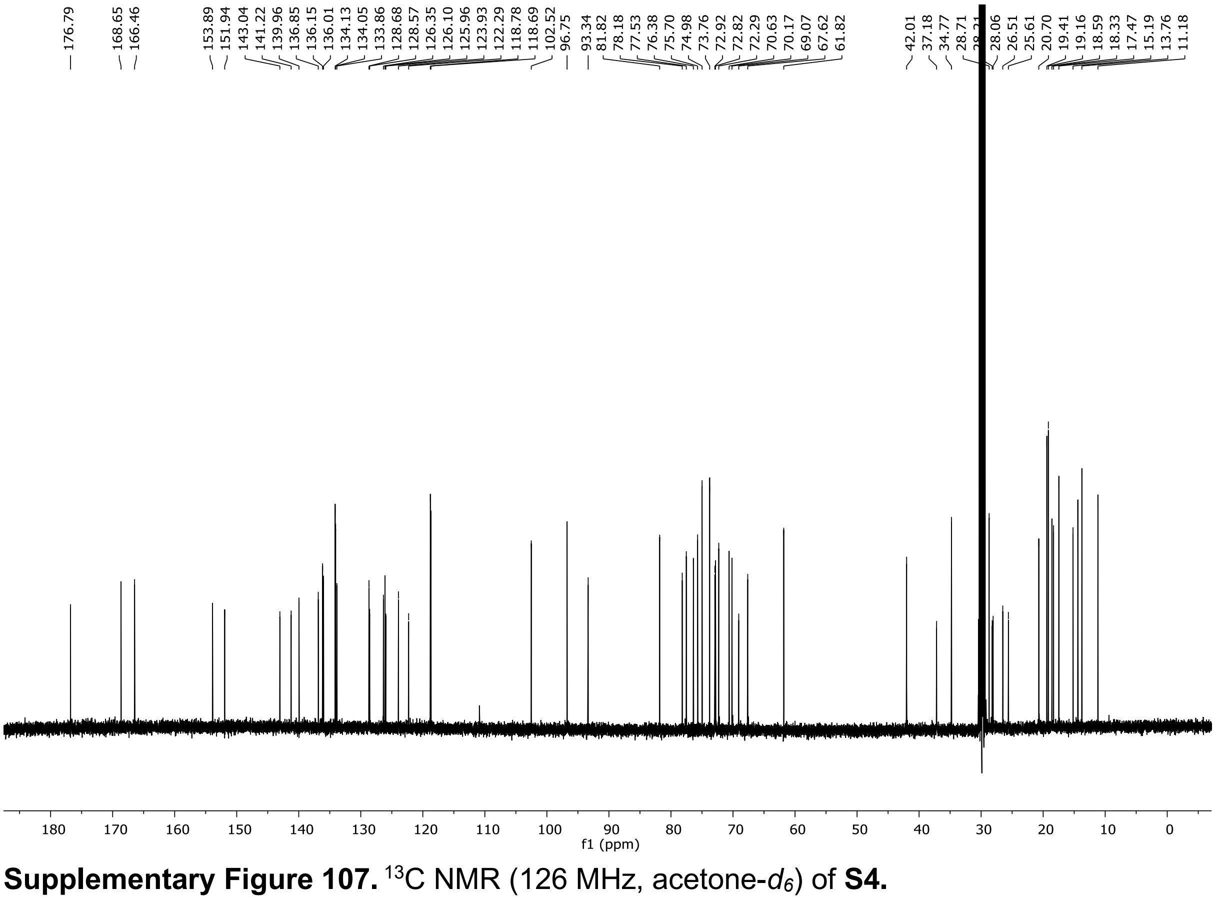

Supplementary Figure 107. ^{13}C NMR (126 MHz, acetone-d_6) of **S4.**

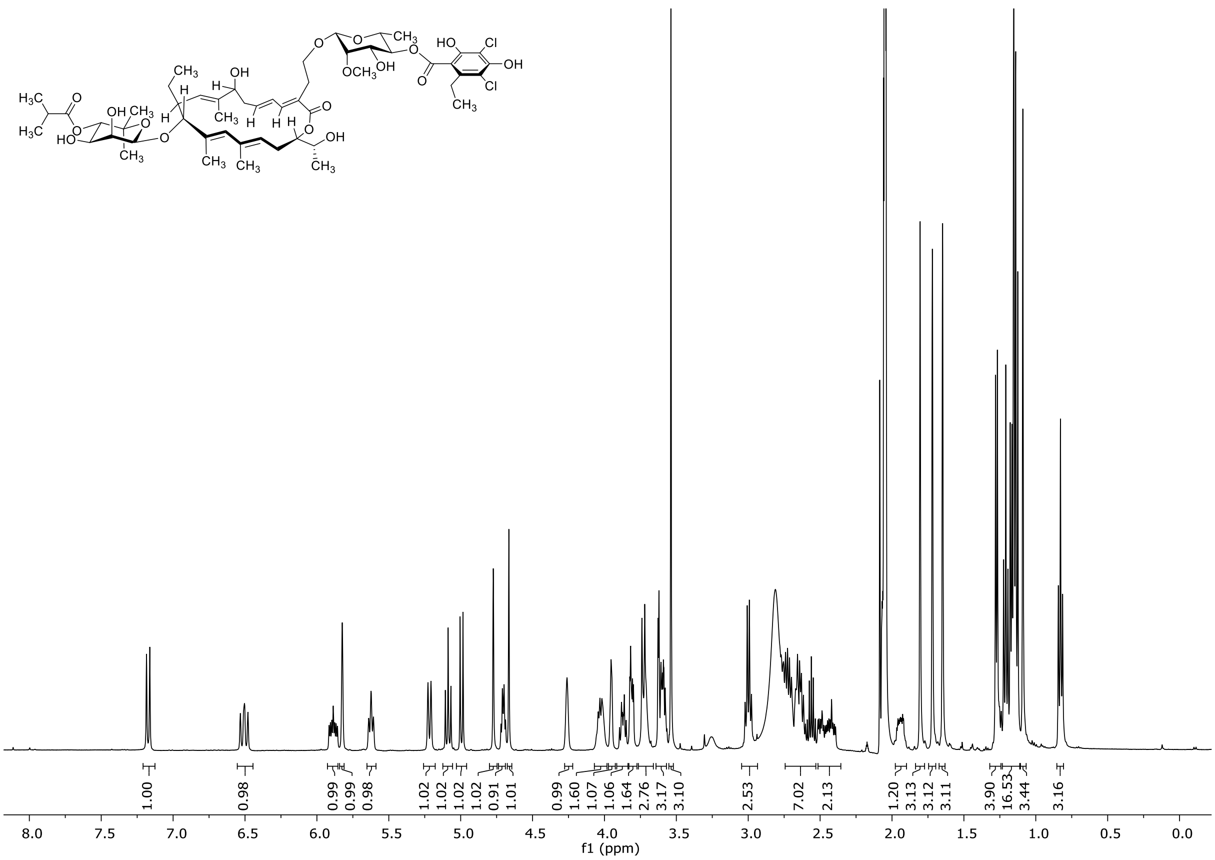

Supplementary Figure 108. ^{1}H NMR (500 MHz, acetone-d_6) of **18.**

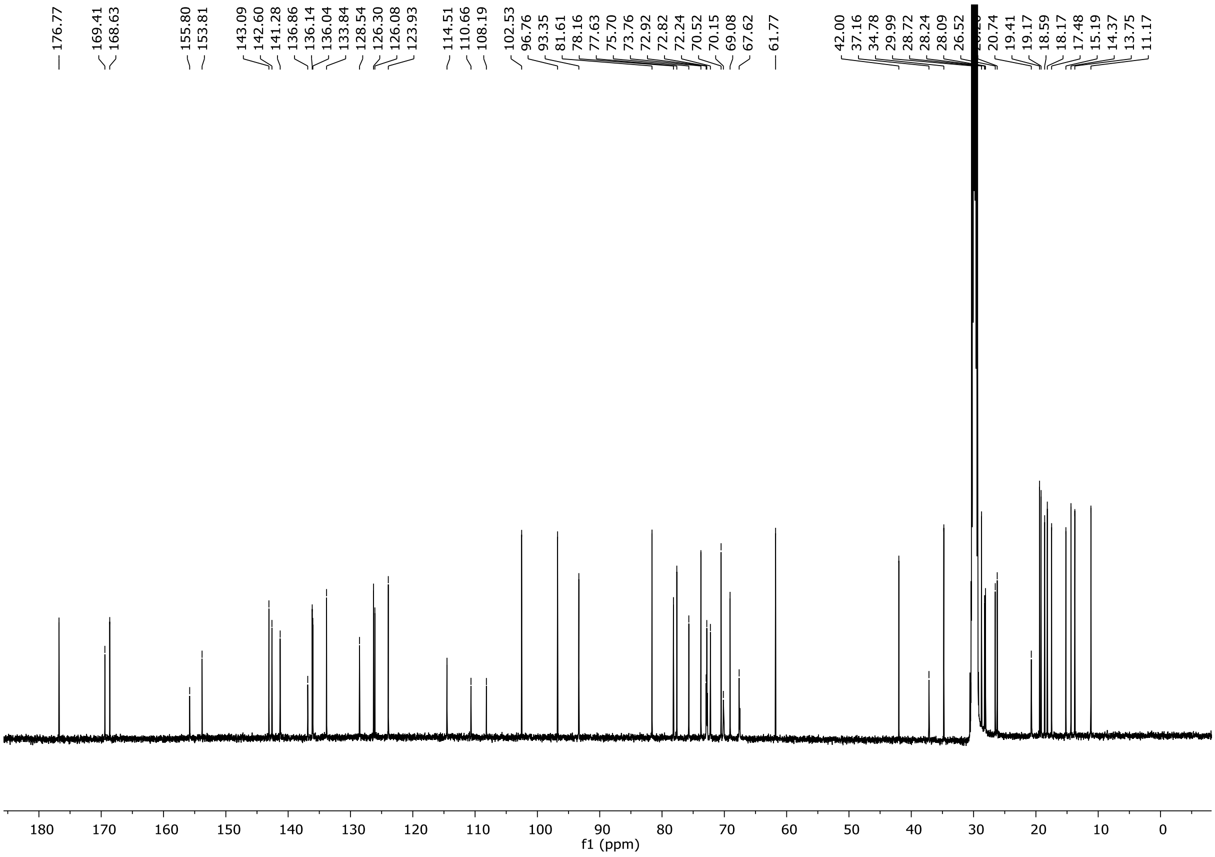

Supplementary Figure 109. ^{13}C NMR (126 MHz, acetone-d_6) of **18**.

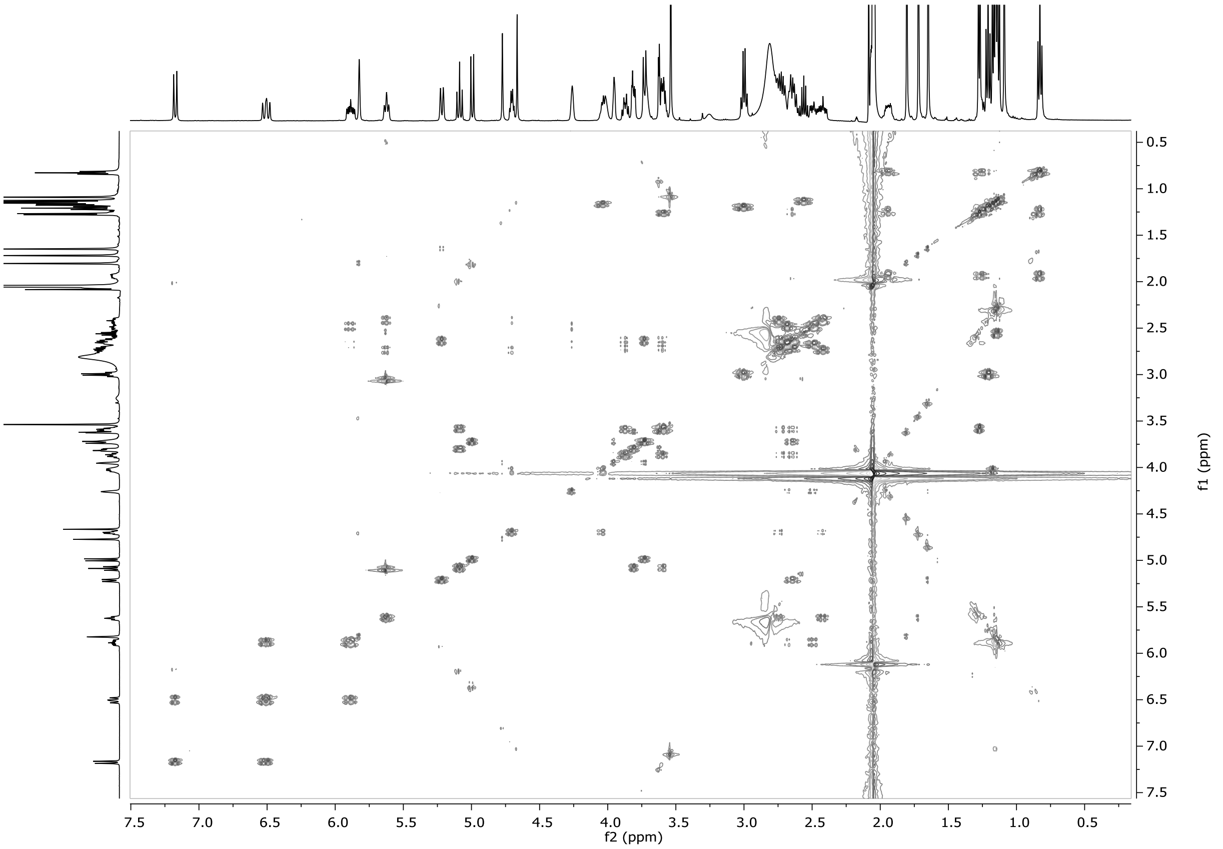

Supplementary Figure 110. COSY NMR (500 MHz, acetone-d_6) of **18.**

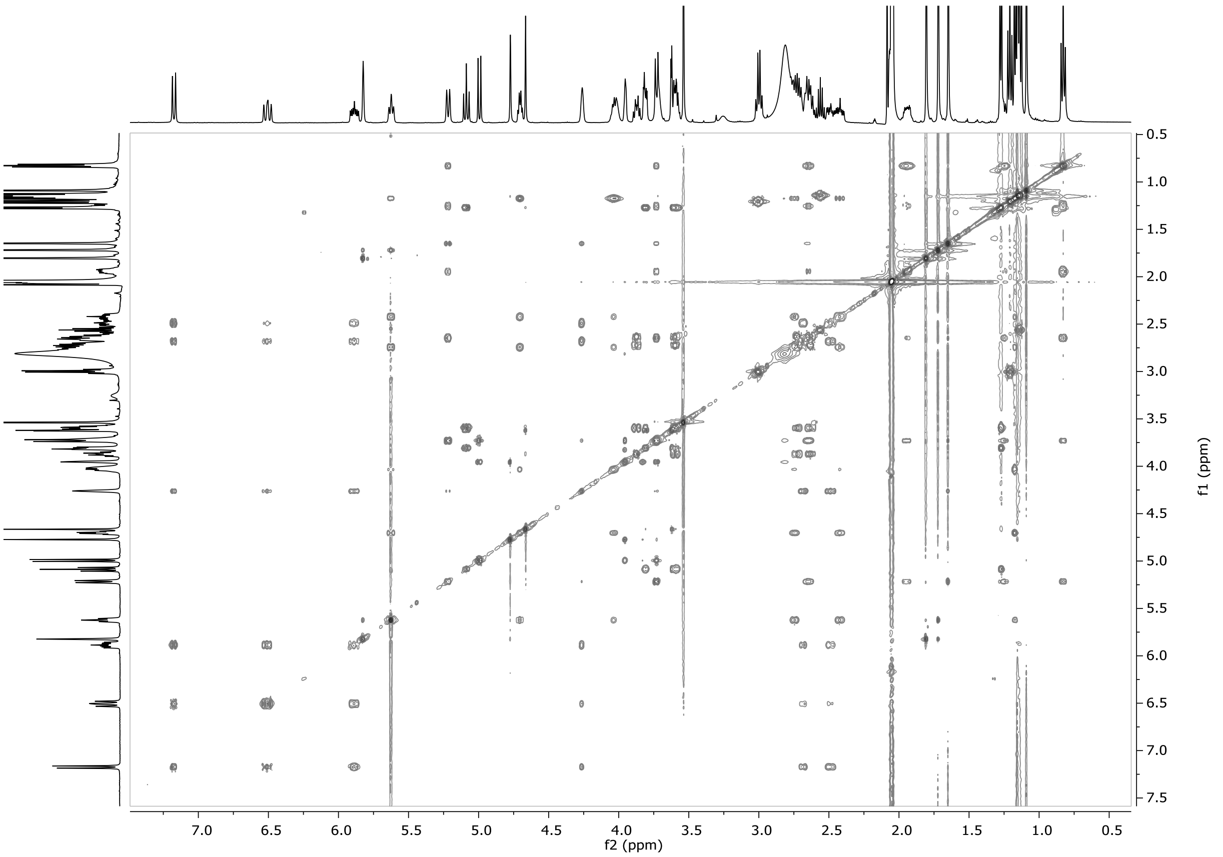

Supplementary Figure 111. TOCSY NMR (500 MHz, acetone-d_6) of **18.**

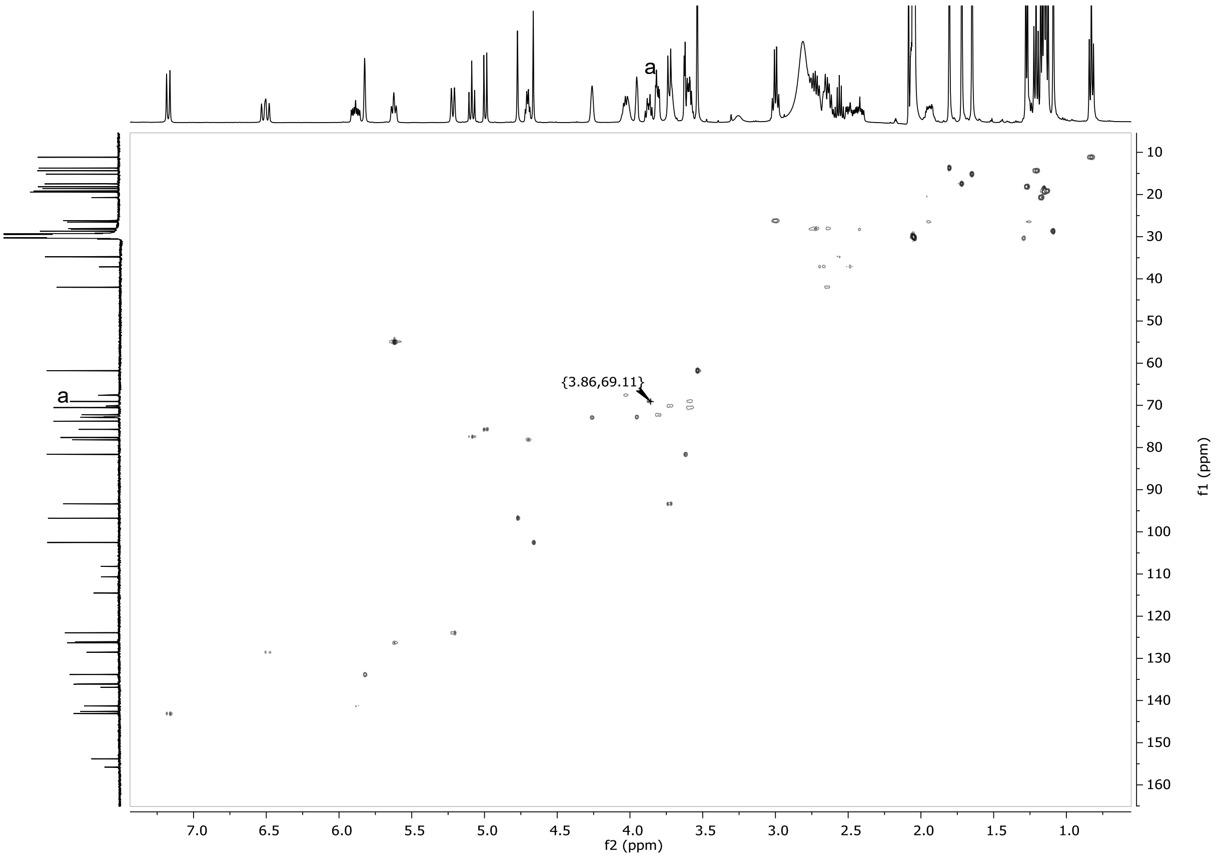

Supplementary Figure 112. HSQC (500 MHz, 126 MHz acetone-d_6) of **18.**

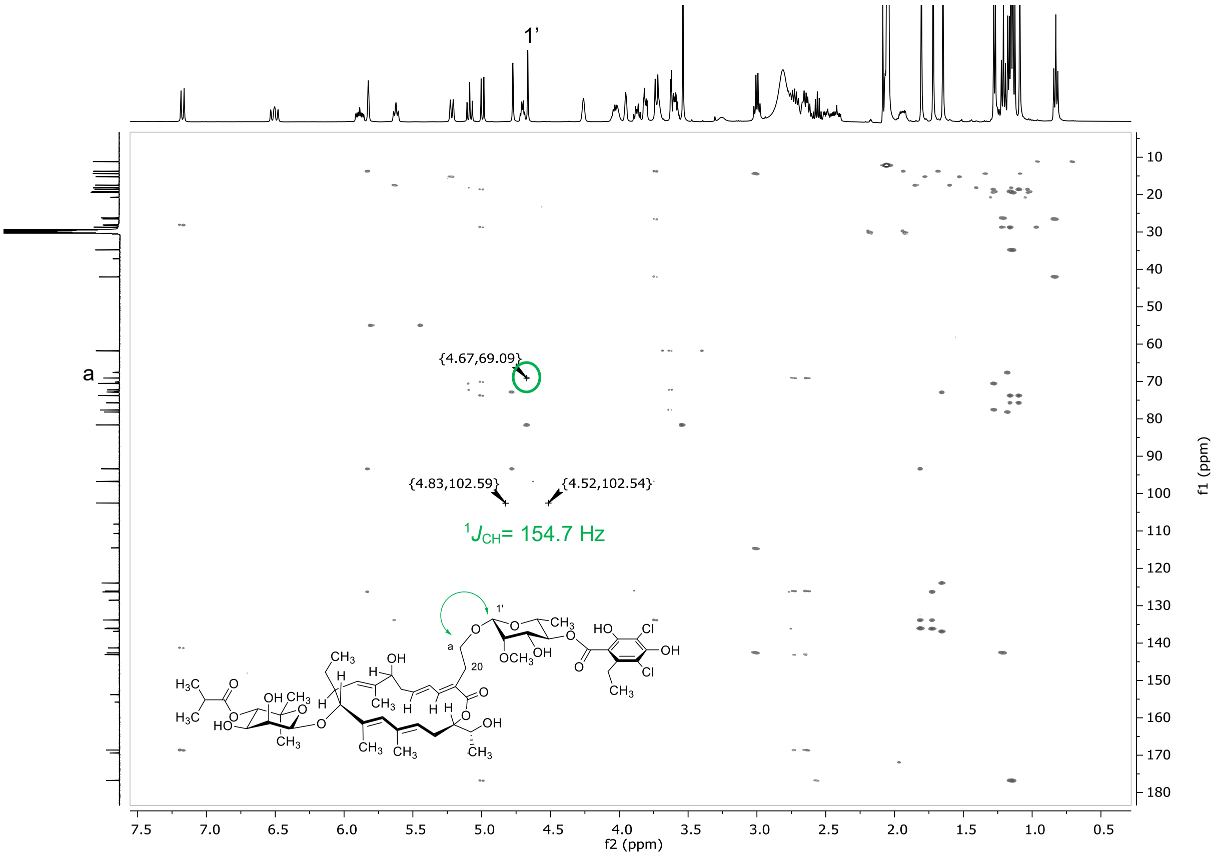

Supplementary Figure 113. HMBC (500 MHz, 126 MHz acetone-d_6) of **18.**

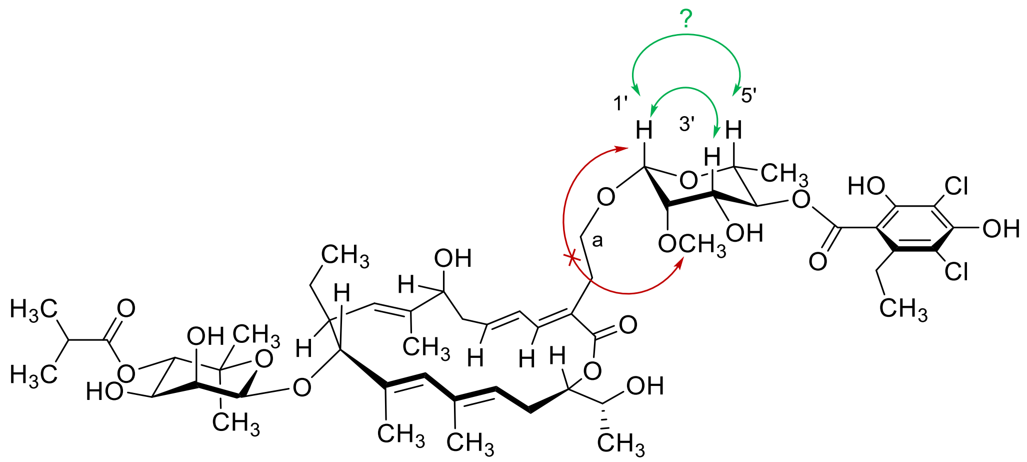

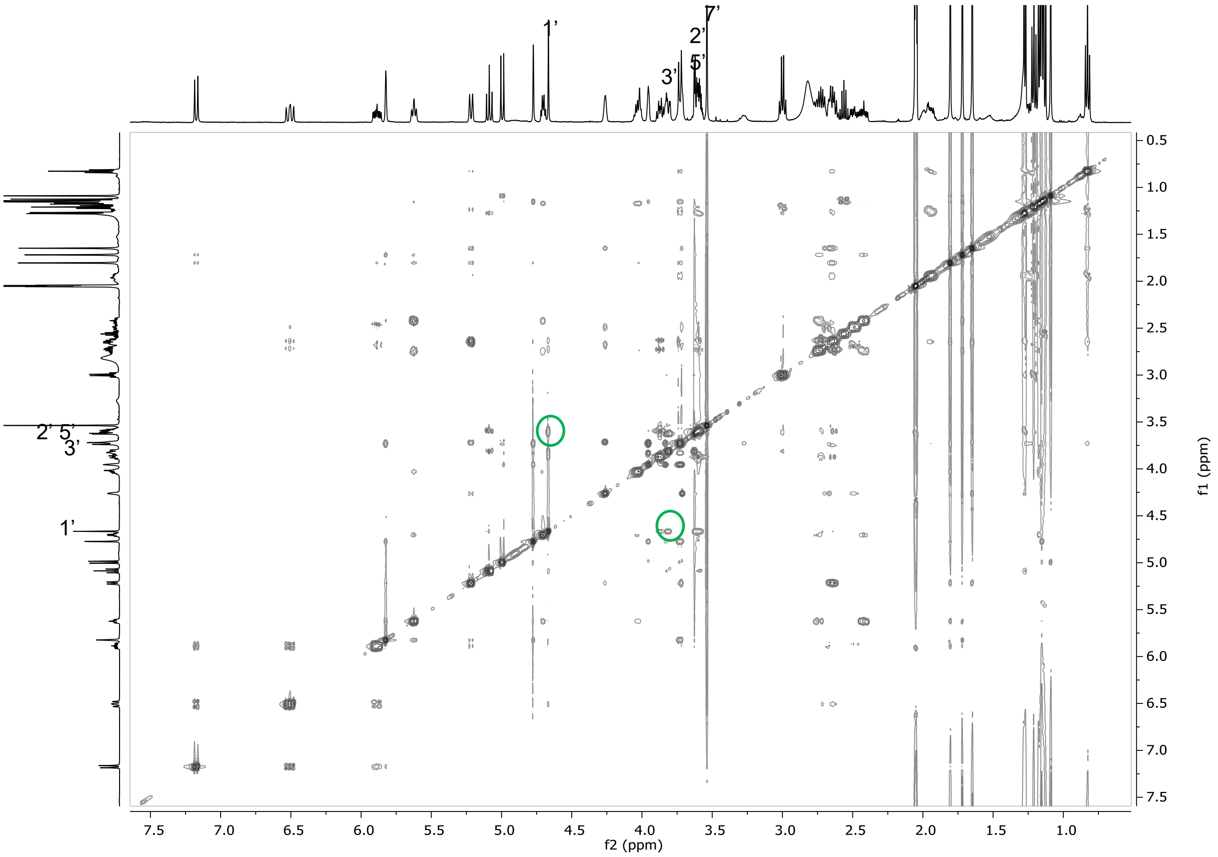

Supplementary Figure 114. ROESY (500 MHz, acetone-d_6) of **18.**

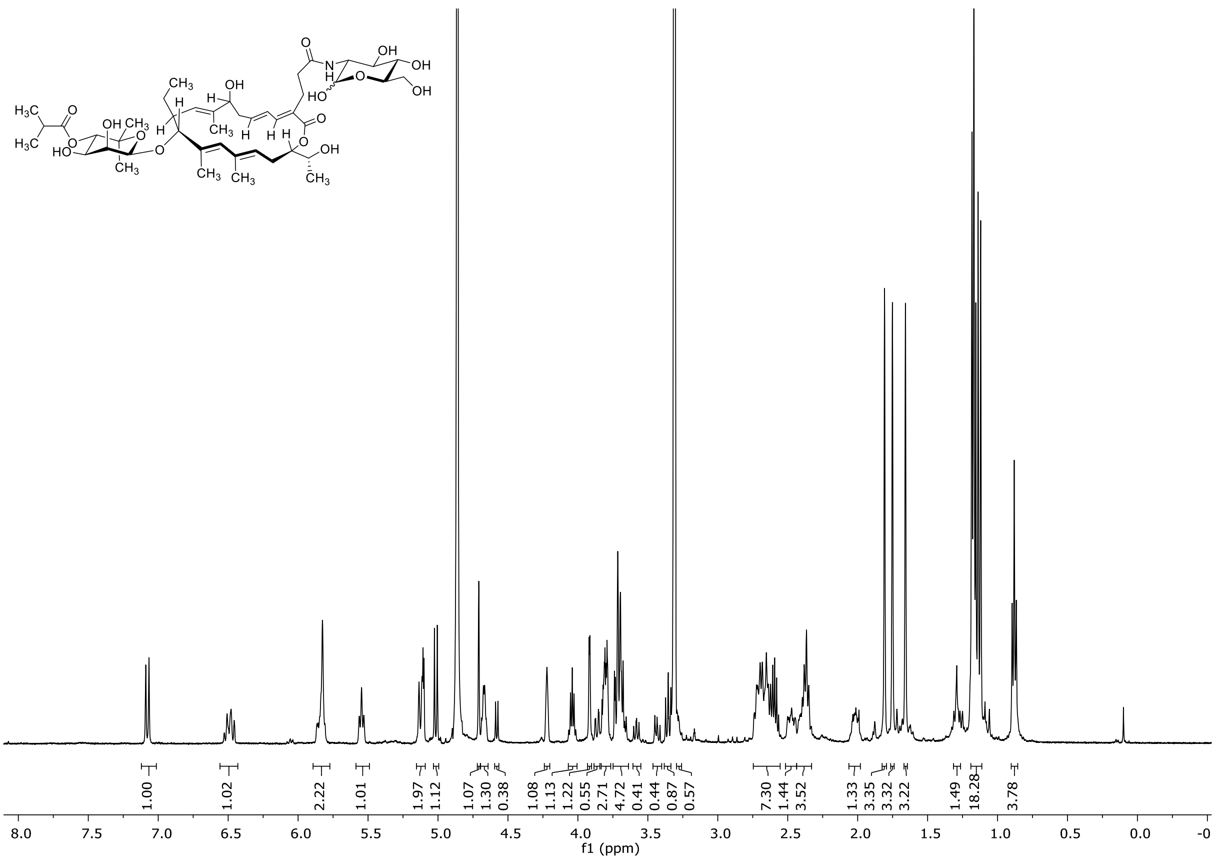

Supplementary Figure 115. ^{1}H NMR (500 MHz, CD_3OD) of **19.**

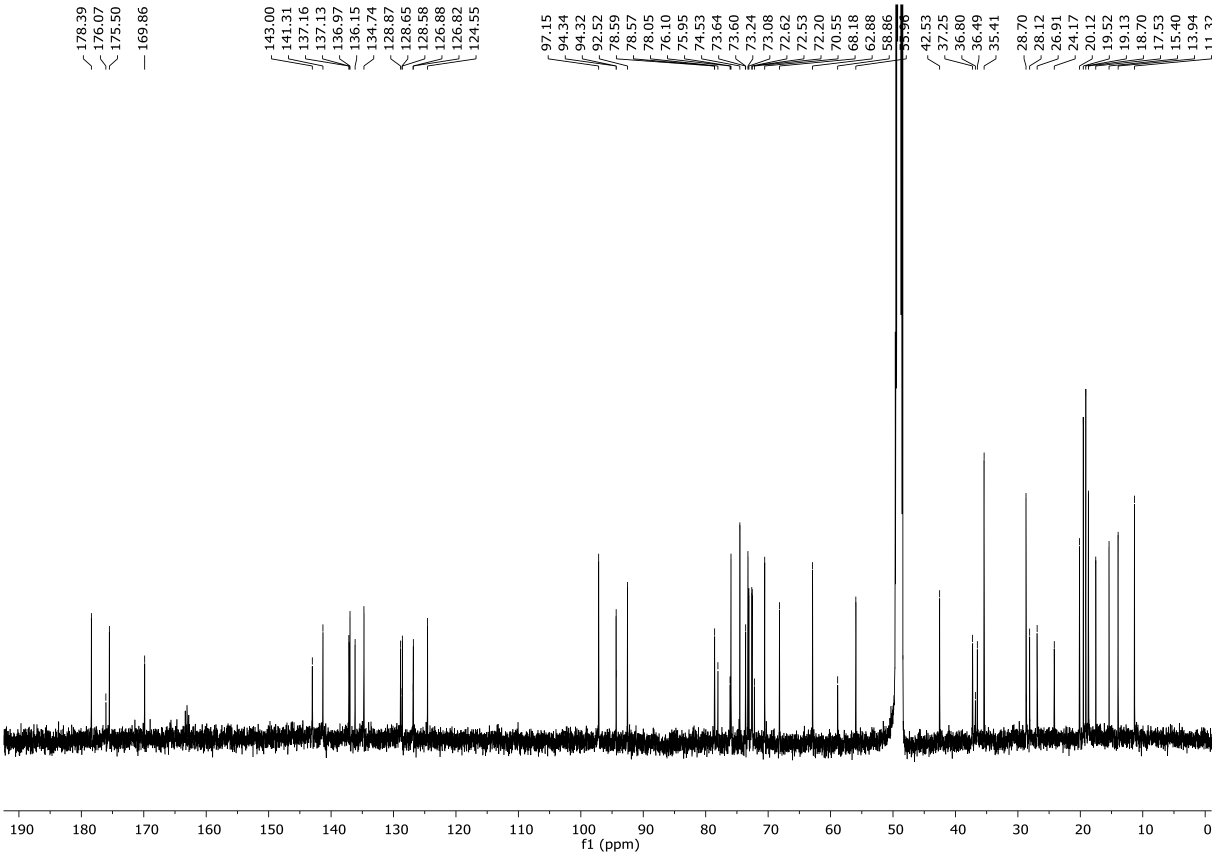

Supplementary Figure 116. ^{13}C NMR (126 MHz, CD_3OD) of **19.**

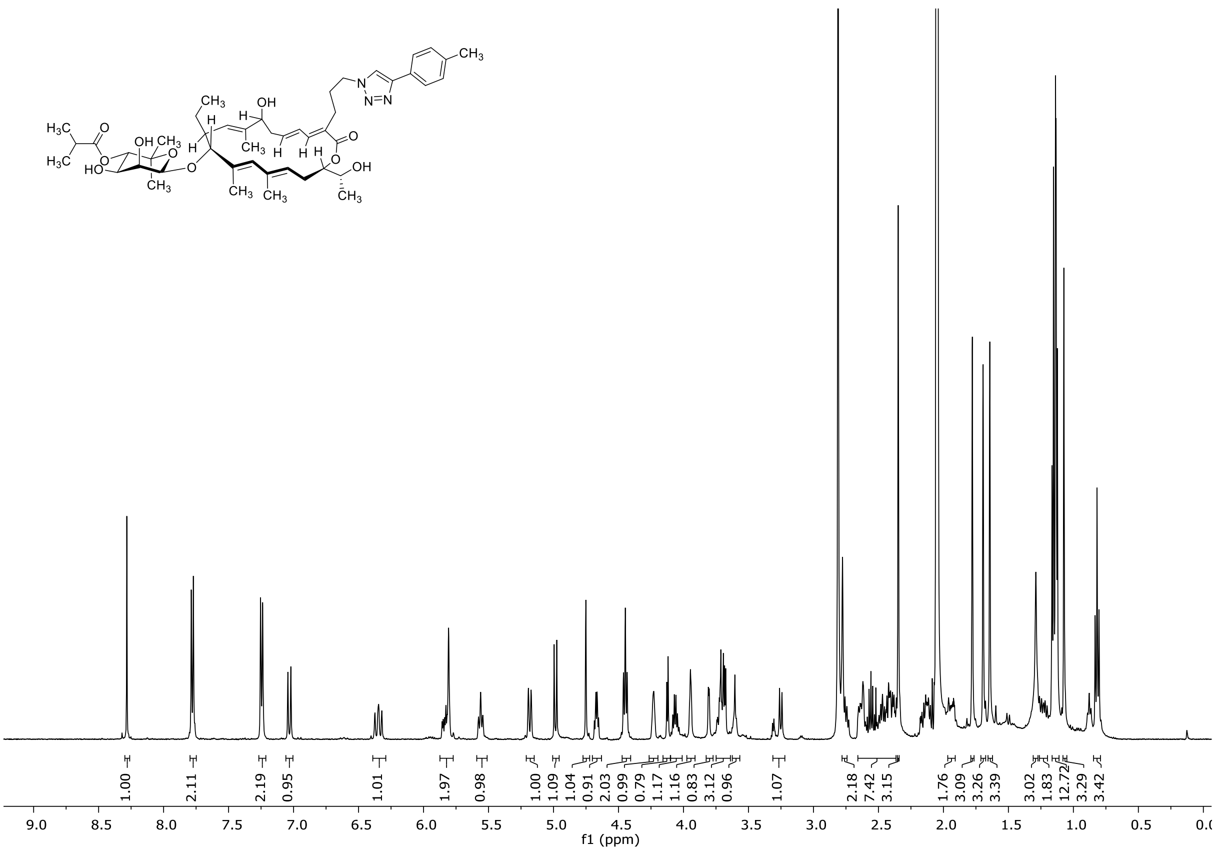

Supplementary Figure 117. ^{1}H NMR (500 MHz, acetone-d_6) of **20.**

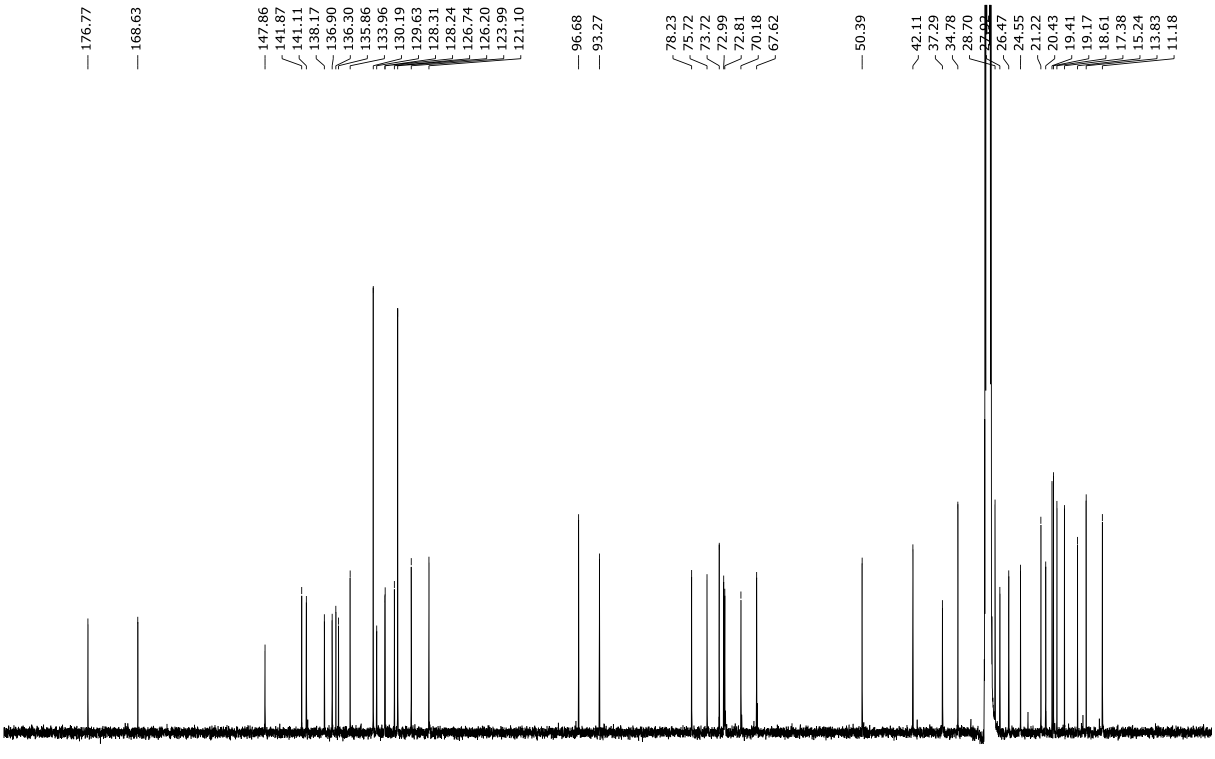

Supplementary Figure 118. ^{13}C NMR (126 MHz, acetone-d_6) of **20.**
